# Impact of an INtervention to increase MOBility in older hospitalized medical patients (INTOMOB): Study protocol for a cluster randomized controlled trial

**DOI:** 10.1186/s12877-023-04285-3

**Published:** 2023-10-31

**Authors:** Blandine Mooser, Dominique Bergsma, Fabian D. Liechti, Christine Baumgartner, Jenny Gentizon, Marie Méan, Maria M. Wertli, Marco Mancinetti, Joachim Schmidt-Leuenberger, Carole E. Aubert

**Affiliations:** 1 Department of General Internal Medicine, Inselspital, Bern University Hospital, University of Bern, Bern, Switzerland; 2 Institute of Primary Health Care (BIHAM), University of Bern, Bern, Switzerland; 3 Institute of Higher Education and Research Healthcare, Lausanne University Hospital and University of Lausanne, Lausanne, Switzerland; 4 Department of Medicine, Internal Medicine, Lausanne University Hospital, University of Lausanne, Lausanne, Switzerland; 5 Department of Internal Medicine, Baden Cantonal Hospital, Baden, Switzerland; 6 Department of General Internal Medicine, Fribourg Cantonal Hospital, Fribourg, Switzerland; 7 Department of Physiotherapy, Inselspital, Bern University Hospital, Bern, Switzerland

**Keywords:** Hospital mobility, Exercise, Physical activity, Older adults, Randomized controlled trial

## Abstract

**Background:**

Low mobility during an acute hospitalization is frequent and associated with adverse effects, including persistent functional decline, institutionalization and death. However, we lack effective interventions to improve mobility that are scalable in everyday practice. The INTOMOB trial – INtervention to increase MOBility in older hospitalized medical patients – will test the effect of a multilevel intervention to improve mobility of older hospitalized patients on functional mobility.

**Methods:**

The INTOMOB multicenter superiority parallel cluster randomized controlled trial will enroll in total 274 patients in Swiss hospitals. Community-dwelling adults aged ≥ 60 years, admitted to a general internal medicine ward with an anticipated length of hospital stay of ≥ 3 days, will be eligible for participation. Unit of randomization will be the wards. A multilevel mobility intervention will be compared to standard of care and target the patients (information and exercise booklets, mobility diary, iPad with exercise videos), healthcare professionals (e-learning, oral presentation, mobility checklist), and environment (posters and pictures on the wards). The primary outcome will be life-space level, measured by the University of Alabama at Birmingham Study of Aging Life-Space Assessment (LSA), at 30 days after enrollment. The LSA is a measure of functional mobility, i.e., how far participants move from bedroom to outside town. Secondary outcomes include, among others, LSA at 180 days, mobility and falls during hospitalization, muscle strength at discharge, and falls, emergency room visits, readmissions, and death within 180 days.

**Discussion:**

This study has the potential to improve outcomes of older hospitalized patients through an intervention that should be scalable in clinical practice because it fosters patient empowerment and does not require additional resources. The tools provided to the patients can help them implement better mobility practices after discharge, which can contribute to better functional outcomes. The choice of a functional patient-reported outcome measure as primary outcome (rather than a “simple” objective mobility measure) reinforces the patient-centeredness of the study.

**Trial registration:**

clinicaltrials.gov (NCT05639231, released on December 19 2022); Swiss National Clinical Trial Portal (SNCTP000005259, released on November 28 2022).

**Supplementary Information:**

The online version contains supplementary material available at 10.1186/s12877-023-04285-3.

## Introduction

### Background and rationale

Up to 80% of patients are able to ambulate independently during an acute hospitalization, while bed rest is not indicated in up to 60% of bed rest episodes [1, 2]. However, low mobility of hospitalized patients is a widespread problem: 83% of hospitalization time is spent in bed, and only 3% walking or standing [1, 2]. Low hospital mobility is associated with cascading adverse effects, including muscle atrophy and contractures, bone loss, falls, delirium, depression, constipation, prolonged length of hospital stay, disability, institutionalization, and death [1–5]. Only 30% of patients with functional decline during hospitalization recover within one year, while two thirds die or are institutionalized [6, 7]. Nevertheless, higher hospital mobility was associated with better outcomes in older medical patients, such as fewer readmissions and institutionalizations [8–11].

The consequences of low hospital mobility therefore impose a burden on patients, healthcare systems, and society, and constitute a priority to address. However, previous studies that focused on older patients during an acute medical hospitalization present several limitations: most were conducted in a single center, assessed only mobility (and not functional / patient-relevant) outcomes, did not follow patients after hospital discharge, or were not randomized [8–14]. Moreover, they often incompletely considered barriers and facilitators to mobility or required additional resources [10, 11], rendering them not easily scalable in everyday practice.

We thus still need to provide clinicians and patients with a scalable and effective way to improve older patient mobility on general internal medicine wards with the goal of reducing the adverse consequences of low hospital mobility.

### Objectives

The primary objective of the INTOMOB trial is to test the effect of the INTOMOB intervention, compared to standard of care, on patient functional mobility, assessed by the life-space level, measured by the University of Alabama at Birmingham Study of Aging Life-Space Assessment (LSA), at 30 days after enrollment. The LSA is a measure of functional mobility, i.e., how far participants move from bedroom to outside town. The hypothesis is that patients randomized to the INTOMOB intervention will have a higher LSA at 30 days after enrollment, compared to those randomized to standard of care.

The secondary objectives are to evaluate the effect of the intervention on functioning, quality of life, depression, pressure ulcers, delirium, mobility, muscle strength, fear of/concerns about falling, prescribing of fall-risk increasing drugs, falls, new institutionalization, discharge destination, emergency room visits, readmissions, death, and satisfaction with hospital stay. The acceptability of the intervention for the patients and of the healthcare professionals (HCPs) is also assessed.

### Trial design

This is a multicenter superiority parallel cluster randomized controlled trial with a 180-day follow-up. The randomization occurs at the ward level (= clusters), while data are analyzed at individual patient level. The trial includes four assessments: baseline, discharge (-1 to +2 days), 30 ± 5 days (D30) after enrollment, and 180 ± 5 days (D180) after enrollment. The primary outcome is assessed at D30, while secondary outcomes are assessed at discharge, D30 and/or D180. A cluster design is chosen because the tested intervention targets not only the patients, but also the HCPs and the hospital environment (wards). The trial was preceded by a pilot study that assessed the feasibility and acceptability of the study intervention and procedures that were then adapted as required [15].

## Methods

This study protocol is presented in accordance with the SPIRIT-Outcomes 2022 checklist [16], and corresponds to the last version (version 5.0) submitted to the ethical committee, dated from April 19, 2023.

### Study setting

The INTOMOB trial is conducted on acute general internal medicine wards of three hospitals, covering different linguistic regions of Switzerland (and thus representing cultural differences): Bern University Hospital (mostly German-speaking, with 130 general internal medicine beds), Baden Cantonal Hospital (German-speaking, with 123 general internal medicine beds) and Fribourg Cantonal Hospital (mostly French-speaking, with 130 general internal medicine beds). Of note, the pilot study that preceded the trial was conducted in Tiefenau Hospital in Bern instead of Baden Cantonal Hospital. The site change is due to an unforeseen closure of Tiefenau Hospital.

The multicenter approach is chosen to increase generalizability of findings by covering different linguistic and cultural regions and hospitals of different sizes and with various already existing practices regarding hospital mobility. Only general internal medicine wards are included to ensure patients are comparable, and because other units (e.g., orthopedics, neurology) often have specific mobilization protocols that could interfere with the intervention.

### Study population and eligibility criteria

Patient inclusion criteria are: age ≥ 60 years, being ambulatory during the two weeks preceding admission (self-report), community-dwelling for at least the 30 days prior to enrollment, understanding French or German, and planned length of hospital stay ≥ 3 days after enrollment. Exclusion criteria are: medical contraindication to walk, wheelchair-bound, end-of-life, severe psychiatric disorder (severe depression, schizophrenia, psychosis), delirium (according to the Confusion Assessment Method) [17], and severe visual impairment. Patients with cognitive impairment (based on clinical judgment) can be included if a proxy can provide consent and actively support the patient during the study. The rationale for including patients with cognitive impairment is that they are also particularly vulnerable to adverse outcomes of low mobility and more likely to stay in bed, however frequently excluded from trials [18]. Including such patients can increase generalizability of study results.

### Intervention and control procedures

The INTOMOB intervention was developed based on barriers and facilitators identified in the literature [7] and through a mixed methods study conducted by the authors with 200 patients recently hospitalized on general internal medicine wards in Switzerland, as well as 142 HCPs (nursing staff, physicians, physiotherapists) working on those wards [19]. The intervention had to be scalable, i.e., not to require additional resources (e.g., more staff) that might prevent its implementation in clinical practice. The INTOMOB intervention and study procedures were adapted based on the results of a pilot study conducted between December 2022 and March 2023. Feasibility, acceptability and scalability of the intervention were assessed during the pilot study, and the comfort, practicability and acceptability of a wrist-worn and an ankle-worn accelerometer were compared [15].

**Fig. 1:**
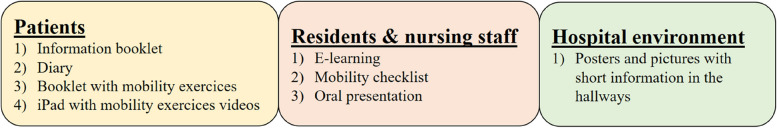
INTOMOB multilevel intervention

The intervention targets are: 1) the patients; 2) the HCPs (nursing staff and medical residents); and 3) the hospital environment (Fig. 1). The preeminent component of the INTOMOB intervention is the patient part, which encompasses: 1) an information booklet that highlights the preservation of functionality and autonomy as the main goal of hospital mobility (Supplement 1); 2) a customizable diary allowing patients to write their mobility goals, results, and difficulties (Supplement 2); 3) a booklet with 29 mobility exercises (in lying, sitting, and standing position) that includes pictures and explanations (Supplement 3); and 4) an iPad 10.2’’ provided to the patients during hospitalization with access to videos for each mobility exercise of the booklet. The videos are stored on a hidden website so that they cannot be found by patients or HCPs of the control group.

The HCP intervention includes an e-learning covering mobility evaluation and recommendations, motivational communication (including two short videos depicturing examples of appropriate and inappropriate patient-HCP communication regarding mobility), barriers and facilitators to hospital mobility, goal setting and evaluation, and presentation of the INTOMOB intervention (e-learning as PowerPoint in Supplement 4a-b). Completion of the e-learning is monitored by the investigators to ensure adherence. The study is presented regularly by the study investigators via a PowerPoint and oral presentation to the HCPs (Supplement 5). In addition, a mobility checklist (Supplement 6) is provided to the nursing staff as a pocket card, hung in resident and nursing offices and in the wards, and placed on carts, to remind the HCPs to assess and address mobility. Nursing staff is the main focus of the HCP intervention given that it does not change wards like medical residents do. Physiotherapists are not directly targeted by the intervention but are informed about it. They can support the patients with the intervention materials, while not using them in the control group.

The environment intervention is designed to foster mobility by making hallways more welcoming to patients. We hang up in the hallways posters on topics of interest to older persons (sleep, nutrition, hospital staff, mobility at hospital and after hospitalization, walking aids, polypharmacy, famous people), as well as pictures of landscapes, flowers, animals and famous people with brief information on them (Supplement 7a-e).

The intervention is compared to standard of care: patients receive standard of care regarding mobility by nursing staff and physicians, as well as physiotherapy if deemed necessary by hospital staff. HCPs on control wards do not complete the e-learning and do not receive the checklist or the presentation. The environment is not modified on control wards.

### Recruitment and assessments

All patients admitted to the ward are screened by the research team for eligibility based on electronic health records and information from the ward staff. Potentially eligible patients are informed by a research team member and asked for consent to participate. Participants can choose if they additionally agree for data reuse for potential ancillary studies. Given the cluster design, randomization arm is known to the research team before approaching the patients. To preserve blinding, participants of the control wards receive information on the study, but not on the INTOMOB intervention. Those participants are informed orally at the end of follow-up about other aspects of the study. Informed consent forms are available in Supplements 8a-b.

The trial includes four assessments, conducted by the research team: 1) baseline; 2) discharge; 3) D30; 4) D180. The schedule of patient-level assessments is detailed in Table 1. The baseline visit is conducted on the ward at enrollment. The discharge visit is conducted on the ward within one day prior to discharge. In case discharge is not well-planned, items not requiring patient examination can be completed by phone call (or on-site in case of a transfer within the hospital) within two days after discharge, to avoid delaying discharge. D30 and D180 assessments are conducted by phone with the study participants or, if not directly reachable, with a contact provided by the patients or with their general practitioner. For patients with cognitive impairment, assessments are conducted with the proxy.

**Table 1:** Schedule of patient-level assessments [17, 20–30]

**Assessments**	**Time points**			
	**Baseline **	**Discharge **	**D30 **	**D180**
Age, sex, weight, height, vital parameters	X			
Home support, health insurance	X			
Mobility aid at admission	X			
Admission date, mode (elective/urgent), hospital, ward	X			
Hospitalization(s) in the last 180 days	X			
Nutritional status (NRS) [20]	X			
Life-space level (UAB Study of Aging LSA or LSA-IS) [21, 22]	X		X	X
ADLs (Barthel Index) [23]	X		X	X
IADLs (Lawton Index) [24]	X		X	X
Quality of life (EQ-5D-5L) [25]	X		X	X
Depression (PHQ-2) [26]	X		X	X
Delirium during hospitalization (CAM) [17]		X		
Pressure ulcer (NPIAP/EPUAP classification) [27]	X	X		
Mobility (DEMMI) [28]	X	X		
Lower-limb and hand-grip muscle strength	X	X		
Step count & level of activity*	X	X		
Fear of / concerns about falling (FES-I) [29]	X	X	X	X
Medications	X	X**	X	X
Relevant comorbidities / Diagnoses	X	X	X	X
Intervention acceptability (intervention group)		X		
Satisfaction with hospital stay (Satisfaction questionnaire adapted & simplified) [30]		X		
Perspectives on hospital mobility (Supplement 9)		X		
Falls (number, consequences)***		X	X	X
New institutionalization		X	X	X
Discharge date & destination		X		
Ward change during hospitalization		X		
Medical devices during hospitalization		X		
Emergency room visits and readmissions***			X	X
Date and cause of death			X	X

*Abbreviations*: *ADLs* Activities of Daily Living, *CAM* Confusion Assessment method, *DEMMI* De Morton Mobility Index, *EPUAP* European Pressure Ulcer Advisory Panel, *FES-I* Falls Efficacy Scale International, *IADLs* Instrumental Activities of Daily Living, *LSA-IS* Life-Space Assessment in Institutionalized Settings, *NPIAP* National Pressure Injury Advisory Panel, *NRS* Nutritional Risk Screening, *PHQ-2* Patient Health Questionnaire-2, *UAB Study of Aging LSA* University of Alabama Study of Aging Life-Space Assessment

X = means that the assessment is conducted at that time point; Empty boxes = means that the assessment is not conducted at that time point

^*^ Participants on both control and intervention wards are asked to wear a wrist-worn ActiGraph [31] accelerometer 24/7 to measure mobility

^**^ Includes medications during hospitalization and at discharge

^***^ To minimize recall bias, participants receive at discharge a diary to record falls, emergency room visits and hospitalizations during follow-up. Number, date and first diagnosis of emergency rooms visits and readmissions are assessed

Mobility is objectively assessed in both trial arms using the ActiGraph wrist-worn accelerometer during hospitalization [31]. This device was chosen following feedback of patients and HCPs who participated in the pilot study.

### Study outcomes

**Fig. 2:**
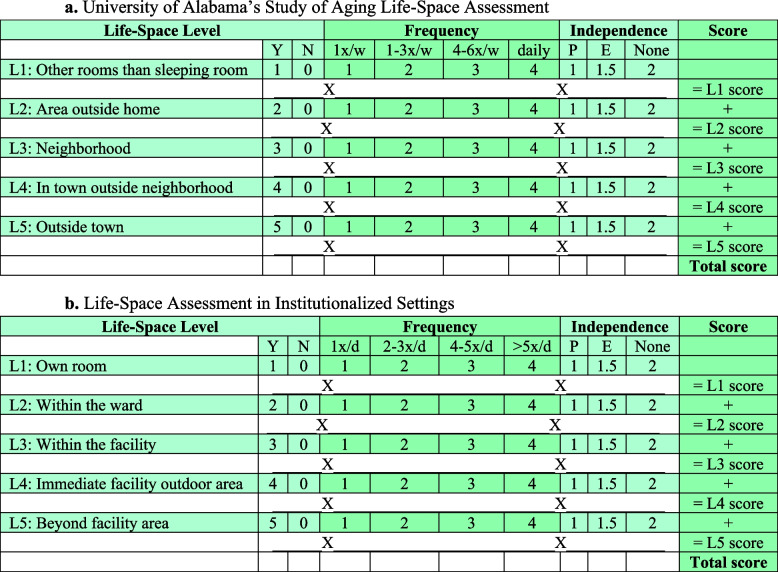
**a** University of Alabama’s Study of Aging Life-Space Assessment [21]. **b.** Life-Space Assessment in Institutionalized Settings [22]. Abbreviations: E, walking equipment assistance; L, level; N, no; P, personal assistance; d, day; w, week; X, multiplied by; x, times; Y, yes

The primary outcome is life-space level at D30 measured by the LSA [21] or the LSA in Institutionalized Settings (LSA-IS) for patients living in an institution (hospital, rehabilitation, nursing home, other institution) [22]. The LSA is a self-reported assessment of mobility capacity and frequency, as well as mobility independence (equipment/personal assistance), over the last four weeks (Table 1 Fig. 2a). It is a validated and comprehensive tool with good test–retest reliability (interclass correlation 0.96) to evaluate the whole continuum of mobility, reflecting functional mobility [21]. The LSA can be easily used during a phone interview and correlates closely with physical functioning [32–38]. It has been used in several studies on mobility, allowing future comparison [13]. The LSA-IS is a validated adaptation of the LSA assessing the same aspects of mobility, but for the last day (Fig. 2b). The LSA and the LSA-IS use a similar scale, ranging from 0 to 120 points, and can be merged. A 5-point change is considered a minimal important change [39].

Secondary outcomes (detailed in Table 2) include functioning, quality of life, depression, pressure ulcer, delirium, mobility, muscle strength, fear of falling, prescribing of fall-risk increasing drugs, falls, new institutionalization, emergency room visits, discharge destination, readmissions, death, satisfaction with hospital stay, and acceptability of the intervention. In addition to patient-level assessments, acceptability of the intervention is assessed through a survey and semi-structured interviews in a sample of HCPs of the intervention group.

**Table 2:** Study outcomes [17, 21–30]

	**Discharge **	**D30 **	**D180**
Life-space level (UAB Study of Aging LSA or LSA-IS) [21, 22]		X*	X
ADLs (Barthel Index) [23]		X	X
IADLs (Lawton Index) [24]		X	X
Quality of life (EQ-5D-5L) [25]		X	X
Depression (PHQ-2) [26]		X	X
Delirium during hospitalization (CAM) [17]			
Pressure ulcer (NPIAP/EPUAP classification) [27]	X		
Mobility (DEMMI) [28]	X		
Lower-limb and hand-grip muscle strength	X		
Step count & level of activity during hospitalization			
Fear of / concerns about falling (FES-I) [29]	X	X	X
Fall-risk increasing drugs	X**	X	X
Intervention acceptability (intervention group)	X		
Satisfaction with hospital stay (Satisfaction questionnaire adapted & simplified) [30]	X		
Experience of the intervention	X		
Perspectives on hospital mobility (Supplement 9)		X	
Falls during hospitalization		X	X
Falls after hospitalization		X	X
New institutionalization	X	X	X
Discharge destination	X		
Emergency room visits and readmissions		X	X
Death		X	X

*Abbreviations*: *ADLs* Activities of Daily Living, *CAM* Confusion Assessment method, *DEMMI* De Morton Mobility Index, *EPUAP* European Pressure Ulcer Advisory Panel, *FES-I* Falls Efficacy Scale International, *IADLs* Instrumental Activities of Daily Living, *LSA-IS* Life-Space Assessment in Institutionalized Settings, *NPIAP* National Pressure Injury Advisory Panel, *NRS* Nutritional Risk Screening, *PHQ-2* Patient Health Questionnaire-2, *UAB Study of Aging LSA* University of Alabama Study of Aging Life-Space Assessment

X = means that the outcome is assessed at that time point; Empty boxes = means that the outcome is not assessed at that time point

^*^ Primary outcome

^**^ Includes medications during hospitalization and at discharge

Finally, perspectives on hospital mobility of patients and HCPs of the intervention group are assessed through the survey that was used in the mixed methods study conducted in the preparatory phase of the trial, and assesses determinants of intention and behavior, based on barriers and facilitators to mobility and on the Health Action Process Approach, which specifies the mechanisms leading to an intention and then to a behavior (Supplement 9) [40, 41].

### Randomization

**Fig. 3:**
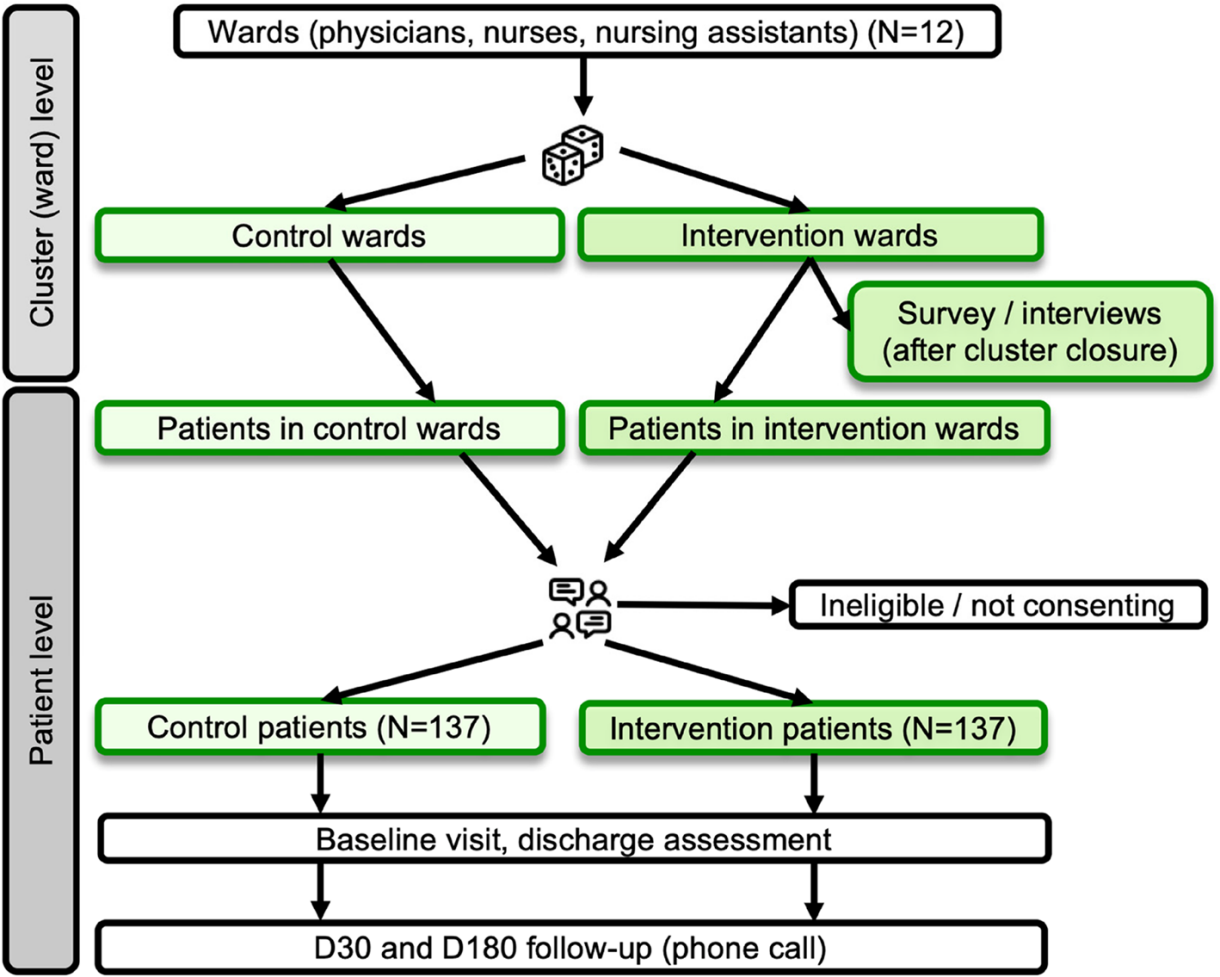
Flow chart of the trial design

Randomization, done by computer through a blinded statistician before start of the trial, occurs at the ward level (= cluster unit) in a 1:1 ratio, with a block size of two and stratification by hospital and by ward size (>30 vs. <=30 beds). HCPs working on, and patients admitted to an intervention ward, receive the intervention procedure. HCPs working on, and patients admitted to a control ward, receive the control procedure. Figure 3 shows a flow chart of the trial design.

### Blinding

The study is partially blinded: complete blinding is difficult due to the nature of the intervention, the communication between HCPs working on different wards (= clusters), and staff rotation. Patient blinding occurs through a differential patient information (see section on recruitment). HCP intervention focuses on nursing staff to minimize contamination bias because medical residents change wards more frequently. Outcome assessors are blinded for D30 and D180 assessments. Data analysis is blinded.

### Sample size

Based on the trial by Brown et al. [13], reporting a 10.9-point difference in the LSA between intervention and control patients hospitalized on medical wards and aged 65 or older (*N* = 100), we calculated that 232 patients (116 in each arm) in 12 clusters (6 in each arm, mean cluster size: 19.3 patients) provides 80% power to detect a 10-point difference in the LSA (standard deviation 19 points) with an intraclass correlation coefficient of 0.05 (based on intraclass correlation coefficients from previous cluster trials) [42], a coefficient of variation for cluster size of 0.5, and a 0.05 two-sided alpha level. We increased the number of patients to 274 (137 in each arm; in 12 clusters in total) to allow for a 15% attrition rate.

### Data management, safety and monitoring

For each participant, an electronic case report form with appropriate coded identification is maintained in a REDCap® (Research Electronic Data Capture) study database, a secure web application for building and managing online surveys and databases. Only authorized research team can access the database with personal login data and specific access rights. The final dataset is accessible to the primary investigator and statisticians.

Adverse events are systematically collected. The investigators comply with all regulations concerning safety set by the ethical committee, and report serious adverse events occurring during the trial within 24 h to the ethical committee, for which a causal relationship with the intervention cannot be excluded. Intervention discontinuation occurs in case of significant risk for a study participant. On-site and central data monitoring is part of the quality control activities implemented for this study and is performed according to separate monitoring plans. All involved parties keep participant data strictly confidential. Audit or monitoring visits by independent monitors or members of the ethical committee are possible at any time point of the trial. Participants are informed about that.

### Statistical analysis plan

Baseline characteristics will be presented using descriptive summary statistics. Continuous variables will be presented as mean with standard deviation or median with quartiles, as appropriate. Categorical variables will be presented as absolute and relative frequencies.

The primary analysis will be an intention-to-treat analysis including all randomized patients, to compare the LSA between intervention and control groups at D30. The secondary analysis will be a per-protocol analysis, excluding patients that violated any eligibility criteria, did not receive the allocated procedure, withdrew consent, or were discharged less than 3 days after enrollment.

The LSA will be analyzed using generalized estimating equations (GEEs) with Gaussian distribution, exchangeable correlation structure, and robust standard errors with small sample correction. This model accounts for the cluster design and for the small number of clusters, and will be adjusted for baseline LSA. Absolute difference between groups will be presented with 95% confidence interval (CI).

Continuous secondary outcomes will be analyzed similarly. Model residuals will be inspected visually for normality using quantile–quantile-plots. For non-normal distributions, we will apply an adequate distribution in GEEs (e.g., negative binomial or gamma). Absolute differences will be presented with 95% CI.

Count outcomes (number of falls/emergency room visits/readmissions) will be analyzed using GEE with negative binomial distribution, log link, exchangeable correlation structure, and robust standard errors. Relative differences will be presented as rate ratio with 95% CI.

Time-to-event outcomes (mortality/time to first readmission/emergency room visit/institutionalization) will be analyzed using Cox regression. To account for clustering, we will use shared frailties for clusters and robust standard errors. Relative differences will be presented as hazard ratio with 95% CI.

Categorical outcomes (discharge destination) will be analyzed using multinomial logistic regression with robust standard errors to account for clustering. Relative differences will be presented as odds ratio with 95% CI.

We will conduct predefined subgroup analyses by age (60–80 vs. > 80 years), sex, length of stay, baseline LSA, baseline activities of daily living/instrumental activities of daily living, and number of comorbidities (cutoffs for continuous variables based on the median).

In sensitivity analyses, we will use generalized linear mixed-effects models instead of GEEs to account for clustering. For outcomes measured at D30 and D180, we will use a repeated-measures mixed-effects model. Should a GEE model not converge, we will use a mixed-effects model for that specific outcome as primary analysis approach. Because cluster randomization may lack the excellent balancing in characteristics between groups seen in individual-level randomization, we will adjust each model for additional pre-defined patient-level variables (age, sex, body mass index, number of limitations in activities of daily living/instrumental activities of daily living, fear of/concerns about falling, and baseline muscle strength) to account for case-mix differences between groups in a sensitivity analysis. Moreover, we will assess imbalances of patient characteristics between groups. If we observe imbalances in covariates not considered in the sensitivity analyses, we will perform additional adjustments to assess the robustness of our results.

If the primary outcome is missing in more than 5% of patients, we will employ multiple imputation in the primary analysis and additionally perform an available case analysis as sensitivity analysis disregarding missing data.

Patient and HCP acceptability of the intervention will be analyzed using descriptive summary statistics for the quantitative questions. The qualitative questions will be analyzed using a mixed deductive and inductive approach. Quantitative and qualitative results will be integrated using joint displays to draw meta-inferences from the mixed data, describing the results as confirming, expanding, or divergent. The results will be used to improve the intervention.

We will analyze the survey on patient and HCP perspectives on hospital mobility as in the preparatory phase. We will assess correlations between variables using Pearson correlation coefficients, and use the variance inflation factor to assess multicollinearity for variables with a correlation coefficient ≥ 0.60. We will conduct hierarchical regressions to assess the determinants of intention and behavior, and present the results as beta-coefficients with 95% CI. We will use delta R-squared (R2) to assess the improvement of the models through the different steps of the hierarchical regression. We will compare the results of intervention patients and HCPs with the results obtained in INTOMOB preparatory phase.

We will report results in accordance with the 2010 Consolidated Standards of Reporting Trials statement extension to cluster-randomized trials [43]. Any deviation from the original statistical plan will be described and justified in the final report. There is no interim analysis planned, i.e., there are no stopping rules on the individual or trial level. We will use Stata/MP (StataCorp LP, College Station, Texas) and R (R Project for Statistical Computing; r-project.org) for quantitative data analysis, and MAXQDA for qualitative data analysis (VERBI Software, Berlin, Germany).

### Potential limitations and risk for bias

Although patient cross-over occurs rarely in the participating hospitals (based on local data, about 1–2% of all admitted patients are transferred between wards), the following strategy has been decided in such cases: if patients move from an intervention to a control or non-randomized ward, they are encouraged to pursue the patient intervention. Conversely, if patients from a control ward move to an intervention ward, they do not receive the patient intervention, but might be influenced by the environment/HCP intervention. However, since the core part of the intervention is the patient intervention and cross-overs remain rare, we deem that it will not introduce a major bias. Any such cross-over will be accounted for in the per-protocol analysis.

Contamination bias is possible due to medical resident rotations. To minimize this, HCPs are asked to avoid talking about the intervention with colleagues of other wards, and the focus of HCP intervention is the nursing staff instead of the medical residents.

Selection bias is possible because randomization arm is known before approaching the patients. To minimize this, the study team is instructed to approach all patients fulfilling inclusion criteria similarly. Recruitment and acceptance rates are monitored and compared between the intervention and control groups. Misbalance of patient characteristics or recruitment numbers are analyzed and discussed with the recruitment team. 

Finally, with hospital stays getting shorter and shorter, patients might not stay long enough to fully benefit from the intervention (although we include only patients with a planned length of stay of ≥ 3 days). However, this represents also real-life practice.

### Ethical considerations and dissemination

The protocol was approved by the local ethical committees (“Ethikkommission für die Forschung am Menschen – Universität Bern”, "Ethikkommission Nordwest- und Zentralschweiz (EKNZ)", "Commission cantonale d'éthique de la recherche sur l'être humain du canton de Vaud (CER-VD") and the study registered before pilot study initiation, on the Clinical Trials Registry platform of the National Institute of Health (NIH) – clinicaltrials.gov (NCT05639231, first release on December 19, 2022) – and on the Swiss National Clinical Trial Portal (SNCTP000005259, first release on November 28, 2022). Any important protocol modifications will be submitted to the ethical committee for approval before implementation.

The results of the trial will be communicated at scientific meetings and in peer-reviewed journals. The guidelines of the International Committee of Medical Journal Editors will be applied to define authorship eligibility for any publication related to the trial [44]. Participants can be informed of the study results after completion of the analyses. Statistical codes and participant-level data can be made available in an anonymized form upon appropriate request.

## Discussion

Low hospital mobility is a widespread problem and a major issue to tackle urgently, in light of its numerous cascading adverse effects, especially detrimental for the older population. Many published studies on older patient hospital mobility have limitations, such as being monocentric or not randomized, lacking follow-up after discharge, lacking assessment of patient-relevant outcomes, or testing an intervention that is not scalable in clinical practice, so they did not lead to practice changes on a larger scale [8–14]. INTOMOB aims to circumvent these limitations by proposing a multicenter, superiority cluster randomized controlled trial testing a multilevel patient-empowering scalable intervention, with a 6-month follow-up and a focus on functional patient-relevant outcomes.

### Strengths, innovation and expected impact

INTOMOB has several strengths. First, it is conducted in three hospitals of different sizes and with different existing practices regarding mobility, and in different linguistic/cultural regions, which will increase generalizability. Second, patients with cognitive impairment, an understudied and vulnerable population [18], can be included in the study. Third, the intervention addresses the barriers and the facilitators to hospital mobility, and was developed to be scalable, which is primordial for successful implementation in everyday practice. Finally, the intervention and study procedures were pilot-tested, allowing for a seamless conduction of the trial.

The main innovation of the INTOMOB intervention is its comprehensive approach by targeting not only the patients, but also the environment and the HCPs, allowing for multifaceted, global patient care. Moreover, the intervention was designed to avoid overloading the HCPs, which should ensure its scalability in everyday clinical practice.

If the INTOMOB intervention is successful, it will provide a way to improve mobility and functional outcomes of older hospitalized patients without requiring additional resources that are not available in clinical practice. The INTOMOB study has the potential to offer a scalable solution for broad-scale implementation of best practices to improve quality of care of the increasing older and vulnerable patient population.

### Supplementary Information

**Additional file 1: Supplement 1.** Information booklet.

**Additional file 2: Supplement 2.** Diary.

**Additional file 3: Supplement 3.** Exercise booklet.

**Additional file 4: Supplement 4.** a. E-learning residents. b. E-learning nursing staff.

**Additional file 5: Supplement 5. **Slides for HCP oral presentation.

**Additional file 6: Supplement 6.** Checklist.

**Additional file 7: Supplement 7.** a. Posters. b. Landscapes - environment intervention. c. Flowers - environment intervention. d. Animals - environment intervention. e. - Famous people - environment intervention.

**Additional file 8: Supplement 8.** a. - ICF -control. b. - ICF - intervention.

**Additional file 9: Supplement 9.** Perspectives on mobility survey.

## Data Availability

Data sharing not applicable to this article as no datasets were generated or analyzed during the current study.

## Abbreviations

CI: Confidence interval
D30: 30 (± 5) Days after enrollment
D180: 180 (± 5) Days after enrollment
GEE: Generalized estimating equation
HCP: Healthcare professional
LSA: Life-space Assessment
REDCap: Research electronic data capture
