# Supplementary material for: Impact of an INtervention to increase MOBility in older hospitalized medical patients (INTOMOB): Study protocol for a cluster randomized controlled trial
Source: BMC Geriatr. 2023 Oct 31;23:705. doi: 10.1186/s12877-023-04285-3 (PMC10617203; doi:10.1186/s12877-023-04285-3)
Supplement: Supplementary file 1 — Additional file 1: Supplement 1. Information booklet. [file 12877_2023_4285_MOESM1_ESM.pdf]

INFORMATION

Moving to maintain autonomy

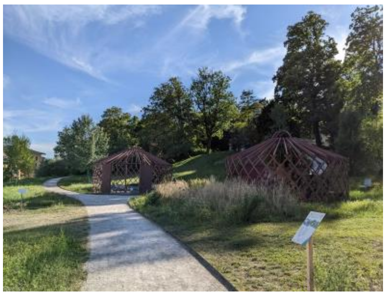

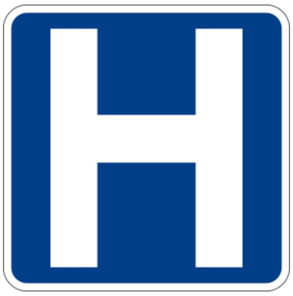

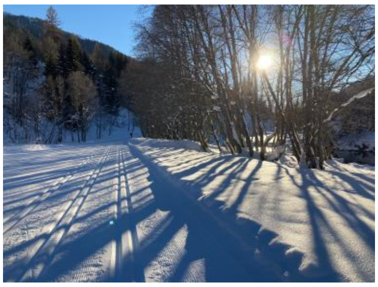

PAGE

CONTENTS

- Why is move at the hospital important 1
- False beliefs 2
- Moving in practice 3
- Moving during daily activities 4
- Walking itineraries - posters 5
- Where to move on the hospital site 6

Why should you move as much as possible during hospitalization?

The **main goal of** movement at the hospital is to **maintain one's autonomy**:
remain as independent as before the hospitalization.

When we are sick, we prefer to stay **in bed** and minimize our efforts. This can have **negative consequences**:

1) Muscle loss: 5% per day spent in bed
2) Loss of appetite - constipation
3) Depression - anxiety - confusion
4) Pneumonia - thrombosis
5) Dizziness - falls
6) Difficulty sleeping at night
7) Extension of hospitalization
8) Loss of autonomy

To avoid this, one must try:

- to move as much as possible,
- to continue with one's **daily activities**,
- to spend as little time as possible in bed.

It's **not easy**. The logbook, exercises and healthcare professionals are there to help you.

In rare cases, it is recommended to avoid moving.
If this is the case, we will inform you.

We recommend that you discuss your questions, fears (e.g. of falling), concerns, progress and goals with your healthcare professionals on a daily basis!

Movement in the hospital: FALSE BELIEFS

If I stay still, I will get better faster.	FALSE! **Recovery** is **longer**.
If I stay still, I'm less likely to fall.	FALSE! The **risk of falling** increases.
If I just stay a few days in bed, nothing will happen.	FALSE! It goes fast: e.g. loss of 5% of muscles in 1 day.
If I stay still, my breathing difficulties will improve.	FALSE! Movement helps with recovery of cardiopulmonary function.
I'm not the athletic type, so I don't need to move.	FALSE! Movement in the hospital ≠ **Sport**. Goal: **Maintenance of autonomy.**
Since I don't leave my home anymore, I don't need to move around in the hospital.	FALSE! You could lose your current **autonomy**.
I have to stay in my room, otherwise I may miss a medical visit / exam.	WRONG! We can **call** you / **tell** you when you need to be there.
Movement prevents healing (of wounds).	FALSE! This is rare. If this is the case, we will inform you.

MOVING IN PRACTICE

What to do?

- ✓ **Activities of daily living**
- ✓ **Sitting up** during **visits**
- ✓ **Exercises** (see specific brochure and videos)
- ✓ **Walks** (see pages 5-6)

How to do it?

- ✓ With **relatives** or other patients
- ✓ With **walking aids** (e.g. walker) if necessary
- ✓ With hospital staff: **ask us**, you are not disturbing us!
- ✓ If you have tubes / catheters: we can explain how to handle them / accompany you.

How often?

- ✓ **Achieve your own goals ≥3 times a day**

When?

- ✓ **Anytime**! Any opportunity is good to take!
- ✓ Please let us **know** when you leave the unit so we can call you back if necessary.
- ✓ If you need to limit your movement, we will inform you.

MOVEMENT THROUGH DAILY ACTIVITIES

Movement helps maintain autonomy. Continue to do your **daily activities as much as possible**. Even though it can be demanding, it is important for your recovery and your health! Here are some examples.

Eating at the table!

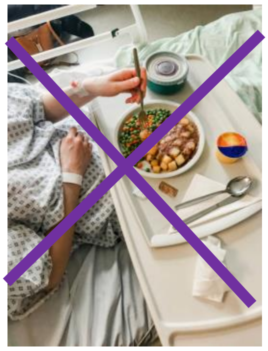
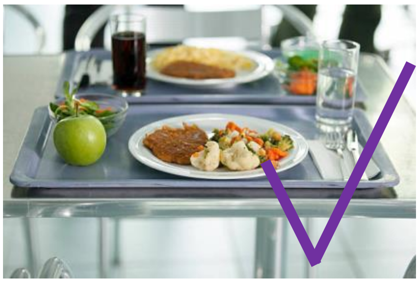

Going to the bathroom!

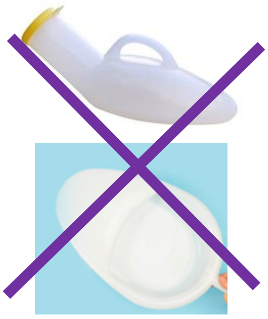
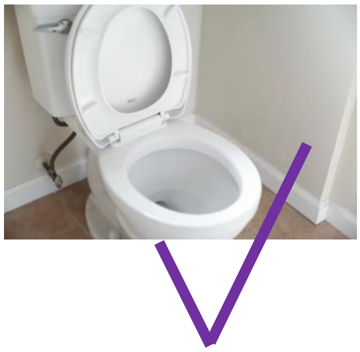

Washing one's self in the shower or at the sink!

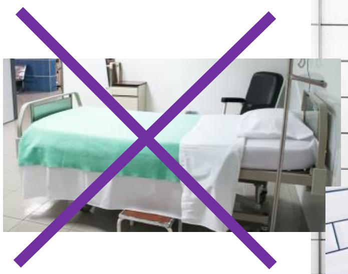
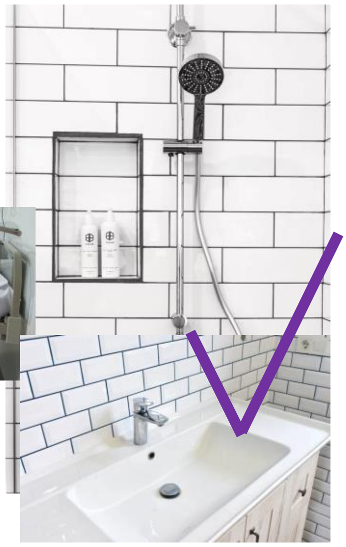

Dressing in your own clothes instead of a hospital gown or pajamas!

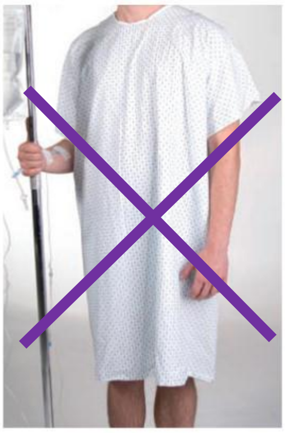
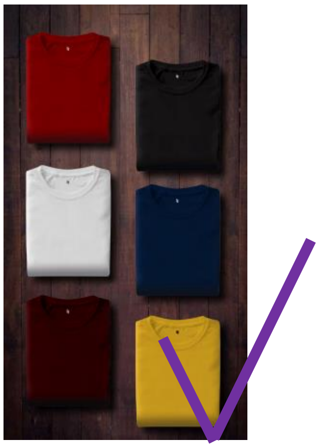

WHERE TO MOVE TO?

- ✓ **Anywhere on the hospital site!**
- ✓ The hallways are **also available to you!** Don’t be afraid to disturb the hospital staff, you are not in the way!

SITE-SPECIFIC PICTURE OF THE HOSPITAL SITE TO BE ADDED HERE

ITINERARIES - POSTERS

We've created **poster routes** for you to give your mobilization a **purpose**.

Go into the hallway to find out more!

WHERE TO MOVE TO AT THE HOSPITAL?

The staff can accompany you and give you more information.

THIS PAGE IS SITE SPECIFIC AND SHOULD INCLUDE A MAP AND/OR PICTURES OF THE HOSPITAL SITE WHERE PATIENTS CAN MOVE

Moving to maintain autonomy

A study from
InselGruppe AG, Bern

Supported by the Swiss National Science Foundation

Contact

PD Dr. med. Carole Elodie Aubert, MD MSc
General Internal Medicine Clinic
Inselspital, Bern University Hospital

Sources of the photos used in the brochure: C. Aubert, www.insel.ch

INTOMOB Study – Version 2.0 – 06.04.2023
