# Supplementary material for: Impact of an INtervention to increase MOBility in older hospitalized medical patients (INTOMOB): Study protocol for a cluster randomized controlled trial
Source: BMC Geriatr. 2023 Oct 31;23:705. doi: 10.1186/s12877-023-04285-3 (PMC10617203; doi:10.1186/s12877-023-04285-3)
Supplement: Supplementary file 2 — Additional file 2: Supplement 2. Diary. [file 12877_2023_4285_MOESM2_ESM.pdf]

INTOMOB

DIARY

Moving to maintain autonomy

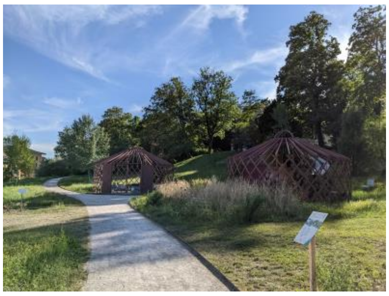

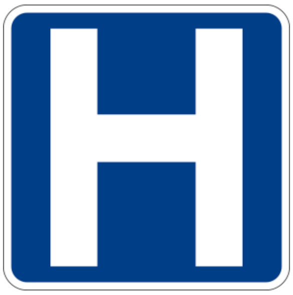

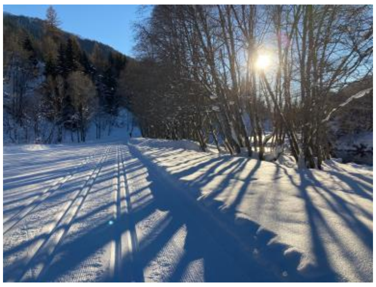

CONTENTS

- Introduction regarding the use of the diary
- Situation before the hospitalization
- Support for your objectives, results, difficulties and needs

WHAT IS THE PURPOSE OF THIS DIARY?

This diary allows you to **write down** your goals **each day**, whether you have achieved them, your difficulties and your needs.

HOW SHOULD ONE USE THIS DIARY?

This diary contains **2 pages for each day**.

The **first page** helps you set your **individual goals**. We recommend that you fill out this page the day beforehand or in the morning. You can check off the proposed goals and/or add others, then indicate how often you want to achieve them, and if you need help (cane, walker, accompaniment, ...).

The **second page** is to record your **results** at the end of the day: What you have achieved, your **difficulties** and your **needs**.

Talk about your goals and results with your healthcare professionals and visitors, and the other patients! This can help you set and achieve your goals.

SITUATION BEFORE HOSPITALISATION

Please note what your situation was like before hospitalization.

This can help you set appropriate goals.

Where did I move at least once a day before hospitalization?

☐ In my home/residence
☐ Outside of my residence
☐ In the village/city where I live

Where did I move at least once a week before hospitalization?

☐ In my home/residence
☐ Outside of my residence
☐ In the village/city where I live

What could I achieve alone?

☐ Going to the bathroom
☐ Putting on my clothes
☐ Personal hygiene
☐ Eating

Did I need help to move around?

☐ YES : Help from somebody else
☐ NO
☐ YES : auxiliary means (walker, cane,…)

OBJECTIVES

DATE ______________________

Here you can find examples of goals. You don't have to achieve them all!
Choose a few goals that suit you.
You can add easier or more difficult objectives!

Objectives	Frequency?	Help?*	Reached?
Doing exercises in bed.			
Sitting up on the side of the bed			
Moving from the bed to the chair			
Moving around the room			
Moving around the corridor			
Going up/down the stairs			
Walking around outside of the unit			
Going to the cafeteria			
Going to the bathroom			
Showering			
Dressing with my own clothes			
Eating at the table			
Doing exercises sitting up			
Doing exercises standing up			

*cane, walker, healthcare professional, relative...

RESULTS

DATE ______________________

What have I accomplished?

☐ All my objectives
☐ Part of my objectives
☐ None of my objectives

What were my obstacles?

☐ Lack of motivation
☐ Lack of time
☐ Fear (of falling, for example)
☐ Lack of strength
☐ Waiting for a medical exam
☐ Dizziness

Other:

____
____
____
____
____

What are my needs?

☐ To be accompanied
☐ Explanations
☐ Auxiliary mean (for ex., cane)

Other:

____
____
____
____

OBJECTIVES

DATE ______________________

Here you can find examples of goals. You don't have to achieve them all!
Choose a few goals that suit you.
You can add easier or more difficult objectives!

Objectives	Frequency?	Help?*	Reached?
Doing exercises in bed.			
Sitting up on the side of the bed			
Moving from the bed to the chair			
Moving around the room			
Moving around the corridor			
Going up/down the stairs			
Walking around outside of the unit			
Going to the cafeteria			
Going to the bathroom			
Showering			
Dressing with my own clothes			
Eating at the table			
Doing exercises sitting up			
Doing exercises standing up			

*cane, walker, healthcare professional, relative...

RESULTS

DATE ____________________

What have I accomplished?

☐ All my objectives ☐ Part of my objectives

☐ None of my objectives

What were my obstacles?

☐ Lack of motivation ☐ Lack of time

☐ Fear (of falling, for example) ☐ Lack of strength

☐ Waiting for a medical exam ☐ Dizziness

Other:

____

____

____

____

____

What are my needs?

☐ To be accompanied ☐ Explanations

☐ Auxiliary mean (for ex., cane)

Other:

____

____

____

____

OBJECTIVES

DATE ______________________

Here you can find examples of goals. You don't have to achieve them all!
Choose a few goals that suit you.
You can add easier or more difficult objectives!

Objectives	Frequency?	Help?*	Reached?
Doing exercises in bed.			
Sitting up on the side of the bed			
Moving from the bed to the chair			
Moving around the room			
Moving around the corridor			
Going up/down the stairs			
Walking around outside of the unit			
Going to the cafeteria			
Going to the bathroom			
Showering			
Dressing with my own clothes			
Eating at the table			
Doing exercises sitting up			
Doing exercises standing up			

*cane, walker, healthcare professional, relative...

RESULTS

DATE ____________________

What have I accomplished?

☐ All my objectives ☐ Part of my objectives

☐ None of my objectives

What were my obstacles?

☐ Lack of motivation ☐ Lack of time

☐ Fear (of falling, for example) ☐ Lack of strength

☐ Waiting for a medical exam ☐ Dizziness

Other:

____

____

____

____

____

What are my needs?

☐ To be accompanied ☐ Explanations

☐ Auxiliary mean (for ex., cane)

Other:

____

____

____

____

OBJECTIVES

DATE ______________________

Here you can find examples of goals. You don't have to achieve them all!
Choose a few goals that suit you.
You can add easier or more difficult objectives!

Objectives	Frequency?	Help?*	Reached?
Doing exercises in bed.			
Sitting up on the side of the bed			
Moving from the bed to the chair			
Moving around the room			
Moving around the corridor			
Going up/down the stairs			
Walking around outside of the unit			
Going to the cafeteria			
Going to the bathroom			
Showering			
Dressing with my own clothes			
Eating at the table			
Doing exercises sitting up			
Doing exercises standing up			

*cane, walker, healthcare professional, relative...

RESULTS

DATE ______________________

What have I accomplished?

☐ All my objectives ☐ Part of my objectives

☐ None of my objectives

What were my obstacles?

☐ Lack of motivation ☐ Lack of time

☐ Fear (of falling, for example) ☐ Lack of strength

☐ Waiting for a medical exam ☐ Dizziness

Other:

____

____

____

____

____

What are my needs?

☐ To be accompanied ☐ Explanations

☐ Auxiliary mean (for ex., cane)

Other:

____

____

____

____

OBJECTIVES

DATE ______________________

Here you can find examples of goals. You don't have to achieve them all!
Choose a few goals that suit you.
You can add easier or more difficult objectives!

Objectives	Frequency?	Help?*	Reached?
Doing exercises in bed.			
Sitting up on the side of the bed			
Moving from the bed to the chair			
Moving around the room			
Moving around the corridor			
Going up/down the stairs			
Walking around outside of the unit			
Going to the cafeteria			
Going to the bathroom			
Showering			
Dressing with my own clothes			
Eating at the table			
Doing exercises sitting up			
Doing exercises standing up			

*cane, walker, healthcare professional, relative...

RESULTS

DATE ____________________

What have I accomplished?

☐ All my objectives ☐ Part of my objectives
☐ None of my objectives

What were my obstacles?

☐ Lack of motivation ☐ Lack of time
☐ Fear (of falling, for example) ☐ Lack of strength
☐ Waiting for a medical exam ☐ Dizziness
Other:

____
____
____
____
____

What are my needs?

☐ To be accompanied ☐ Explanations
☐ Auxiliary mean (for ex., cane)
Other:

____
____
____
____

OBJECTIVES

DATE ______________________

Here you can find examples of goals. You don't have to achieve them all!

Choose a few goals that suit you.

You can add easier or more difficult objectives!

Objectives	Frequency?	Help?*	Reached?
Doing exercises in bed.			
Sitting up on the side of the bed			
Moving from the bed to the chair			
Moving around the room			
Moving around the corridor			
Going up/down the stairs			
Walking around outside of the unit			
Going to the cafeteria			
Going to the bathroom			
Showering			
Dressing with my own clothes			
Eating at the table			
Doing exercises sitting up			
Doing exercises standing up			

*cane, walker, healthcare professional, relative...

RESULTS

DATE ______________________

What have I accomplished?

☐ All my objectives ☐ Part of my objectives
☐ None of my objectives

What were my obstacles?

☐ Lack of motivation ☐ Lack of time
☐ Fear (of falling, for example) ☐ Lack of strength
☐ Waiting for a medical exam ☐ Dizziness

Other:

____
____
____
____
____

What are my needs?

☐ To be accompanied ☐ Explanations
☐ Auxiliary mean (for ex., cane)

Other:

____
____
____
____

OBJECTIVES

DATE ______________________

Here you can find examples of goals. You don't have to achieve them all!
Choose a few goals that suit you.
You can add easier or more difficult objectives!

Objectives	Frequency?	Help?*	Reached?
Doing exercises in bed.			
Sitting up on the side of the bed			
Moving from the bed to the chair			
Moving around the room			
Moving around the corridor			
Going up/down the stairs			
Walking around outside of the unit			
Going to the cafeteria			
Going to the bathroom			
Showering			
Dressing with my own clothes			
Eating at the table			
Doing exercises sitting up			
Doing exercises standing up			

*cane, walker, healthcare professional, relative...

RESULTS

DATE ______________________

What have I accomplished?

☐ All my objectives ☐ Part of my objectives

☐ None of my objectives

What were my obstacles?

☐ Lack of motivation ☐ Lack of time

☐ Fear (of falling, for example) ☐ Lack of strength

☐ Waiting for a medical exam ☐ Dizziness

Other:

____

____

____

____

____

What are my needs?

☐ To be accompanied ☐ Explanations

☐ Auxiliary mean (for ex., cane)

Other:

____

____

____

____

OBJECTIVES

DATE ______________________

Here you can find examples of goals. You don't have to achieve them all!
Choose a few goals that suit you.
You can add easier or more difficult objectives!

Objectives	Frequency?	Help?*	Reached?
Doing exercises in bed.			
Sitting up on the side of the bed			
Moving from the bed to the chair			
Moving around the room			
Moving around the corridor			
Going up/down the stairs			
Walking around outside of the unit			
Going to the cafeteria			
Going to the bathroom			
Showering			
Dressing with my own clothes			
Eating at the table			
Doing exercises sitting up			
Doing exercises standing up			

*cane, walker, healthcare professional, relative...

RESULTS

DATE ____________________

What have I accomplished?

☐ All my objectives ☐ Part of my objectives

☐ None of my objectives

What were my obstacles?

☐ Lack of motivation ☐ Lack of time

☐ Fear (of falling, for example) ☐ Lack of strength

☐ Waiting for a medical exam ☐ Dizziness

Other:

____

____

____

____

____

What are my needs?

☐ To be accompanied ☐ Explanations

☐ Auxiliary mean (for ex., cane)

Other:

____

____

____

____

OBJECTIVES

DATE ______________________

Here you can find examples of goals. You don't have to achieve them all!
Choose a few goals that suit you.
You can add easier or more difficult objectives!

Objectives	Frequency?	Help?*	Reached?
Doing exercises in bed.			
Sitting up on the side of the bed			
Moving from the bed to the chair			
Moving around the room			
Moving around the corridor			
Going up/down the stairs			
Walking around outside of the unit			
Going to the cafeteria			
Going to the bathroom			
Showering			
Dressing with my own clothes			
Eating at the table			
Doing exercises sitting up			
Doing exercises standing up			

*cane, walker, healthcare professional, relative...

RESULTS

DATE ______________________

What have I accomplished?

☐ All my objectives ☐ Part of my objectives

☐ None of my objectives

What were my obstacles?

☐ Lack of motivation ☐ Lack of time

☐ Fear (of falling, for example) ☐ Lack of strength

☐ Waiting for a medical exam ☐ Dizziness

Other:

____

____

____

____

____

What are my needs?

☐ To be accompanied ☐ Explanations

☐ Auxiliary mean (for ex., cane)

Other:

____

____

____

____

OBJECTIVES

DATE ______________________

Here you can find examples of goals. You don't have to achieve them all!
Choose a few goals that suit you.
You can add easier or more difficult objectives!

Objectives	Frequency?	Help?*	Reached?
Doing exercises in bed.			
Sitting up on the side of the bed			
Moving from the bed to the chair			
Moving around the room			
Moving around the corridor			
Going up/down the stairs			
Walking around outside of the unit			
Going to the cafeteria			
Going to the bathroom			
Showering			
Dressing with my own clothes			
Eating at the table			
Doing exercises sitting up			
Doing exercises standing up			

*cane, walker, healthcare professional, relative...

RESULTS

DATE ______________________

What have I accomplished?

☐ All my objectives ☐ Part of my objectives
☐ None of my objectives

What were my obstacles?

☐ Lack of motivation ☐ Lack of time
☐ Fear (of falling, for example) ☐ Lack of strength
☐ Waiting for a medical exam ☐ Dizziness
Other:

____
____
____
____
____

What are my needs?

☐ To be accompanied ☐ Explanations
☐ Auxiliary mean (for ex., cane)
Other:

____
____
____
____

OBJECTIVES

DATE ______________________

Here you can find examples of goals. You don't have to achieve them all!
Choose a few goals that suit you.
You can add easier or more difficult objectives!

Objectives	Frequency?	Help?*	Reached?
Doing exercises in bed.			
Sitting up on the side of the bed			
Moving from the bed to the chair			
Moving around the room			
Moving around the corridor			
Going up/down the stairs			
Walking around outside of the unit			
Going to the cafeteria			
Going to the bathroom			
Showering			
Dressing with my own clothes			
Eating at the table			
Doing exercises sitting up			
Doing exercises standing up			

*cane, walker, healthcare professional, relative...

RESULTS

DATE ______________________

What have I accomplished?

☐ All my objectives
☐ Part of my objectives
☐ None of my objectives

What were my obstacles?

☐ Lack of motivation
☐ Lack of time
☐ Fear (of falling, for example)
☐ Lack of strength
☐ Waiting for a medical exam
☐ Dizziness

Other:

____
____
____
____
____

What are my needs?

☐ To be accompanied
☐ Explanations
☐ Auxiliary mean (for ex., cane)

Other:

____
____
____
____

OBJECTIVES

DATE ______________________

Here you can find examples of goals. You don't have to achieve them all!
Choose a few goals that suit you.
You can add easier or more difficult objectives!

Objectives	Frequency?	Help?*	Reached?
Doing exercises in bed.			
Sitting up on the side of the bed			
Moving from the bed to the chair			
Moving around the room			
Moving around the corridor			
Going up/down the stairs			
Walking around outside of the unit			
Going to the cafeteria			
Going to the bathroom			
Showering			
Dressing with my own clothes			
Eating at the table			
Doing exercises sitting up			
Doing exercises standing up			

*cane, walker, healthcare professional, relative...

RESULTS

DATE ____________________

What have I accomplished?

☐ All my objectives ☐ Part of my objectives

☐ None of my objectives

What were my obstacles?

☐ Lack of motivation ☐ Lack of time

☐ Fear (of falling, for example) ☐ Lack of strength

☐ Waiting for a medical exam ☐ Dizziness

Other:

____

____

____

____

____

What are my needs?

☐ To be accompanied ☐ Explanations

☐ Auxiliary mean (for ex., cane)

Other:

____

____

____

____

OBJECTIVES

DATE ______________________

Here you can find examples of goals. You don't have to achieve them all!
Choose a few goals that suit you.
You can add easier or more difficult objectives!

Objectives	Frequency?	Help?*	Reached?
Doing exercises in bed.			
Sitting up on the side of the bed			
Moving from the bed to the chair			
Moving around the room			
Moving around the corridor			
Going up/down the stairs			
Walking around outside of the unit			
Going to the cafeteria			
Going to the bathroom			
Showering			
Dressing with my own clothes			
Eating at the table			
Doing exercises sitting up			
Doing exercises standing up			

*cane, walker, healthcare professional, relative...

RESULTS

DATE ______________________

What have I accomplished?

☐ All my objectives ☐ Part of my objectives
☐ None of my objectives

What were my obstacles?

☐ Lack of motivation ☐ Lack of time
☐ Fear (of falling, for example) ☐ Lack of strength
☐ Waiting for a medical exam ☐ Dizziness
Other:

____
____
____
____
____

What are my needs?

☐ To be accompanied ☐ Explanations
☐ Auxiliary mean (for ex., cane)
Other:

____
____
____
____

OBJECTIVES

DATE ______________________

Here you can find examples of goals. You don't have to achieve them all!
Choose a few goals that suit you.
You can add easier or more difficult objectives!

Objectives	Frequency?	Help?*	Reached?
Doing exercises in bed.			
Sitting up on the side of the bed			
Moving from the bed to the chair			
Moving around the room			
Moving around the corridor			
Going up/down the stairs			
Walking around outside of the unit			
Going to the cafeteria			
Going to the bathroom			
Showering			
Dressing with my own clothes			
Eating at the table			
Doing exercises sitting up			
Doing exercises standing up			

*cane, walker, healthcare professional, relative...

RESULTS

DATE ____________________

What have I accomplished?

☐ All my objectives ☐ Part of my objectives

☐ None of my objectives

What were my obstacles?

☐ Lack of motivation ☐ Lack of time

☐ Fear (of falling, for example) ☐ Lack of strength

☐ Waiting for a medical exam ☐ Dizziness

Other:

____

____

____

____

____

What are my needs?

☐ To be accompanied ☐ Explanations

☐ Auxiliary mean (for ex., cane)

Other:

____

____

____

____

Moving to maintain autonomy

A study from
Insel Gruppe AG, Bern

Supported by the Swiss National Science Foundation

Contact

PD Dr. med. Carole Elodie Aubert, MD MSc
General Internal Medicine Clinic
Inselspital, Bern University Hospital

Sources of the photos used in the brochure: C. Aubert
