# Supplementary material for: Impact of an INtervention to increase MOBility in older hospitalized medical patients (INTOMOB): Study protocol for a cluster randomized controlled trial
Source: BMC Geriatr. 2023 Oct 31;23:705. doi: 10.1186/s12877-023-04285-3 (PMC10617203; doi:10.1186/s12877-023-04285-3)
Supplement: Supplementary file 3 — Additional file 3: Supplement 3. Exercise booklet. [file 12877_2023_4285_MOESM3_ESM.pdf]

INTOMOB

EXERCISES

Moving to maintain autonomy

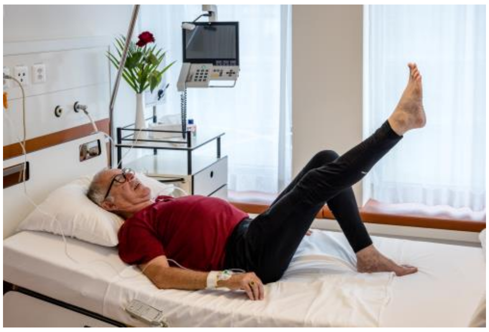
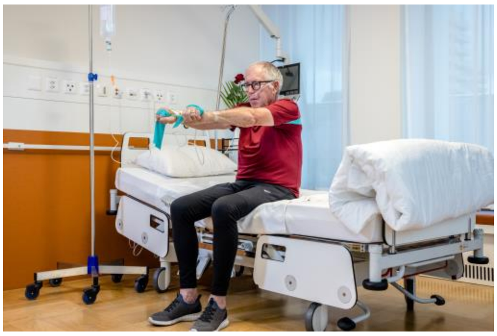
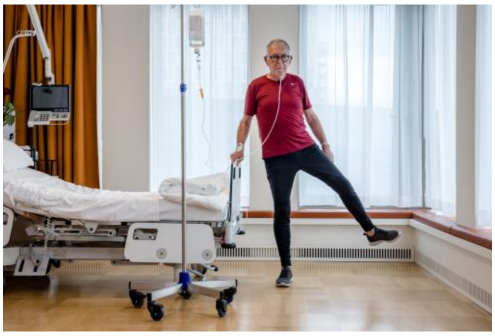

CONTENTS

	PAGE
✓ Introduction	1
✓ Exercises lying down	2-11
✓ Exercises sitting up	12-19
✓ Exercises standing up	20-30

In this brochure you will find exercises that you can do in the hospital and after your hospitalization.

There are exercises for different parts of the body when lying down, sitting or standing.

Choose the exercises and position that best suit your current abilities. This can change every day.

Some exercises can seem too easy or on the opposite too difficult: that's normal!
This can change every day.

The main thing is to move regularly!

All exercises are available in video format on the iPad.

Please ask your healthcare team if you should avoid certain exercises at this time.

If you are in pain or feel unsafe while doing the exercises, talk to your healthcare team.

EXERCICES LYING DOWN

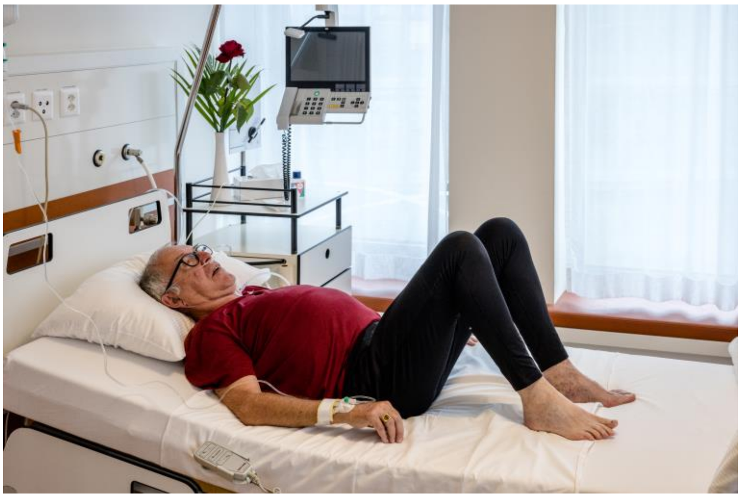

Moving to maintain autonomy

Foot gymnastics (Video 1)

Move your feet rigorously up and down for about a minute, keeping your legs straight. This stimulates blood circulation.

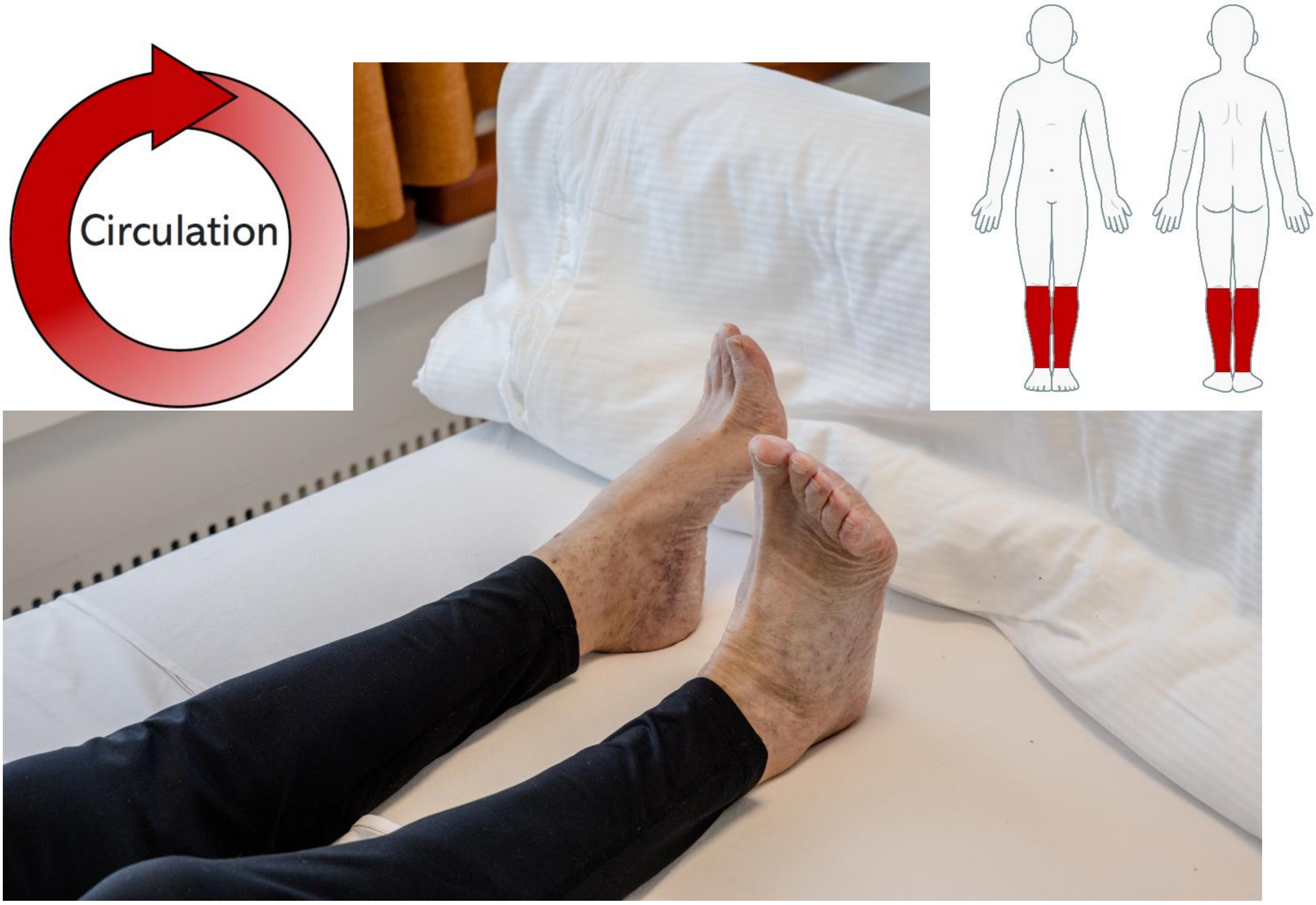

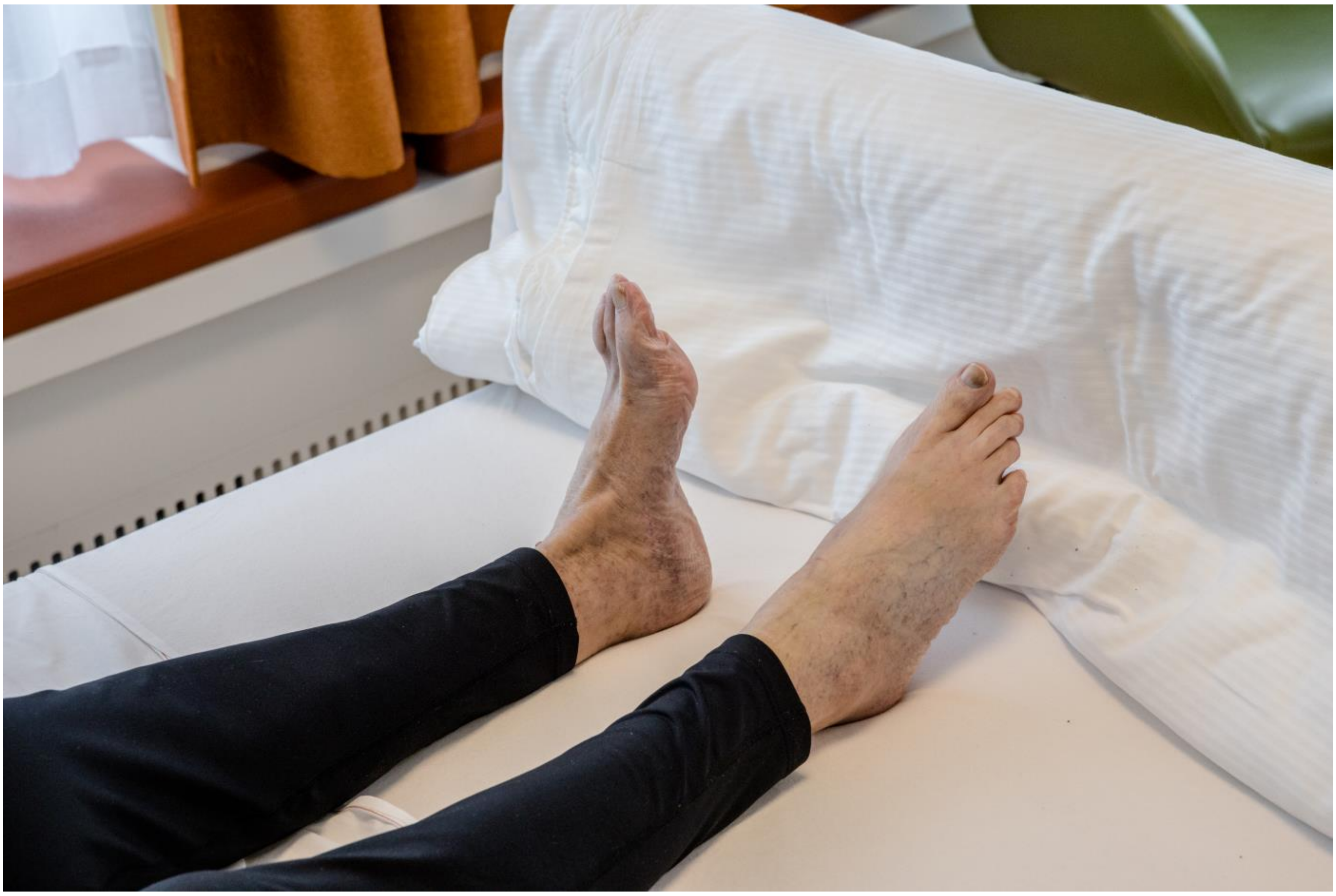

Hand Gymnastics (Video 2)

Alternate fists and fingers stretched rigorously for about a minute, keeping your arms bent.
This stimulates blood circulation.

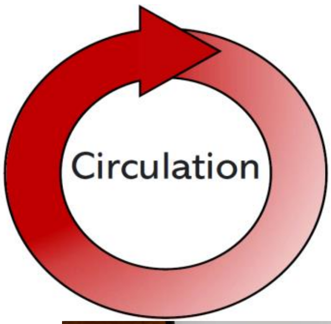

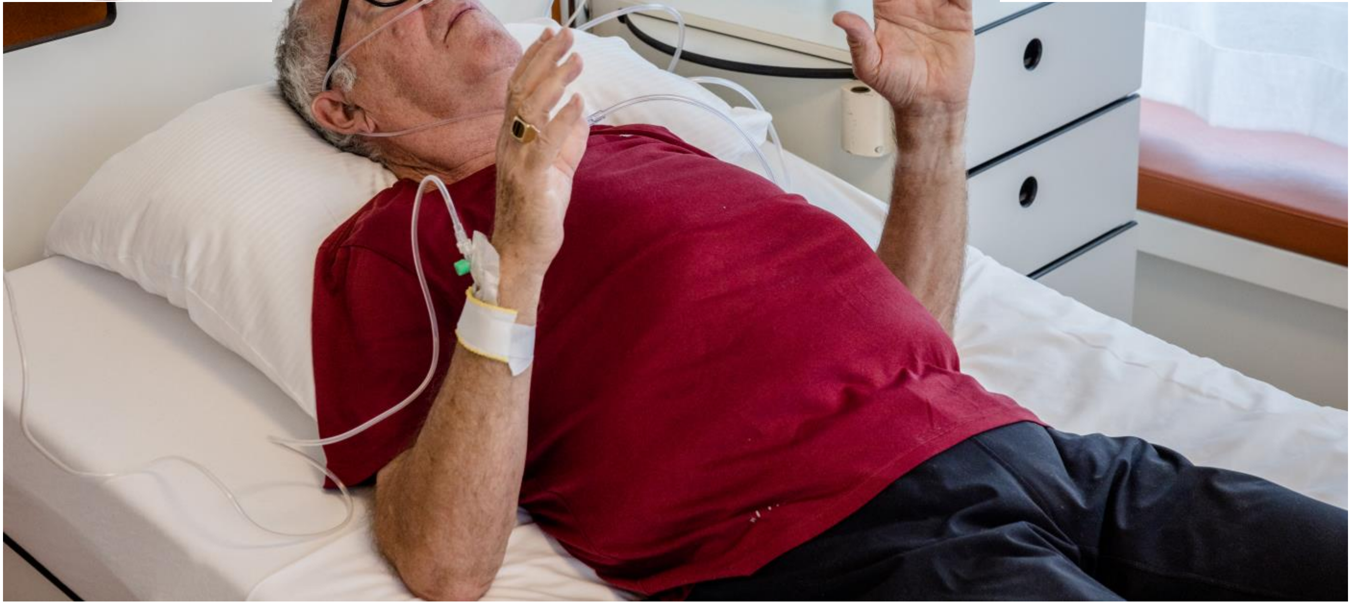

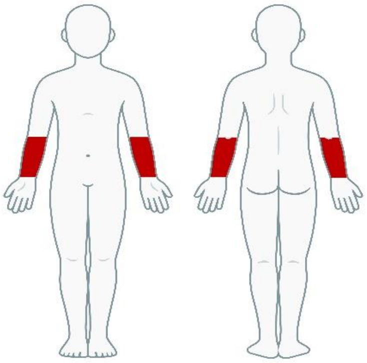

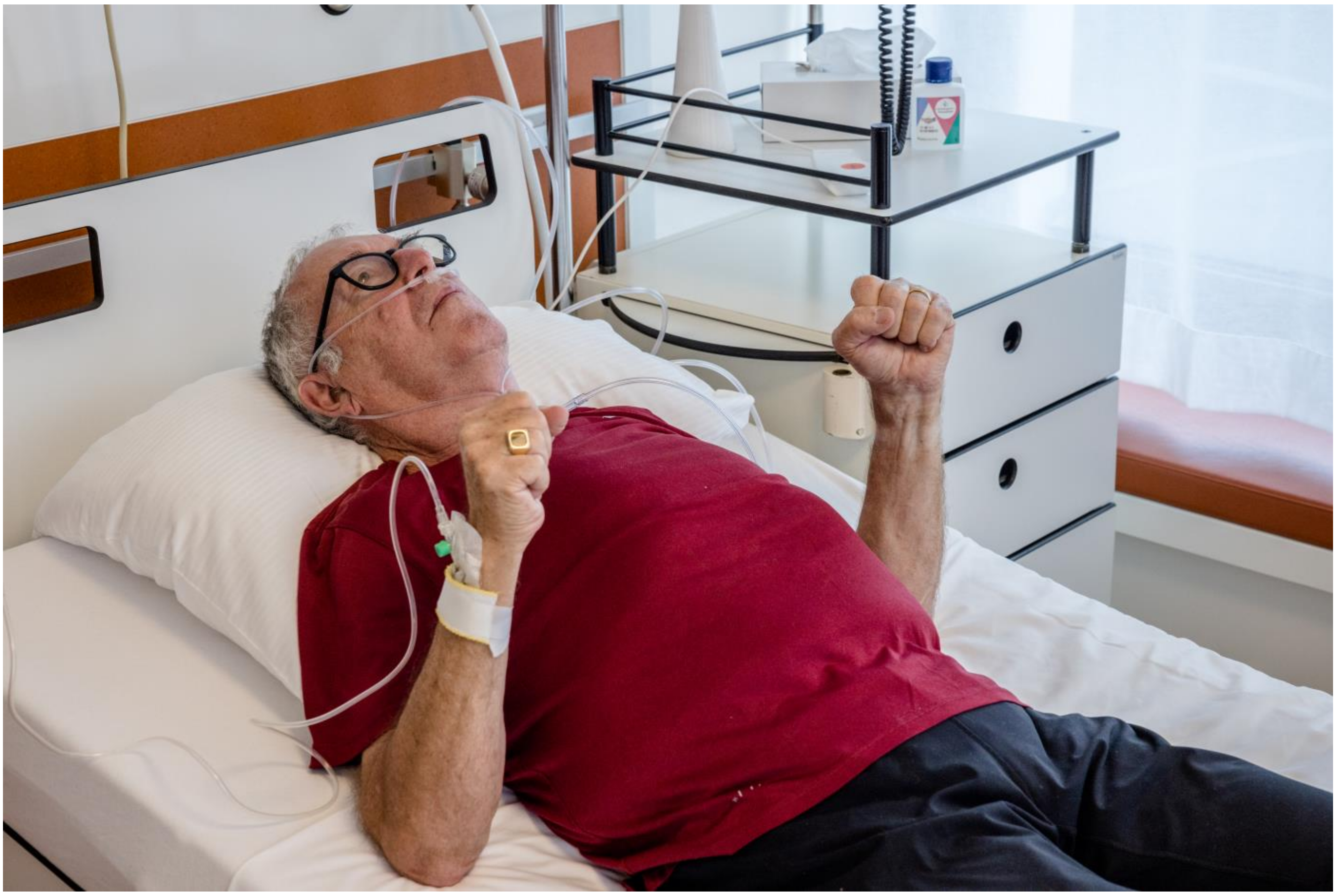

Bridge (Video 3)

Raise your buttocks several times, keeping your legs bent.
The arms may remain bent or extended.
This exercise strengthens different muscles.

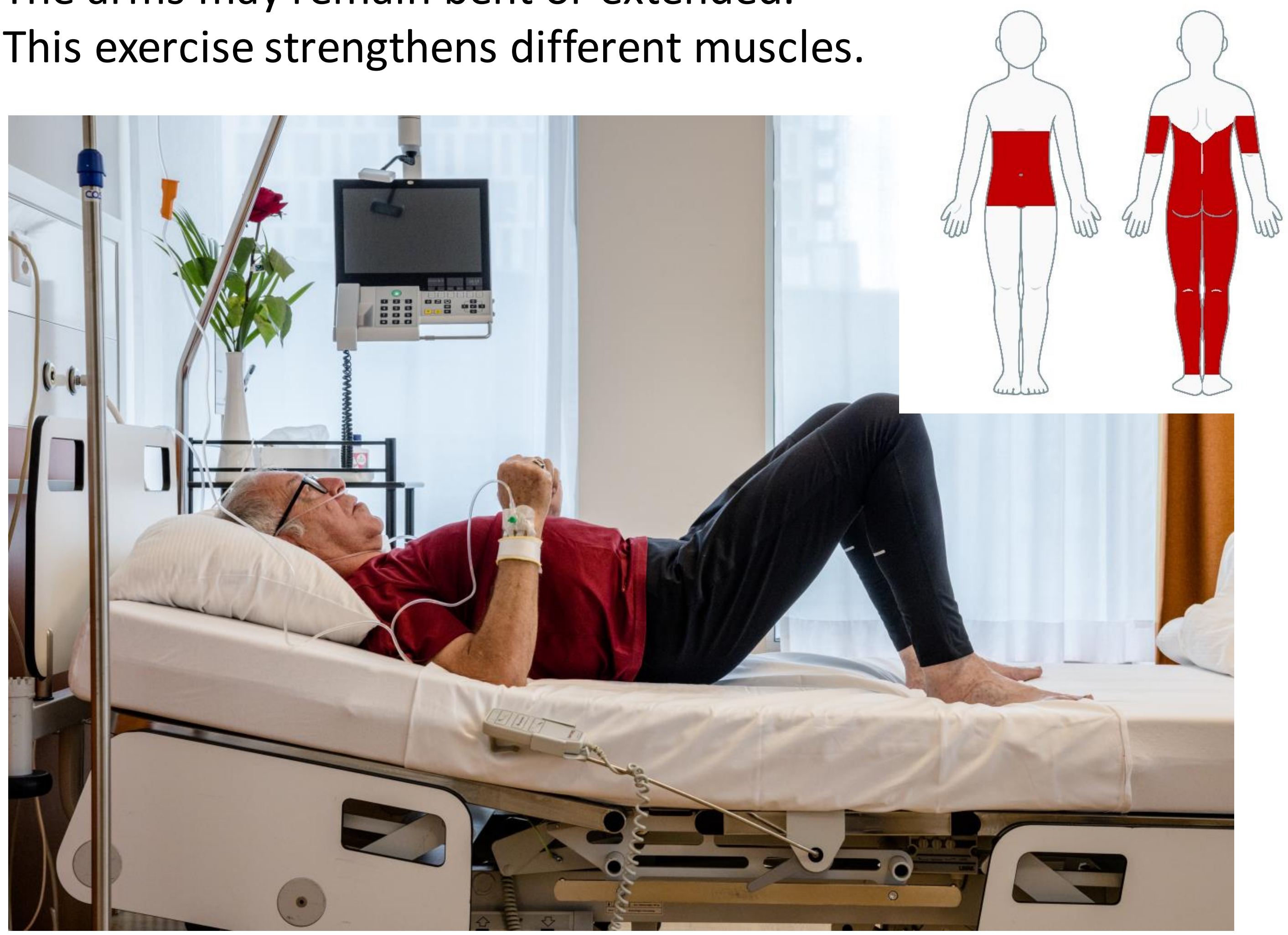

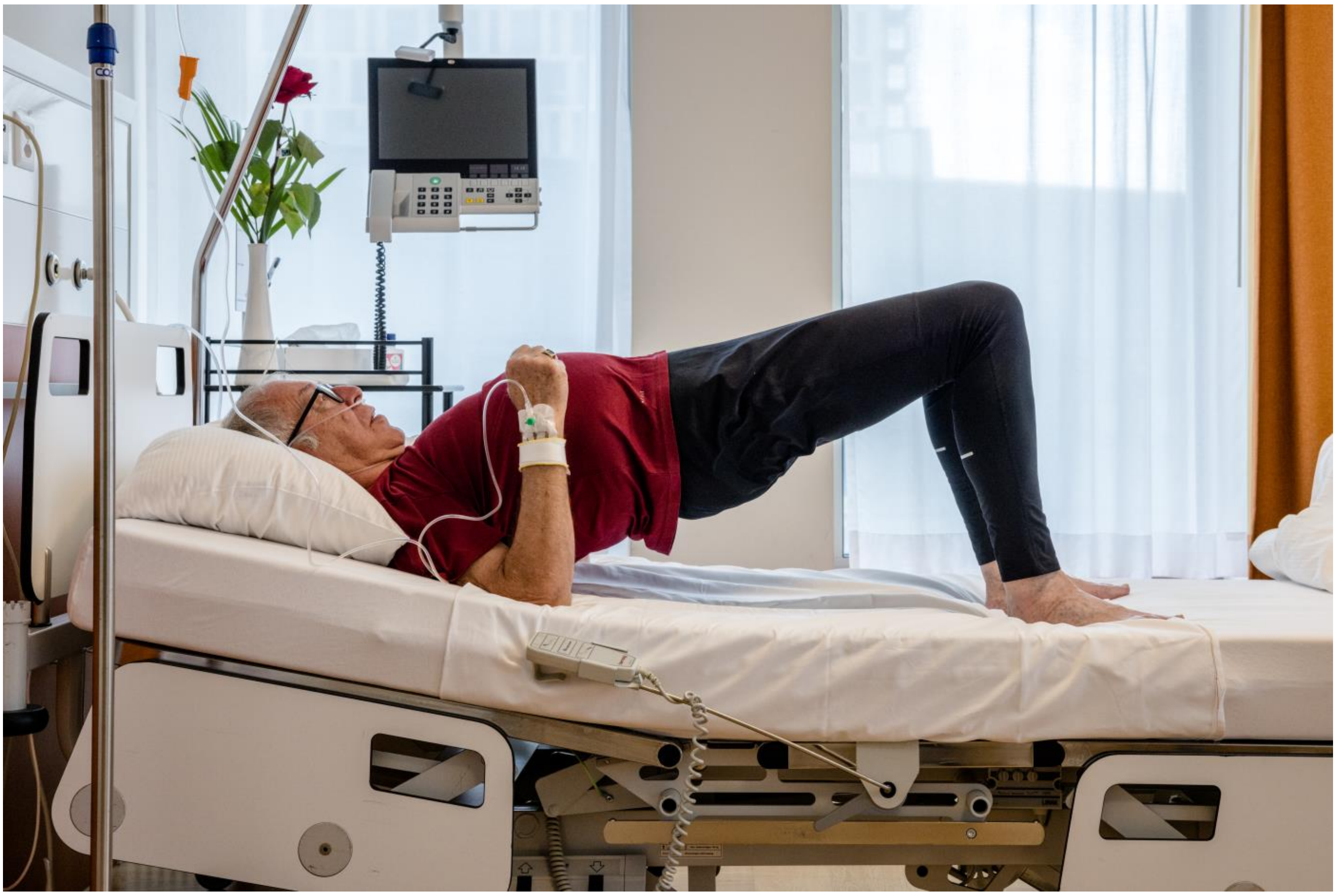

Stretching your legs (Video 4)

Stretch and bend one leg several times, then the other.
This exercise strengthens the thigh muscles.

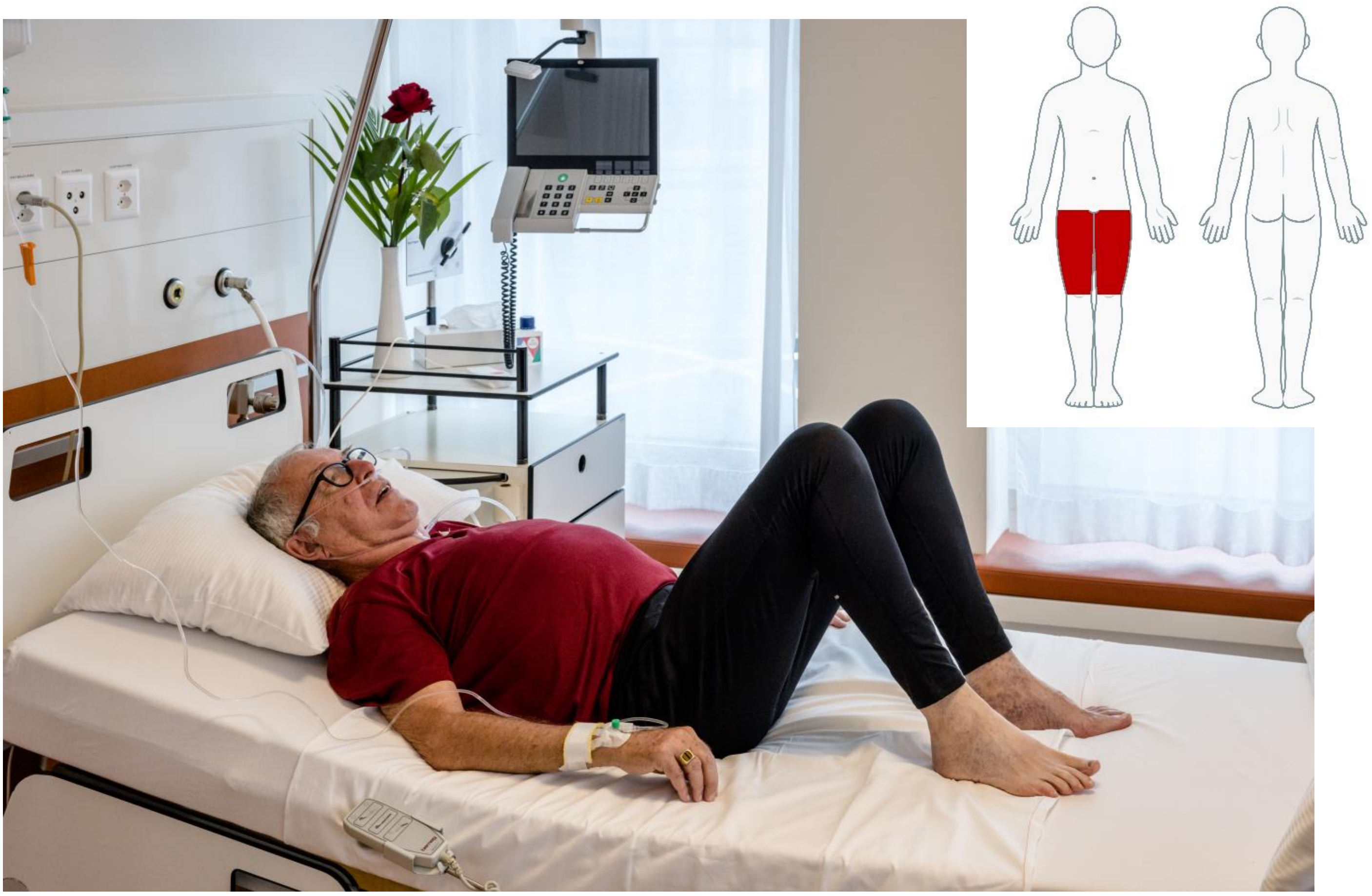

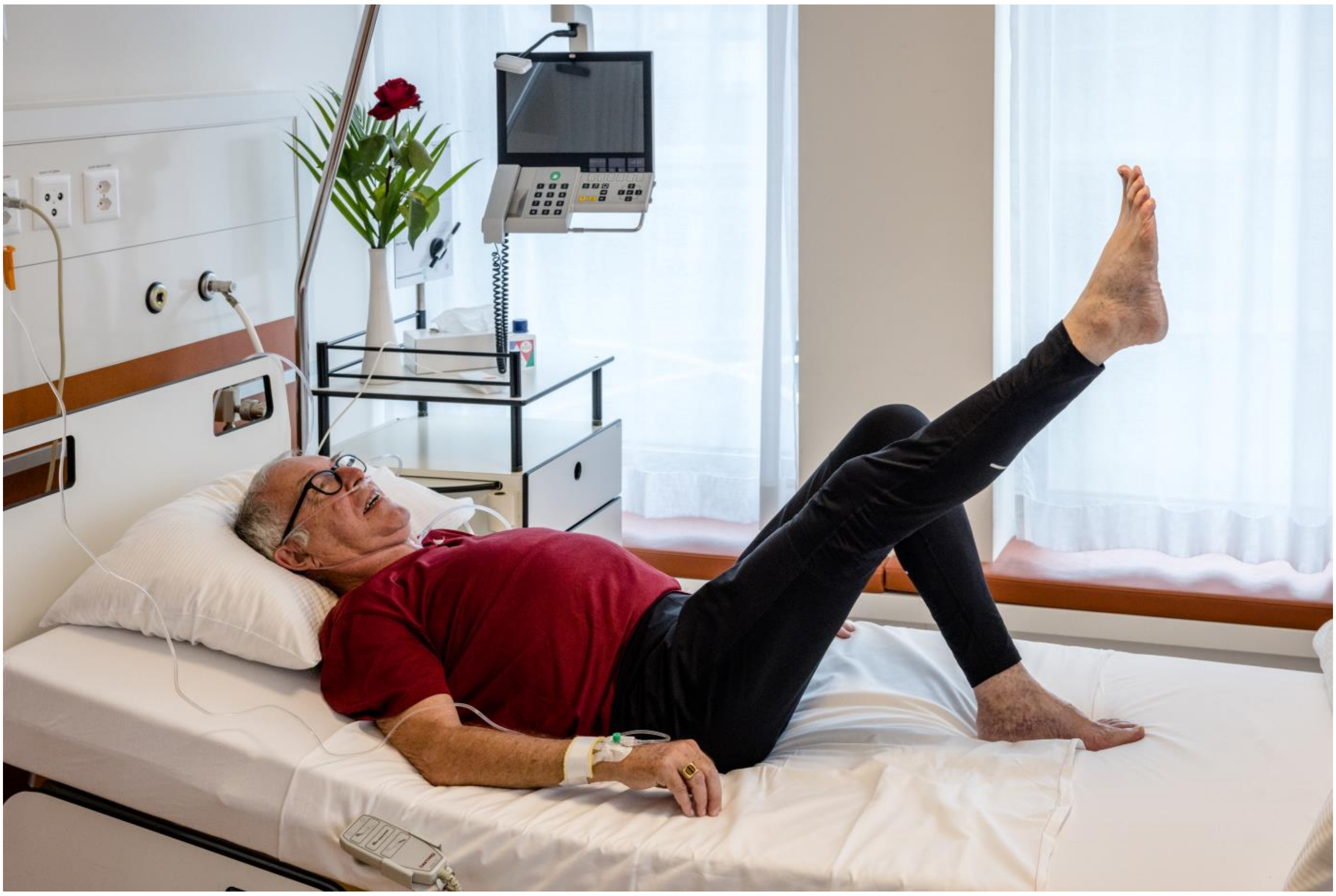

Pressing the knee against the hand (Video 5)

Press one knee against one hand for about a minute with force.
Repeat the exercise with different variations (knee and hand on the same side; opposite knee and hand).
Be sure to train each extremity.
This exercise strengthens the stomach muscles.

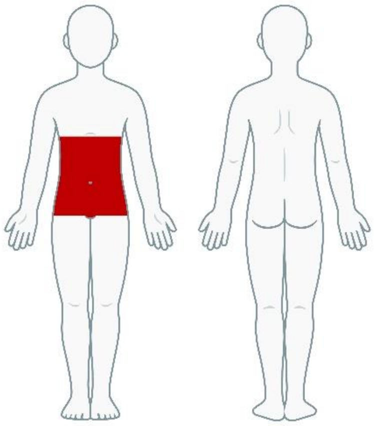

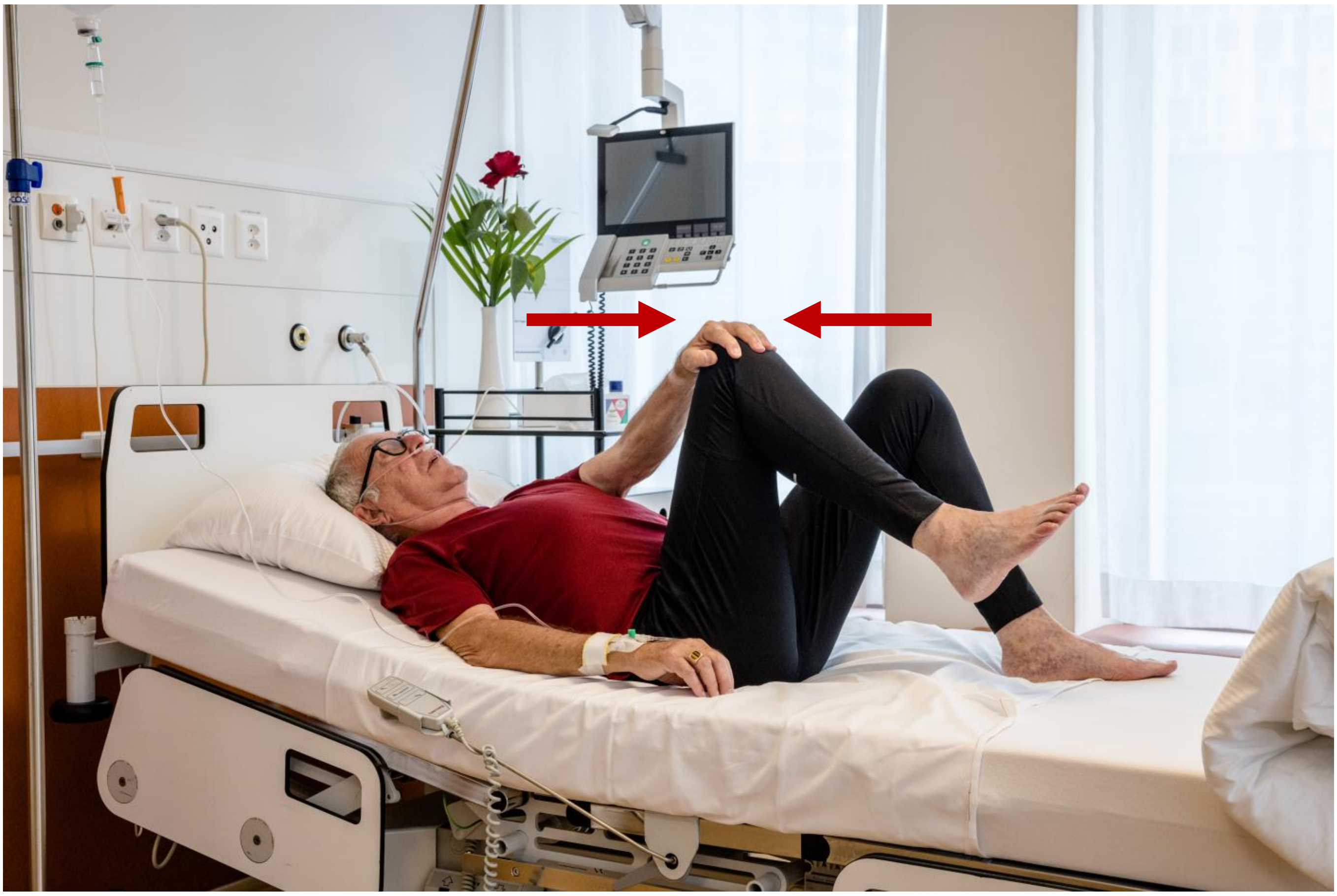

Arm pull with an elastic band (Video 6)

Pull with your arms outstretched several times on an elastic fitness band attached to the bar of the hospital bed.
This exercise strengthens the muscles of the shoulders and trunk.

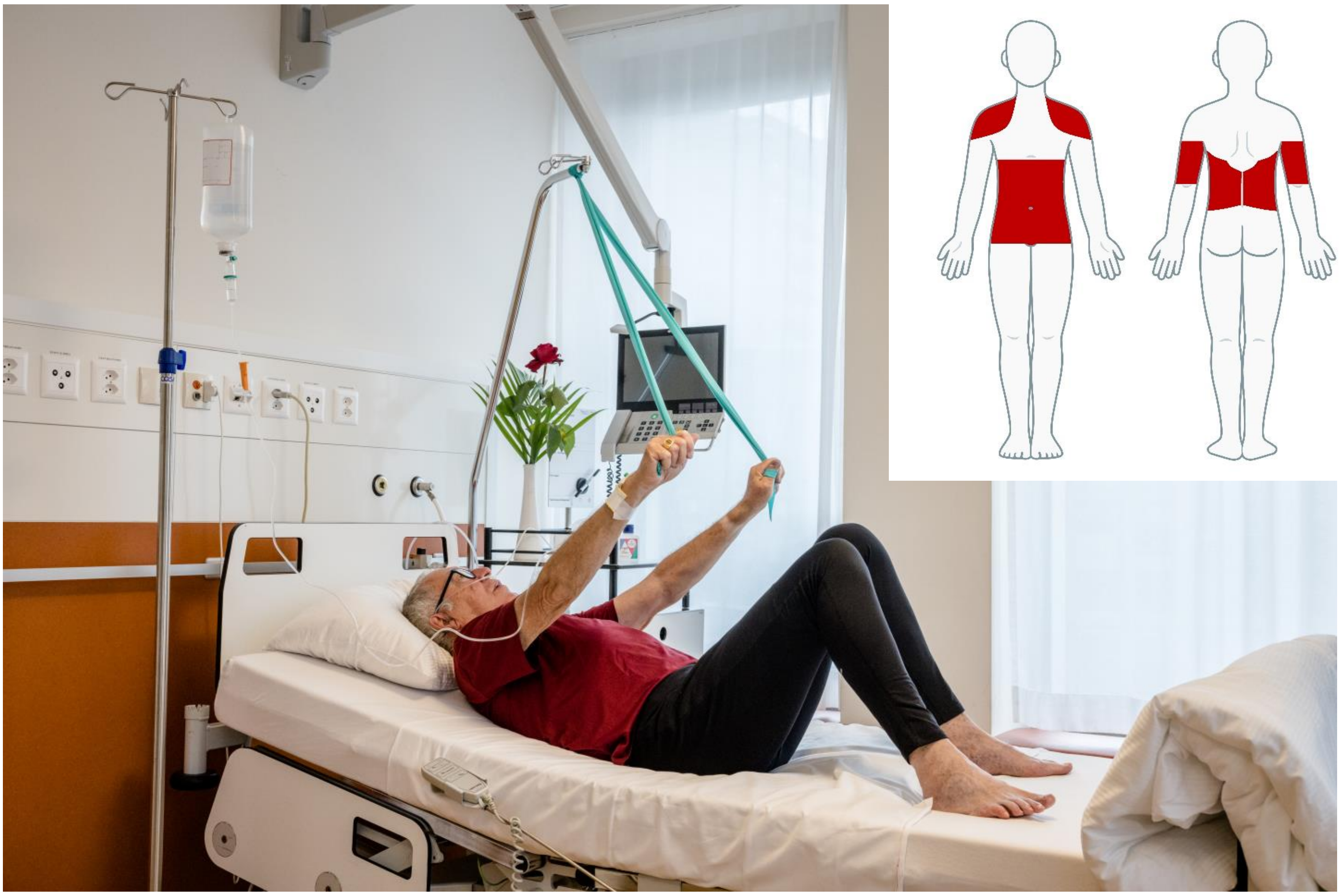

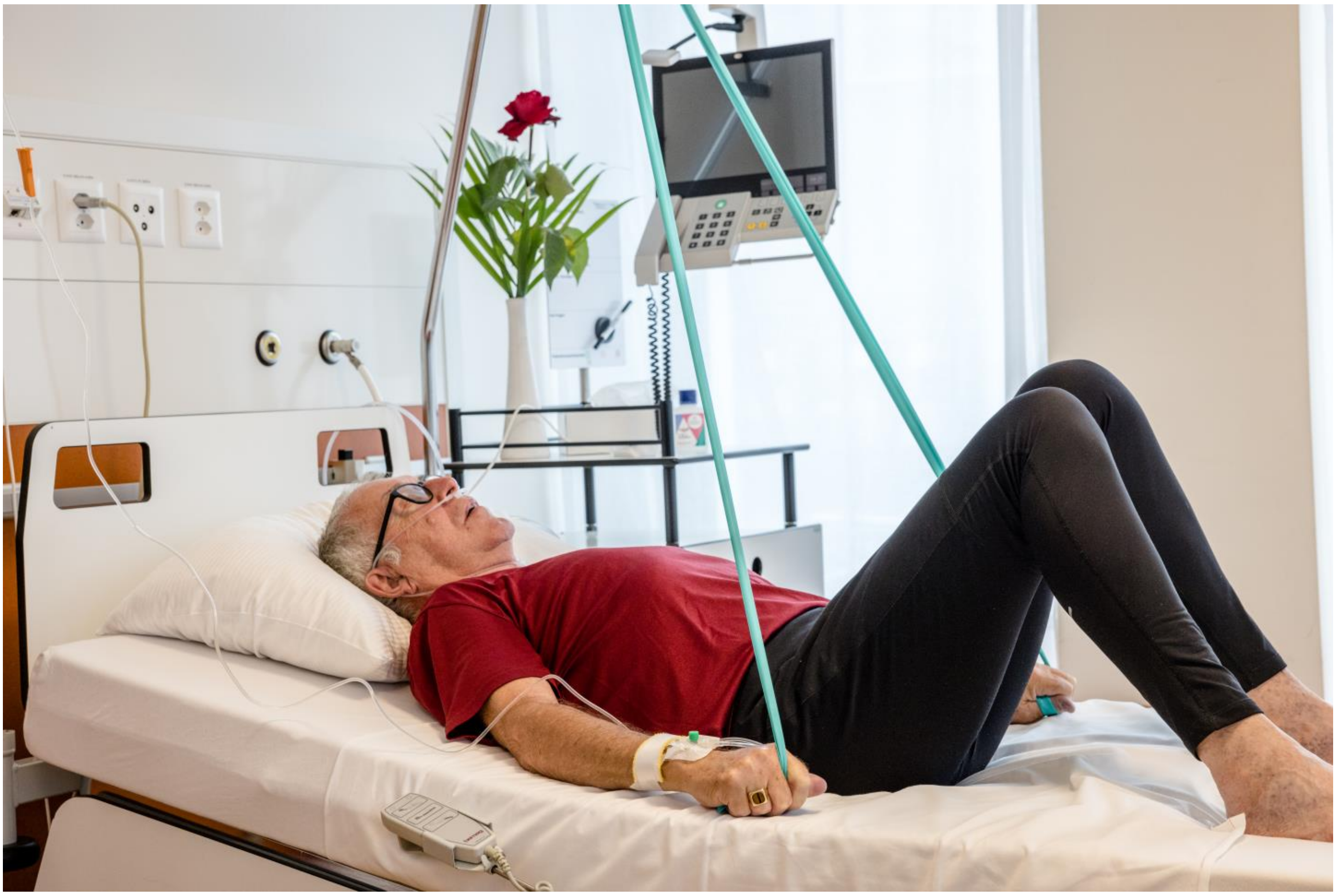

Stretching your arms with an elastic band (Video 7)

Stretch and then bend your arms several times against the resistance of an elastic band attached to the bar of the hospital bed.

This exercise strengthens the arm muscles.

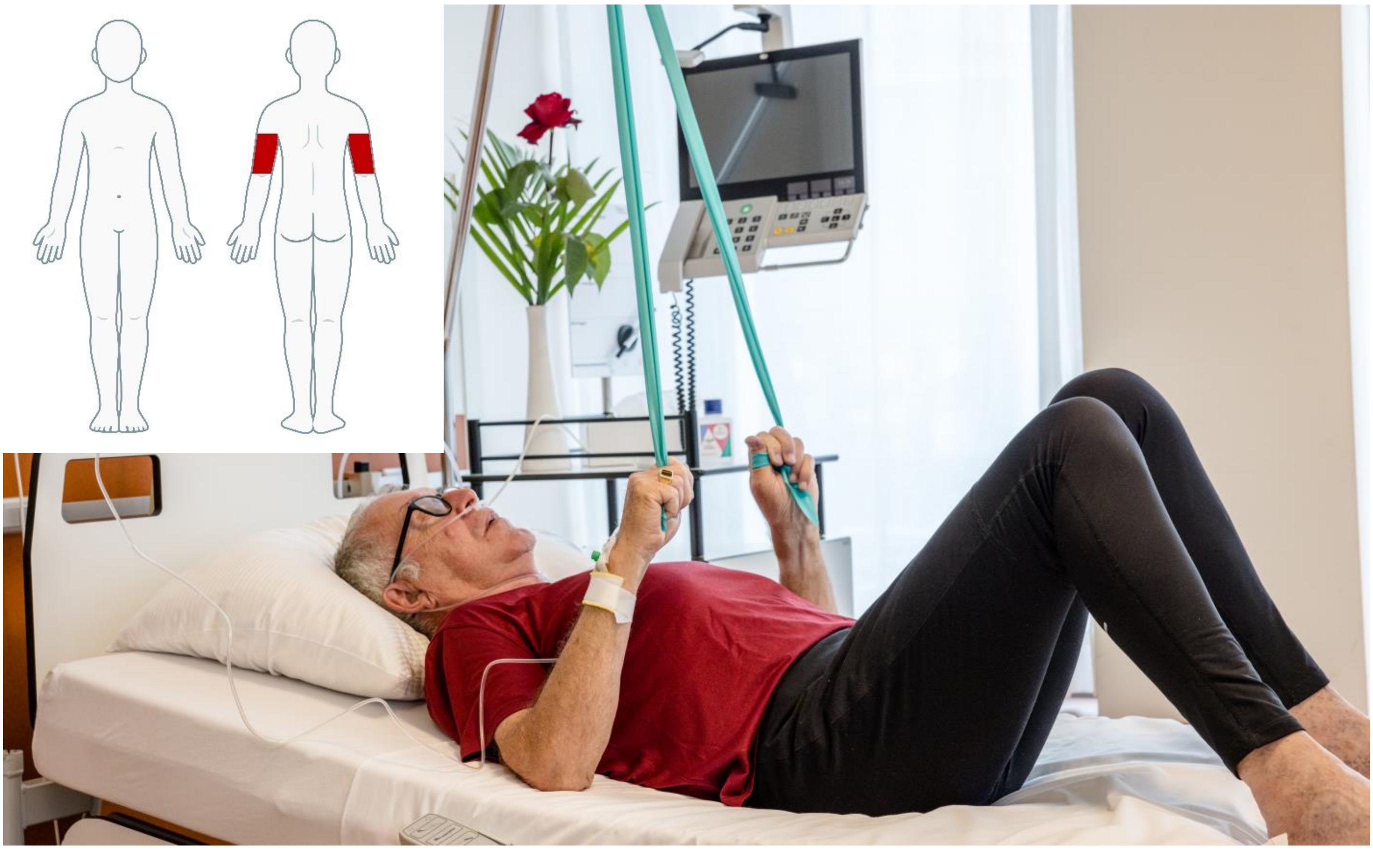

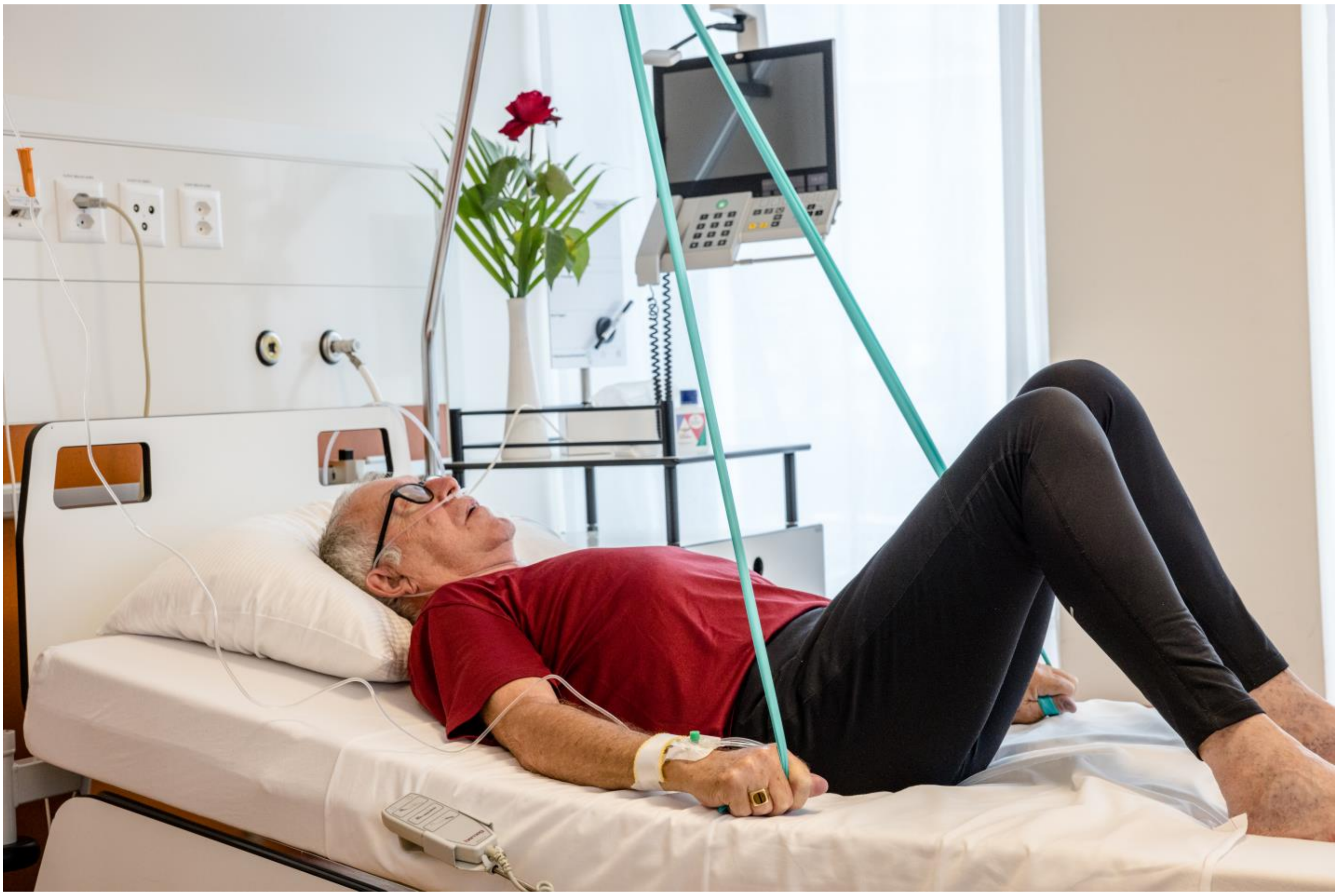

Stretching your legs with an elastic band (Video 8)

Hold the elastic fitness band with the arms bent, and stretch the leg several times against the resistance of the band. Then repeat the exercise with the other leg.
This exercise strengthens the thigh muscles.

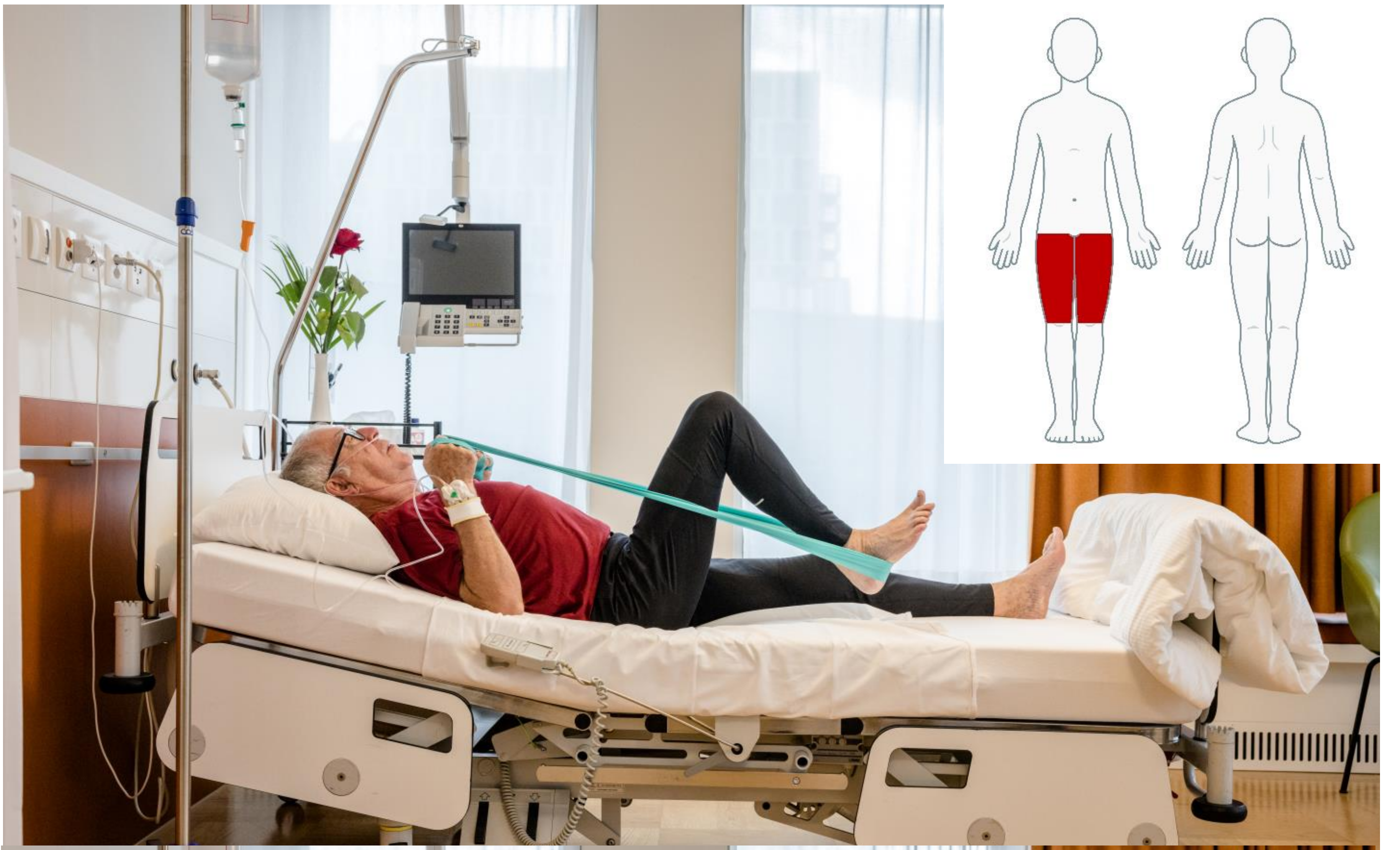

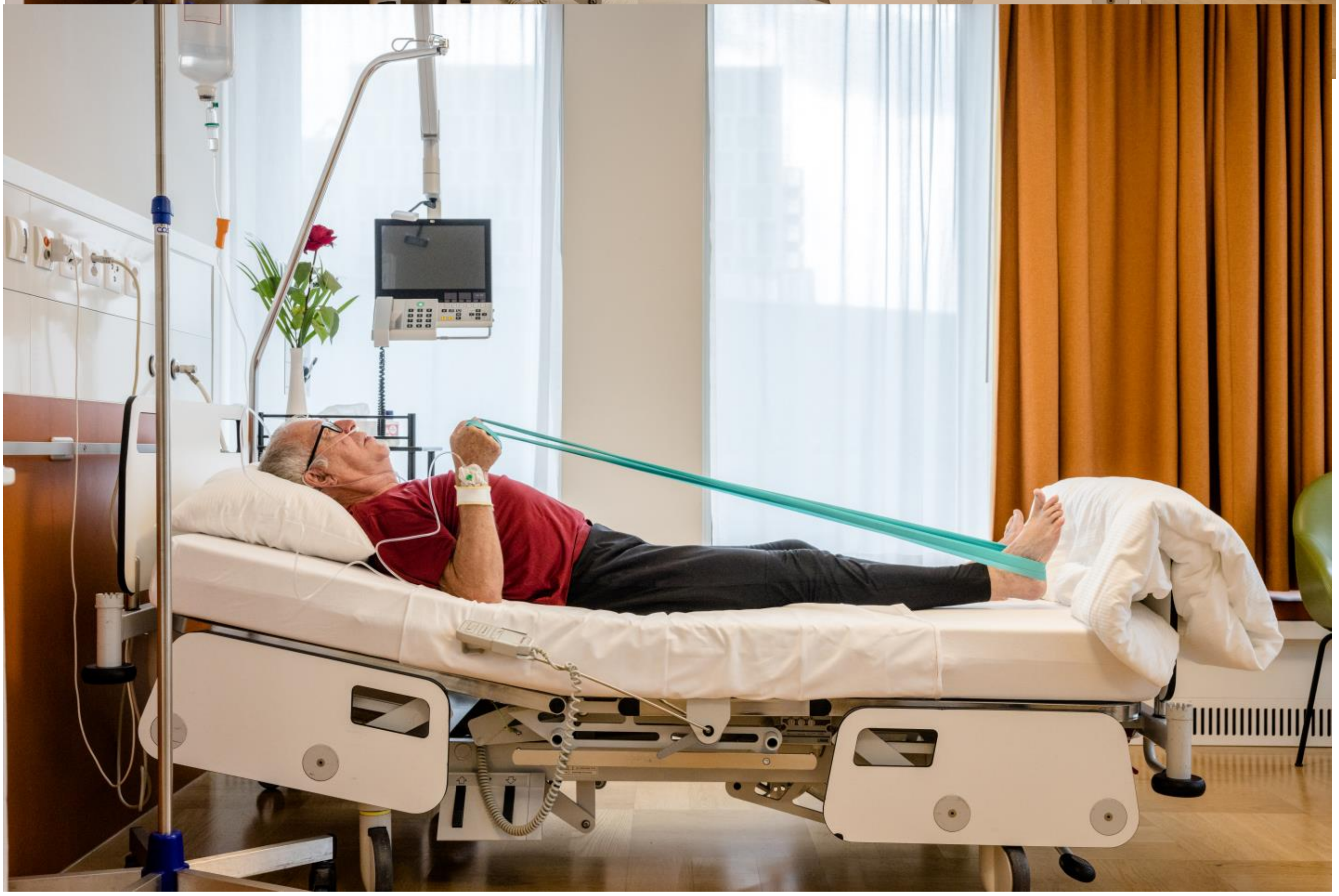

Bend your arms with an elastic band (Video 9)

Bend the arms several times against the resistance of the elastic placed under the soles of the feet, with the legs extended.
This exercise strengthens the arm muscles.

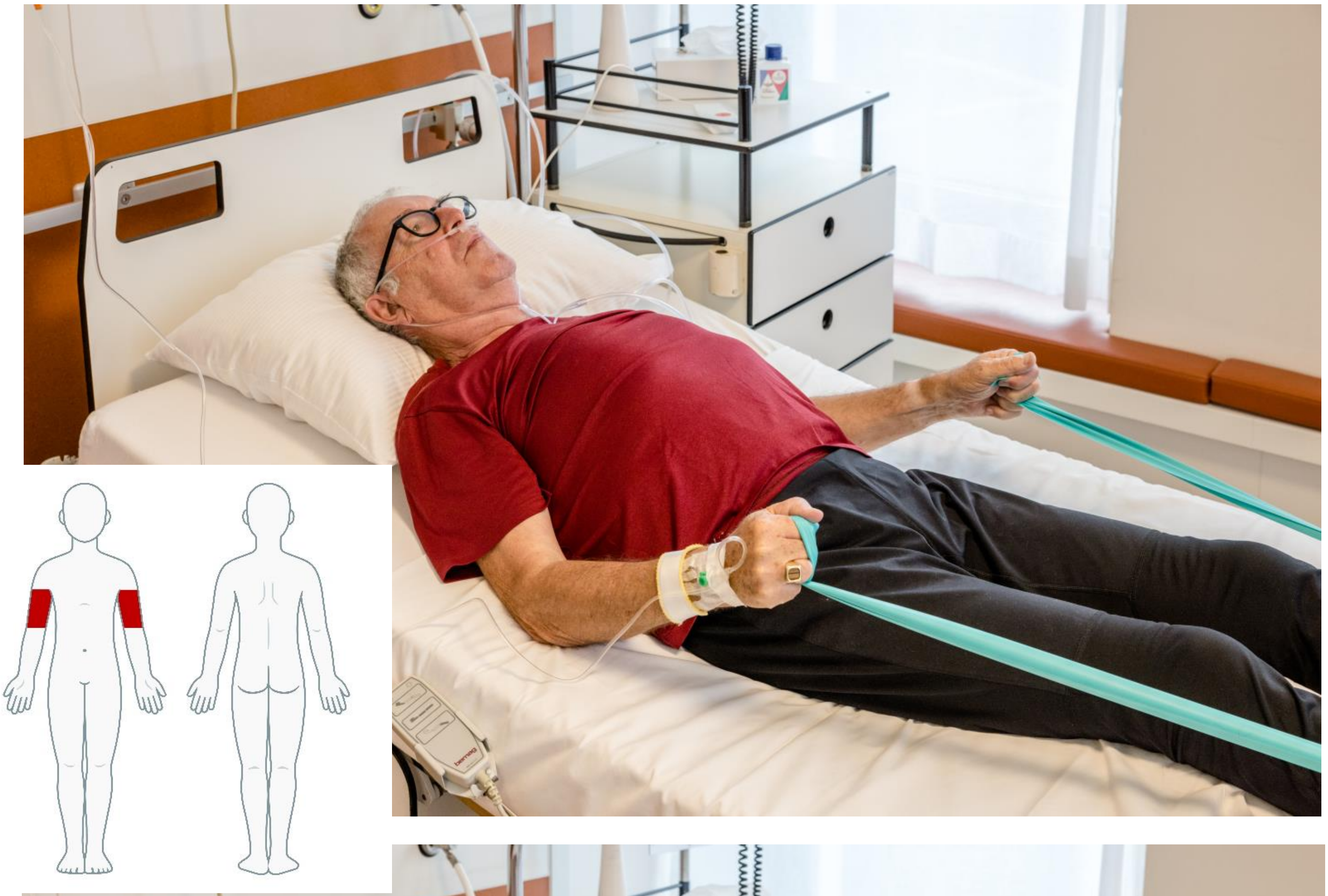

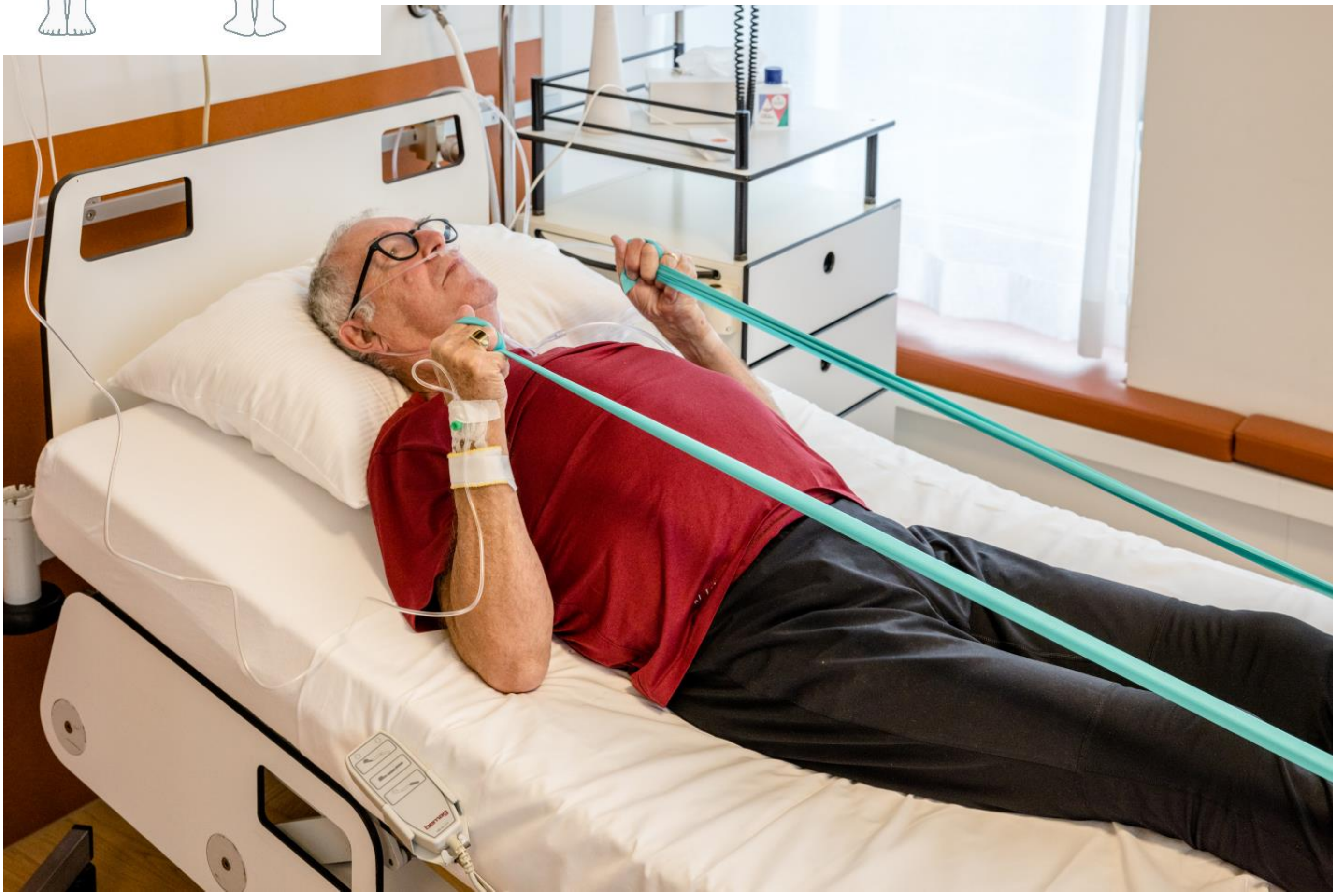

EXERCICES SITTING UP

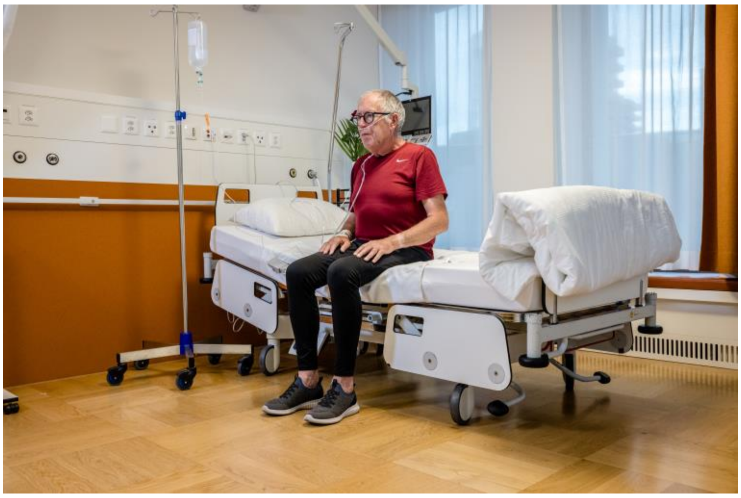

Moving to maintain autonomy

«Seated Walk» (Video 10)

Alternately lift one foot and then the other for about a minute, as if you were walking while sitting.
This exercise activates blood circulation.

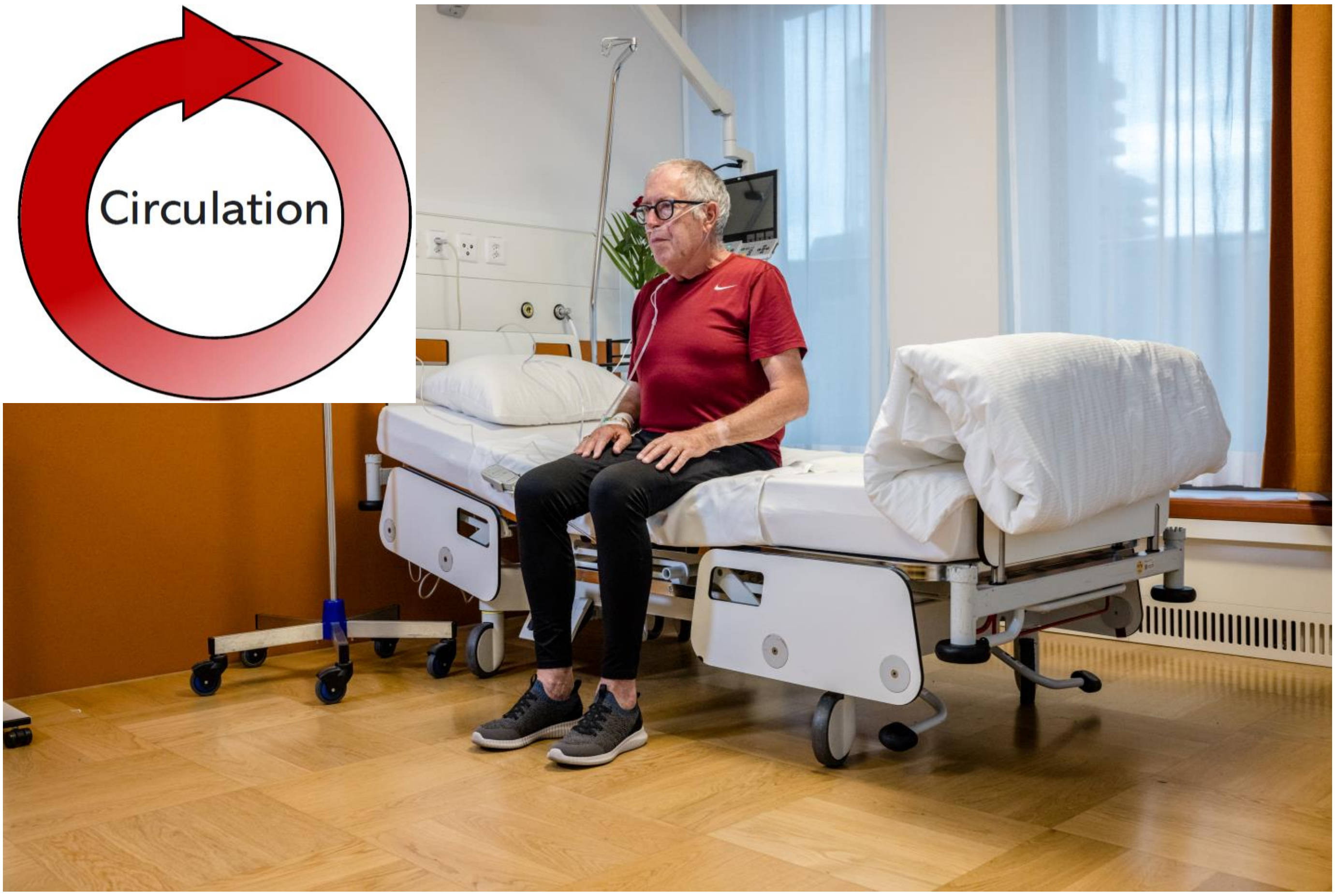

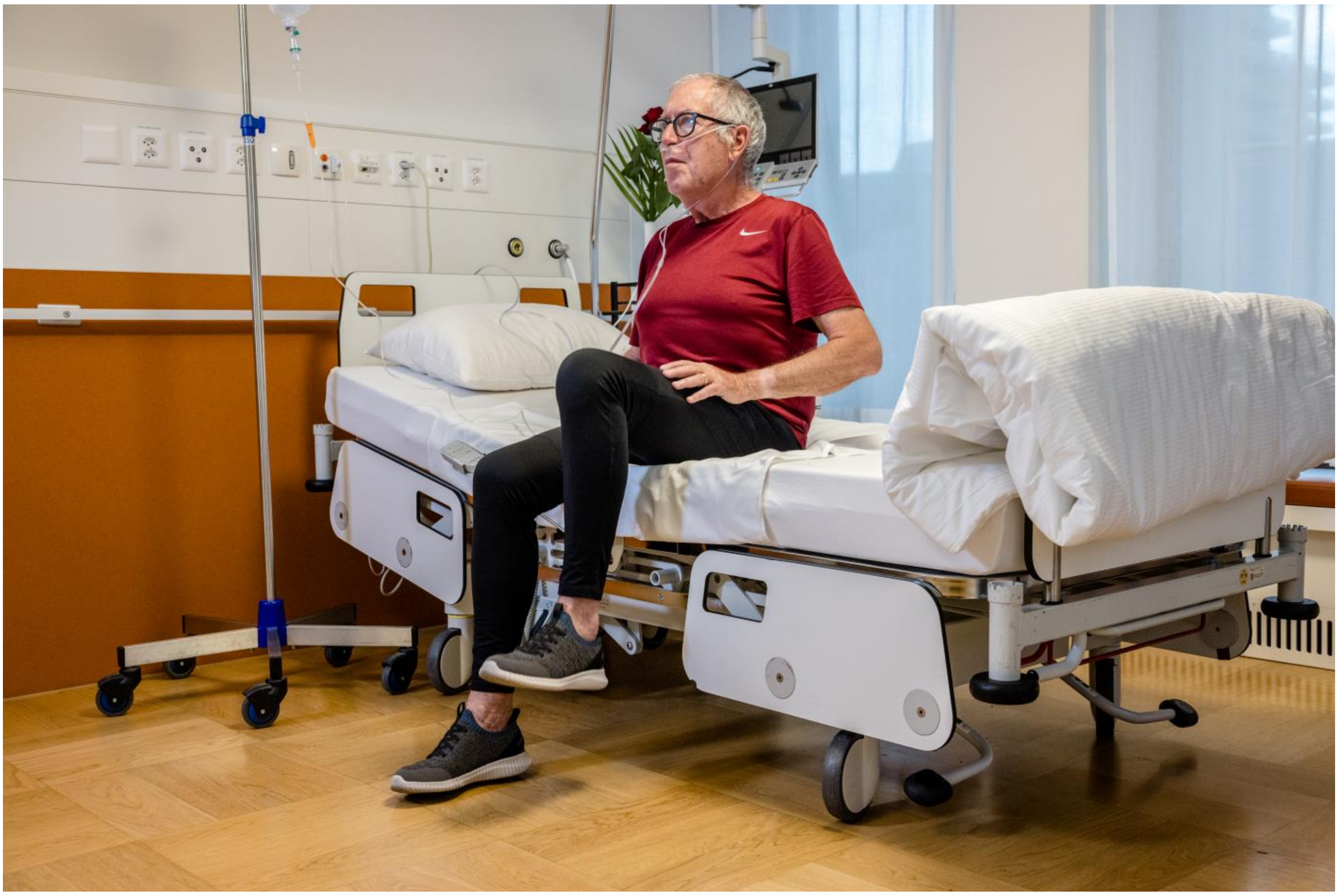

Stretching your legs (Video 11)

First stretch and bend one leg several times, then the other.
This exercise strengthens the thigh muscles.

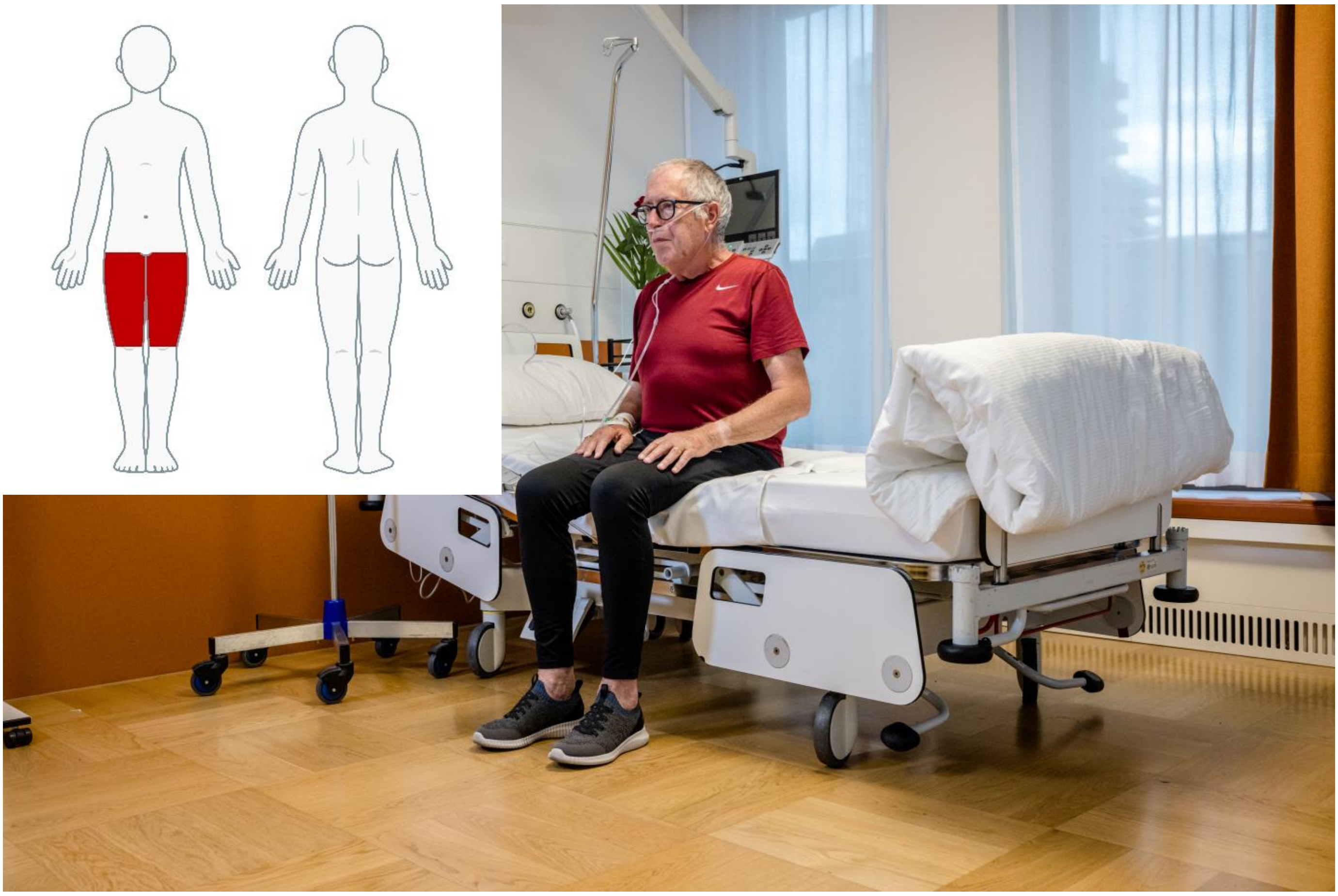

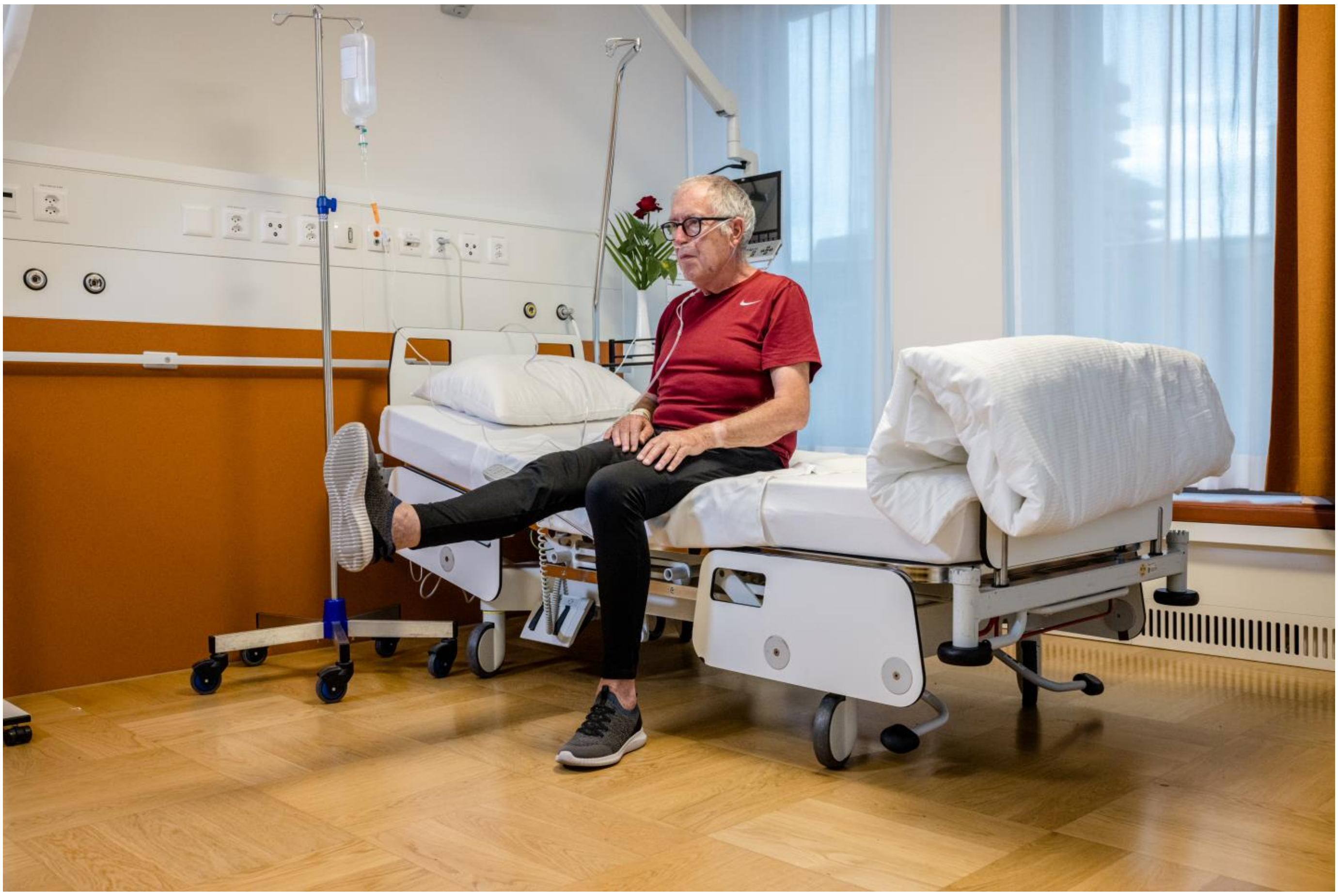

Stretching your legs with an elastic band (Video 12)

Hold the elastic with the arms bent and stretch one leg several times against the resistance of the elastic fitness band. Then repeat the exercise with the other leg.
This exercise strengthens the thigh muscles.

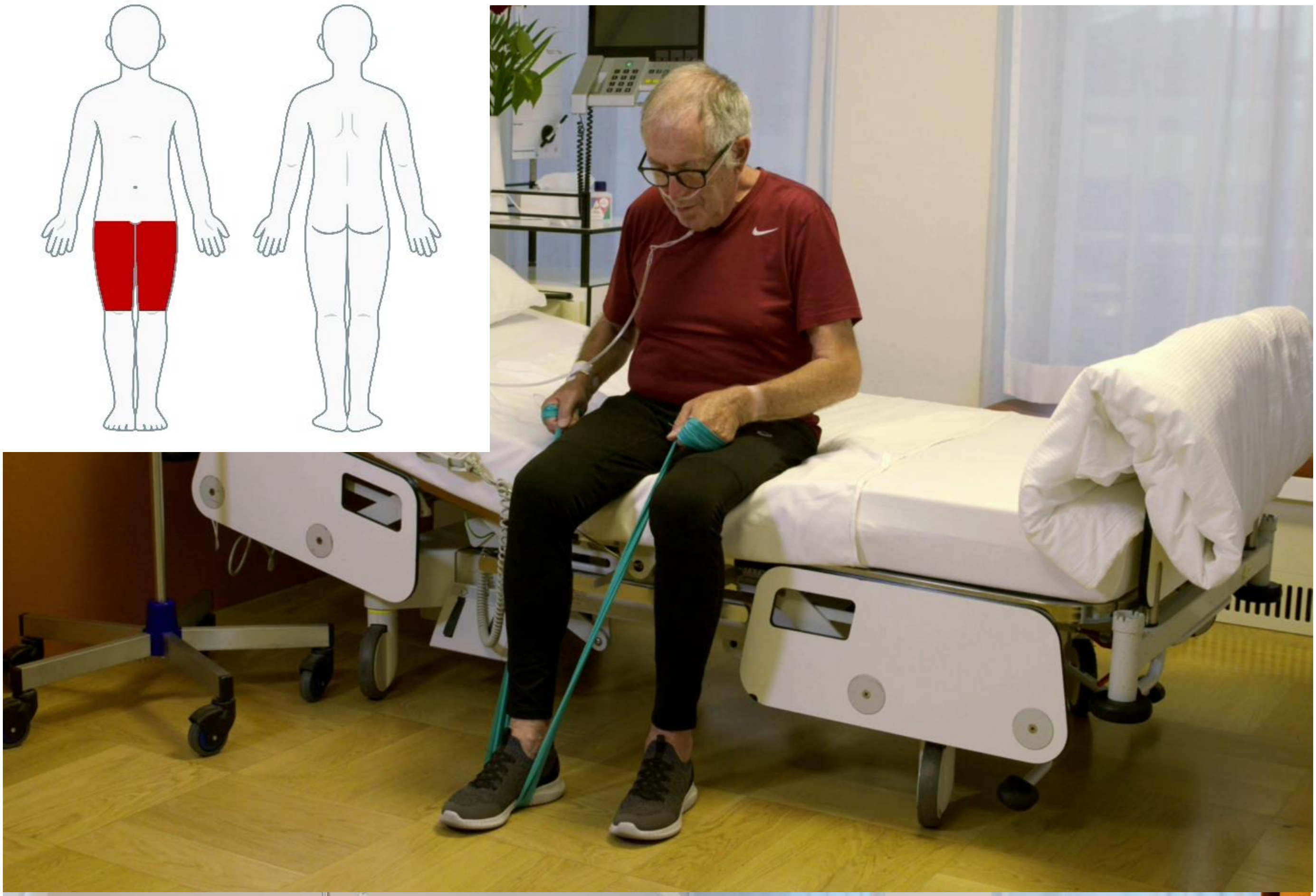

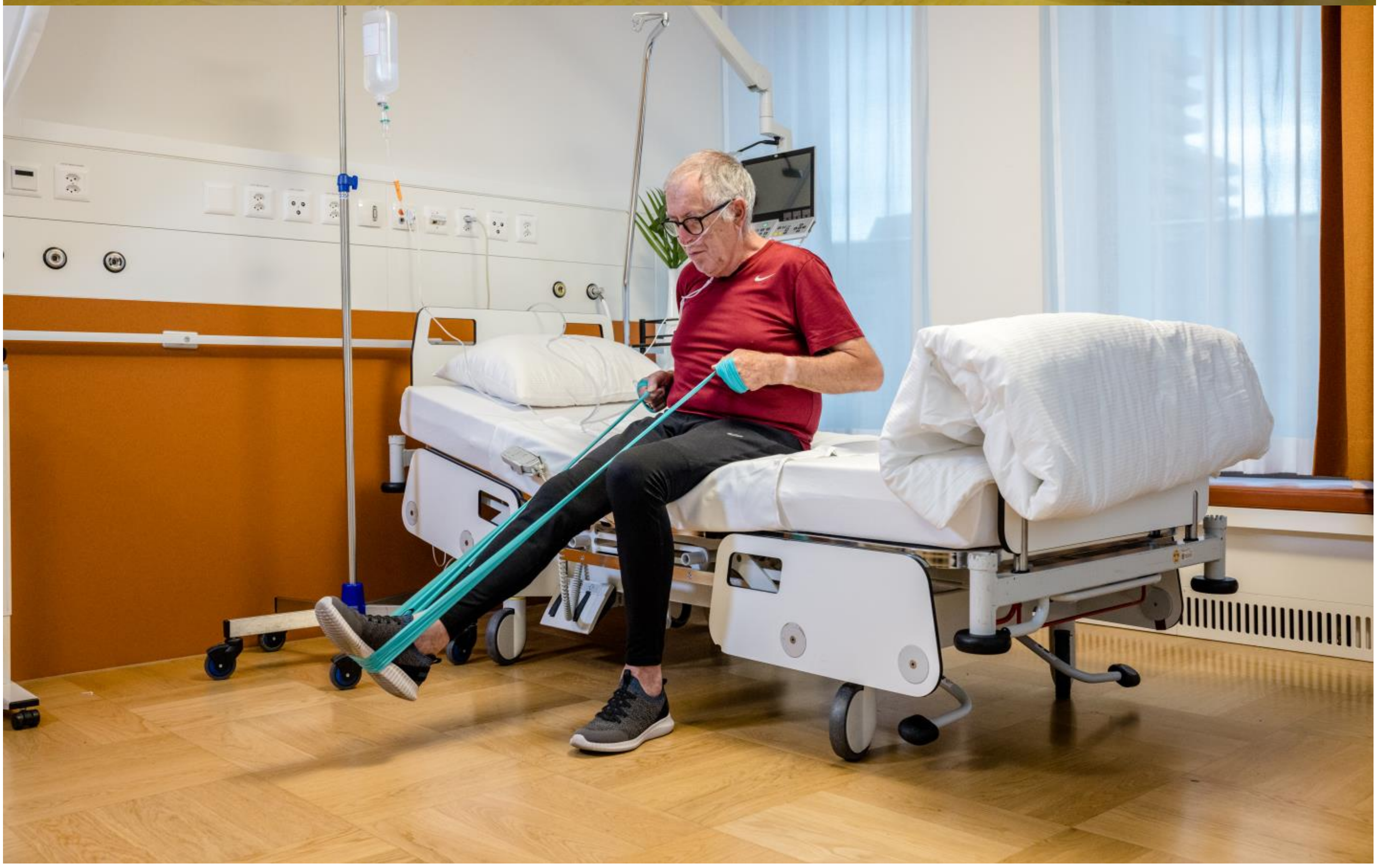

Bend your arms with an elastic band (Video 13)

Bend your arms several times against the resistance of the elastic fitness band placed under the soles of the feet.
This exercise strengthens the muscles of the arms.

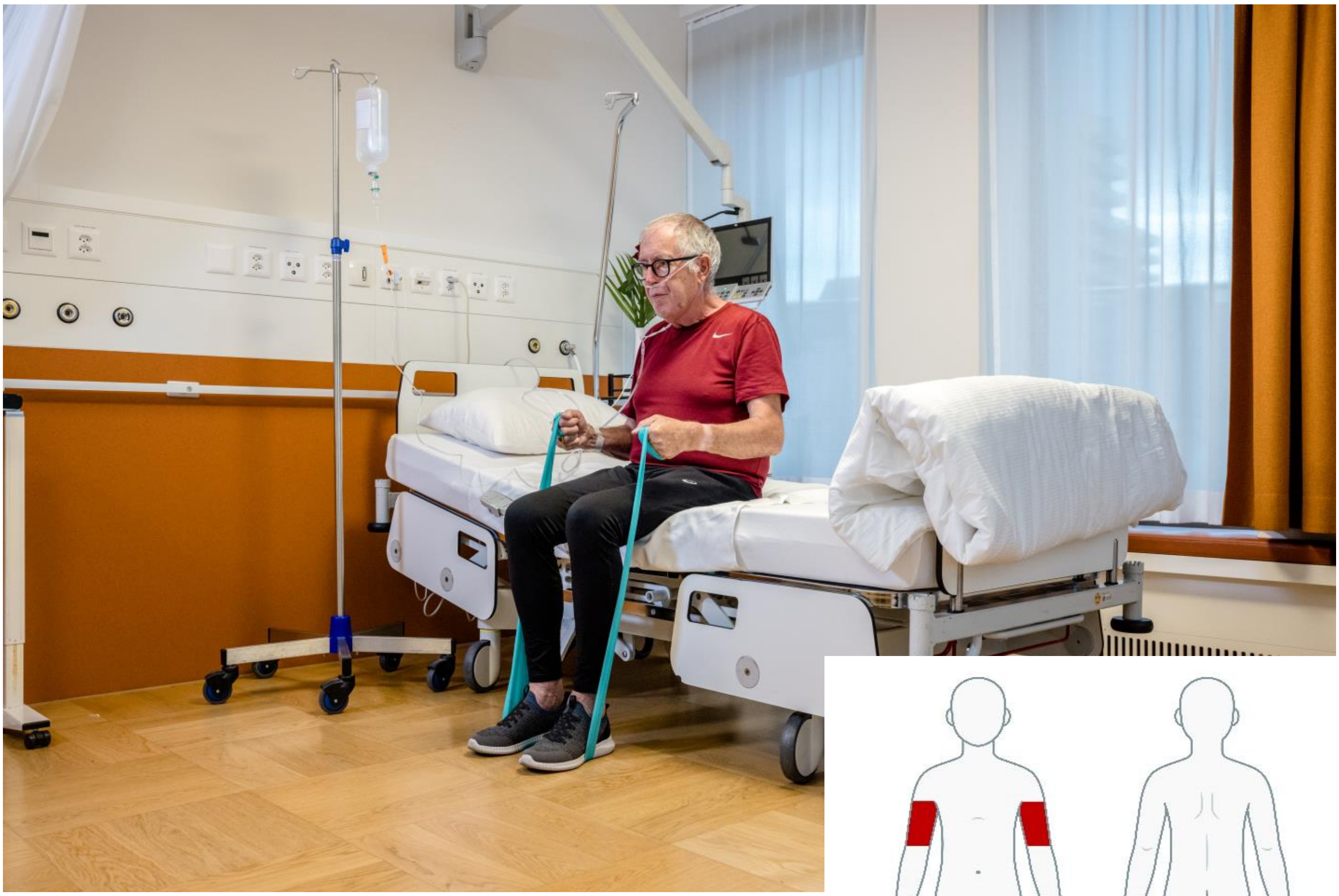

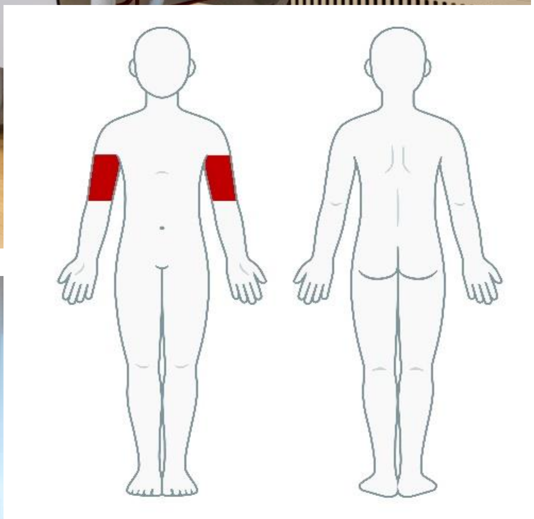

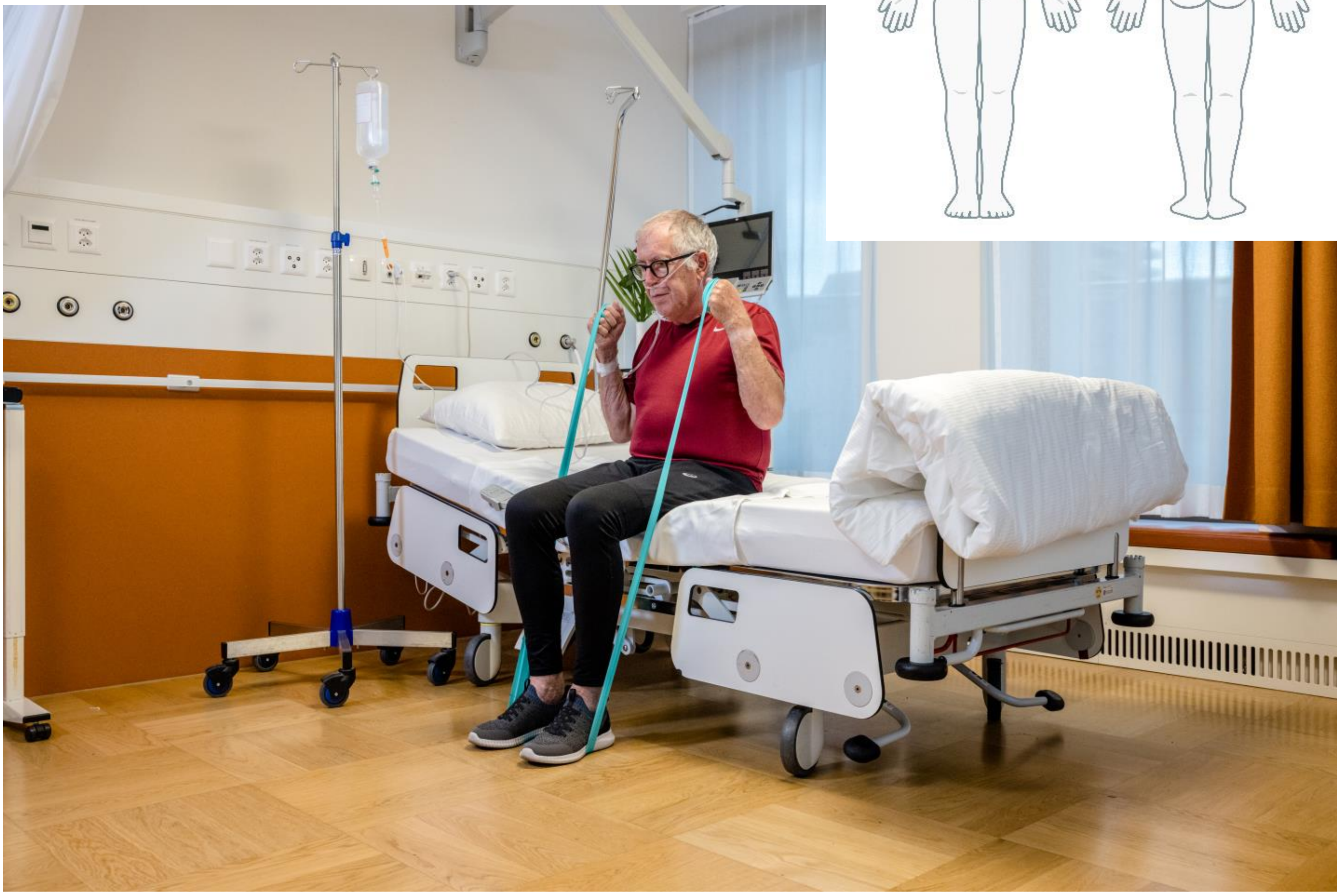

Reaching out with the arms (Video 14)

Stretch and bend the 2 arms for about a minute against the force of gravity.
This exercise strengthens the shoulder muscles.

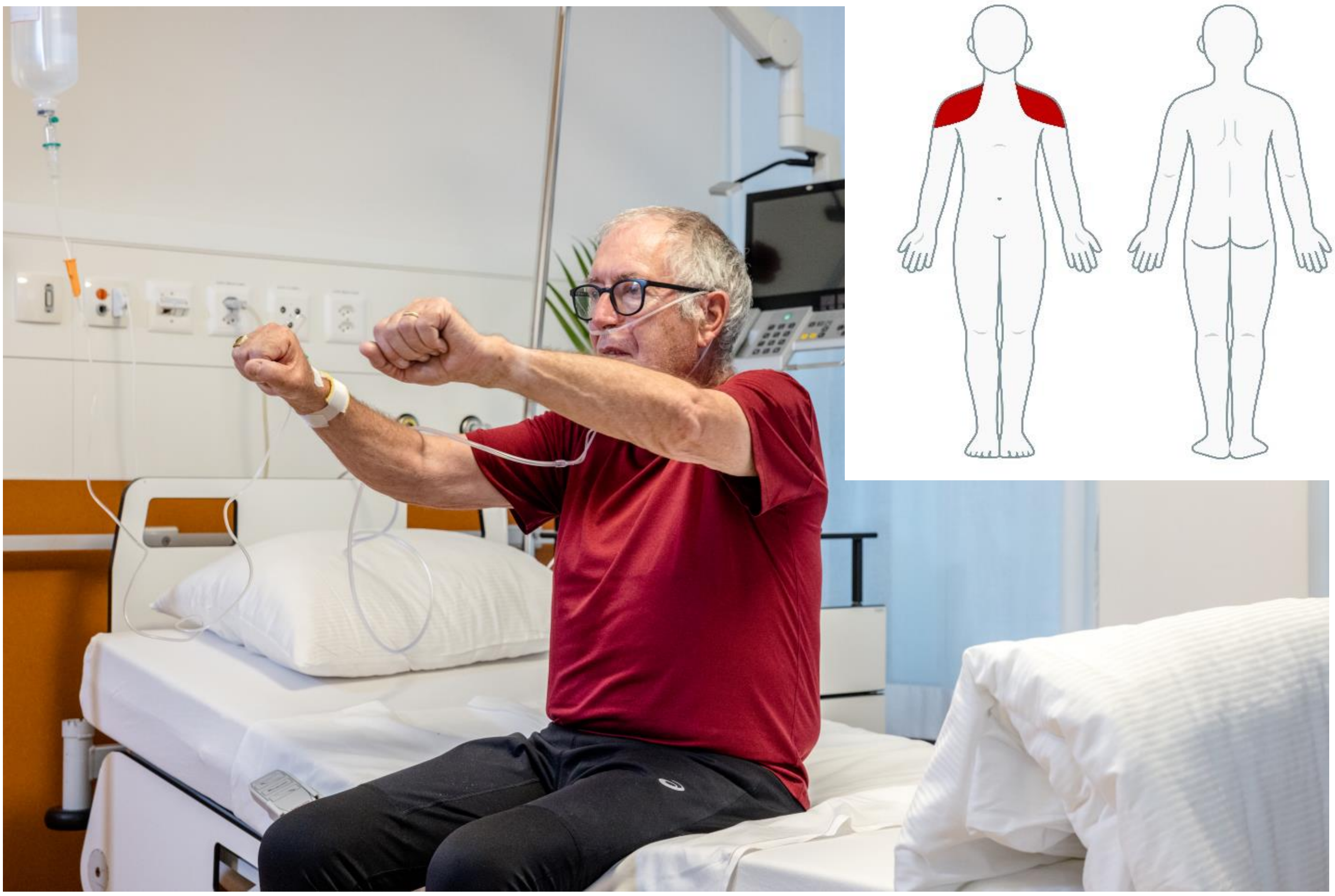

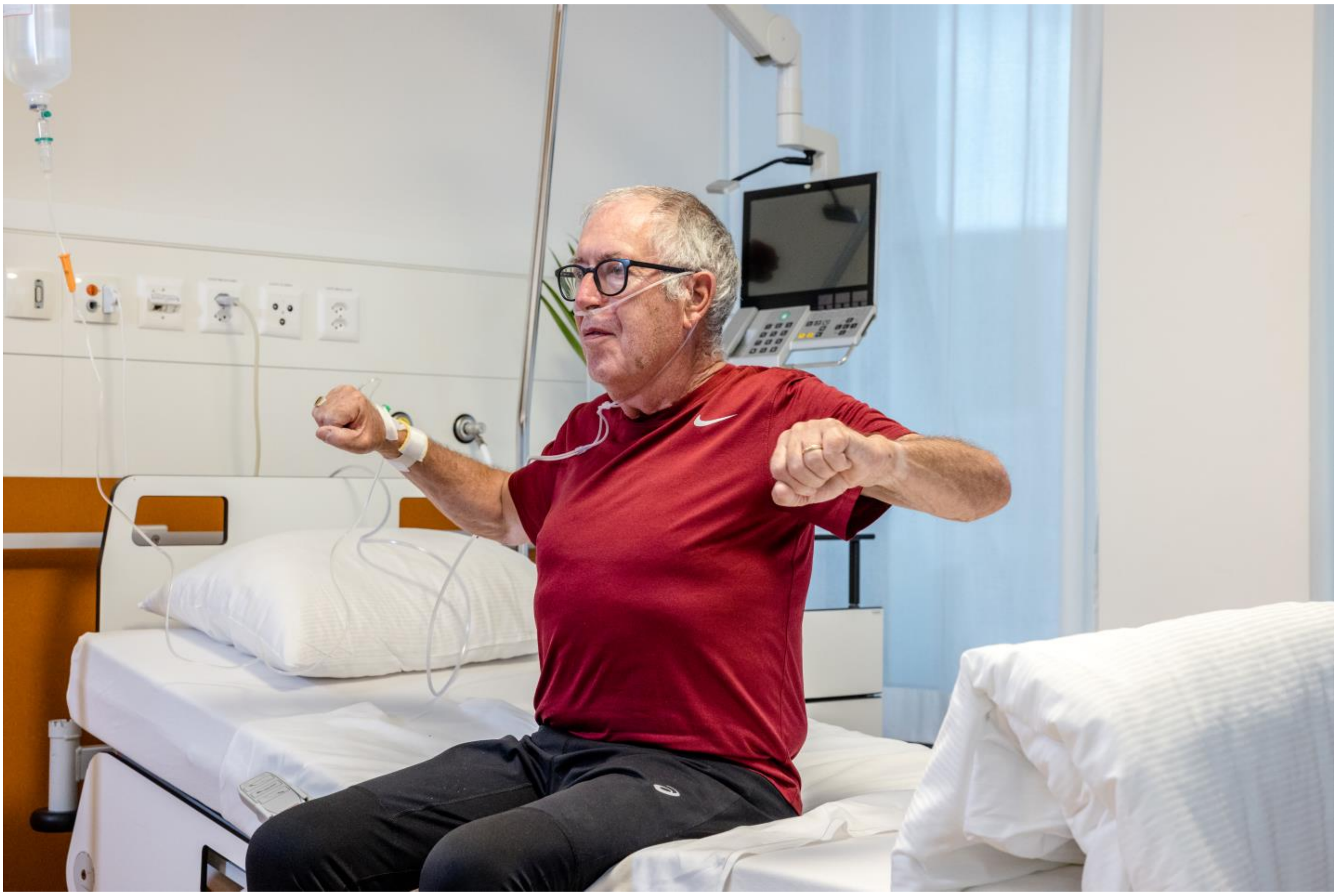

Stretching your arms with an elastic band (Video 15)

Stretch and bend your arms several times against the resistance of the elastic fitness band.
This exercise strengthens the arm muscles.

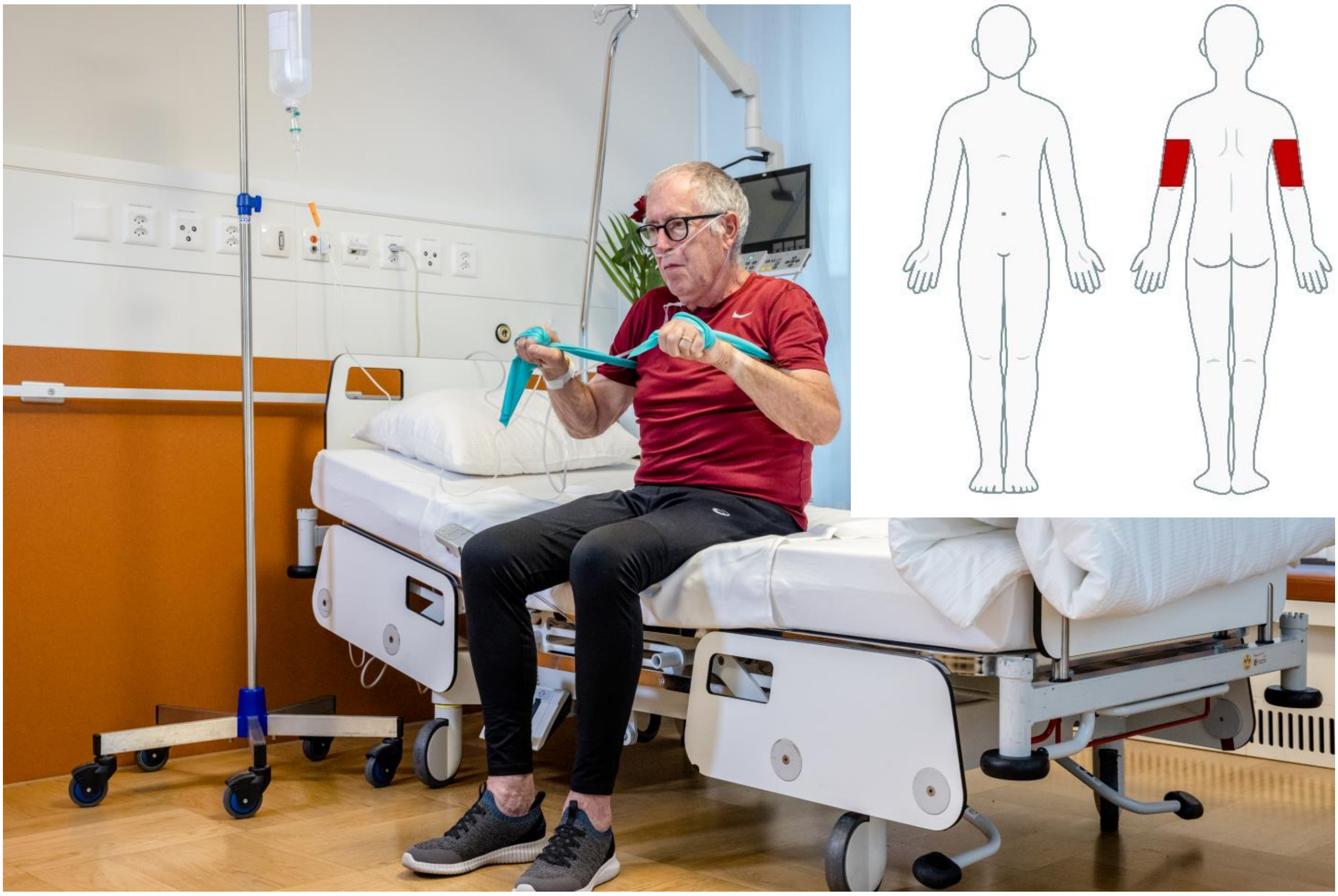

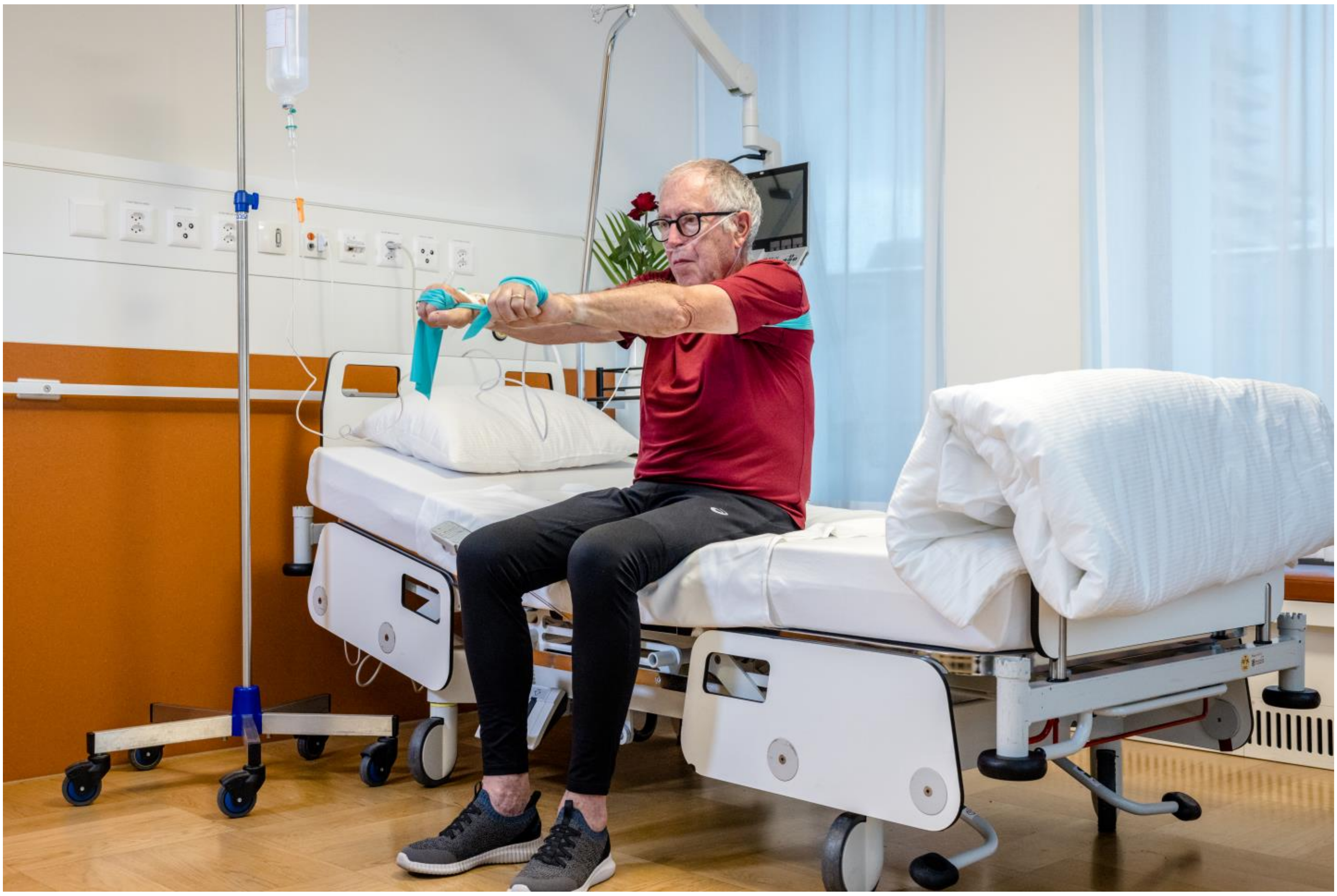

Trunk flexion (Video 16)

Lean back as much as possible for yourself, then straighten up. Repeat the exercise a few times.
This exercise strengthens the stomach muscles.

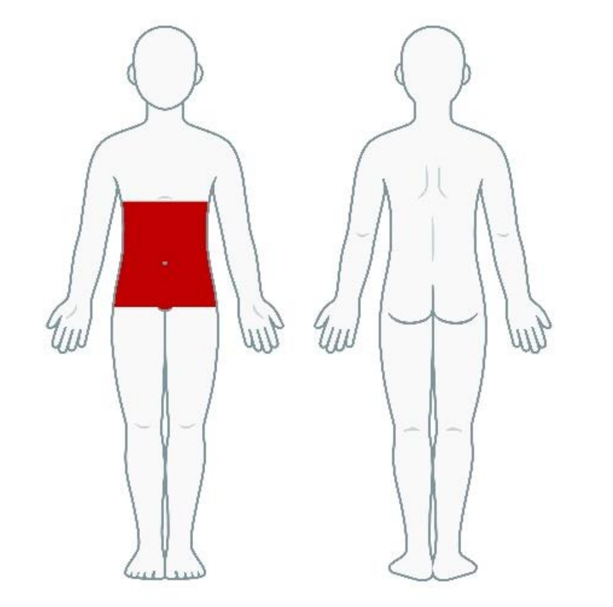

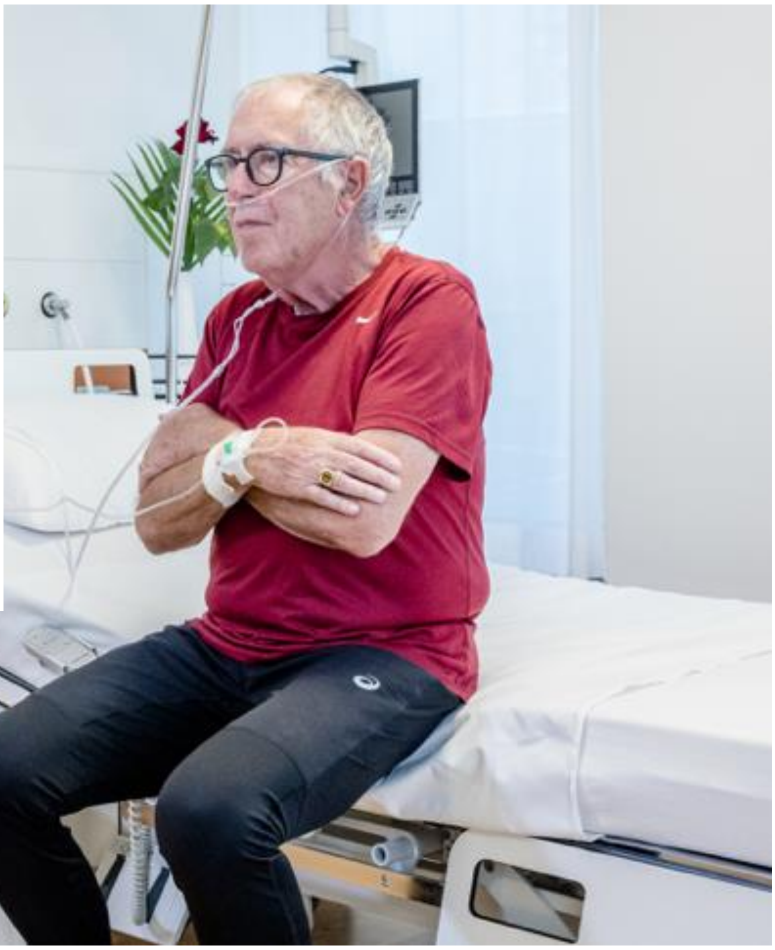

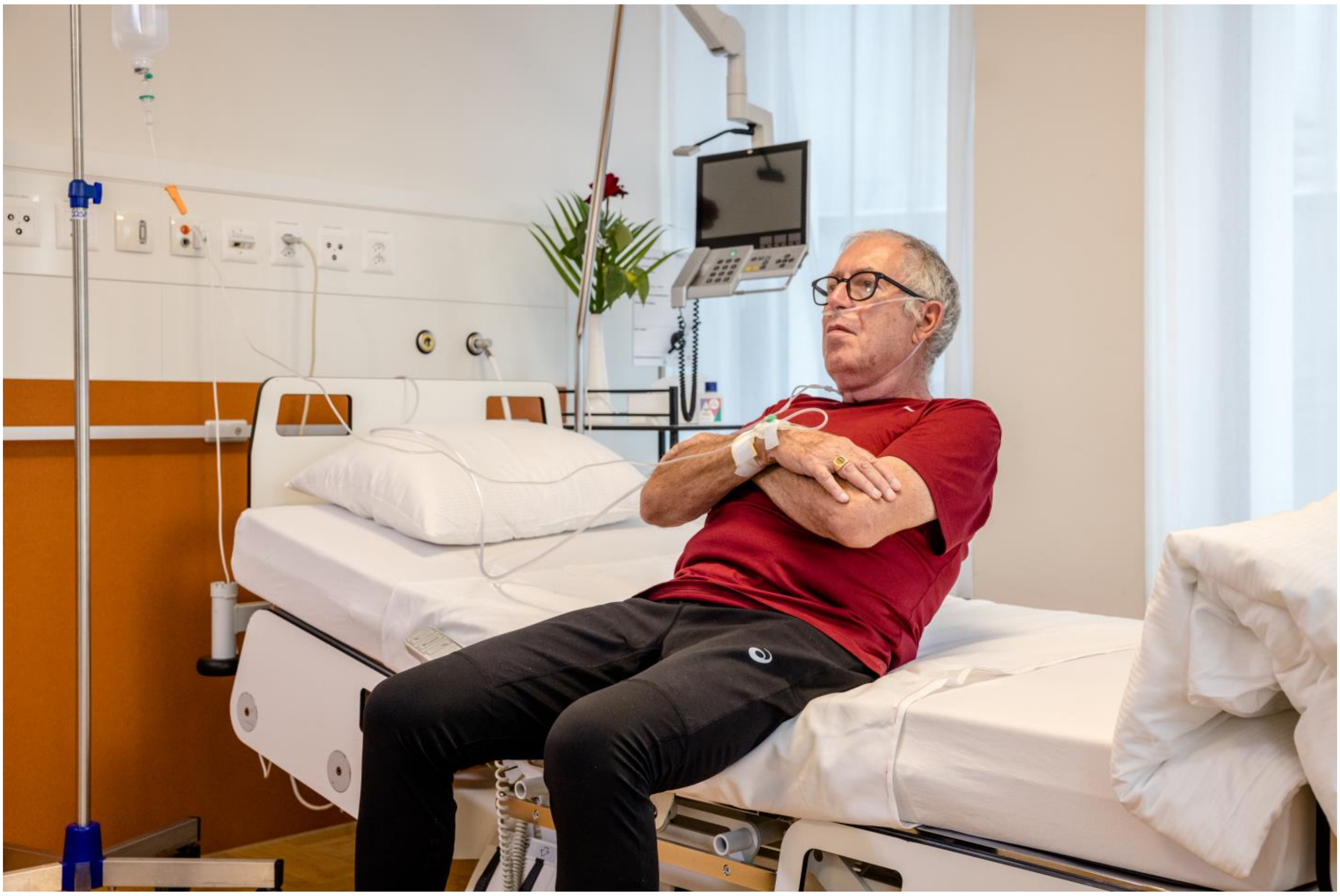

INTOMOB

EXERCICES STANDING UP

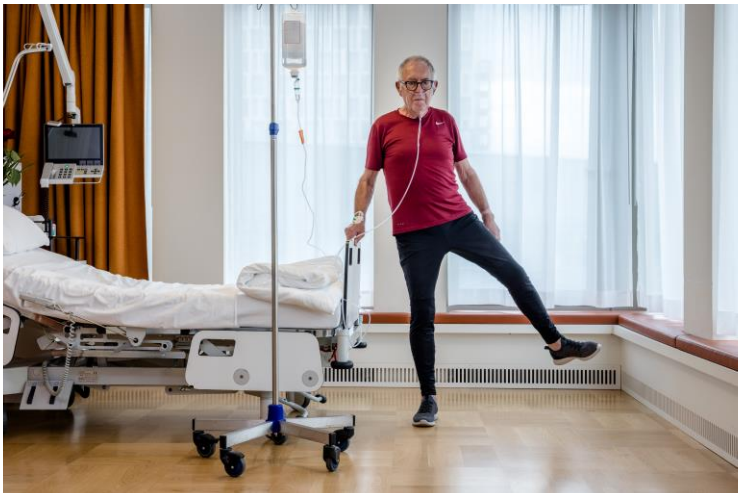

Moving to maintain autonomy

Getting Up and Sitting Down (Video 17)

Get up several times from the edge of the bed or from a chair. Help yourself with your arms only if it is not possible otherwise. The lower the bed or chair, the harder the exercise.

This exercise strengthens the thigh muscles.

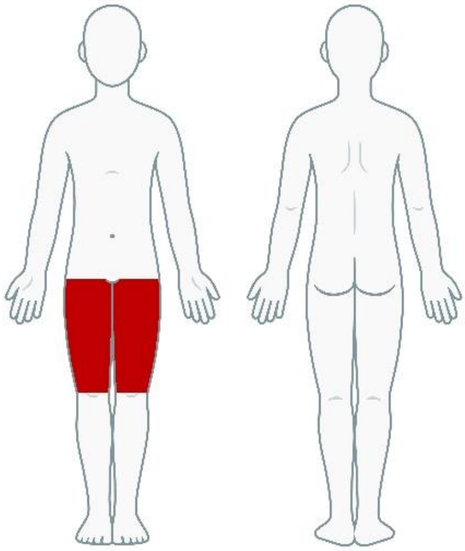

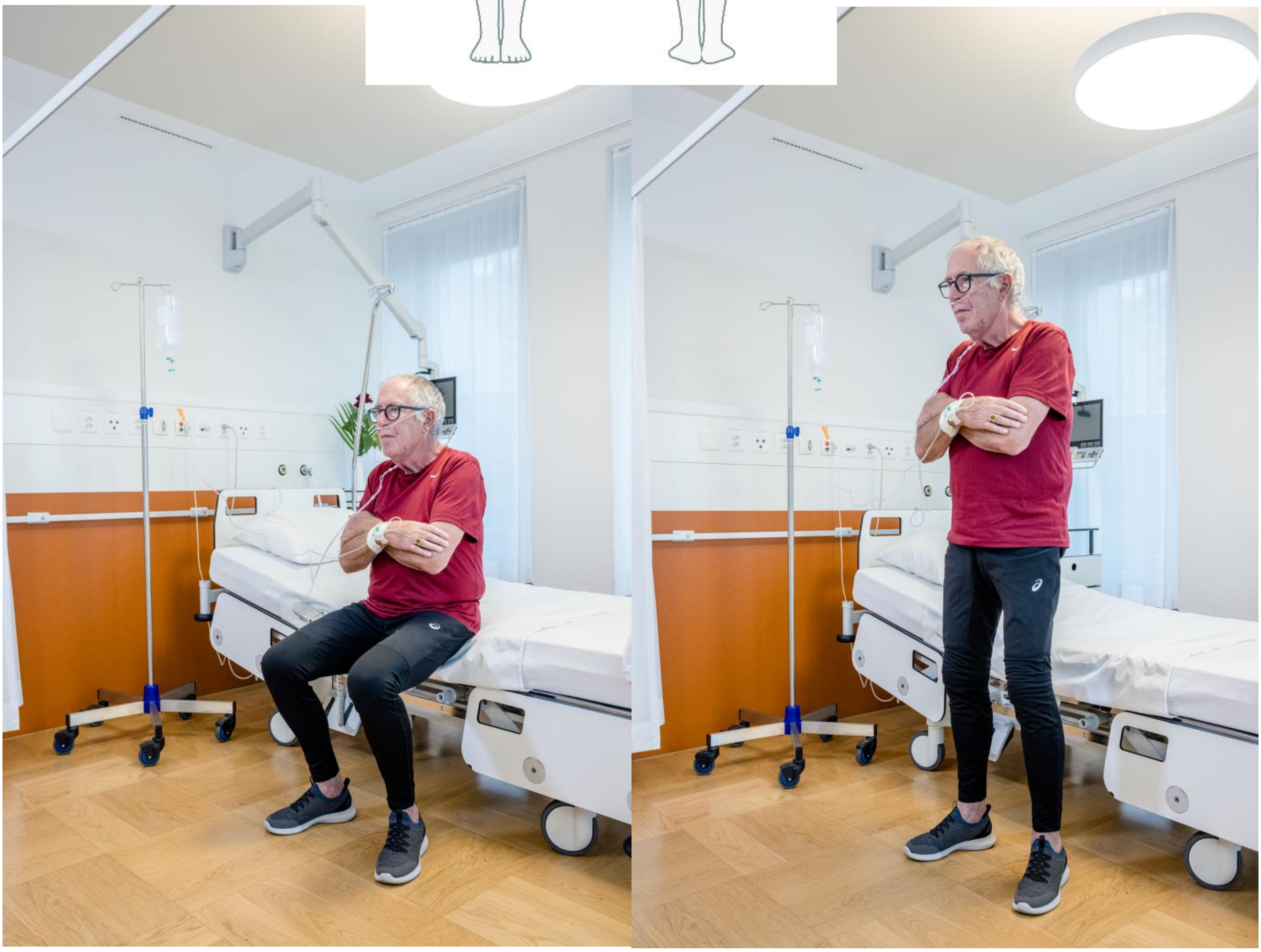

Static balance
Difficulty Level 1 (Video 18)

Stand near a place that you can hold on to with your hands (for example, at the end of the bed). Place your feet together and then stop holding on with your hands. If you can keep yourself balanced for about 10 seconds without holding on with your hands, you can make the exercise more difficult with the following variations:

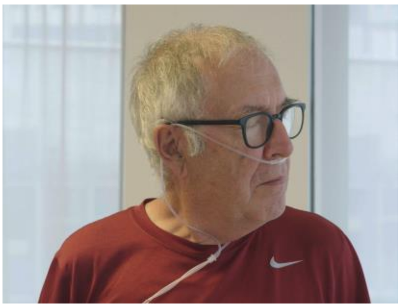

1) Turn head right to left slowly for approx. 10 seconds.
2) Raise and lower the head alternately slowly for approx. 10 seconds.
3) Close your eyes.
4) Do variation 1) or 2) with your eyes closed

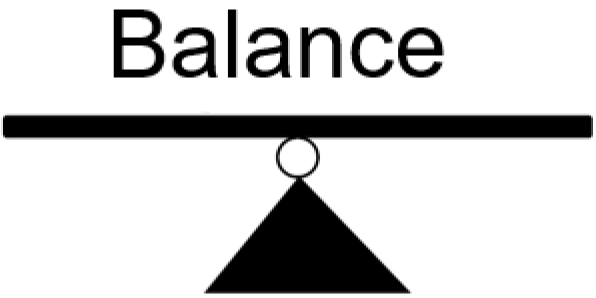

These exercises allow you to train your balance.

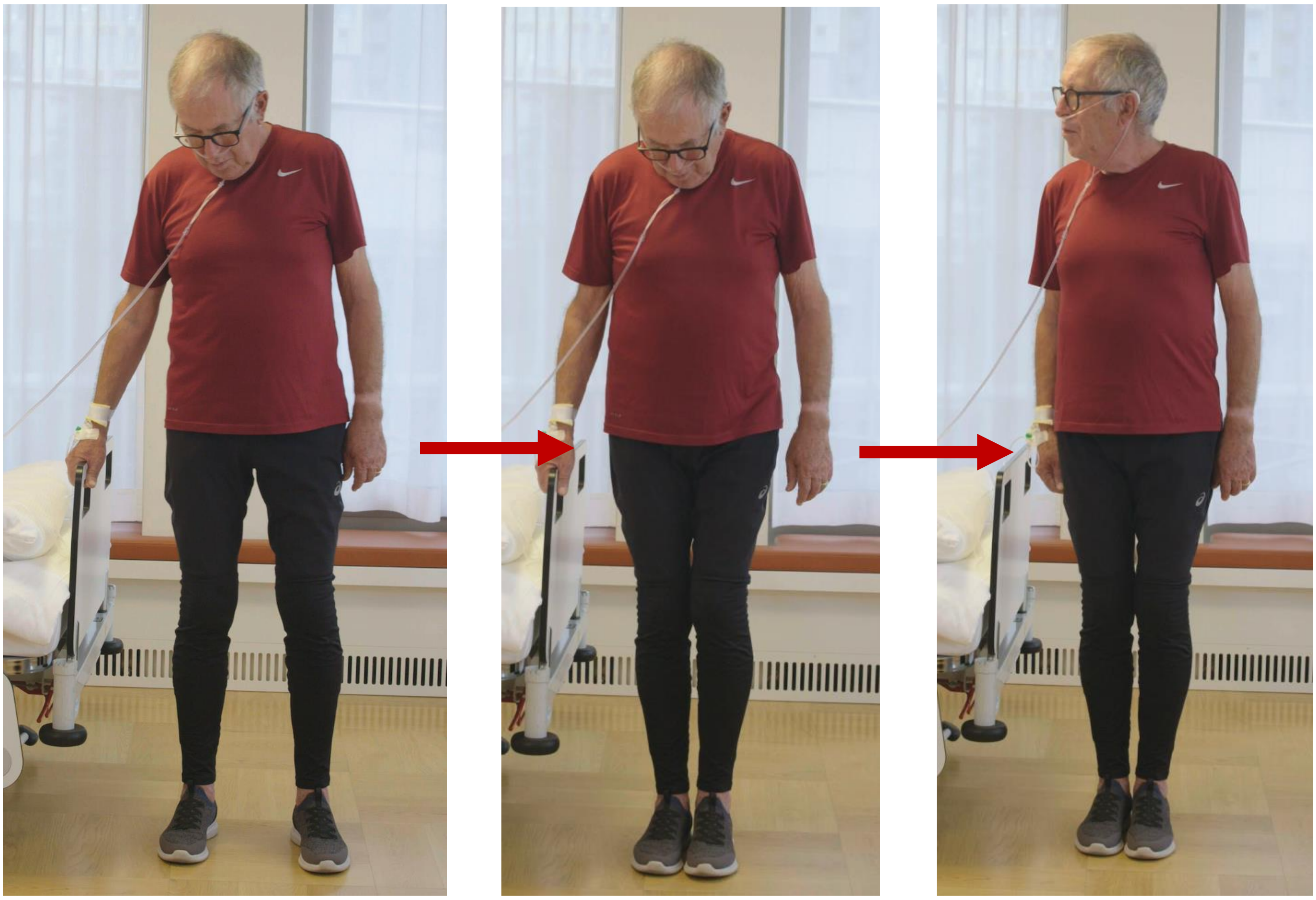

Static balance
Difficulty Level 2 (Video 19)

Stand near a place that you can hold on to with your hands (for example, at the end of the bed). Place one foot in front of the other and then stop holding on with your hands. If you can keep yourself balanced for about 10 seconds without holding on with your hands, you can make the exercise more difficult with the following variations:

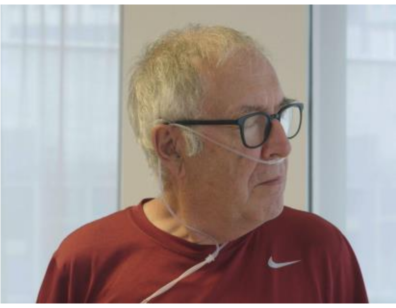

1) Turn head right to left slowly for approx. 10 seconds.
2) Raise and lower the head alternately slowly for approx. 10 seconds.
3) Close your eyes.
4) Do variation 1) or 2) with your eyes closed.

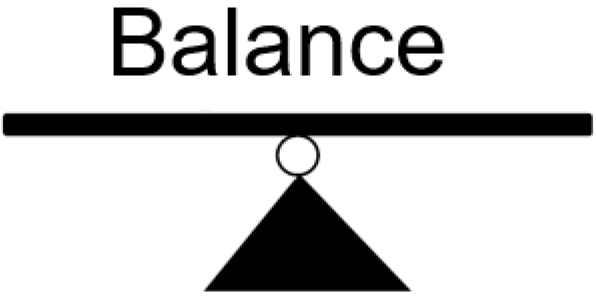

These exercises allow you to train your balance.

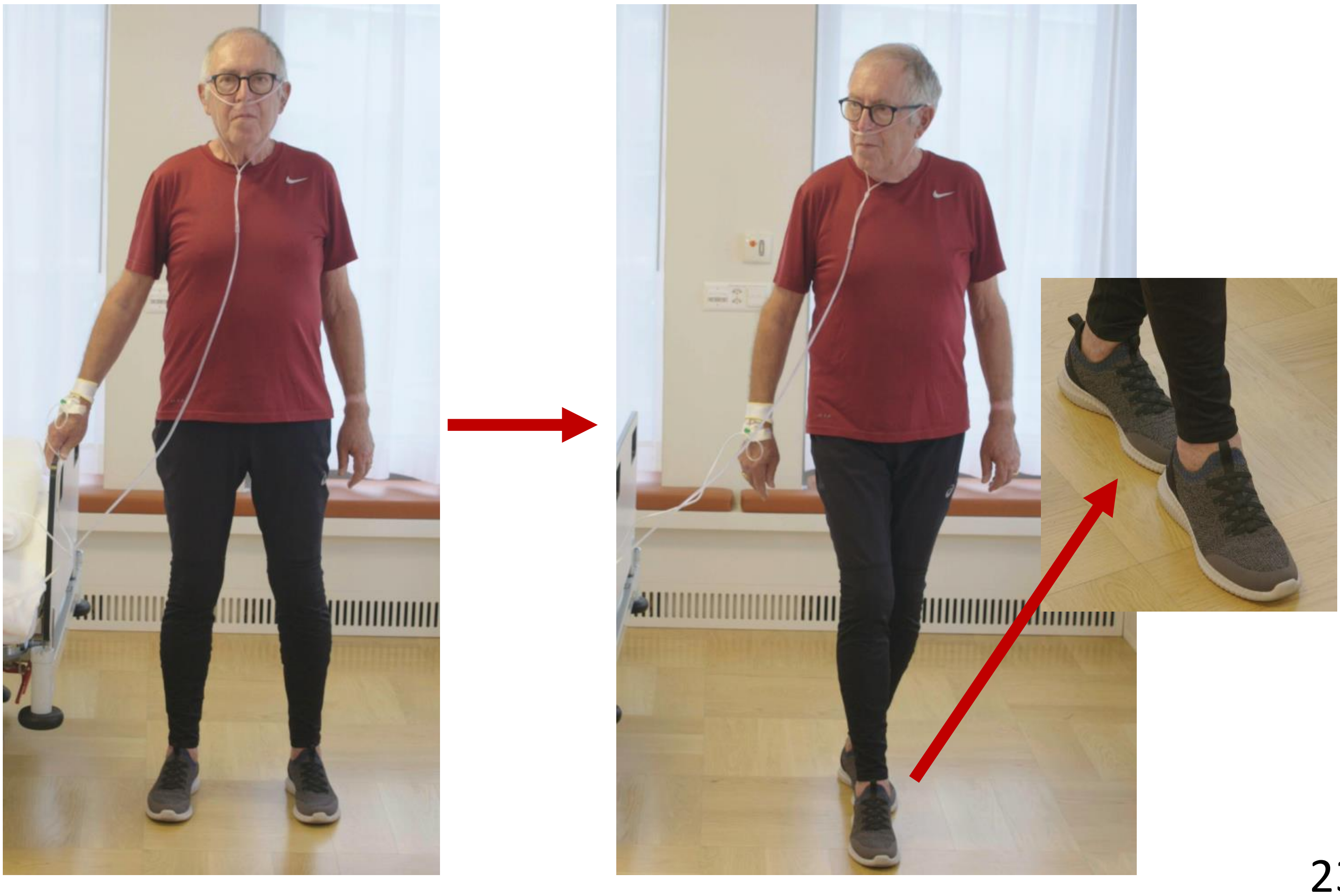

Tiptoe (Video 20)

Stand in a place where you can, if necessary, hold on with your hands (for example, at the end of the bed). Stand onyour tiptoes several times in a row. Hold on with your hands only if necessary.

This exercise strengthens the calf muscles.

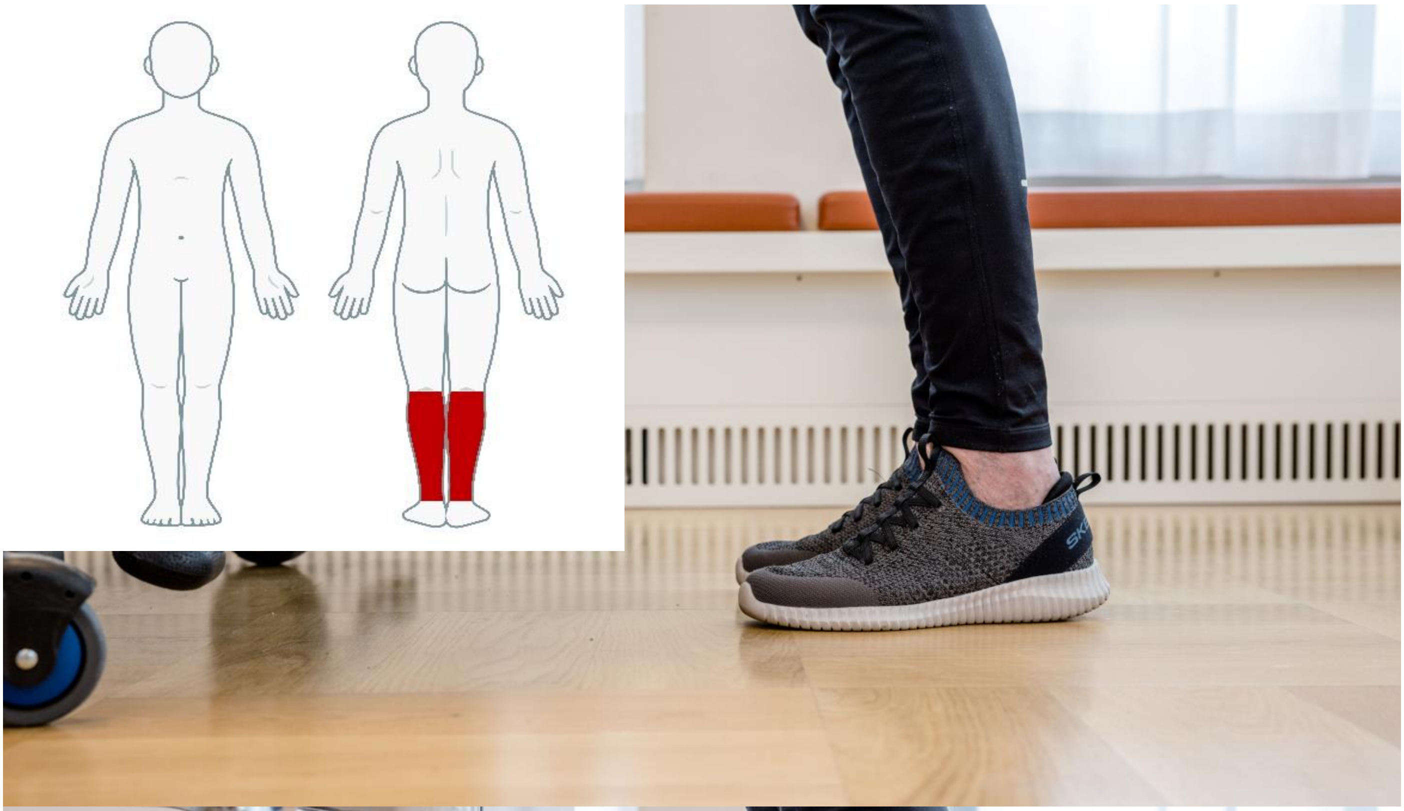

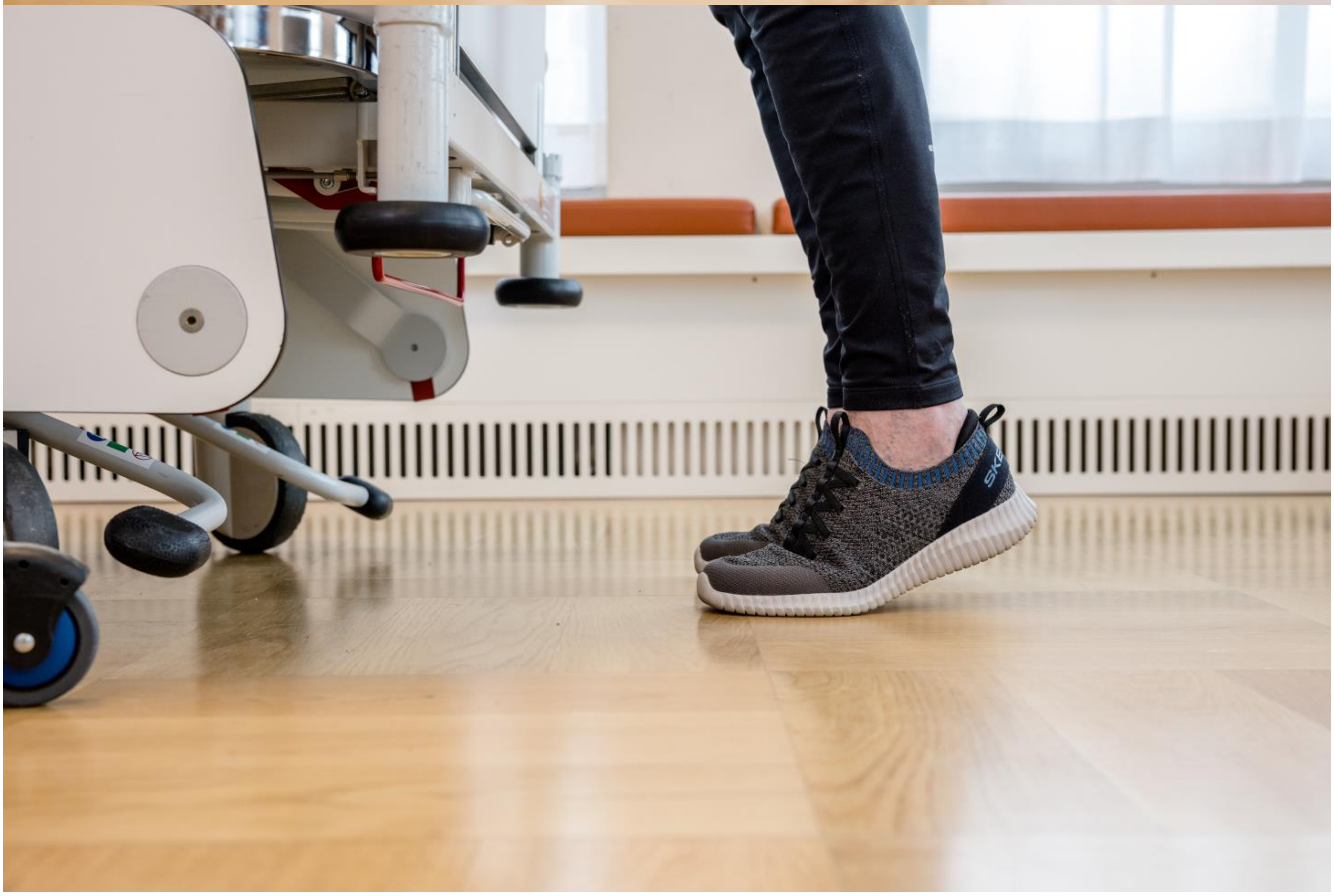

Push-ups (Video 21)

Do a few push-ups by leaning on the end of the bed or against a wall. The lower the bed, the harder the exercise.
This exercise strengthens the muscles of the trunk and arms.

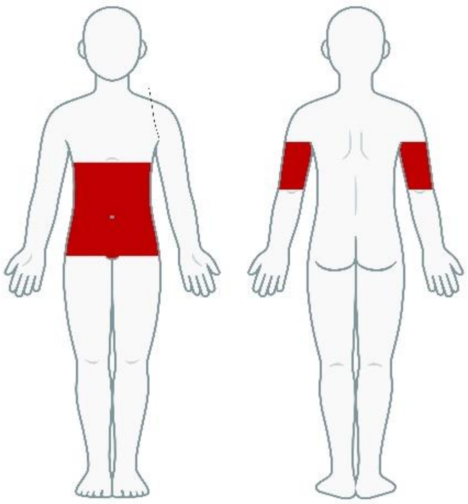

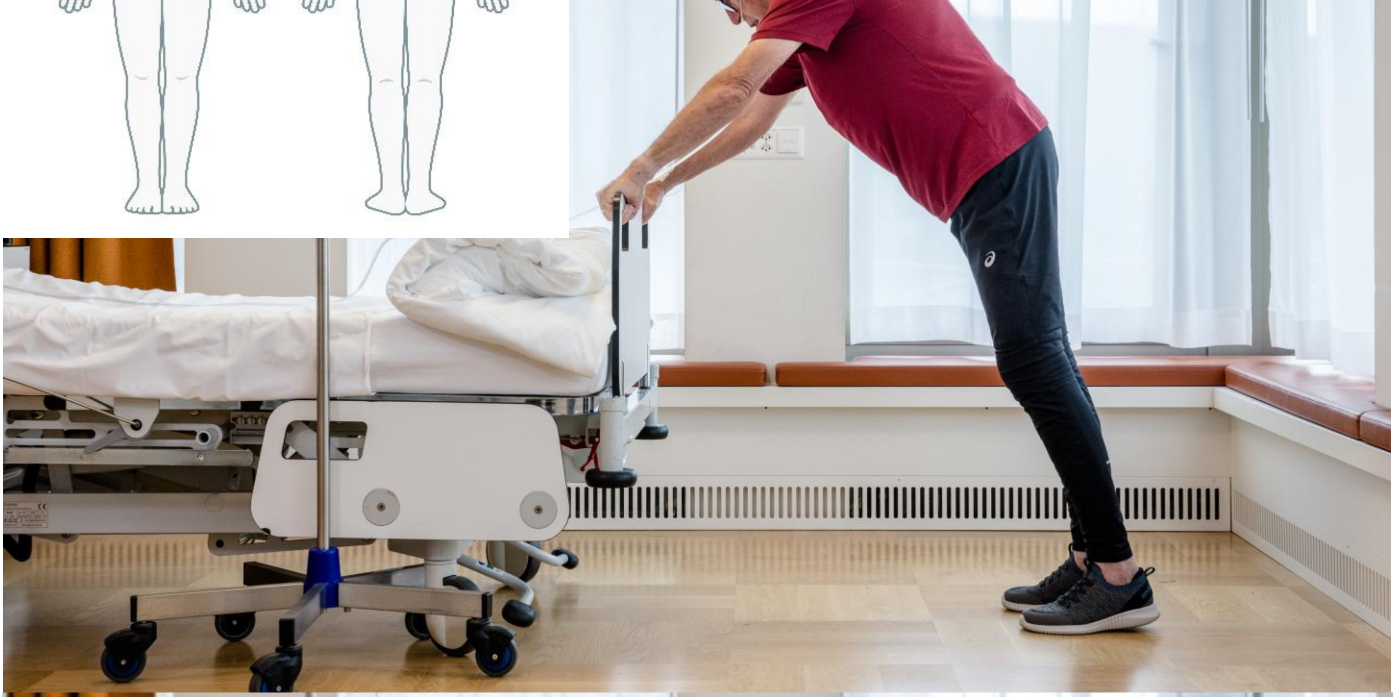

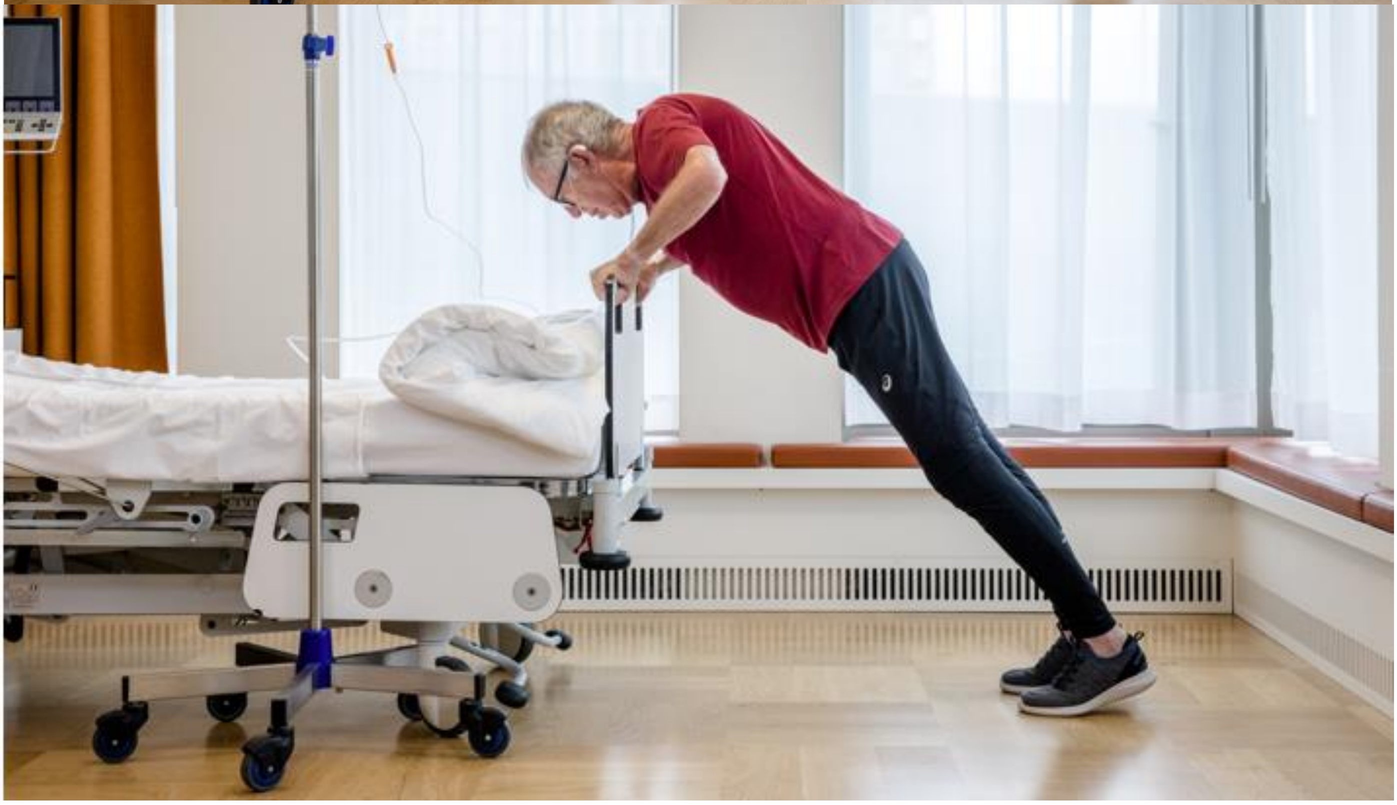

Lunge – step forward (Video 22)

Stand in a place where you can, if necessary, hold on with your hands (for example, at the end of the bed). Take a step forward with one leg, while bending both knees, so that the back knee almost touches the ground. Get up and repeat the exercise with the other leg forward.
This exercise trains dynamic balance and strengthens several leg muscles.

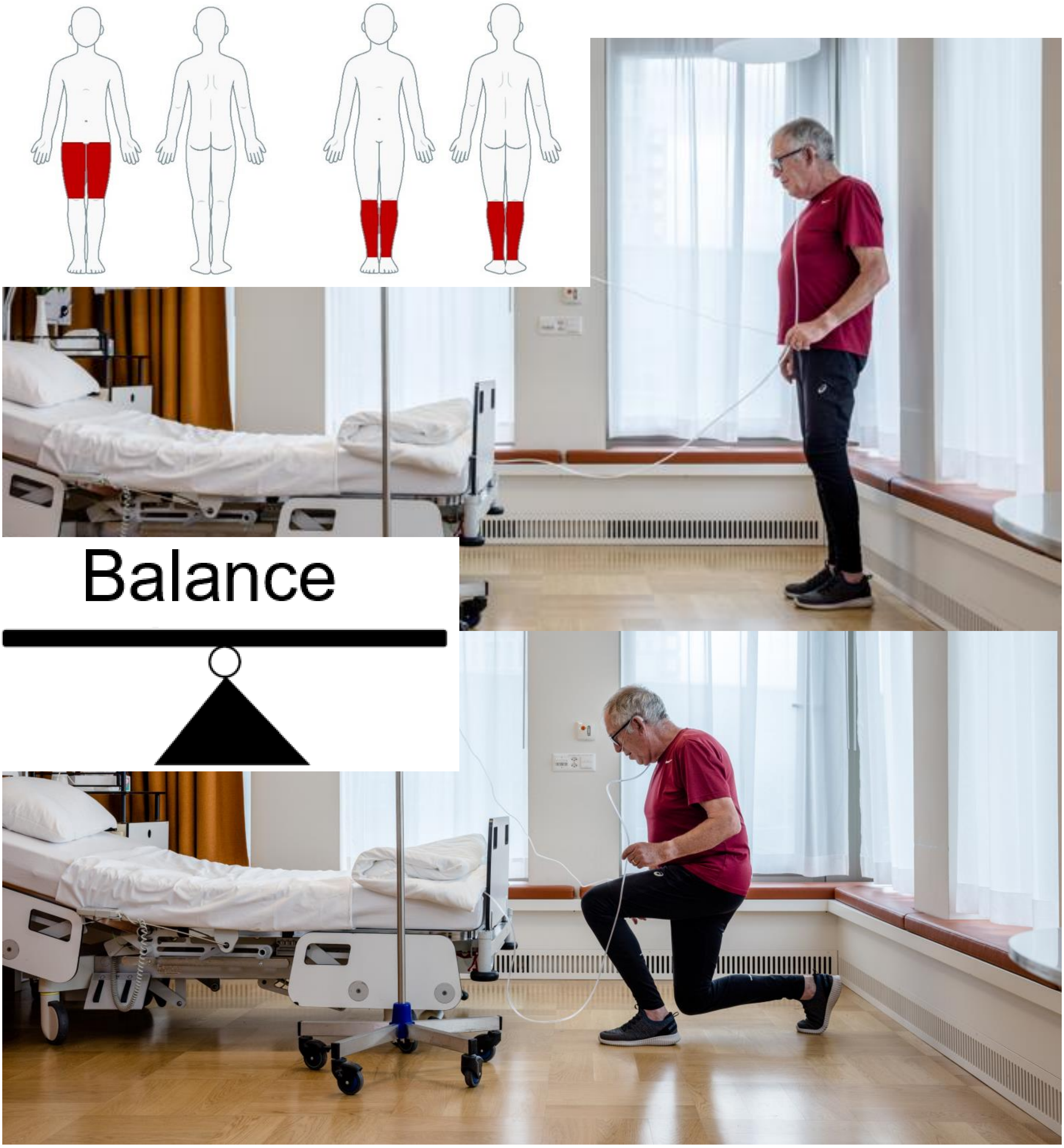

Dynamic balance (Video 23)

Stand in a place where you can, if necessary, hold on with your hands (for example, at the end of the bed). Fix a certain point on the ground with your eyes. Tap this point with your foot. Repeat the exercise with different imaginary dots, forward, sideways, or even behind you. The further the point, the more difficult the exercise.
This exercise trains dynamic balance.

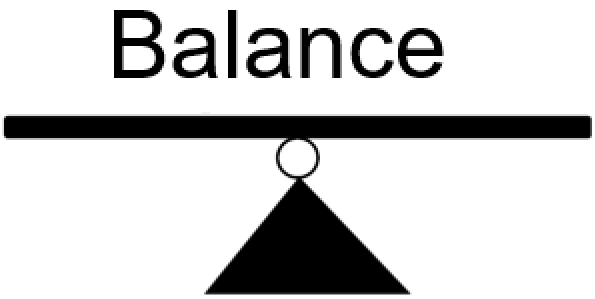

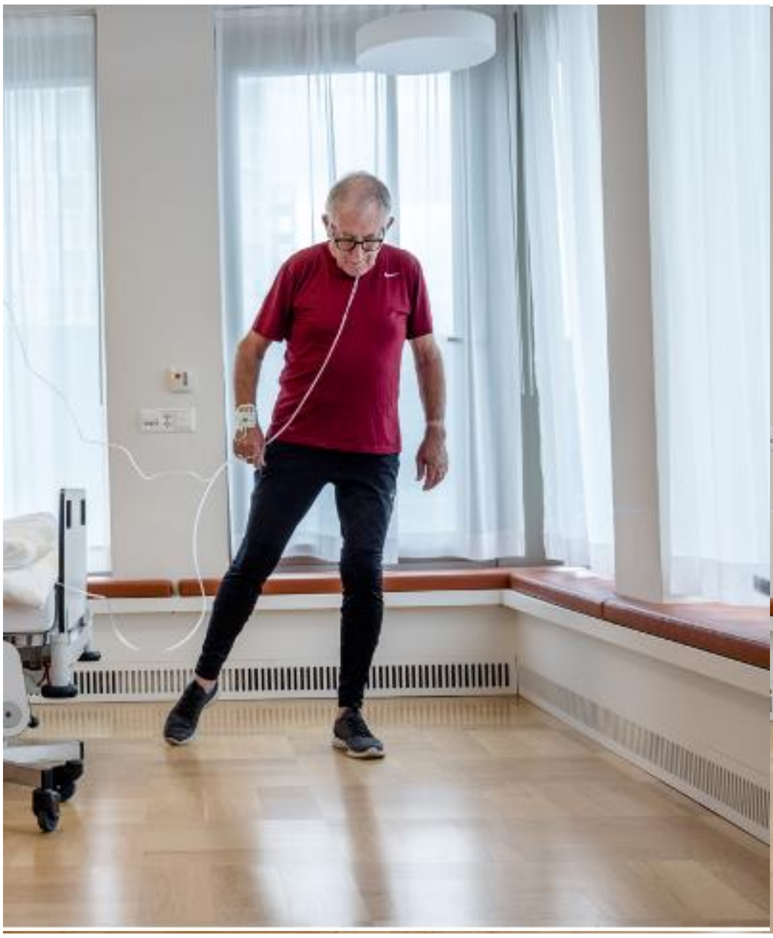

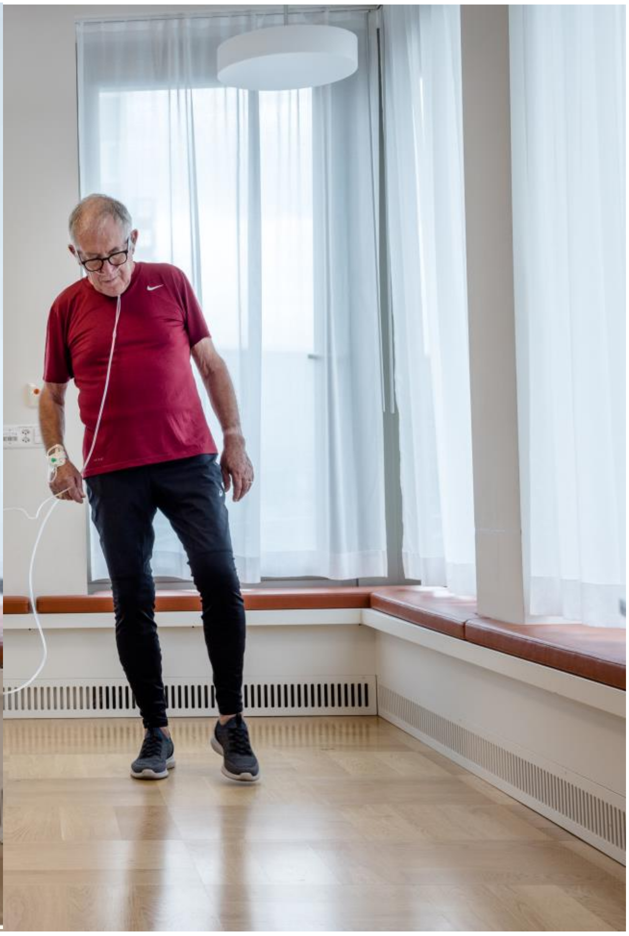

Leg to the side (abduction) (Video 24)

Stand in a place where you can, if necessary, hold on with your hands (for example, at the end of the bed). Stand on one leg and move the other leg away several times to the side, as far as possible. Hold on with your hands only if necessary. Repeat the movement several times, then switch legs.
This exercise strengthens the hip muscles.

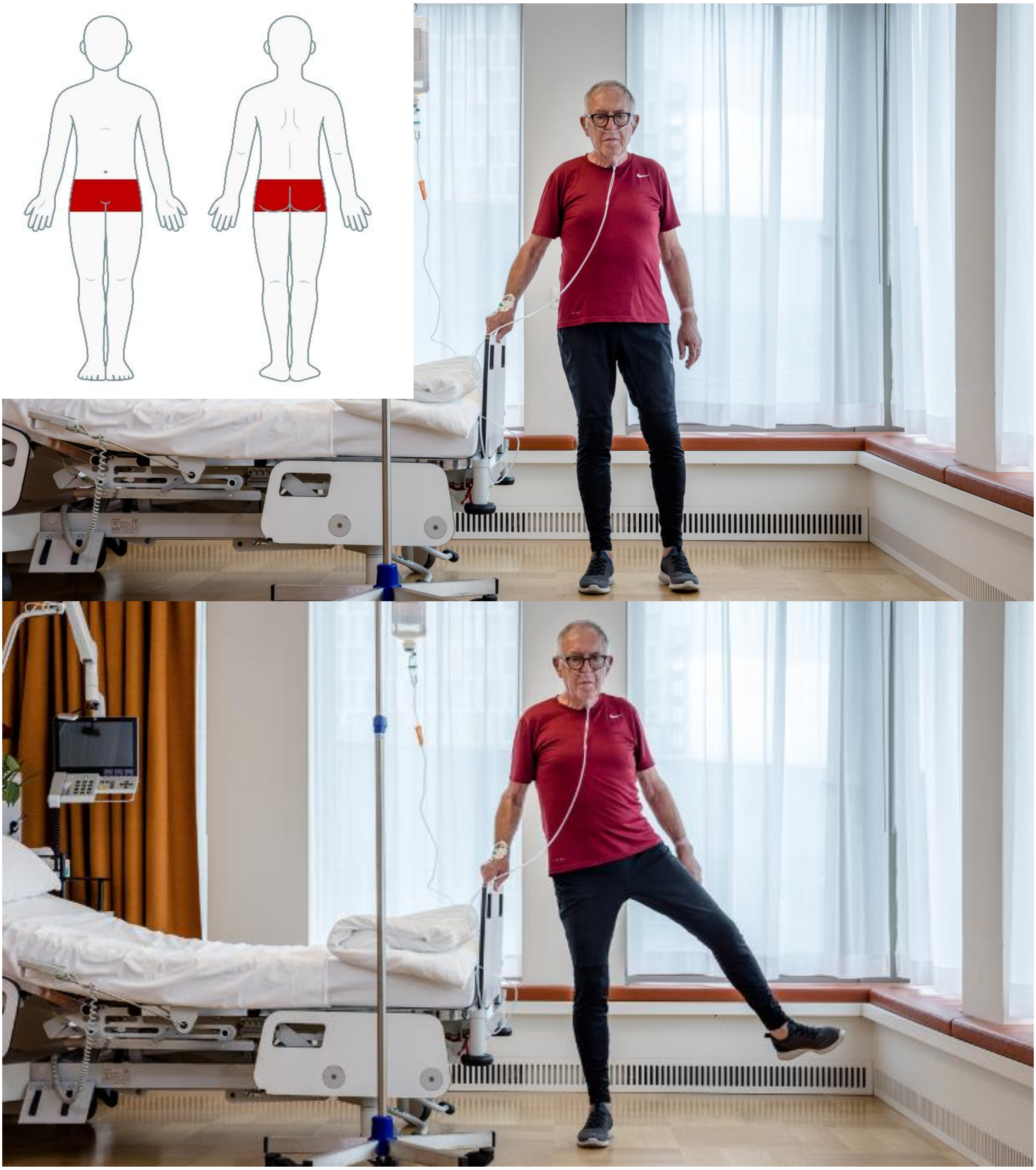

Leg on the side with elastic (Video 25)

Stand in a place where you can, if necessary, hold on with your hands (for example, at the end of the bed). Stand on one leg and move the other leg to the side as far away as possible, against the resistance of the elastic. Hold on with your hands only if necessary. Repeat the movement several times, then switch legs.

This exercise strengthens the hip muscles.

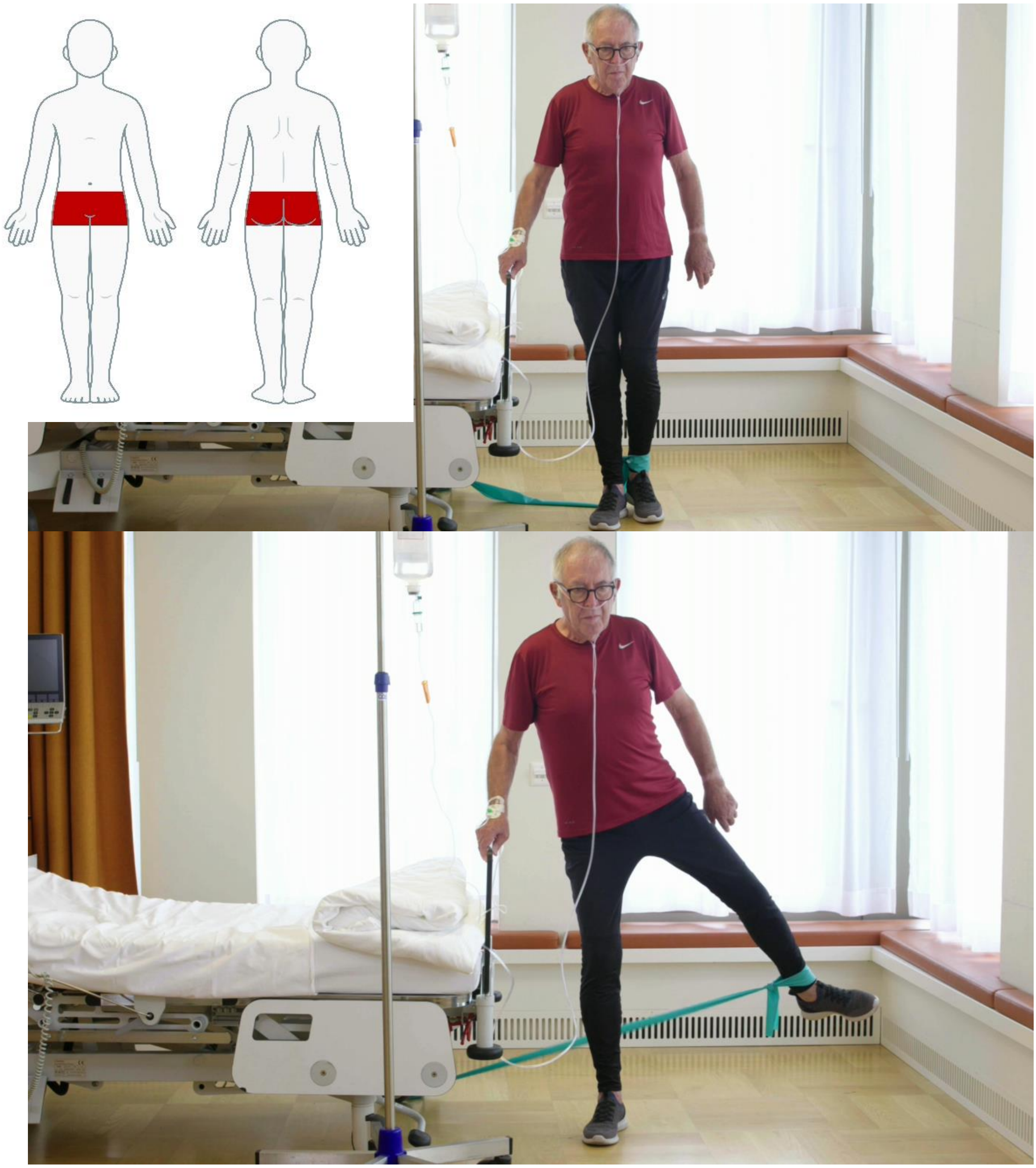

Walking safety (Video 26)

Walk in the corridor, if necessary with your auxiliary means (walker, cane, …), while fixing different points with your eyes, in front, on the side, or even behind you. The faster the gaze moves from one point to another and the farther the points are from each other, the more difficult the exercise.
This exercise trains walking safety.

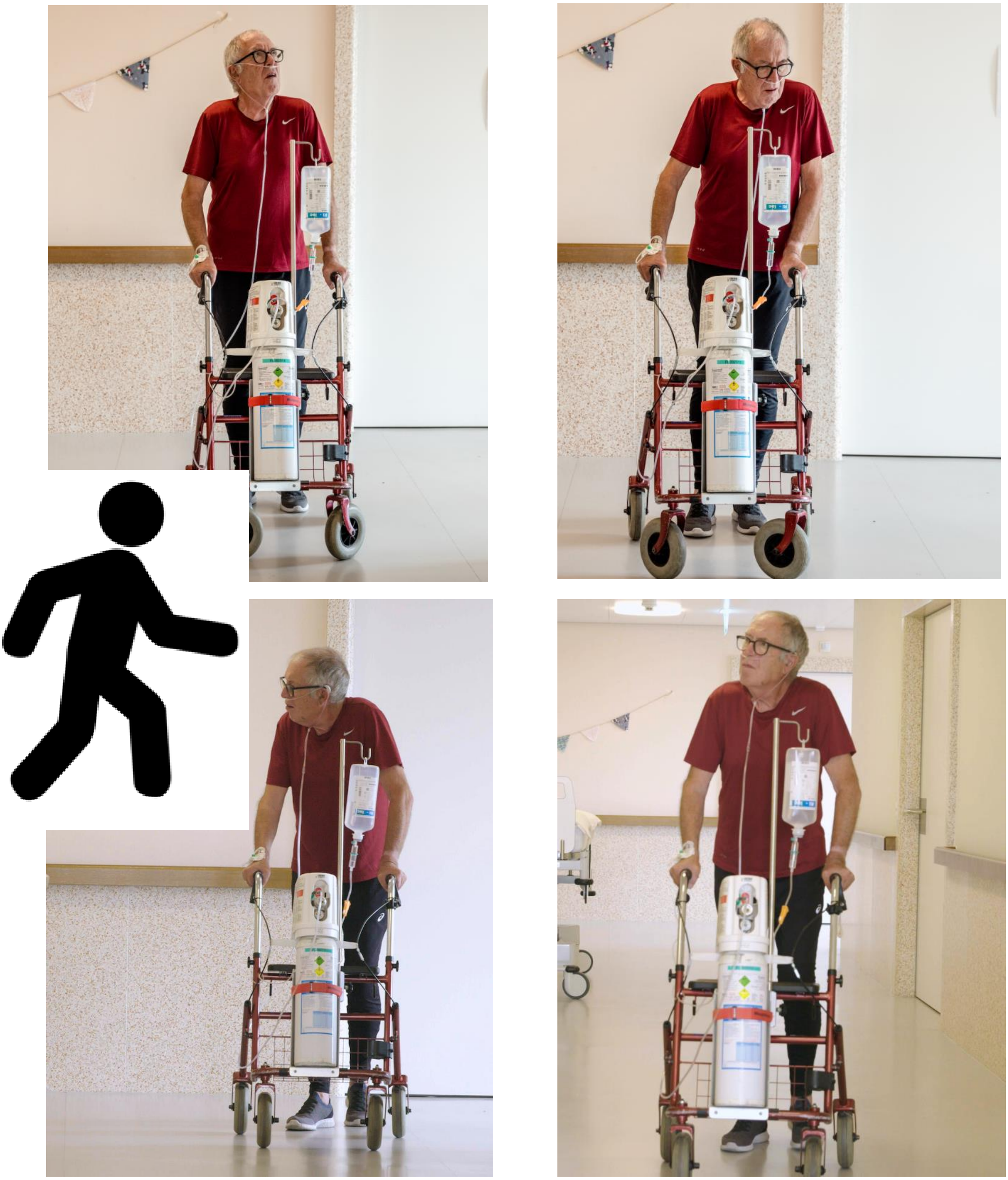

Walking sideways (Video 27)

Walk sideways (like a crab) along a wall one leg against the other or crossing your legs. Stand with your hands only if necessary.
This exercise trains walking safety.

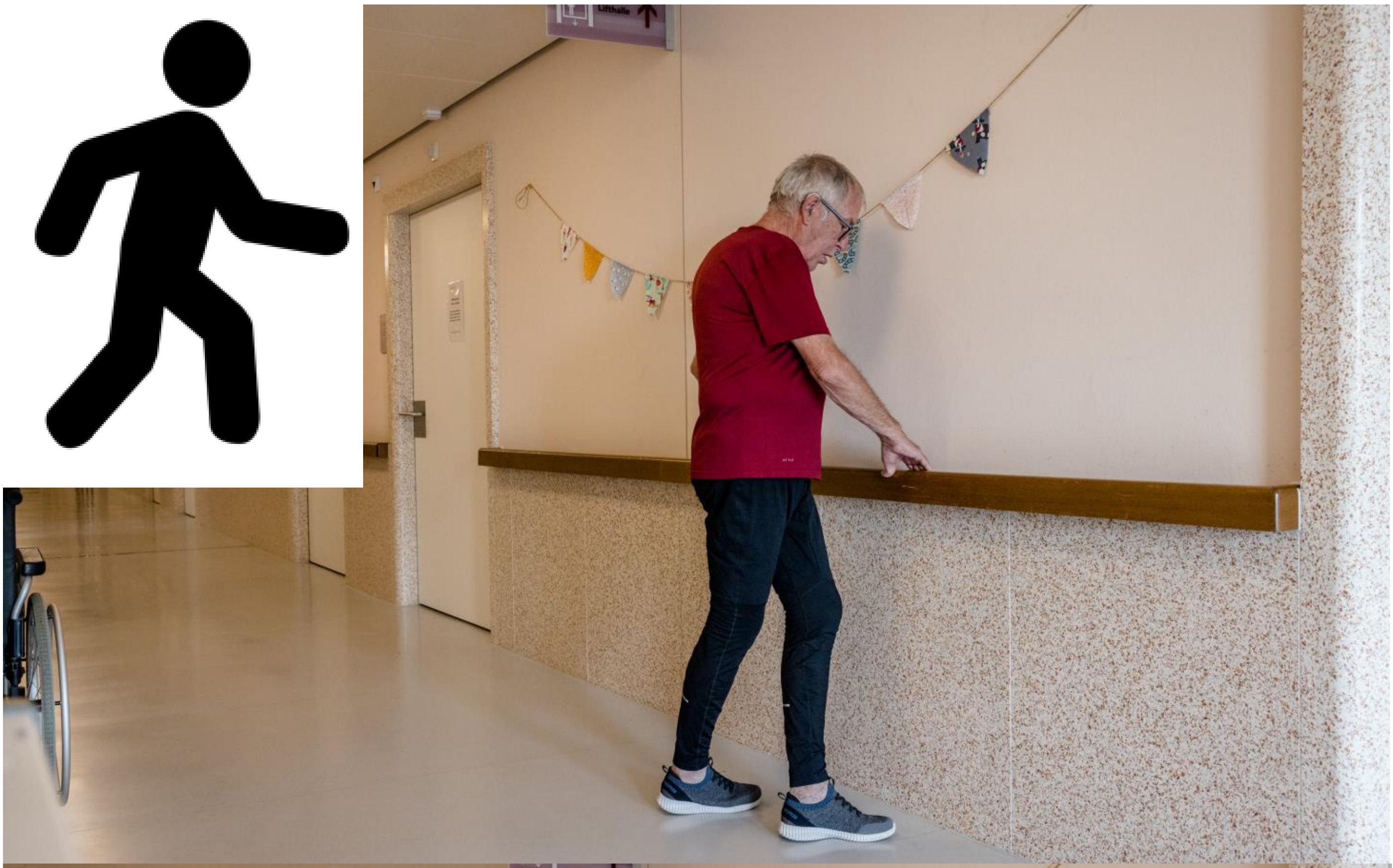

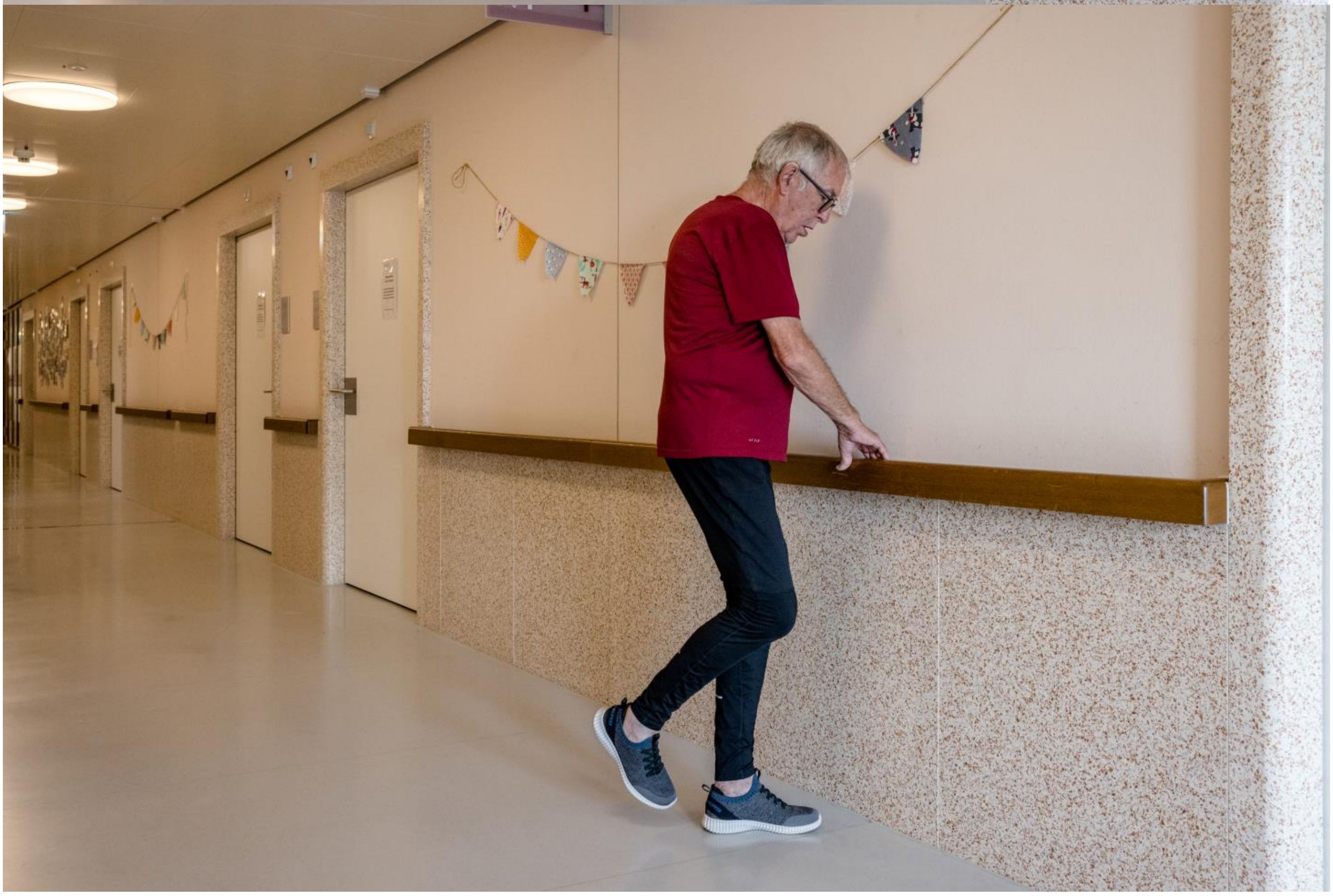

Climbing stairs (Video 28)

Go up and down the last step of the stairs several times in a row, keeping the body straight. The movements of the feet are as follows: 1) left foot up, 2) right foot down, 3) left foot down, 4) right foot down.
After a short pause, change direction: 1) right foot up, 2) left foot up, 3) right foot down, 4) left foot down.
Hold on with your hands only if necessary.
This exercise strengthens several leg muscles and stimulates blood circulation.

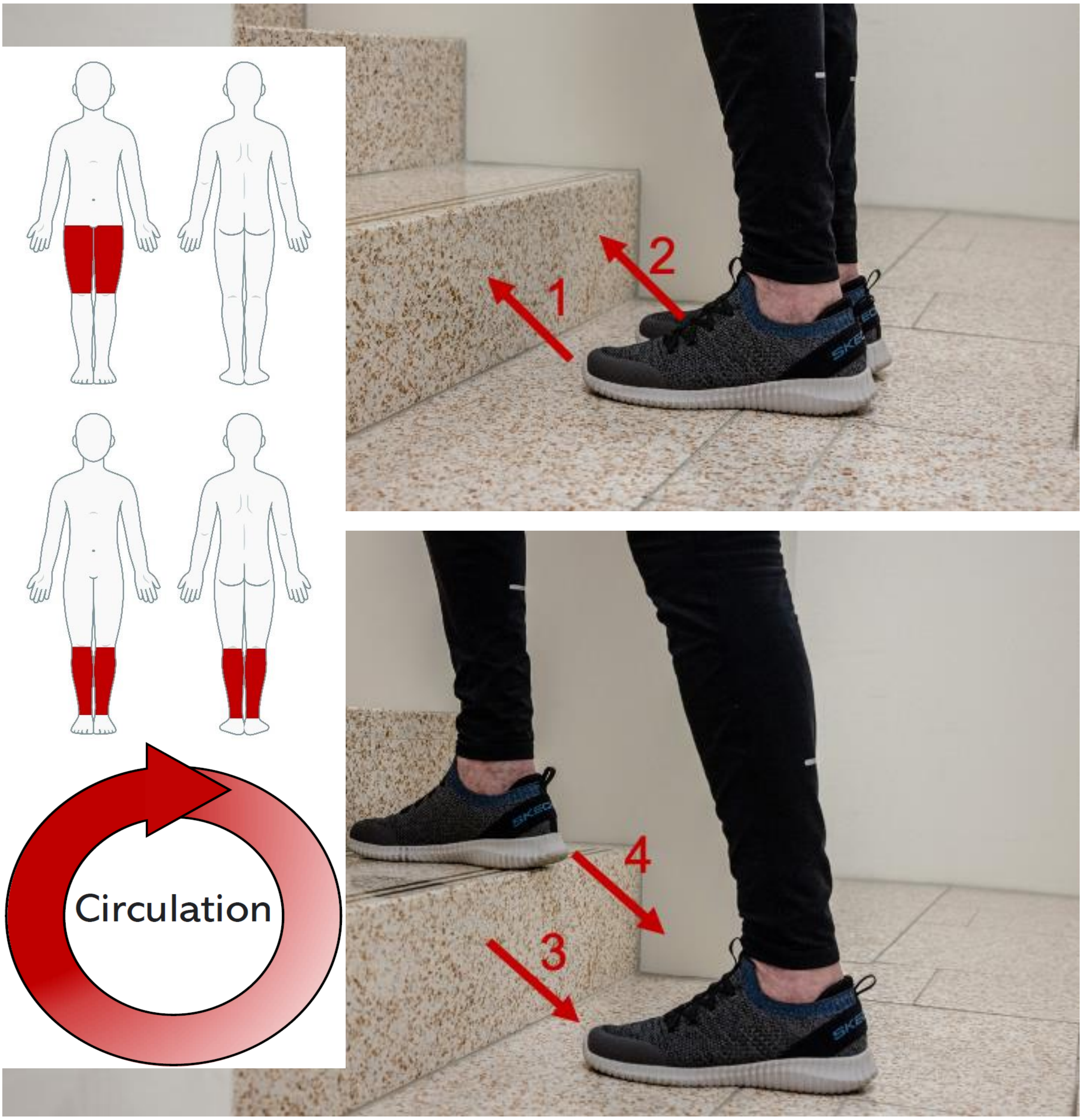

Bending the knees (Video 29)

Stand on the last step of stairs on the right leg and bend the right knee so that the left leg gets as close as possible to the bottom of the stairs. Then stretch the right knee again so that the left leg goes up. Repeat the exercise several times, then switch legs after a short break. Hold on with your hands only if necessary.

This exercise strengthens the thigh muscles.

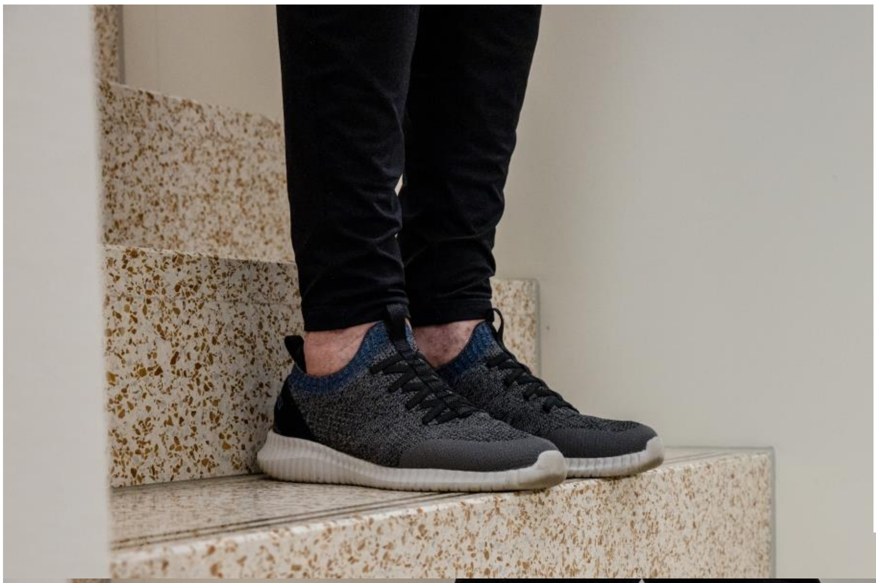

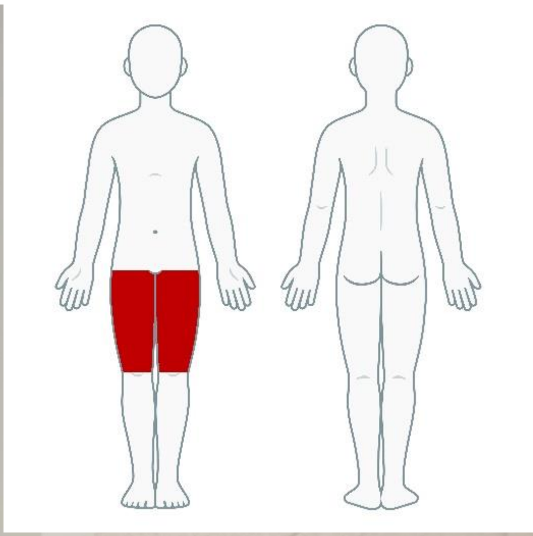

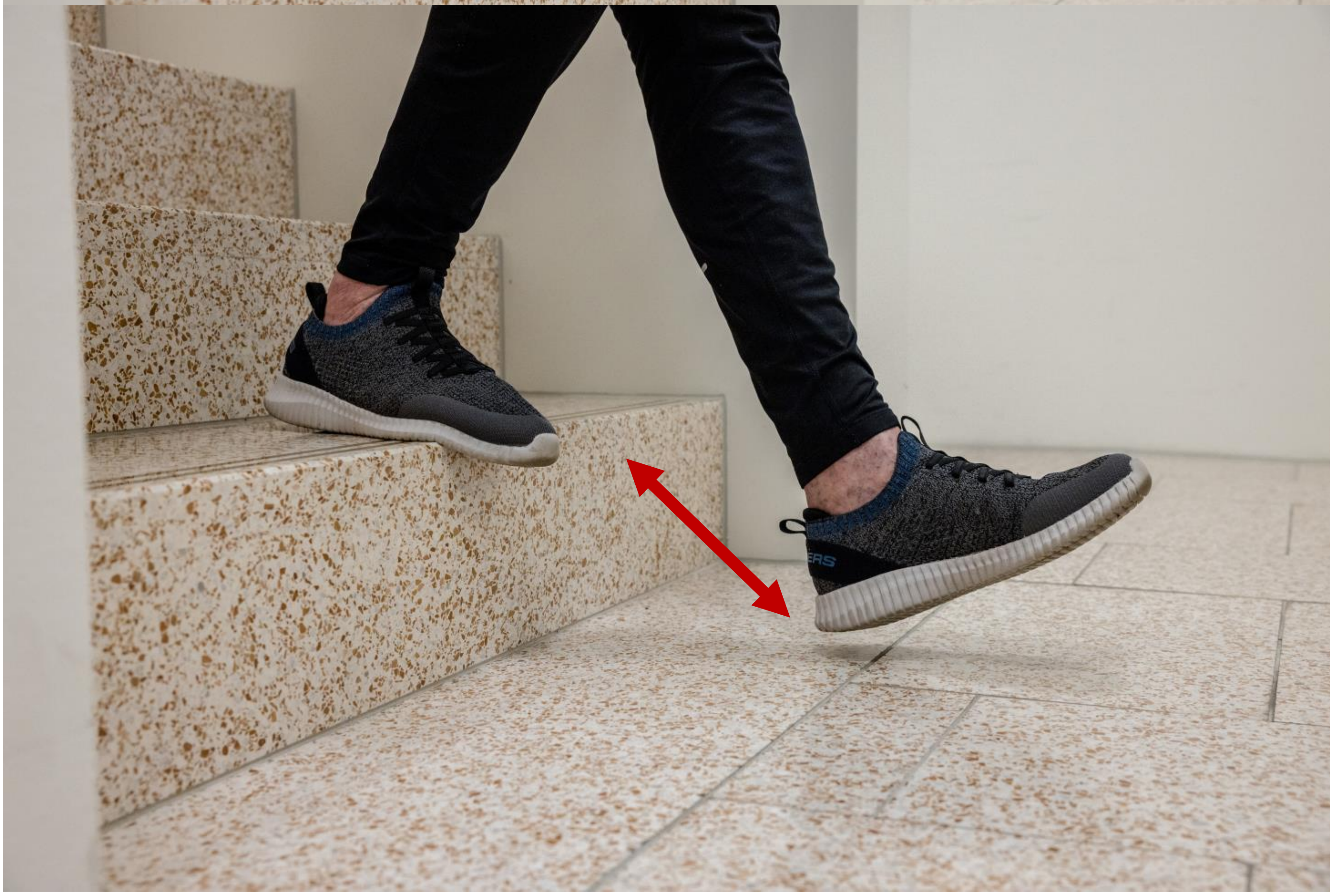

INTOMOB

Moving to maintain autonomy

A study from
Insel Gruppe AG, Bern

INSELGRUPPE

UNIVERSITÄT BERN

Supported by the Swiss National Science Foundation

FNSNF
FONDS NATIONAL SUISSE
SCHWEIZERISCHER NATIONALFONDS
FONDO NAZIONALE SVIZZERO
SWISS NATIONAL SCIENCE FOUNDATION

Contact

PD Dr. med. Carole Elodie Aubert, MD MSc
General Internal Medicine Clinic
Inselspital, Bern University Hospital

Pictures: P. Triponez, Kreation Inselspital; icons by Iyi Kon
