# Supplementary material for: Impact of an INtervention to increase MOBility in older hospitalized medical patients (INTOMOB): Study protocol for a cluster randomized controlled trial
Source: BMC Geriatr. 2023 Oct 31;23:705. doi: 10.1186/s12877-023-04285-3 (PMC10617203; doi:10.1186/s12877-023-04285-3)
Supplement: Supplementary file 4 — Additional file 4: Supplement 4. a. E-learning residents. b. E-learning nursing staff. [file 12877_2023_4285_MOESM4_ESM.zip › Supplement 4b - E-learning nursing staff.pdf]

Mobility of older hospitalized people

This e-learning is about "Mobility of people aged 60 and over during an acute care hospitalization".

Learning Objectives

At the end of this e-learning, you will know about:

1) The recommendations for mobility mobility during an acute hospitalization and their implementation.
2) The INTOMOB intervention to improve mobility during an acute hospitalization.

Content

- Consequences of low mobility during an acute hospitalization
- Barriers and facilitators
- Recommendations, goal setting, documentation
- Interdisciplinarity
- Communication
- INTOMOB Intervention
- Putting it into practice

To be noted

This e-learning is for nursing staff, including nurses and nursing assistants.

Another e-learning was developed for the physicians.

Consequences of low mobility

Low mobility during an acute hospitalization leads to negative consequences:

- Vertigo, thromboses, embolism, tachycardia
- Muscle loss (5% per day spent in bed) and bone loss, falls
- Loss of appetite, malnutrition, constipation
- Pneumonia
- Anxiety, depression, delirium
- Sleep difficulties with disturbance of the nycthemeral rhythm
- Ulcers
- Loss of autonomy, institutionalization
- Prolonged length of hospital stay
- Death

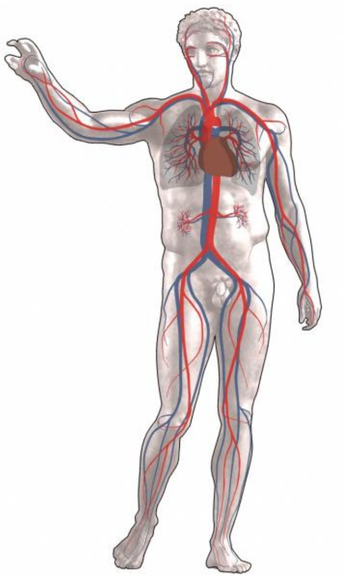

Barriers to mobility

There are many barriers to mobility. Many of them can be modified.

- ✓ **Patients' false beliefs:** «If you are sick, you should rest in bed», «With catheters/perfusion, you cannot move», «If I leave my hospital room, I will miss a round or a medical examination», «Nothing will happen if I rest in bed for a few days only»
- ✓ **Medications**: Anticholinergics, antidepressants, antipsychotics, hypnotics, antiepileptics, antihypertensives, diuretics, alpha blockers, opioids
- ✓ **Tubes / catheters / drains / perfusion / oxygen**
- ✓ Alarm mats
- ✓ **Symptoms:** Pain, difficulties breathing, tiredness, cognitive disorder
- ✓ **Sleep disorders**
- ✓ **Fear of falling** (from staff and patients)
- ✓ **Lack of staff time**: patients notice this and, as a result, are reluctant to ask for help
- ✓ Lack of **stimulation** / lack of motivation

Facilitators to mobility

Here are some mobility facilitators:

- ✓ **Walking aids**
- ✓ **Accompaniment by another person**
- ✓ **Information** (what, when, how, where, how often, ...)
- ✓ Wearing one’s **own clothes instead of a hospital gown**
- ✓ **Daily schedule**
- ✓ **Setting mobility goals**
- ✓ Welcoming **environment**, available **space**

Question: Consequences of low mobility

What are consequences of low hospital mobility?

Several answers are possible.

1) Muscle loss
2) Hypertension
3) Increased risk of fall
4) Diarrhea
5) Sleep disorder

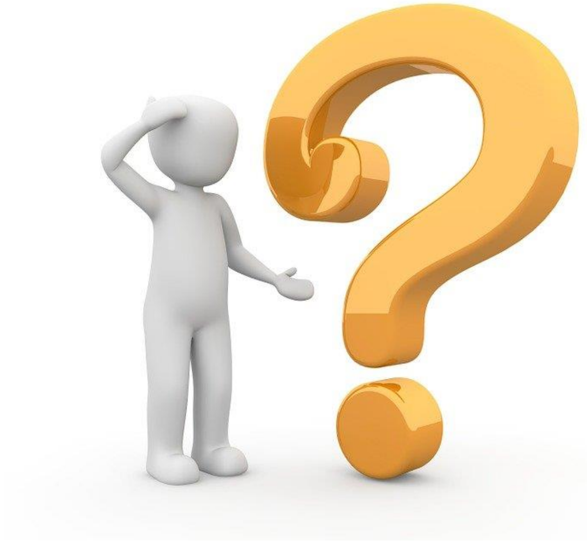

Question: Barriers to mobility

What are the barriers to mobility during an acute hospitalization?

Several answers are possible.

1) A urinary catheter
2) Wearing one's own clothes
3) Using a walking aid
4) Hypnotics
5) An alarm mat

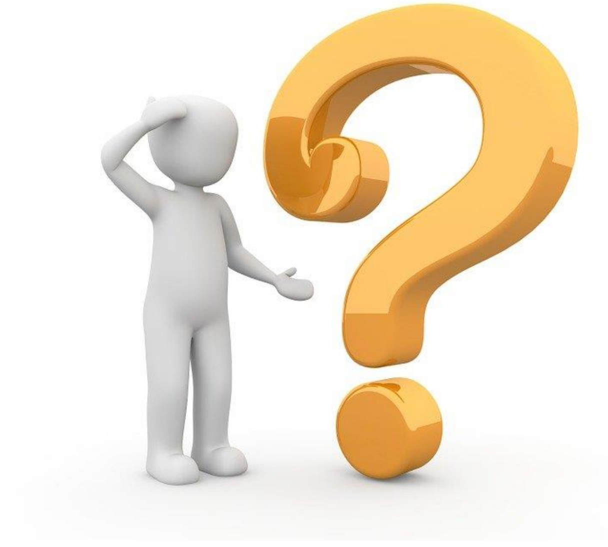

Recommendations: Assessing mobility

Mobility assessment includes:

- Abilities: What the person **can** do
- Behavior: What the person **actually does**

Mobility bef**o**re hospitalization should be assessed by the **nursing staff and the physician** at admission and discussed **interdisciplinary**.

Current mobility should then be assessed **daily** during an acute hospitalization (because it can change quickly) by the **nursing staff**, and if needed discussed interdisciplinary.

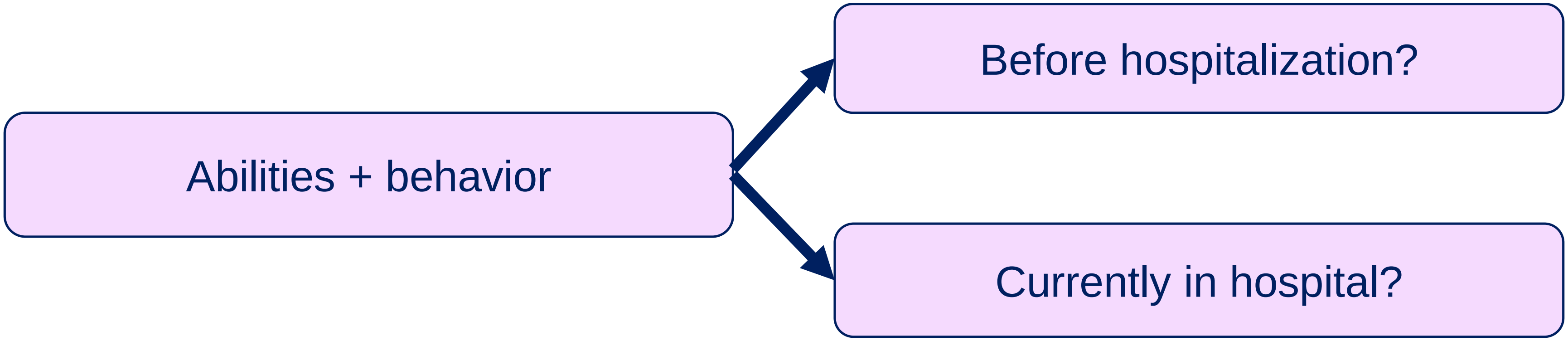

Information

Often, patients do not know what they are allowed to do, or even that they should move. They wait for an authorization and for recommandations.

Please inform your patients:

- Mobility: What, when, where, how, how often
- Goals of mobility during acute care hospitalization: main goal = to maintain autonomy

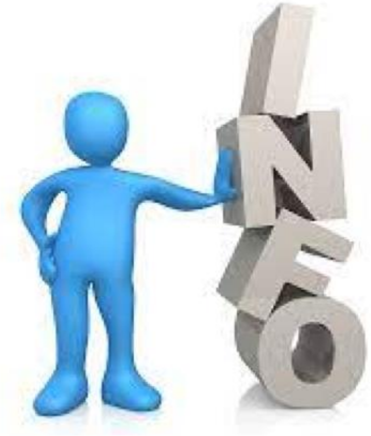

Moving to maintain autonomy

Recommendations: Set goals

In general, patients should achieve their individual goals **at least three times a day**. These goals should require a little effort.

Please **discuss daily those goals** with your patients (+/- with their family/friends) and, if needed, interdisciplinary with physicians / physiotherapists..

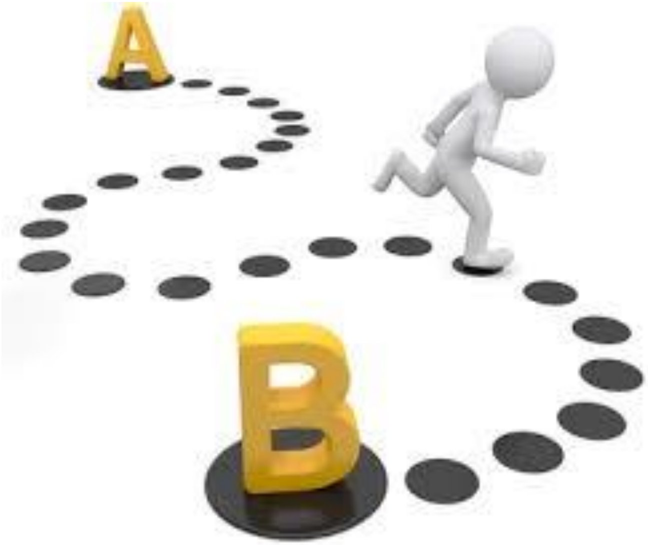

Question: Main goal of mobility at hospital

What is the main goal of mobility during an acute hospitalization?

Only one answer is correct.

1) Weight control
2) Diabetes prevention
3) Maintenance of autonomy
4) Prevention of hypertension
5) Improvement of endurance

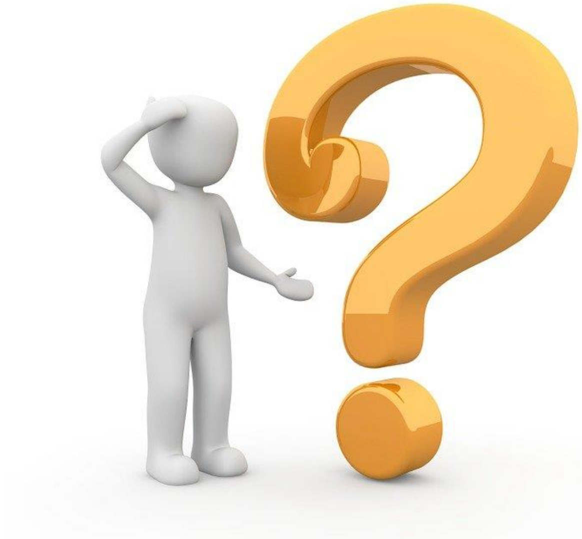

Clinical case 1

A 75-year-old patient, visually impaired and with walking insecurity, is hospitalized because of an ulcer on his left leg. He refuses the walker, saying he is not disabled, and insists on asking you for a Zolpidem for the night (his wife takes this medication).

What do you tell him (one correct answer)?

A. That there is no need to take a walker if he doesn't want to.

B. That you can prescribe a Zolpidem during hospitalization, but not for home use.

C. That a walker does not mean that one is handicapped, but it can secure the walk and prevent falls.

D. That he must rest as much as possible so that the ulcer heals.

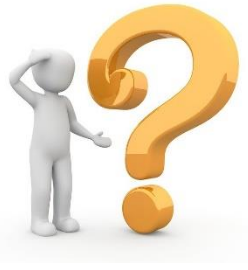

Documentation

Documenting mobility (what, how often, how long) in patient file helps to:

1) Consider mobility as a priority.
2) Plan and organize hospital discharge.
3) Work interdisciplinary.

Interdisciplinary work

- Physiotherapy is important, but not enough!
- Physicians, nursing staff and physiotherapists have a complementary role in improving patient mobility.
- It is important to communicate and read the notes of colleagues in other disciplines.
- Involve **family members / friends** in the **team** as well**!** Encourage them to accompany/support the patients during their mobilization.

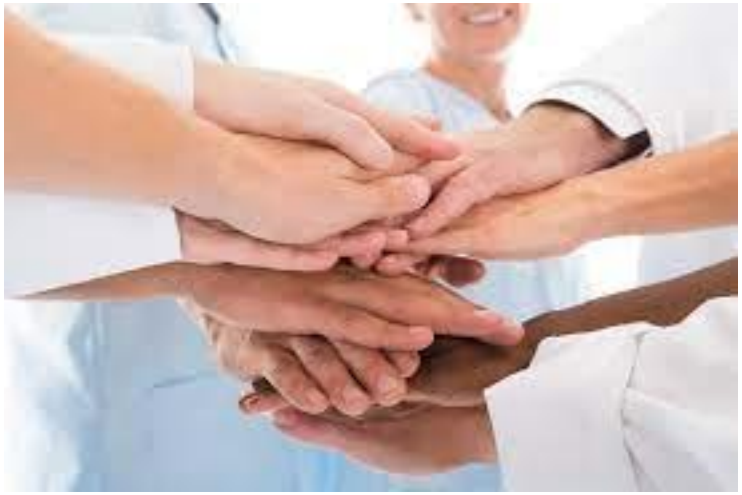

Communication

The way in which patients are approached plays an important role in improving their mobility, especially when motivation is lacking.

You should choose your words carefully to avoid causing additional stress.

We will now show you 2 videos as examples:

1) Video 1: Counterproductive discussion
2) Video 2: Motivational discussion

These videos are **caricatures** and should not be interpreted in any way as personal or reproachful.

Video 1 (2:11): Counterproductive discussion

LINK PROVIDED

Interview: Video 1

This interview was inadequate for the following reasons:

- Physician and nursing staff standing - patient lying down
- Little space left for the patient during the discussion
- No discussion about false beliefs (*resting = pain goes away*), nor about fear and obstacles (*pain, not wanting to, ...*)
- Purpose of mobility not explained
- Reproaches made towards the patient
- Responsibility for movement left to the patient and the physiotherapist

Video 2 (3:26): Motivational discussion

LINK PROVIDED

Interview: Video 2

There are many positive elements in this second video:

- Physician and nursing staff at the same height as the patient
- Empathy ("*I understand your wish and your fear*")
- Meaning and purpose of mobility explained
- Discussion of barriers and facilitators
- Mobility integrated in activities of daily living
- Objectives set together + discussion of the help needed: What, how often, where, how

A comprehensive implementation takes time, but many aspects that help to improve mobility in the hospital do not actually take extra time! We give you later on some tips and tricks for implementation.

Clinical case 2

Your 82-year-old patient, hospitalized for acute heart failure, suffers from a mechanical fall during the night, without traumtatic consequences, while going to the bathroom.

What do you tell her (several answers possible)?

A. That you will install an alarm mat to prevent future falls.
B. That she should not move unaccompanied.
C. That she will get a urinary catheter to avoid future trips to the bathroom.
D. That she should take advantage of the hospitalization to rest.
E. That it is important that she moves to improve her breathing and avoid future falls.

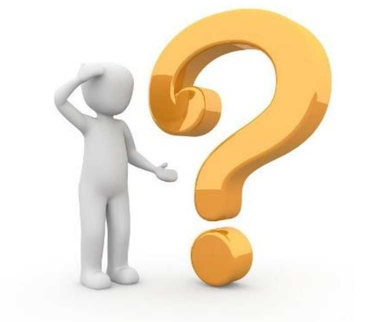

The INTOMOB study

We now present to you the INTOMOB study. It tests an intervention that aims to improve the mobility of people aged 60 years and older who are hospitalized.

The INTOMOB intervention is subdivided into :

1) Intervention for patients
2) Intervention on the hospital environment
3) Intervention for medical and nursing staff

Please DO NOT discuss the intervention with your colleagues in other units to avoid biasing the results of the study.

INTOMOB

INTOMOB: Design

- Randomized trial in the units of general internal medicine.
- Comparison of the INTOMOB intervention with standard of care.
- Patient follow-up over 6 months.
- Primary outcome: Life-Space Assessment (= how much patients move from their bedroom to outside town).

INTOMOB

INTOMOB : Randomization

Randomization means that it is randomly decided whether a unit receives the intervention or the control procedures.

Medical units (and not patients) are randomized.

- Healthcare professionals working on a «control» ward and patients admitted there and who agree to participate to the study → «control» group.
- Healthcare professionals working on an «intervention» ward and patients admitted there and who agree to participate to the study → «intervention» group.

Your medical unit is in the «intervention» group => please do not speak about the study with colleagues from other units.

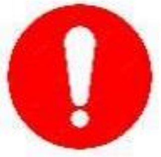

INTOMOB: Patient inclusion and exclusion criteria

Inclusion criteria

- 60 years or older
- Planned length of stay ≥ 3 days
- Mobile during the last 2 weeks before admission
- Able to speak/understand French or German

Exclusion criteria

- Wheelchair
- Bedrest
- Severe psychiatric disease
- Delirium
- Severe visual disorder
- Dementia (except if proxy’s support)
- Living in nursing home

INTOMOB: Intervention for patients - 1

Patients participating in the INTOMOB intervention receive (from the study team):

1) An **information brochure** with explanations of the consequences of lack of mobility, as well as the purpose and practice of mobility

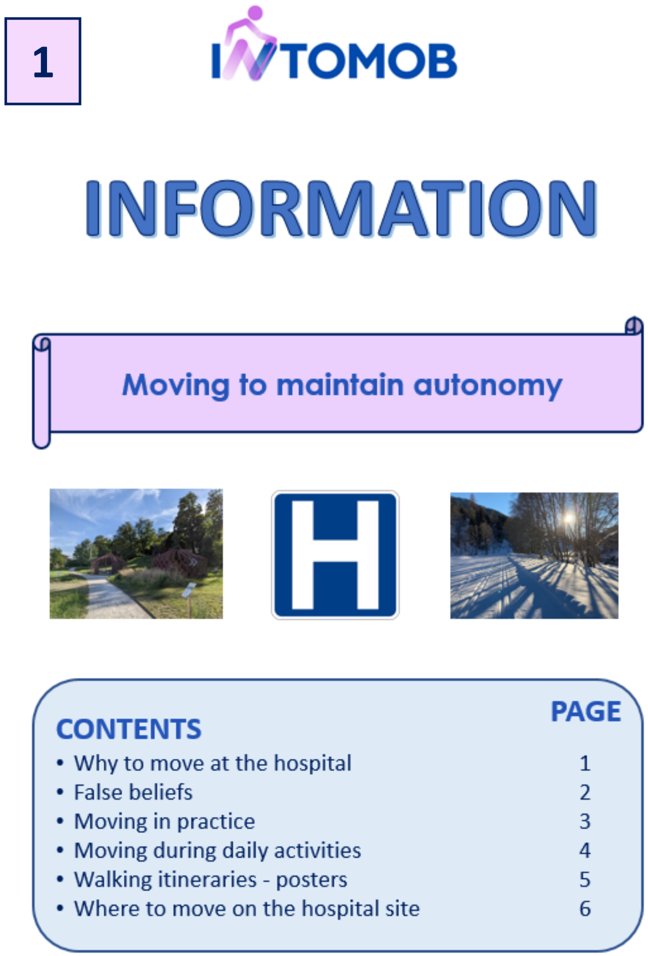

INTOMOB: Intervention for patients - 2

2) A **diary** for daily documents of objectives, outcomes, challenges and needs.

Nursing staff should help patients to set objectives.

As a physician, you help in case of difficulties.

2

INTOMOB

DIARY

Moving to maintain autonomy

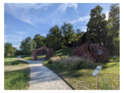

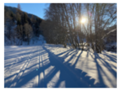

CONTENTS

- Introduction regarding the use of the diary
- Situation before the hospitalization
- Support for your objectives, results, difficulties and needs

OBJECTIVES

DATE ______________________

Here you can find examples of goals. You don't have to achieve them all!
Choose a few goals that suit you.
You can add easier or more difficult objectives!

Objectives	Frequency?	Help?*	Reached?
Doing exercises in bed.			
Sitting up on the side of the bed			
Moving from the bed to the chair			
Moving around the room			
Moving around the corridor			
Going up/down the stairs			
Walking around outside of the unit			
Going to the cafeteria			
Going to the bathroom			
Showering			
Dressing with my own clothes			
Eating at the table			
Doing exercises sitting up			
Doing exercises standing up			

INTOMOB: Intervention for patients - 3

Patients participating in the INTOMOB intervention receive (from the study team):

3) An **exercise booklet**: 29 exercises while lying, sitting and standing, with photos and explanations

3

INTOMOB

EXERCISES

Moving to maintain autonomy

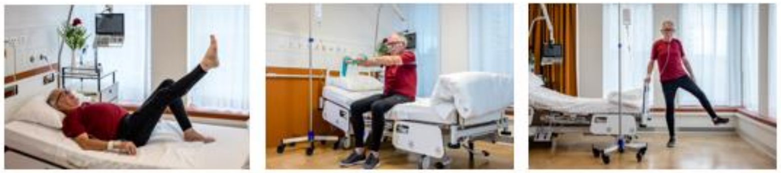

CONTENTS **PAGE**

✓ Introduction	1
✓ Exercises lying down	2-11
✓ Exercises sitting up	12-19
✓ Exercises standing up	20-30

INTOMOB: Intervention for patients - 4

4) A **10.2" iPad** (available during hospitalization) to access the **videos** of the exercises in the booklet

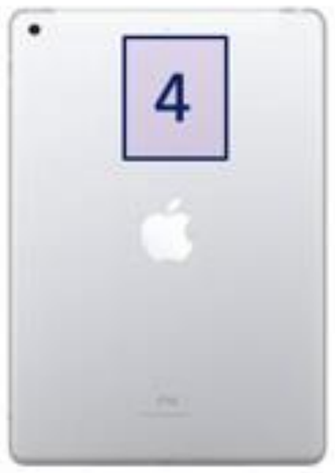

In addition, the movement of the patients is measured by an **accelerometer** which is waterproof and must be removed only for MRI.

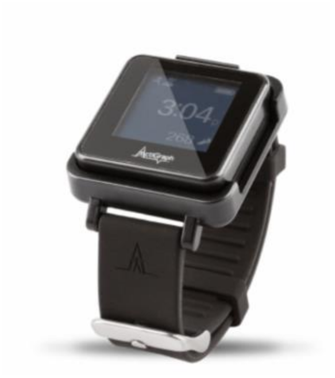

INTOMOB: Intervention for patients – 5

Some exercises can be done with a TheraBand. You can give max. 2 TheraBands to each patient with a request and without physiotherapy (box with TheraBands in nurse office).

TheraBand's level of strength according to exercise type and patient strength (can be assessed by nursing staff):

Exercice	Frail patient	Strong patient
With arms	Level 1	Level 3
With legs	Level 3	Level 4

INTOMOB: Intervention for patients – 6

You can find patient booklets in the nursing office.

We recommend that you have a look at them!

You can also access them online (please do not forward the links):

Booklets and other study documents:

LINK PROVIDED

Exercises (videos) :

LINK PROVIDED

INTOMOB: Intervention on the environment

To stimulate mobility and to make patients feel welcome in the hallway, we created posters and walking paths that are hung in the unit.

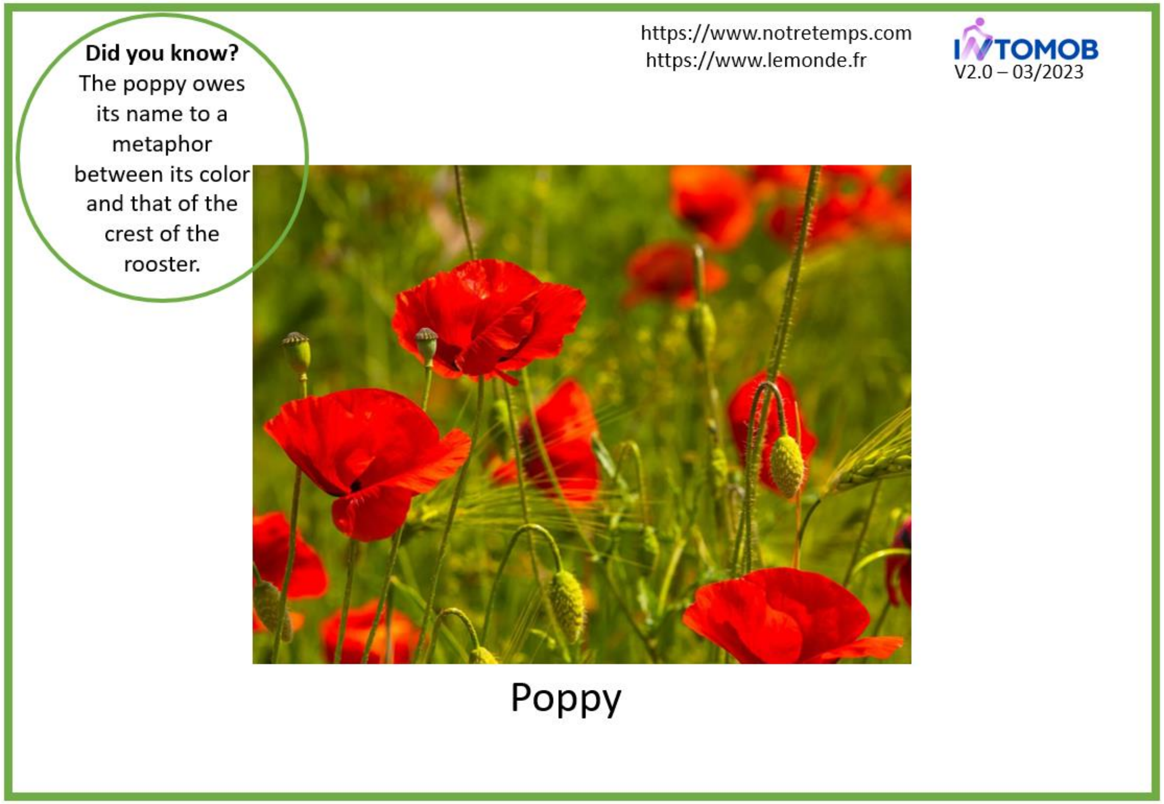

Checklist

Nursing staff receives a checklist to check daily.

This checklist helps you to assess barriers and facilitators to mobility:

- ✓ Can **a barrier be avoided** (catheter, medication, …)?
- ✓ **Is a facilitator missing** (walking aid, physiotherapy, mobility goal)?

Please touch on the checklist during medical round if needed!

You can find the checklist on the unit, in the offices and on the visiting carts.

MOBILITY CHECKLIST

PLEASE CHECK DAILY!

1) Catheter, tube, drainage, perfusion to remove?

2) Mobility-limiting medication to stop?

=> anticholinergics, antidepressives, hypnotics, opioids, antipsychotics, antiepileptics, antihypertensives

3) Mobility aid needed/available?

4) Physiotherapy needed/prescribed?

5) Discuss mobility objectives and behaviors

6) Document mobility!

Question

When must the accelerometer of the INTOMOB study be removed (only one answer is correct) ?

A. Never.
B. For sleeping
C. For an MRI.
D. For a CT.
E. For showering.

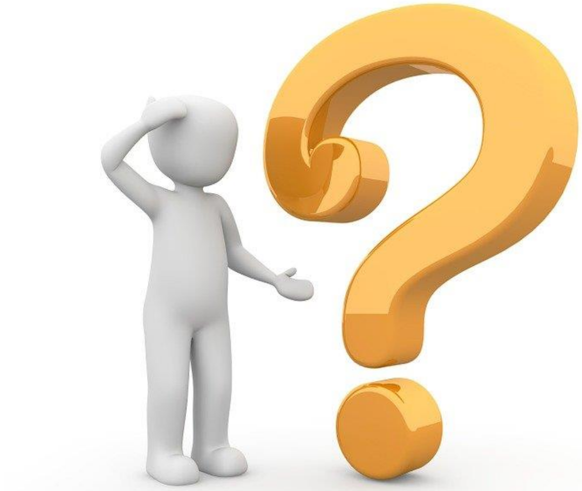

Tasks of the research team

- Check inclusion/exclusion criteria: medical file + questions to the healthcare professionals of the ward: Delirium? Planned length of stay?
- Explain the study to the eligible patients
- Sign the informed consent
- Give and explain the material to the patients
- Set up and install the accelerometer
- Help for technical issues
- Admission and discharge examinations for the study
- Pick up the iPad and accelerometer in the nursing office after discharge

Tasks of the nursing staff in INTOMOB

- Set objectives and discuss goals and difficulties with patients
- Motivate the patients! Discuss difficulties with the patients and advise them
- Give a TheraBand if wished and if no physiotherapy prescribed
- Verify the checklist 1x/day => discuss it if needed with the physician
- Document patient mobility in patient file
- Help with the use of the iPad
- Help to put on/off the accelerometer
- Inform the research team about discharge or transfer (task of the nursing and medical staff)

Tasks of the medical residents in INTOMOB

- Discuss objectives and difficulties with the patients, advise them
- Discuss checklist items if touched on by the nursing staff
- Check mobility-limiting medication
- Prescribe physiotherapy if indicated
- Inform the research team about discharge or transfer (task of the nursing and medical staff)

Possible questions asked by the patients

Technical issues

1) iPad (internet connection, open the website with the exercises)
2) Accelerometer

⇒ **Ask the research team for support if needed!**

Questions on mobility (set goals, which exercises to do, etc.):

⇒ As a healthcare professional, you are responsible to answer those questions (with help of physiotherapists if needed)

Tips for putting mobility into practice – 1

Inform and encourage the patients

- ✓ **Why, when, where, how, how often** they should / are allowed to move + how to handle tubes/catheters/drains/oxygen/perfusion.
- ✓ **Main purpose** of mobility during an acute hospitalization = maintain autonomy.
- ✓ Reduce **false beliefs** (for example: «At hospital, one should stay in bed»).

Objectives

- ✓ Set **concrete objectives daily** and discuss results / difficulties with the patients
- ✓ Incorporate mobility in **activities of daily life**: Personal hygiene, eating at the table, going to the bathroom, wear one’s own cloths.
- ✓ Let patients sit during visits / rounds (instead of letting them lay in bed).

Tips for putting mobility into practice – 2

Provide **walking aids**.

Accompaniment

- ✓ Patients do not want to disturb: Encourage them to ask for support if needed!
- ✓ Family / friends can be an important support to accompany the patients

Place in the hallways

- ✓ Avoid to let them carts.
- ✓ Inform patients that they do not disturb in the hallways.

Tips for putting mobility into practice – 3

Planning the day

- ✓ Explain to the patients that they do not miss anything if they leave the room to move.
- ✓ Inform the patients as much as possible about the daily planning of the rounds and exams.
- ✓ Ask the patients to inform you when they leave the unit.

Mobility is a priority

- ✓ Discuss mobility during nursing handing over and during rounds.
- ✓ **Interdisciplinary work** with physiotherapists, physicians and **family/friends.**
- ✓ **Checklist** + **documentation** daily! This help for **monitoring.**
- ✓ Plan as much as possible when and how you will care for your patients' mobility during your work day.

Take home messages: Mobility

- **Inform and encourage patients**
- **Setting goals with patients**
- **Interdisciplinary work**
- **Mobility = priority for maintaining autonomy**

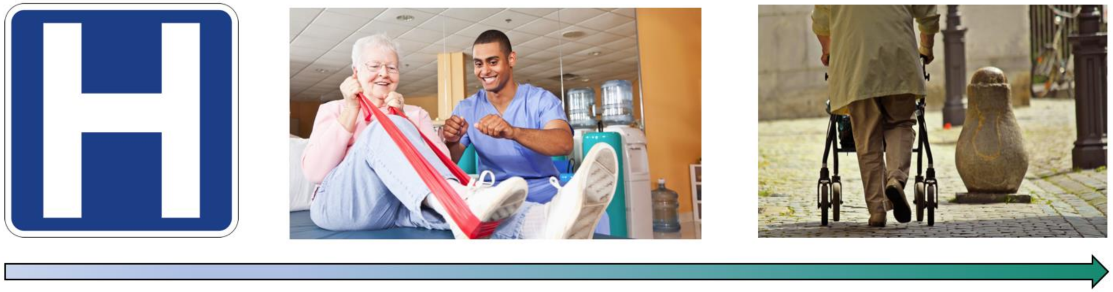

Take home messages: Your role in INTOMOB

- **Inform and encourage patients**
- **Set goals with patients, using the diary and booklets**
- **Assess barriers and facilitators using the checklist**

(Again,) please **DO NOT discuss the intervention with your colleagues in other units** to avoid skewing the results of the study.

Your role is essential to stimulate patients to move!

We really appreciate your work and support. **Thank you so much for your commitment**!

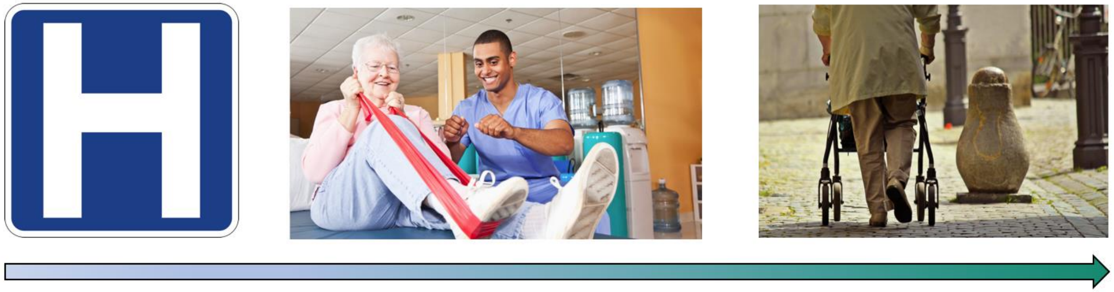

References

- Mani H, Möri C, Mattmann M, Liechti F, Inauen J, Aujesky D, Donzé J, Aubert CE. Barriers and facilitators to mobility of patients hospitalized on an acute medical ward: a systematic review. *Age and Ageing.* 2022.

- Brown CJ, Redden DT, Flood KL, Allman RM. The underrecognized epidemic of low mobility during hospitalization of older adults. J Am Geriatr Soc 2009.

- Brown CJ, Friedkin RJ, Inouye SK. Prevalence and outcomes of low mobility in hospitalized older patients. J Am Geriatr Soc 2004.

- Kalisch BJ, Lee S, Dabney BW. Outcomes of inpatient mobilization: a literature review. J Clin Nurs 2014.

- Van Dijk-Huisman HC, Raeven-Eijkenboom PH, Magdelijns FJH, Sieben JM, de Bie RA, Lenssen AF. Barriers and enablers to physical activity behaviour in older adults during hospital stay: a qualitative study guided by the theoretical domains framesork. BMC Geriatr. 2022.

- Beelen SJG, van Dijk-Huisman HC, de Bie RA, Veenhof C, Engelbert R, van der Schaaf M, Lenssen AF. Barriers and enablers to physical activity in patients during hospital stay: a scoping review. Syst Rev. 2021.

- The MOVE Program - Promoting mobilization during a hospitalization

Creation of e-learning

PD Dr. med. Carole E. Aubert, MD, MSc

Med. pract. Blandine Mooser, medical doctor

Additional information

Contact person

PD Dr. med. Carole E. Aubert, caroleelodie.aubert@insel.ch
