# Supplementary material for: Impact of an INtervention to increase MOBility in older hospitalized medical patients (INTOMOB): Study protocol for a cluster randomized controlled trial
Source: BMC Geriatr. 2023 Oct 31;23:705. doi: 10.1186/s12877-023-04285-3 (PMC10617203; doi:10.1186/s12877-023-04285-3)
Supplement: Supplementary file 5 — Additional file 5: Supplement 5. Slides for HCP oral presentation. [file 12877_2023_4285_MOESM5_ESM.pdf]

INSELGRUPPE

INTOMOB

INTOMOB: An intervention to improve mobility of patients hospitalized in general internal medicine

Presentation for healthcare professionals

Dr. Carole E. Aubert, MD, MSc
Attending physician
Bern University Hospital, Bern

Schweizerischer Nationalfonds

Agenda

- ✓ Context and goal of the project
- ✓ Patient recruitment
- ✓ Intervention (patients, medical unit, healthcare professionals)
- ✓ Role of healthcare professionals (physicians and nursing staff)

Context and goal

Low mobility during an acute hospitalization

- Frequent
- Negative consequences
- Barriers to mobility: healthcare professionals, patients, environment, resources

Goal of the INTOMOB study

To improve mobility of older patients hospitalized on a medical ward through an intervention targeting patients, healthcare professionals and the hospital environment, with the goal to maintain prior autonomy.

Study design

- Randomized study on all general internal medicine wards.
- INTOMOB intervention compared with standard of care.
- 6-month follow-up.
- Primary outcome: Life-Space Assessment (assess how much a person moves from their sleeping room up to outside the city).

Randomization

Randomization means that it is randomly decided whether a unit receives the intervention or the control procedures.

<u>Medical units</u> (and not patients) are randomized.

- Healthcare professionals working on a «control» ward and patients admitted there and who agree to participate to the study
  → «control» group.
- Healthcare professionals working on an «intervention» ward and patients admitted there and who agree to participate to the study
  → «intervention» group.
- Your medical unit is in the «intervention» group => please do not speak about the study with colleagues from other units.

Patient inclusion / exclusion criteria

Inclusion criteria

- 60 years or older
- Planned length of stay ≥ 3 days
- Mobile during the last 2 weeks before admission
- Able to speak/understand French or German

Exclusion criteria

- Wheelchair
- Bedrest
- Severe psychiatric disease
- Delirium
- Severe visual disorder
- Dementia (except if proxy's support)
- Living in nursing home

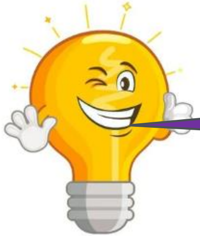

Patients are recuited by the research team!
(**NOT** by healthcare professionals
working on the medical unit)

INSELGRUPPE

INTOMOB

Intervention

3 components:

1) Patients

2) Medical unit (environment)

3) Healthcare professionals: nurses, nursing assistants, medical residents

INSELGRUPPE

Patient intervention

All documents are online and in the offices, have a look at them!

1) Information booklet
2) Diary
3) Exercise booklet (lying, sitting, standing)
4) iPad 10.2'' with videos of the exercises

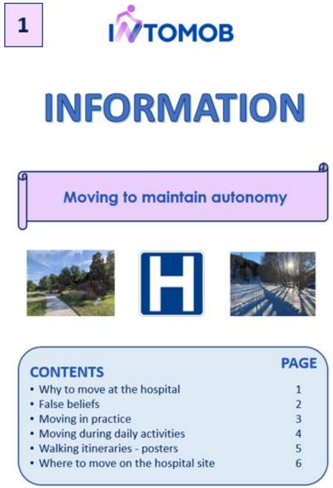

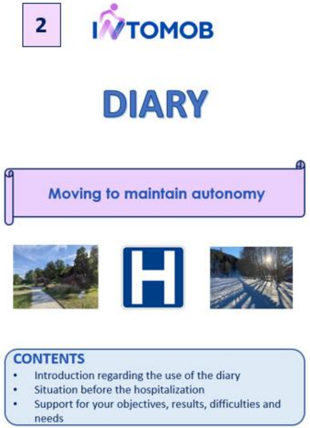

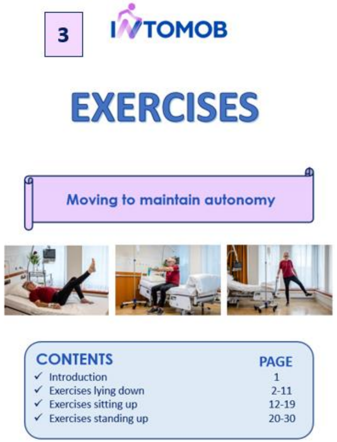

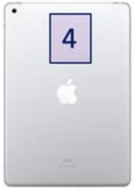

Patient intervention

Some exercises can be done with a TheraBand. You can give max. 2 TheraBands to each patient with a request and without physiotherapy (box with TheraBands in nurse office).

TheraBand's level of strength according to exercise type and patient strength (can be assessed by nursing staff):

Exercice	Frail patient	Strong patient
With arms	Level 1	Level 3
With legs	Level 3	Level 4

=> MAXIMUM 2 TheraBands for each patient!

Patient intervention

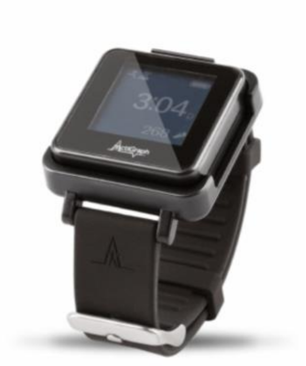

ActiGraph wrist-worn accelerometer

- To be worn 24/7
- Waterproof
- To remove for MRI only
- Do not change wearing side (usually non-dominant side)
- Can be worn over clothes / bandage / compression bandage

Unit intervention

Posters

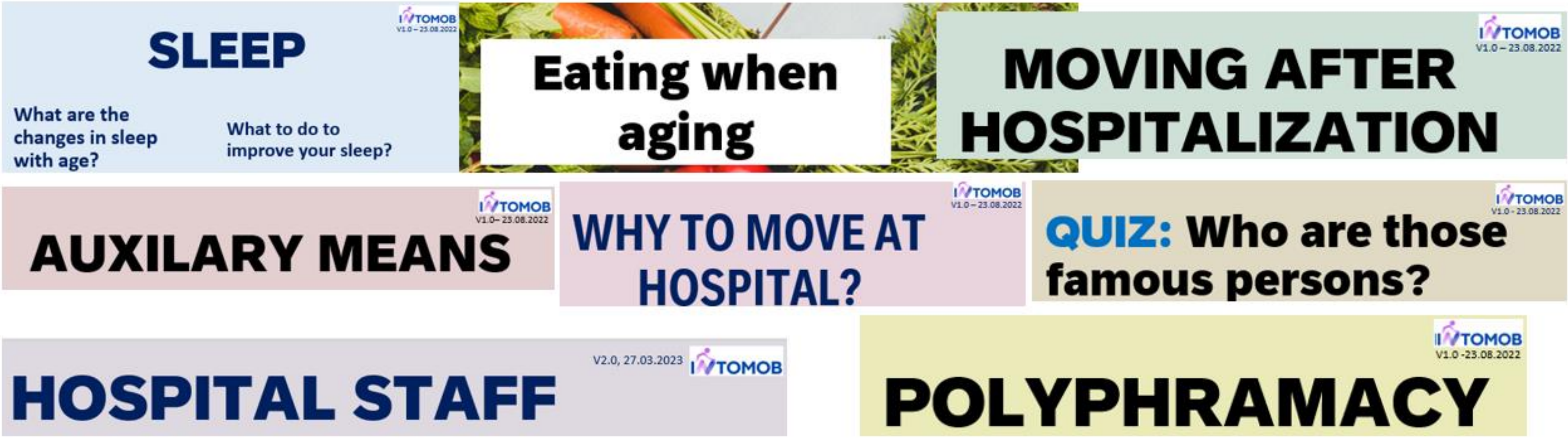

Unit intervention

Walking itineraries with pictures and „fun facts“

- Famous people
- Flowers
- Animals
- Landscapes

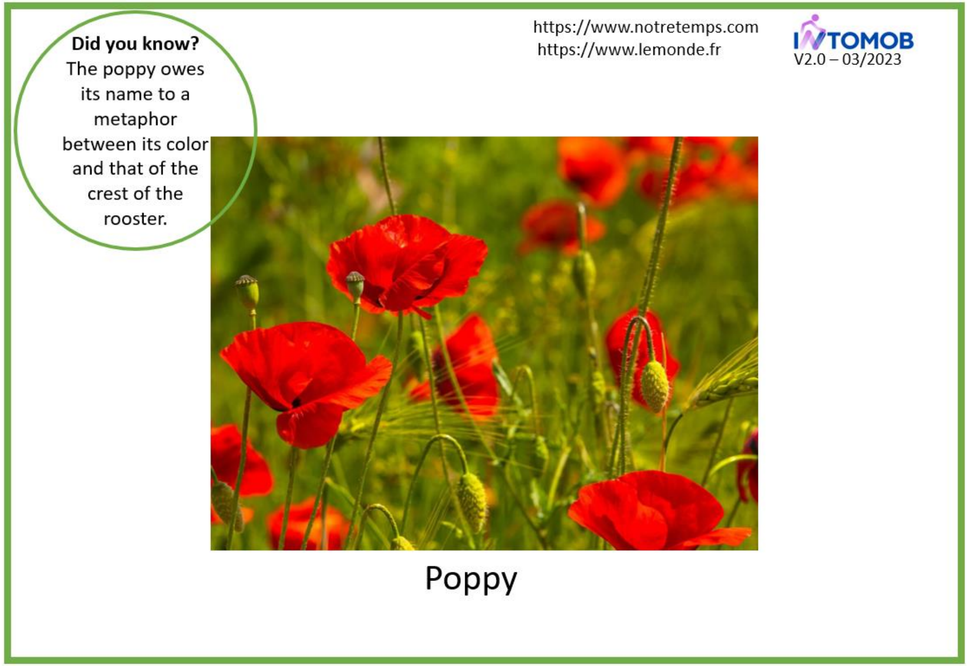

Healthcare professional intervention

Nursing staff = nurses and nursing assistants working on the unit.
Medical staff = medical residents working on the unit.

E-learning (15 minutes): **Mandatory** for your, to be done from now on, or, for new collaborators, within the first week after joining the participating unit.

Your head of nursing will regularly remind you about the study!

Healthcare professional intervention

Checklist

- ✓ Pocket card
- ✓ Hung up in units, offices and on visit cards
- ✓ Available on the units
- ✓ **Nursing staff must verify the checklist daily during the round**, and if needed speak about it with the physician

MOBILITY CHECKLIST

PLEASE CHECK DAILY!

1) Catheter, tube, drainage, perfusion to remove?

2) Mobility-limiting medication to stop?

=> anticholinergics, antidepressives, hypnotics, opioids, antipsychotics, antiepileptics, antihypertensives

3) Mobility aid needed/available?

4) Physiotherapy needed/prescribed?

5) Discuss mobility objectives and behaviors

6) Document mobility!

INSELGRUPPE

Research staff's phone number and e-mail

Discharge / transfer of a patient participating to the study

Discharge

1) Healthcare professionals responsible for the patient inform the research team as soon as discharge date is known and the responsible nurse personal brings the iPad and accelerometer to the unit office at discharge (research team comes to pick them up in the unit). The patient keeps the paper documents.

Change of unit on the department of general internal medicine:

1) Healthcare professionals responsible for the patient inform the research team.
2) The patient keeps iPad, paper documents, accelerometer.

Transfer to ICU or to another department than general internal medicine

1) Healthcare professionals responsible for the patient inform the research team and the responsible nurse personal brings the iPad and accelerometer to the unit office at discharge (research team comes to pick them up in the unit).

Discharge / transfer of a patient participating to the study

Discharge

1) Healthca[illegible]am as soon as di[illegible]d and acc[illegible] up in the [illegible]

Change[illegible]

1) Hea[illegible]
2) The[illegible]

Transfer to[illegible]

1) Healthcare professionals responsible for the patient inform the research team and the responsible nurse personal brings the iPad and accelerometer to the unit office at discharge (research team comes to pick them up in the unit).

INFORM THE RESEARCH TEAM!
phone number
email

Tasks of the research team

- Check inclusion/exclusion criteria (medical file + questions to the healthcare professionals of the ward)
- Explain the study to the patients
- Sign the informed consent
- Give and explain the material to the patients
- Set up and install the accelerometer
- Help for technical issues
- Admission and discharge examinations for the study
- Pick up the iPad and accelerometer in the nursing office after discharge

Tasks of the nursing staff

- Set objectives and discuss goals and difficulties with patients
- Motivate and advise the patients
- Give a TheraBand if wished and if no physiotherapy prescribed
- Verify the checklist 1x/day => discuss it if needed with the physician
- Document patient mobility
- Help with the use of the iPad or put on/off the accelerometer (set up done by the research team)
- Inform the research team about discharge or transfer (rask of the nursing and medical staff)
- Bring the iPad & accelerometer in the box in the nursing office at discharge (pick up is done by the research team)

OBJECTIVES

DATE ______________________

Here you can find examples of goals. You don't have to achieve them all!
Choose a few goals that suit you.
You can add easier or more difficult objectives!

Objectives	Frequency?	Help?*	Reached?
Doing exercises in bed.			
Sitting up on the side of the bed			
Moving from the bed to the chair			
Moving around the room			
Moving around the corridor			
Going up/down the stairs			
Walking around outside of the unit			
Going to the cafeteria			
Going to the bathroom			
Showering			
Dressing with my own clothes			
Eating at the table			
Doing exercises sitting up			
Doing exercises standing up			

MOBILITY CHECKLIST

THANKS TO CHECK DAILY!

1) **Catheter, tube, drainage, perfusion to remove?**

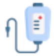

2) **Mobility-limiting medication to stop?**

=> anticholinergics, antidepressives, hypnotics, opioids, antipsychotics, antiepileptics, antihypertensives

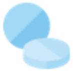

3) **Mobility aid needed/available?**

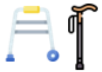

4) **Physiotherapy needed/prescribed?**

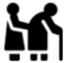

5) **Discuss mobility objectives and behaviors**

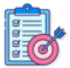

6) **Document mobility!**

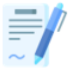

Tasks of the medical residents

- Discuss goals and difficulties with the patients, motivate and advise them
- Discuss checklist items if touched on by the nursing staff
- Check mobility-limiting medication
- Prescribe physiotherapy if indicated
- Inform the research team about discharge or transfer (task of the nursing and medical staff)

Possible questions asked by the patients

Technical issues

1) iPad (internet connection, open the website with the exercises)
2) Accelerometer

$\Rightarrow$ **Ask the research team for support if needed!**

Possible questions asked by the patients

Questions on mobility (set goals, which exercises to do, etc.):

⇒ As healthcare professional, you are responsible to answer those questions (with help of physiotherapists if needed)

Access to exercises:

QR CODE PROVIDED

Access to study documents:

QR CODE PROVIDED

Let’s go, let’s start with the e-learning!

Access to exercises:

QR CODE PROVIDED

Access to study documents:

QR CODE PROVIDED

Thanks for your commitment and your collaboration!

Questions: caroleelodie.aubert@insel.ch
