# Supplementary material for: Impact of an INtervention to increase MOBility in older hospitalized medical patients (INTOMOB): Study protocol for a cluster randomized controlled trial
Source: BMC Geriatr. 2023 Oct 31;23:705. doi: 10.1186/s12877-023-04285-3 (PMC10617203; doi:10.1186/s12877-023-04285-3)
Supplement: Supplementary file 6 — Additional file 6: Supplement 6. Checklist. [file 12877_2023_4285_MOESM6_ESM.pdf]

MOBILITY CHECKLIST

PLEASE CHECK DAILY!

1) Catheter, tube, drainage, perfusion to remove?

2) Mobility-limiting medication to stop?

=> anticholinergics, antidepressives, hypnotics, opioids, antipsychotics, antiepileptics, antihypertensives

3) Mobility aid needed/available?

4) Physiotherapy needed/prescribed?

5) Discuss mobility objectives and behaviors

6) Document mobility!

INTOMOB

INTOMOB study– version 2.0 – 06.04.2023

flaticon by Freepik, Icon Pond, smashingstocks, iconnut, Backwoods, Dave Gandy, Flat Icons
