# Supplementary material for: Impact of an INtervention to increase MOBility in older hospitalized medical patients (INTOMOB): Study protocol for a cluster randomized controlled trial
Source: BMC Geriatr. 2023 Oct 31;23:705. doi: 10.1186/s12877-023-04285-3 (PMC10617203; doi:10.1186/s12877-023-04285-3)
Supplement: Supplementary file 7 — Additional file 7: Supplement 7. a. Posters. b. Landscapes - environment intervention. c. Flowers - environment intervention. d. Animals - environment intervention. e. - Famous people - environment intervention. [file 12877_2023_4285_MOESM7_ESM.zip › 12877_2023_4285_MOESM7_ESM/Supplement 7a - Posters.pdf]

V1.0 – 23.08.2022

SLEEP

What are the changes in sleep with age?

- Less restful and less efficient sleep
- Waking up earlier
- More shallow sleep
- More frequent awakenings (felt or not) during the night

Sleeping pills?

- To avoid!
- Do not improve sleep in the long term
- Numerous side effects: confusion, falls, dizziness, ...
- Induce dependence

Sleep at hospital

- Can be difficult due to "cohabitation”
- Use earplugs and an eye mask
- During the day: avoid staying in bed: get dressed, move around, expose yourself to daylight

What to do to improve your sleep?

- Get out and about during the day
- Schedule: always go to bed and get up at the same time
- Avoid long naps during the day
- Avoid heavy meals in the evening; eat at least 2 hours before bedtime
- Avoid excitants (coffee, tea) at the end of the day
- Avoid exposure to screens 2 hours before bedtime
- Create favorable conditions for sleep: dark, quiet, well ventilated room, temperature at 18-20°C

Sources: Special file "Sommeil et personnes âgées" – Société française de gériatrie et de gérontologie- SFGG, www.flaticon.com (Vectors Market, srip, Freepik, Smashicons)

Eating when aging

INTOMOB
V1.0 – 23.08.2022

- Food pyramid: still relevant!
- Eat more protein (1 - 1.2 g per kg of body weight per day) and vary the sources of protein:
  - 1 **yoghurt** ≈ 6 g proteins
  - 1 «**séré/quark**» ≈ 10 g proteins
  - 30 g **cheese** ≈ 9 g proteins
  - 100 g lentils (raw) = 9 g proteins
  - 100 g de **meat or fish** ≈ 20 g proteins
  - 1 **egg** = 6 g proteins

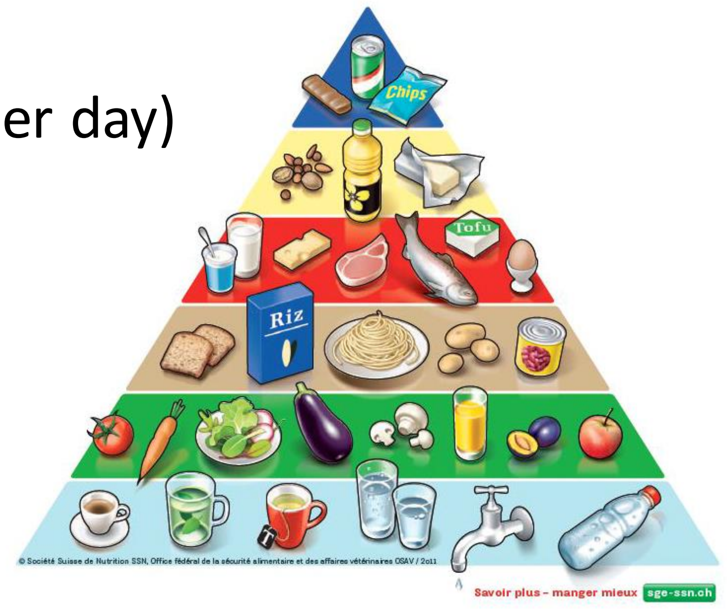

- Eat 3 servings of dairy products a day for the bones! The risk of **malnutrition** is often greater than the risk of being overweight: avoid dieting!
- Be careful with **alcohol**: increased sensitivity and risk of interaction with medications.

Why is that important?

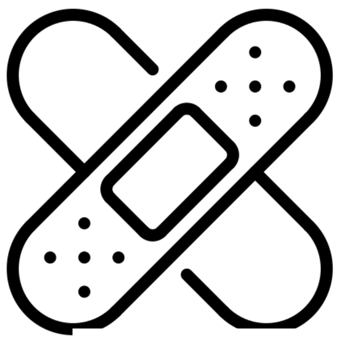
Wound healing

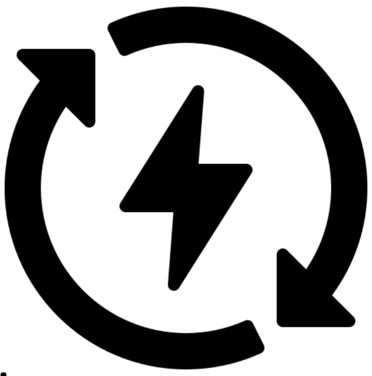
Energy

Sugar
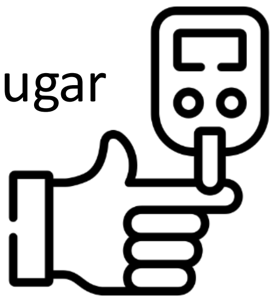

Muscles
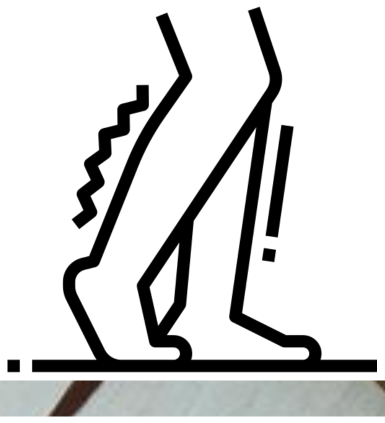

What if…?

You are lacking appetite?
Split your meals into 5-6 servings per day
Cook/eat what you feel like
Share meals with others

You have dental pain or chewing problems?
Discuss with the health care team
Favor soft foods
Insist on dental hygiene

Cooking has become too hard?
Go to restaurants if possible
Have meals delivered to your home
Eat at a friend's / family member's house

Sources: www.sge-ssn.ch ; Icons produced by alkhalifi design, nawicon, Freepik and Nhor Phai for www.flaticon.com

Some parts are specific to Switzerland and should be adapted for other countries!

V2.0, 27.03.2023

HOSPITAL STAFF

Physician

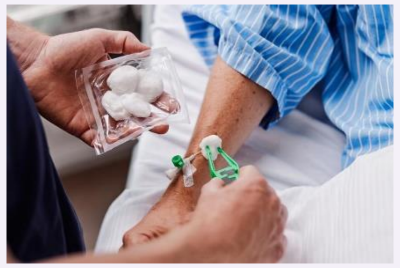

Studies: 6 years (University)

Duties:

- Makes diagnoses.
- Prescribes exams, medication and physiotherapy.
- Evaluates patient mobility capacities.
- ➢ **Medical resident:** supervised by chief resident.
- ➢ **Chief resident:** supervision and teaching duties.

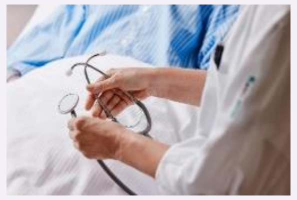

Registered nurse

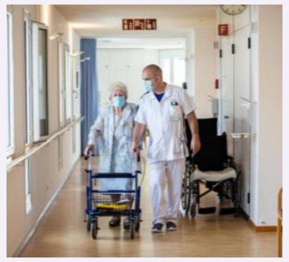

Studies: 3 years (specialized high school)

Duties:

- Administration of medication; planning, conducting, delegating and monitoring nursing care.
- Helps patients to move and accompanies them.

Occupational therapist

Studies: 3 years (specialized high school)

Duties:

- Helps patients regain or maintain their autonomy in daily, social and professional activities, etc.
- Proposes auxiliary means and adapts patient environment.

Dietician

Studies: 3 years (specialized high school)

Duties:

- Assessment of nutritional status and adaptation of diet (e.g. supplements, tube feeding).

Advanced nursing assistant

Studies: 3 years (certificate)

Duties:

- Performs nursing tasks (care, feeding, administration of treatments) under the supervision of registered nurses. Helps patients to move and accompanies them.

Physiotherapist

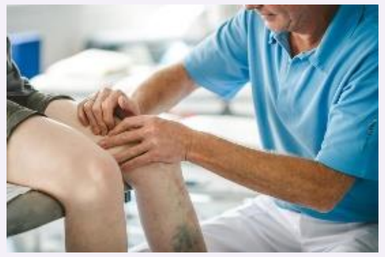

Studies: 3 years (specialized high school)

Duties:

- Specialized evaluation of patient mobility capacities.
- Prevents, supports and treats mobility problems.
- Provides patients with an individualized mobility program.

Nursing assistant

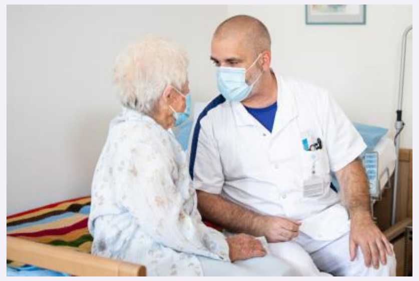

Studies: 2 years (certificate)

Duties:

- Provides body care & wellness to patients under the supervision of registered nurses.
- Helps patients to move and accompanies them.

Sources photos: insel.frontify.com
Text based on www.orientation.ch

WHY MOVE AT THE HOSPITAL?

Moving helps to:

Maintain muscles

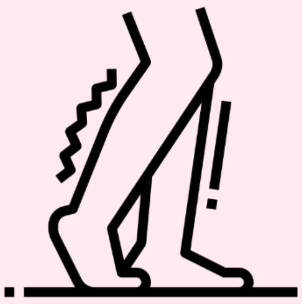

Maintain autonomy

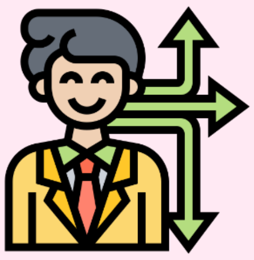

Avoid infections

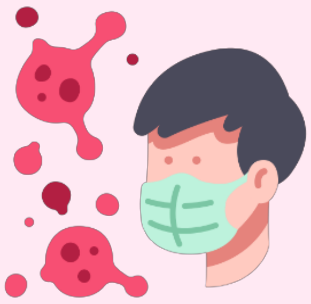

Reduce falls

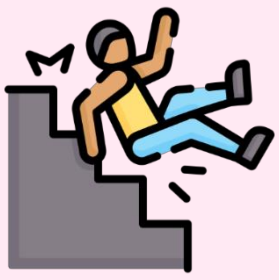

Reduce pain

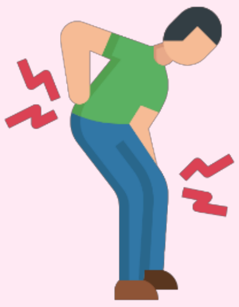

Avoid thrombosis / embolism

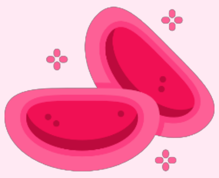

Feel well mentally

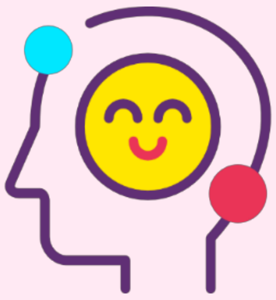

Improve sleep

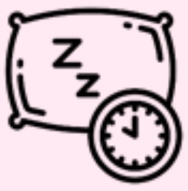

Improve appetite and avoid constipation

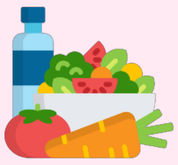

How to move as much as possible at hospital?

- Eating at the table
- Sitting down when having visits with caregivers or relatives
- Putting on your own clothes instead of a hospital gown
- Washing at the sink or in the shower (not in bed)
- Going to the bathroom (avoid bottle or vase)
- Walk around at least 3 times a day:
  - If necessary with assistance: walking aid (cane, ...), relative, caregiver
  - Even with tubes/catheters: caregivers can explain how to handle them
  - Inform the staff when leaving the unit

Sources: Icons produced by DariusDan, surang, max.icons, Eucalyp, Konkapp, Smashicons and FreePik on www.flaticon.com

INTOMOB
V1.0 – 23.08.2022

MOVING AFTER HOSPITALIZATION

Why move?

To maintain autonomy

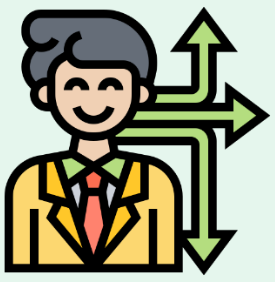

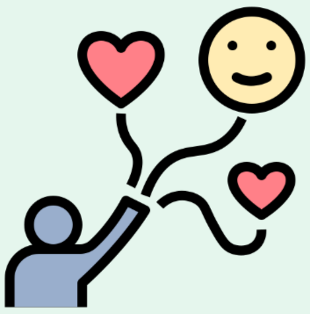

To have fun

To feel well physically and mentally

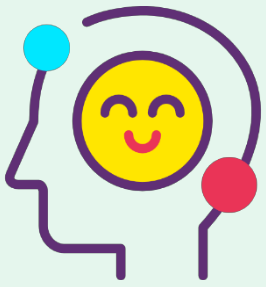

What to do in practice? Every movement is valuable!

Activities of daily living

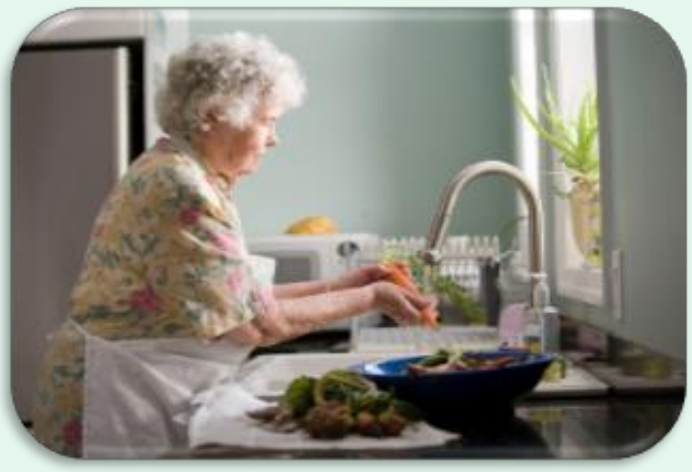

As independently as possible: eating, dressing, grooming, cooking, shopping,...

An activity that is fun

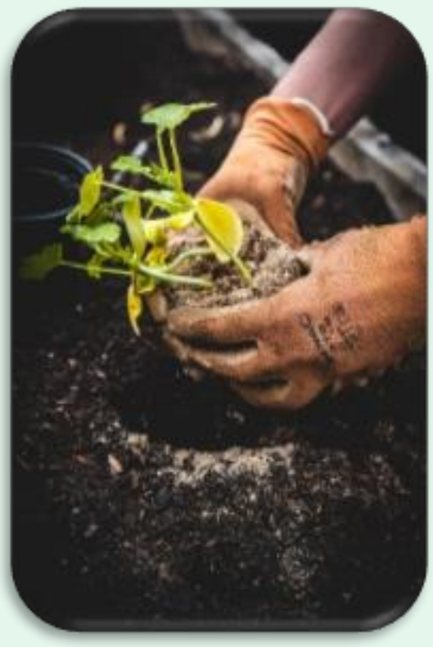

Aquafit, gardening, pétanque, walks, ...

If possible: exercise

Talk to your doctor before you start!

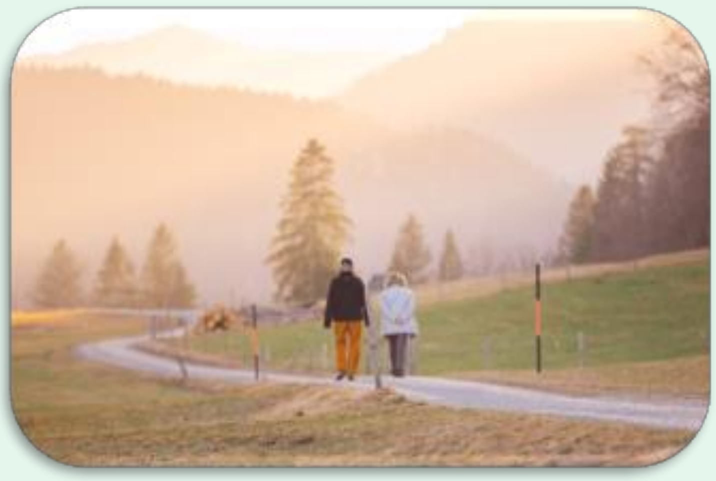

Do you need help?

Talk to your health care team!

- Home care
- Physical / occupational therapy
- Walking aids (for example: walker)
- Ambulatory rehabilitation

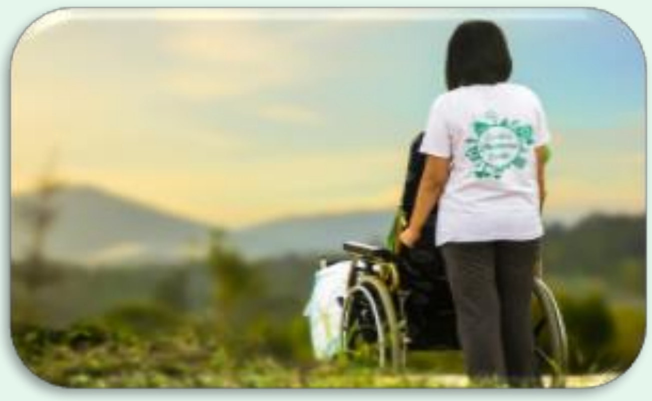

Sources: Photos by www.unsplash.com, icons by Work Life Balance, Eucalyp, Freepik for www.flaticon.com

INTOMOB
V1.0– 23.08.2022

AUXILARY MEANS

What are auxillary means?

«Simple, adequate and economical means designed to facilitate daily life" and be as independent as possible :

- ✓ Canes, crutches, walkers, wheelchairs
- ✓ Orthopedic shoes, hearing aids, protheses, orthoses, glasses, contact lenses
- ✓ Stair lifts, sanitary facilities modifications, motor vehicles

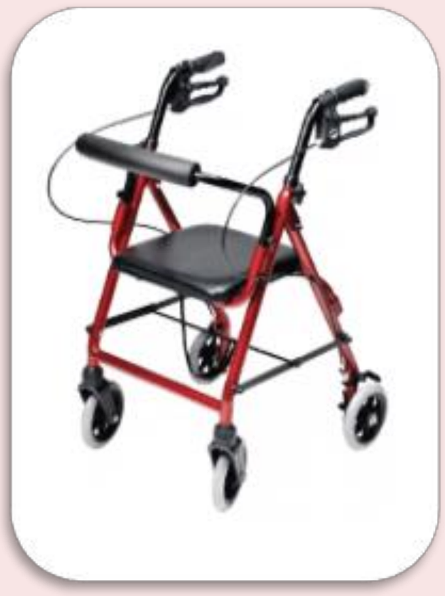

An appropriate way to help you maintain your independence!
Healthcare professionals are there to advise you!

Who pays for the auxilary means?

- Disability Insurance (DI): the aid must be included in the List of aids and appliances (LiMA) established by the Federal Council (available at www.bag.admin.ch)
- If the DI does not cover the cost of the aid, there are other possibilities for covering the cost:
  - Supplementary benefits
  - AVS
  - Pro Infirmis

Some parts are specific to Switzerland and should be adapted for other countries!

How to apply for payment?

- Fill out the form "001.002 - Auxiliary means" at the DI office of your canton of residence or at www.avs-ai.ch
- The social service will be pleased to help you.

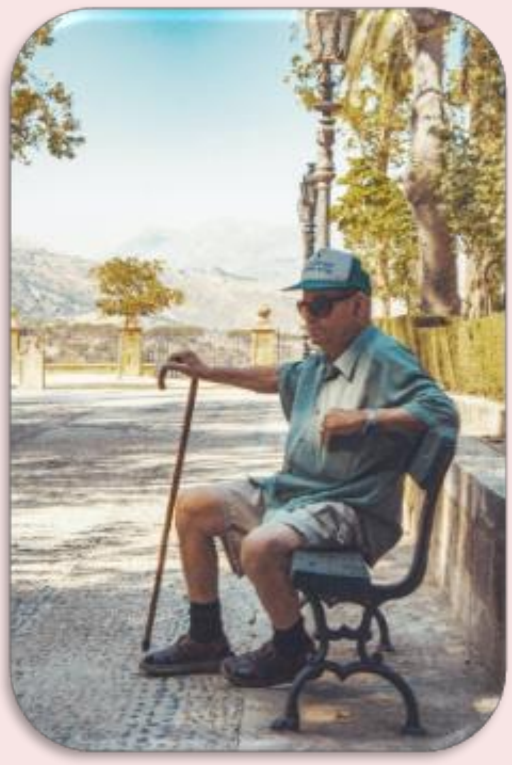

Where to find auxillary means?

- Orthoconcept (Fribourg)
- Orthoteam (Bern)
- SAHB Hilfsmittelberatung (Bern)
- Hilfsmittelstelle HMS AG (Bern)

For more information:

- www.avs-ai.ch; www.bag.admin.ch

Sources: www.ahg-iv.ch; www.bag.admin.ch; www.unsplash.com; https://medicdepot.ca/produit

INTOMOB
V1.0 -23.08.2022

POLYPHRAMACY

What is polypharmacy?

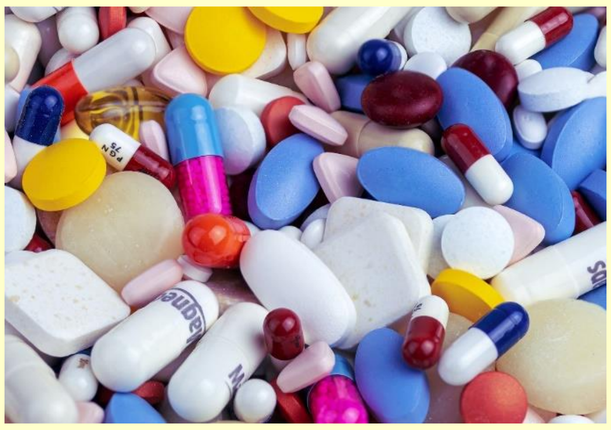

- Taking 5 or more medications per day

Who is affected in Switzerland?

- Over 40% of people aged >65 years

Why is polypharmacy so frequent?

- More medications available
- Chronic diseases more frequent (hypertension, diabetes, heart failure, etc.)
- Prevention of certain diseases with medication

What questions to ask healthcare professionals?

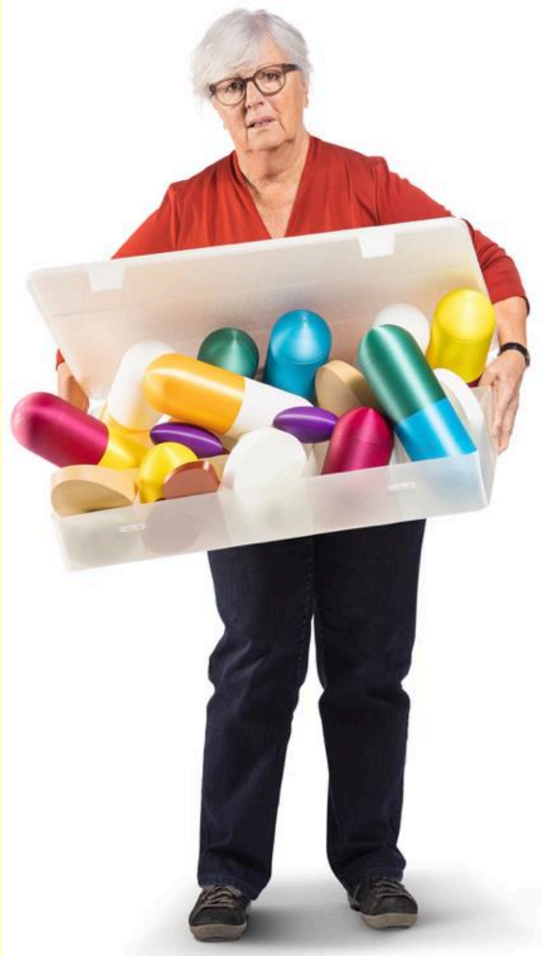

- **CHANGES:** Have any medications been added, removed or changed and why?
- **CONTINUE:** Which medication should I continue to take and why?
- **PROPER USE:** How should I take my medication and for how long?
- **MONITOR:** How will I know if my medication is working and what side effects should I watch out for?
- **FOLLOW-UP:** Will I need check-ups and if so, what and when?

Which medication to avoid in older persons?

- Anti-inflammatory medication (ex: ibuprofen)
- Sleeping pills (ex: zolpidem, oxazepam, lorazepam)

How to avoid forgetting your medication or making mistakes with intake?

- Pillbox, distribution through pharmacy, home care

Sources: CHUMontreal.qc.ca, Revue Médicale Suisse (Mazzocato, C., Monod, S., Polymédication et personne âgée : ni trop ni trop peu !, 2013); Blozik et al. Drugs & Aging 2013; www.patientsafetyinstitute.ca ; www.unsplash.com, www.smartermedicine.ch

V1.0 - 23.08.2022

QUIZ: Who are those famous persons?

1.

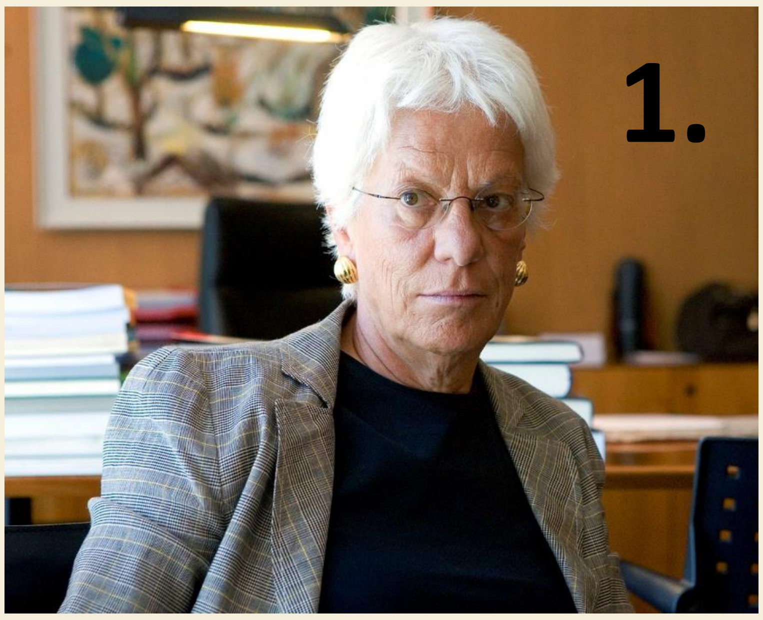

2.

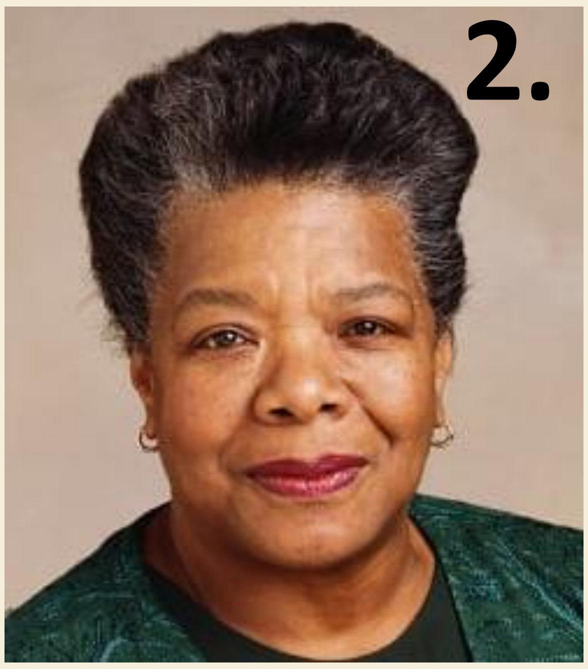

3.

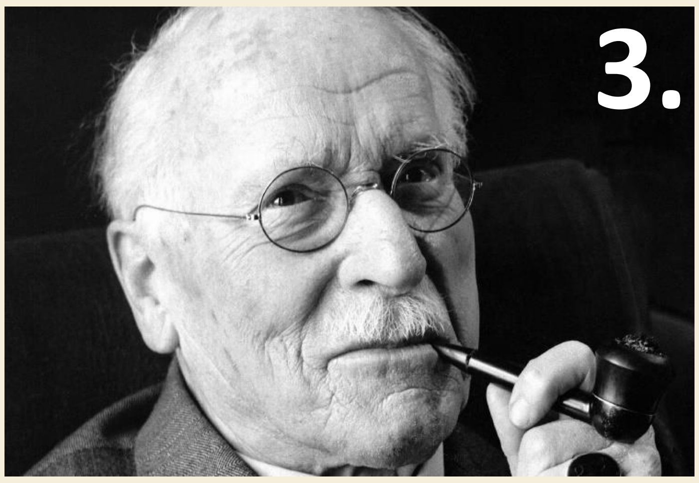

4.

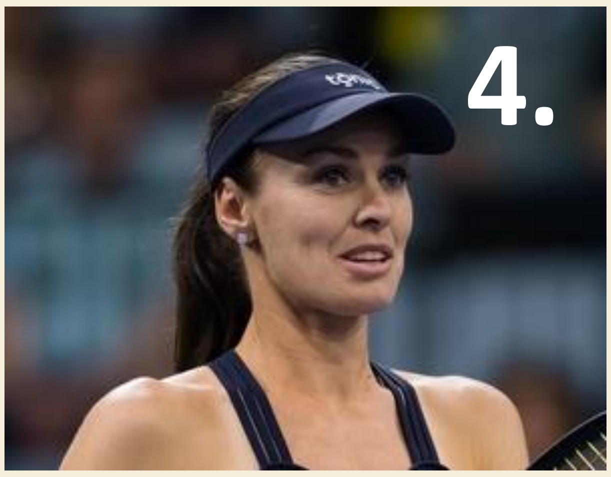

5.

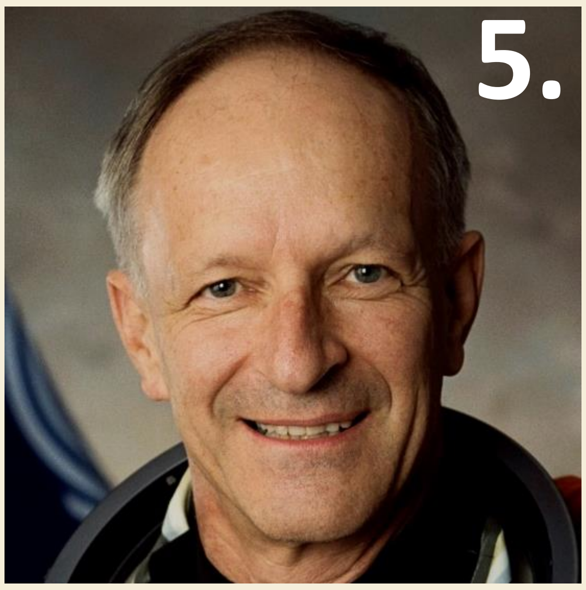

6.

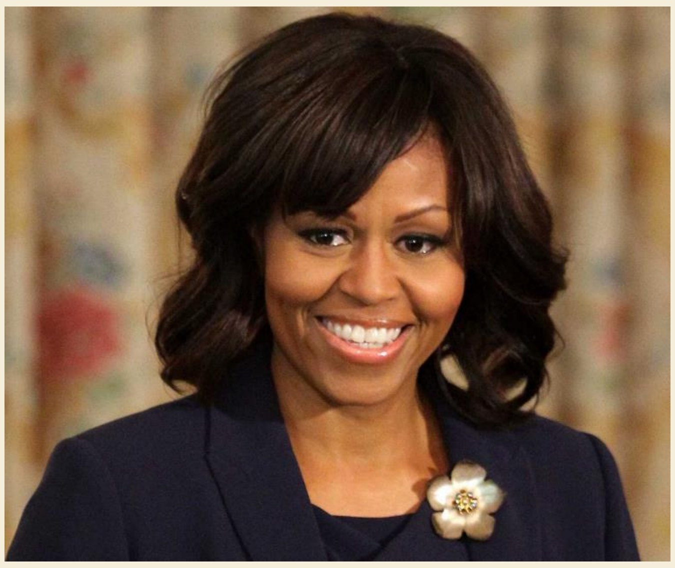

7.

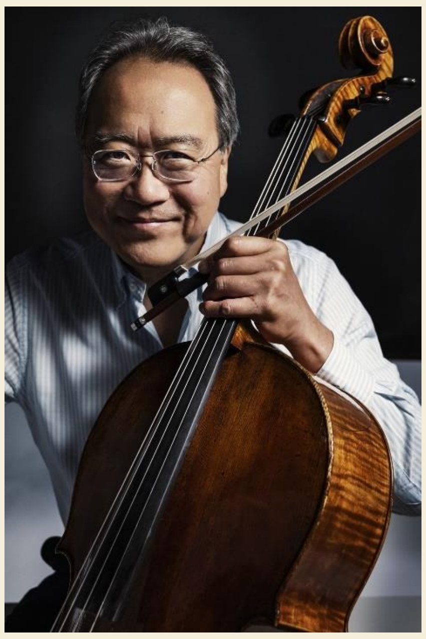

8.

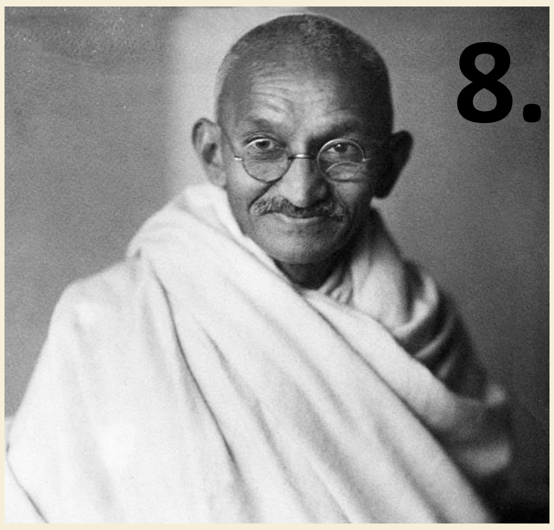

9.

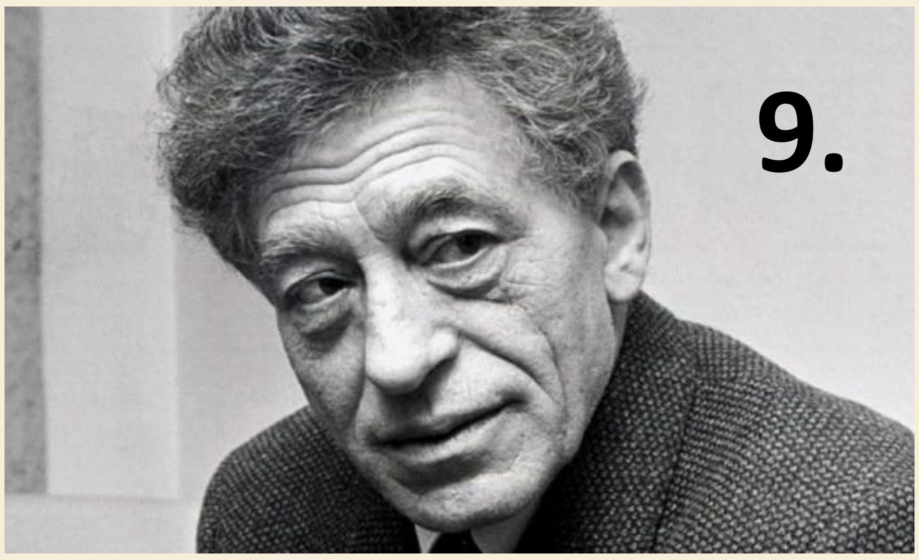

Choice:

a) Maya Angelou, author
b) Alberto Giacometti, sculptor
c) Carla del Ponte, jurist et diplomat
d) Michelle Obama, lawyer and author
e) Claude Nicollier, astronaut
f) Yo-Yo Ma, cellist
g) Carl Jung, psychiatrist
h) Mohandas Gandhi, political and spiritual leader
i) Martina Hingis, tennis player

Responses and sources are on another poster!

INTOMOB
V1.0 - 23.08.2022

ANSWERS: Who are those famous persons?

Carla del Ponte,
jurist and diplomat

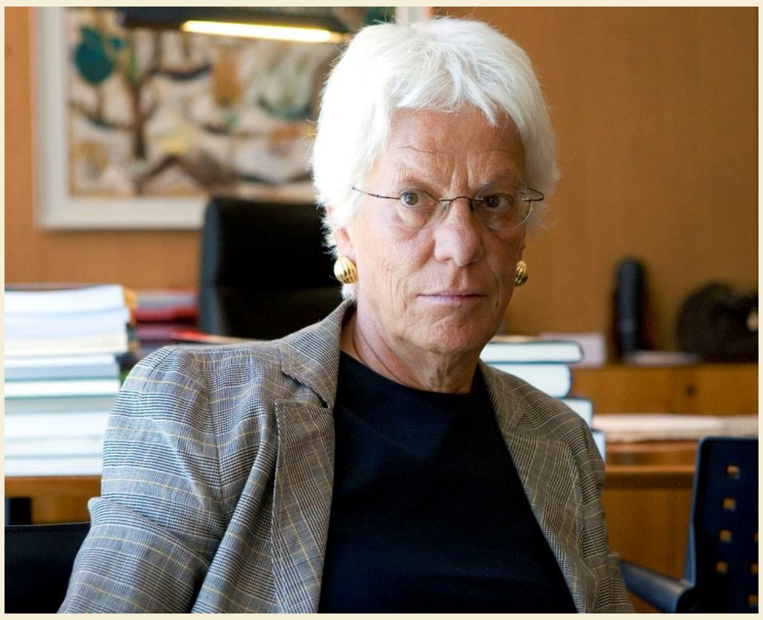

www.blick.ch

Maya Angelou,
author

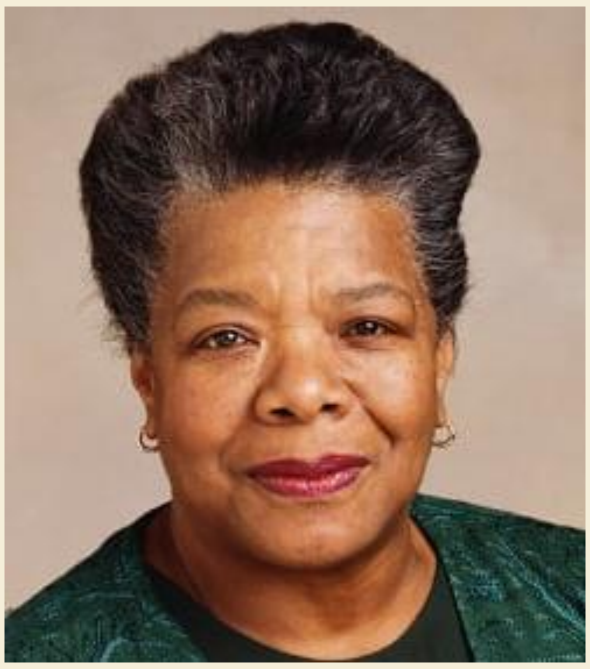

Biography.com

Carl Jung, psychiatrist

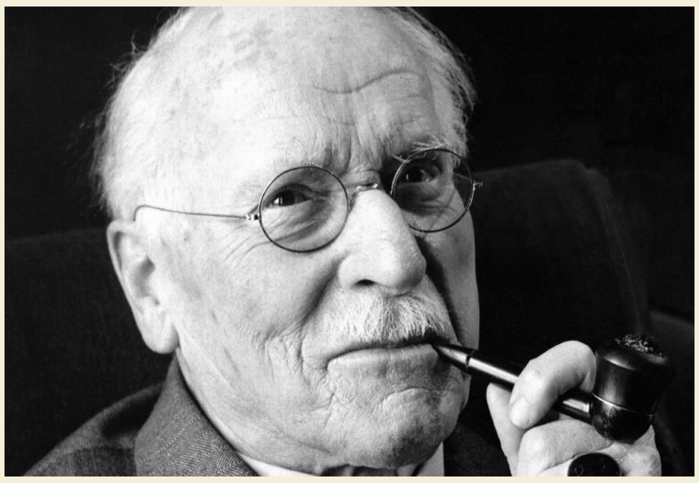

www1.wdr.de

Martina Hingis,
tennis player

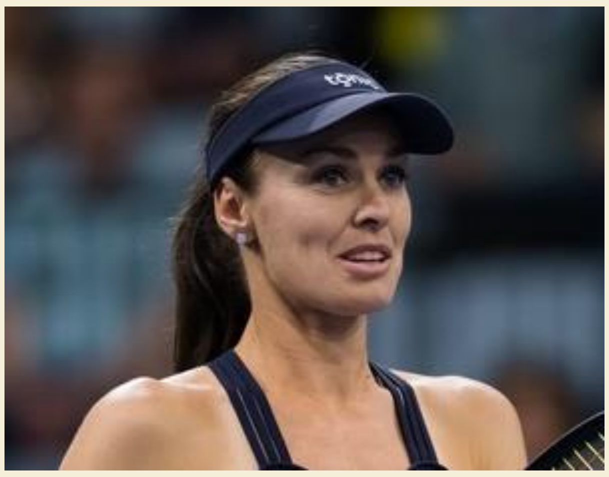

Shutterstock.com

Claude Nicollier,
astronaut

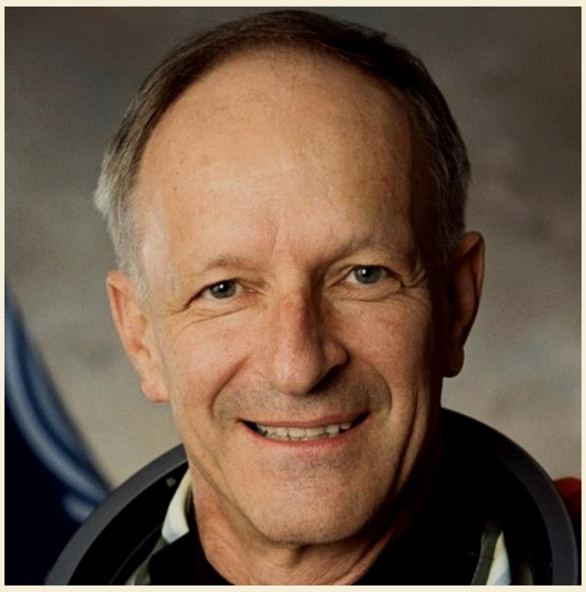

https://upload.wikimedia.org

Michelle Obama,
lawyer and author

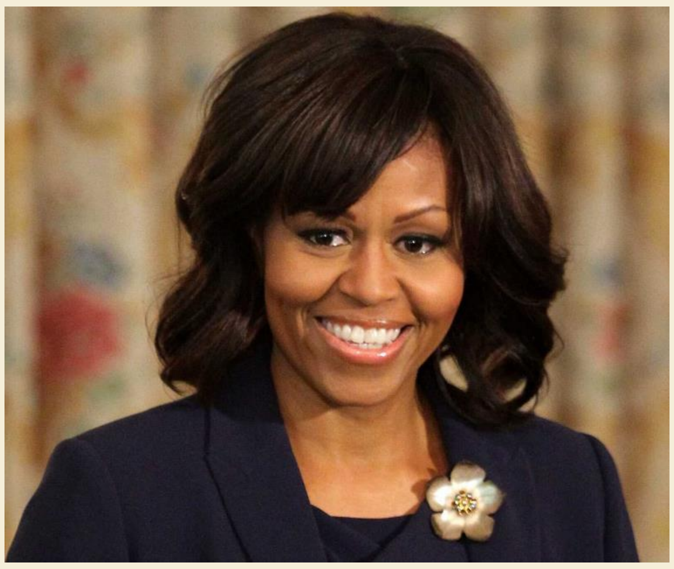

Gala.fr

Yo-Yo Ma,
cellist

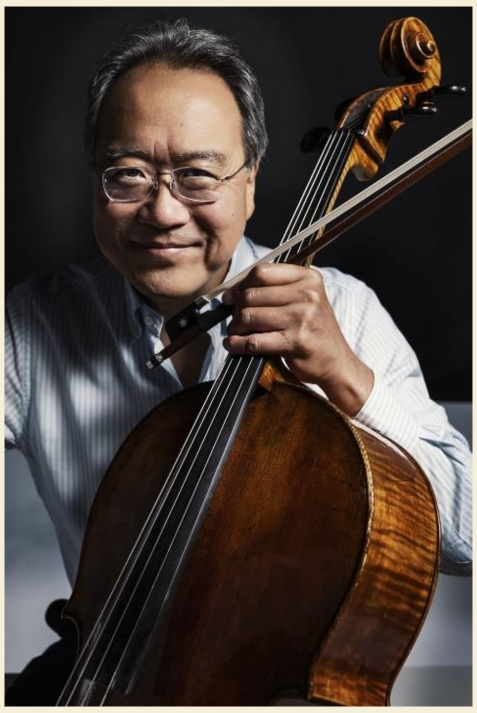

www.harpersbazaar.com

Mohandas Gandhi,
political and spiritual leader

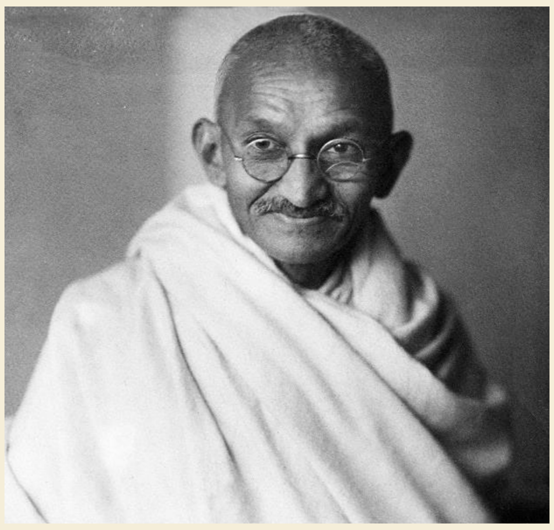

Wikipedia.org

Alberto Giacometti,
sculptor

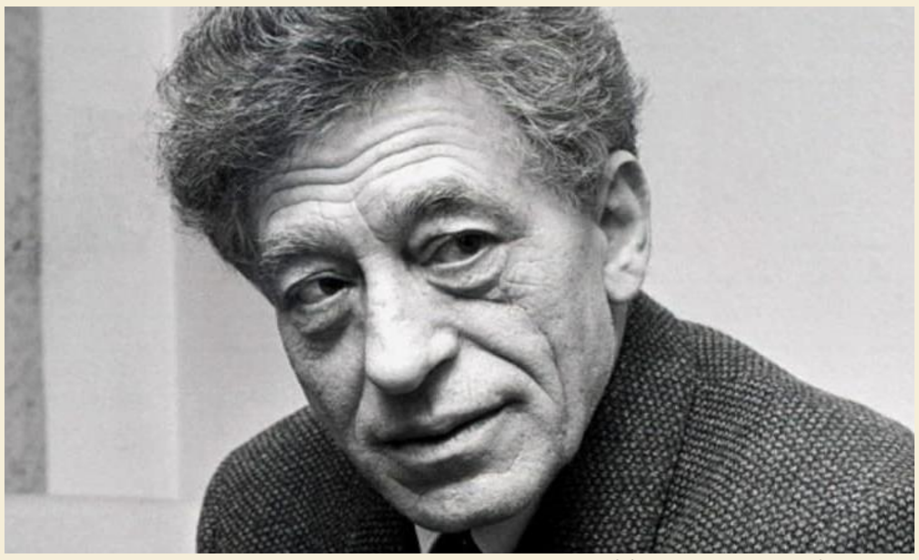

https://www.srf.ch/audio
