# Supplementary material for: Impact of an INtervention to increase MOBility in older hospitalized medical patients (INTOMOB): Study protocol for a cluster randomized controlled trial
Source: BMC Geriatr. 2023 Oct 31;23:705. doi: 10.1186/s12877-023-04285-3 (PMC10617203; doi:10.1186/s12877-023-04285-3)
Supplement: Supplementary file 7 — Additional file 7: Supplement 7. a. Posters. b. Landscapes - environment intervention. c. Flowers - environment intervention. d. Animals - environment intervention. e. - Famous people - environment intervention. [file 12877_2023_4285_MOESM7_ESM.zip › 12877_2023_4285_MOESM7_ESM/Supplement 7b - Landscapes - environment intervention.pdf]

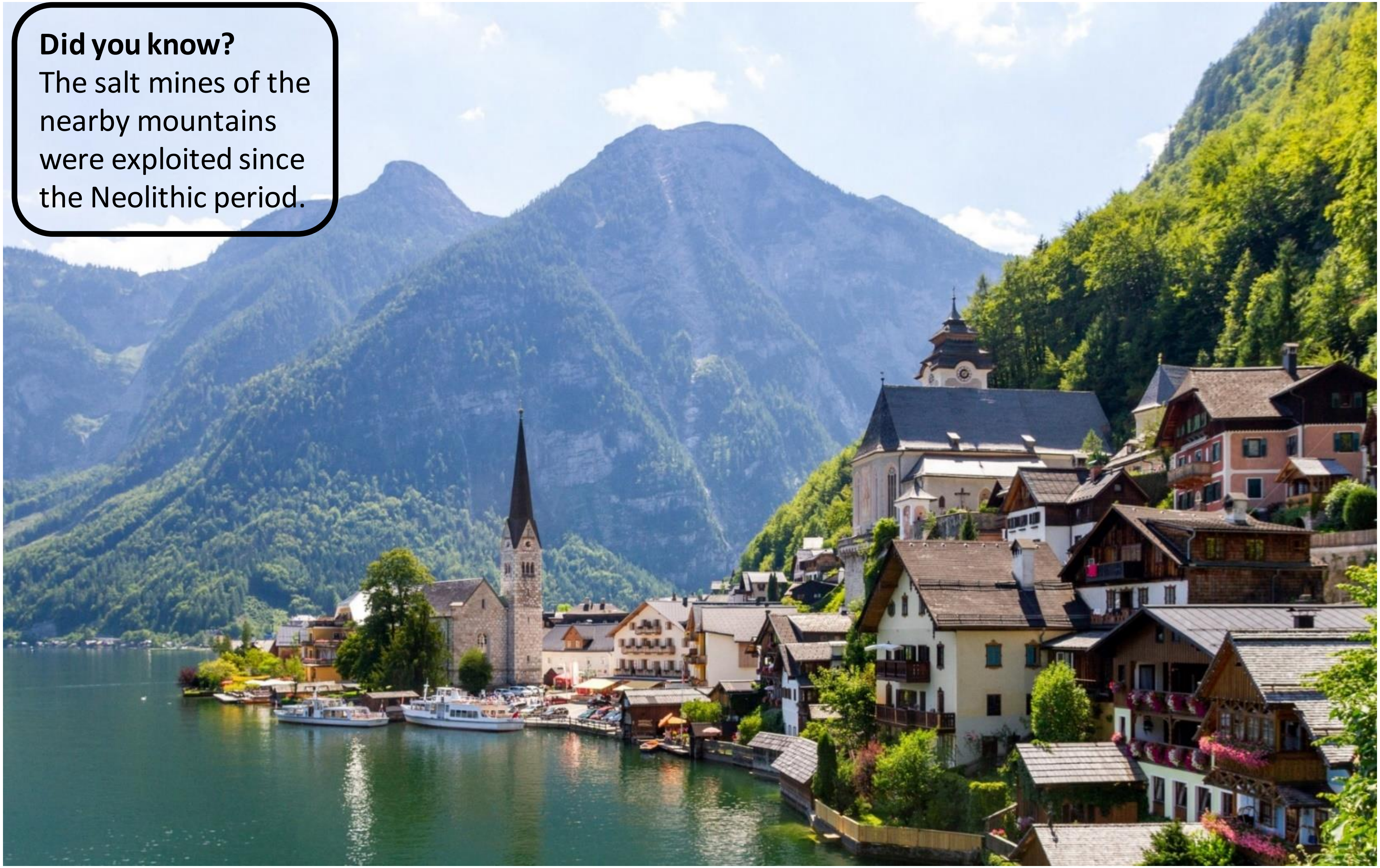

Unsplash.com, wikipedia

Hallstatt, Austria

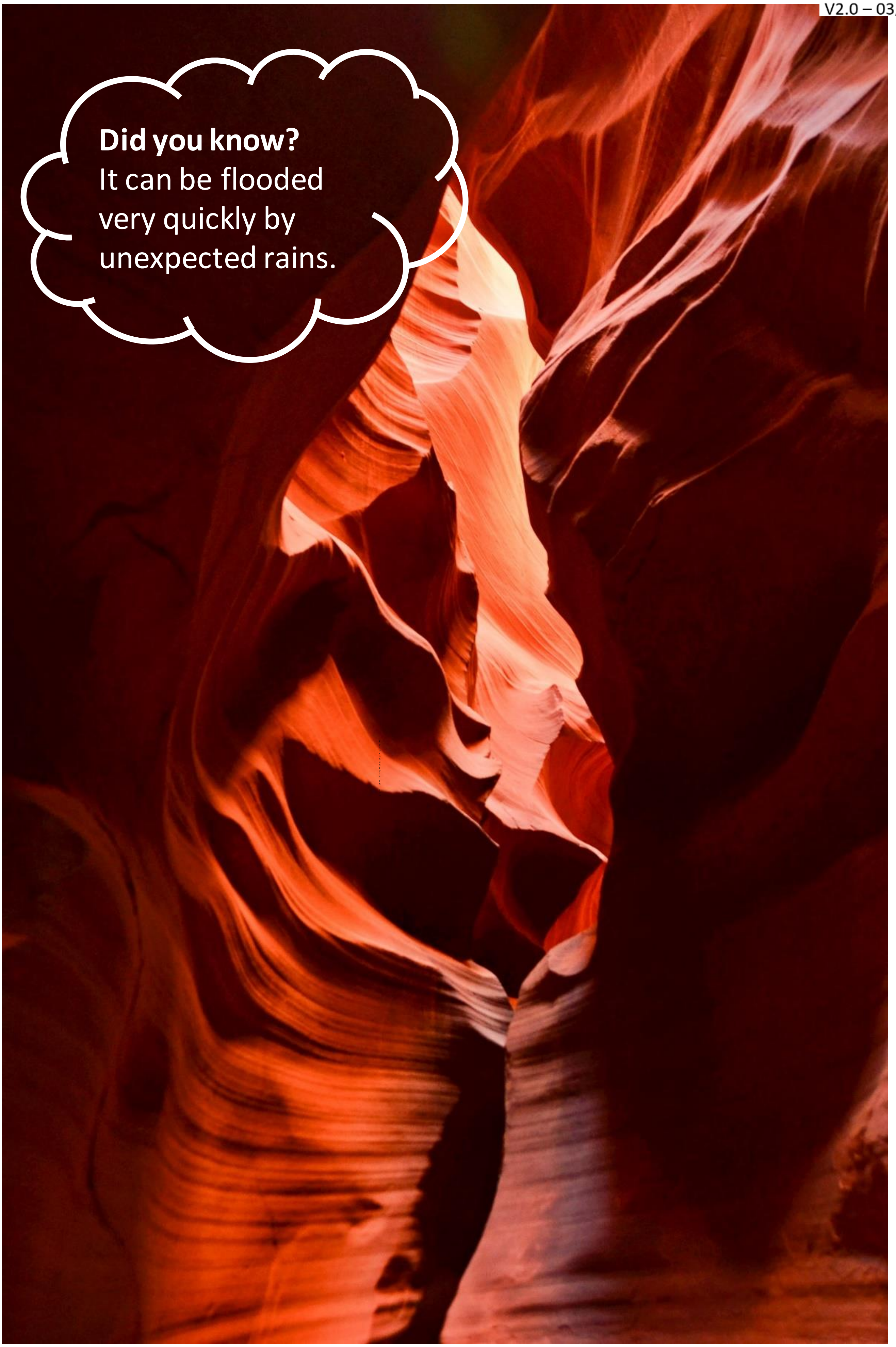

Unsplash.com, wikipedia

Antelope Canyon, Arizona, USA

Did you know?
In 1997, UNESCO included the *Cinque Terre* on the World Heritage List as a cultural landscape.

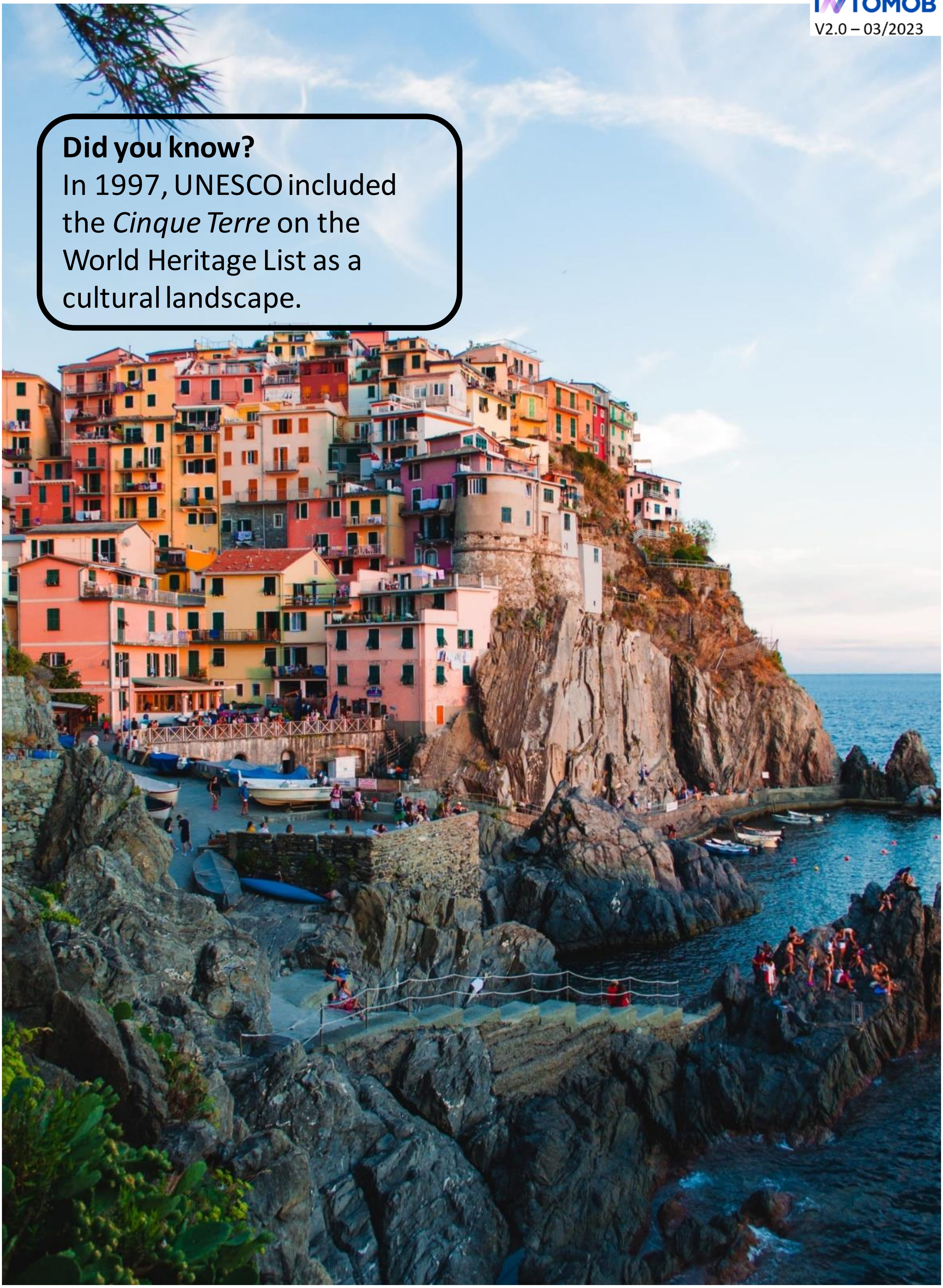

Cinque Terre, Italy

Unsplash.com, wikipedia

Unsplash.com, wikipedia

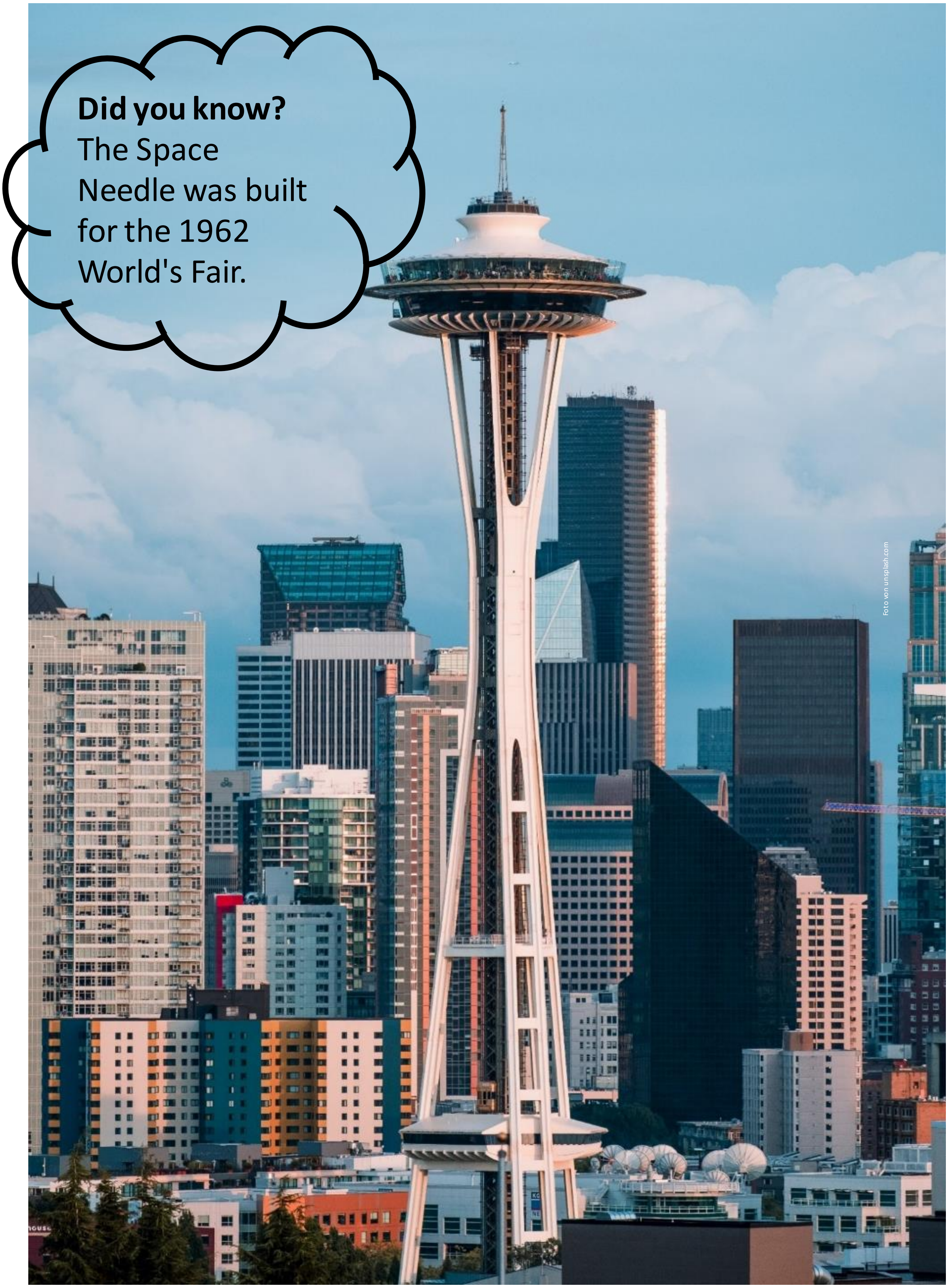

Seattle, Washington, USA

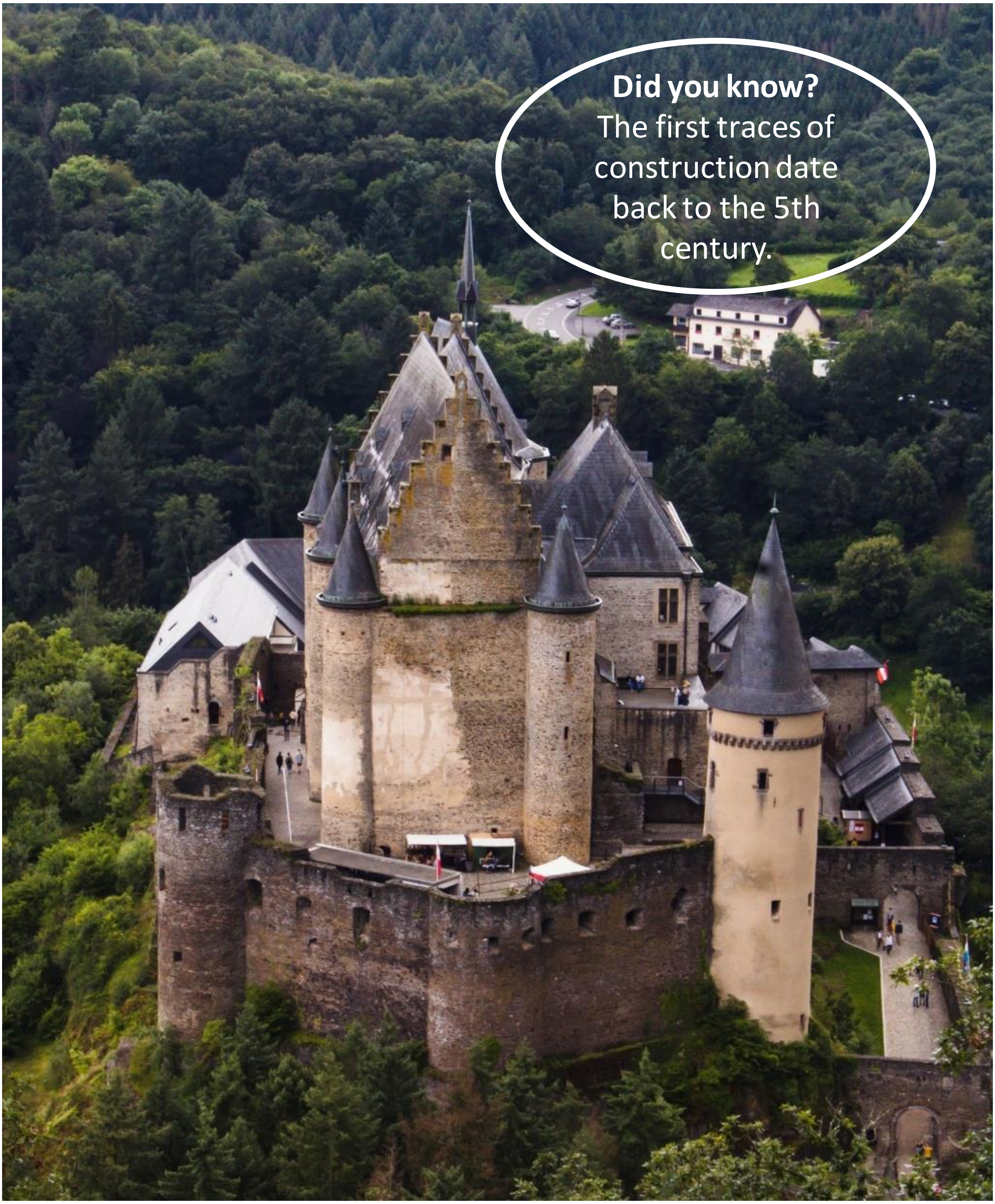

Unsplash.com, wikipedia

Vianden Castle, Luxemburg

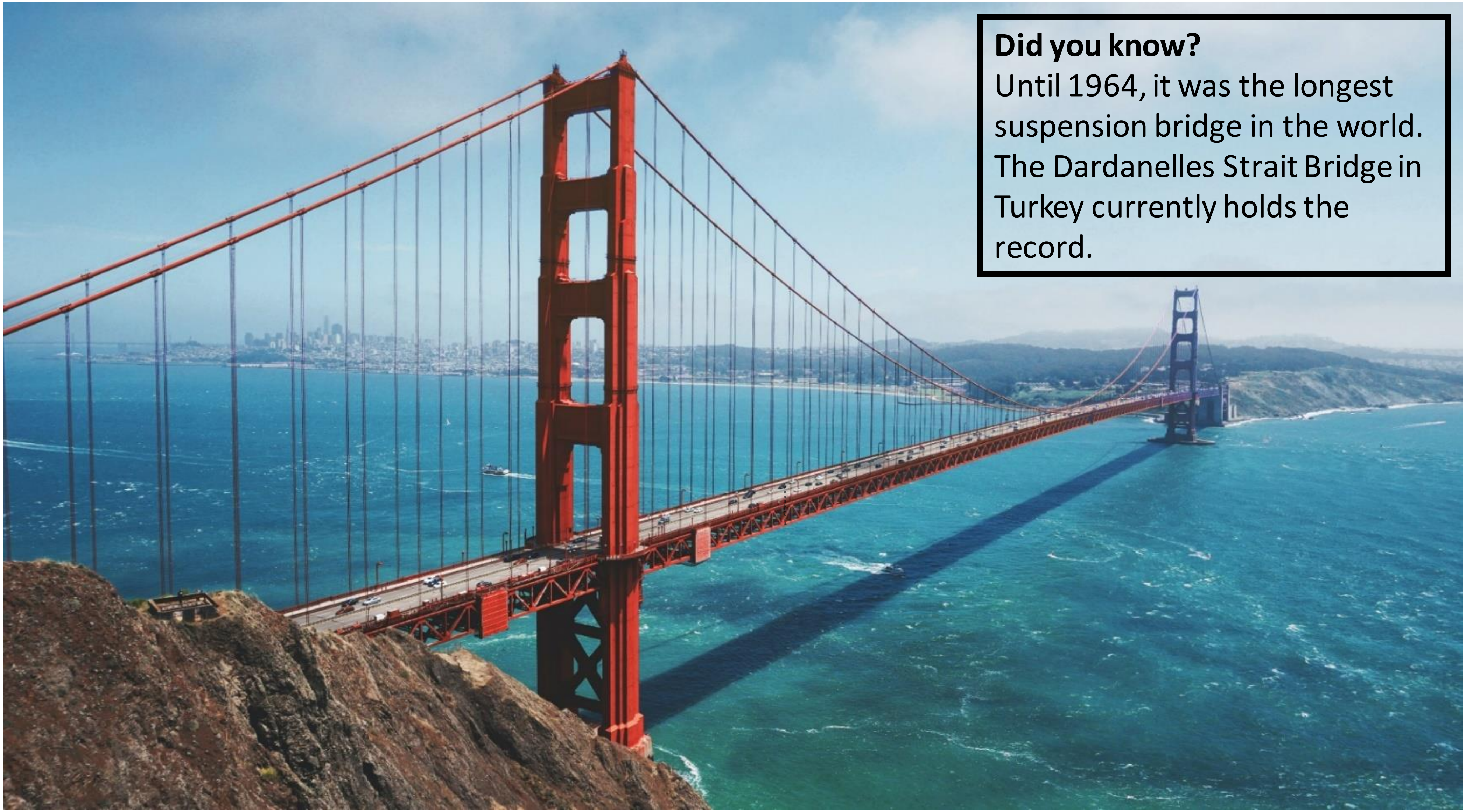

Did you know?
Until 1964, it was the longest suspension bridge in the world. The Dardanelles Strait Bridge in Turkey currently holds the record.

Unsplash.com, wikipedia

Golden Gate Bridge, San Francisco, USA

Unsplash.com, wikipedia

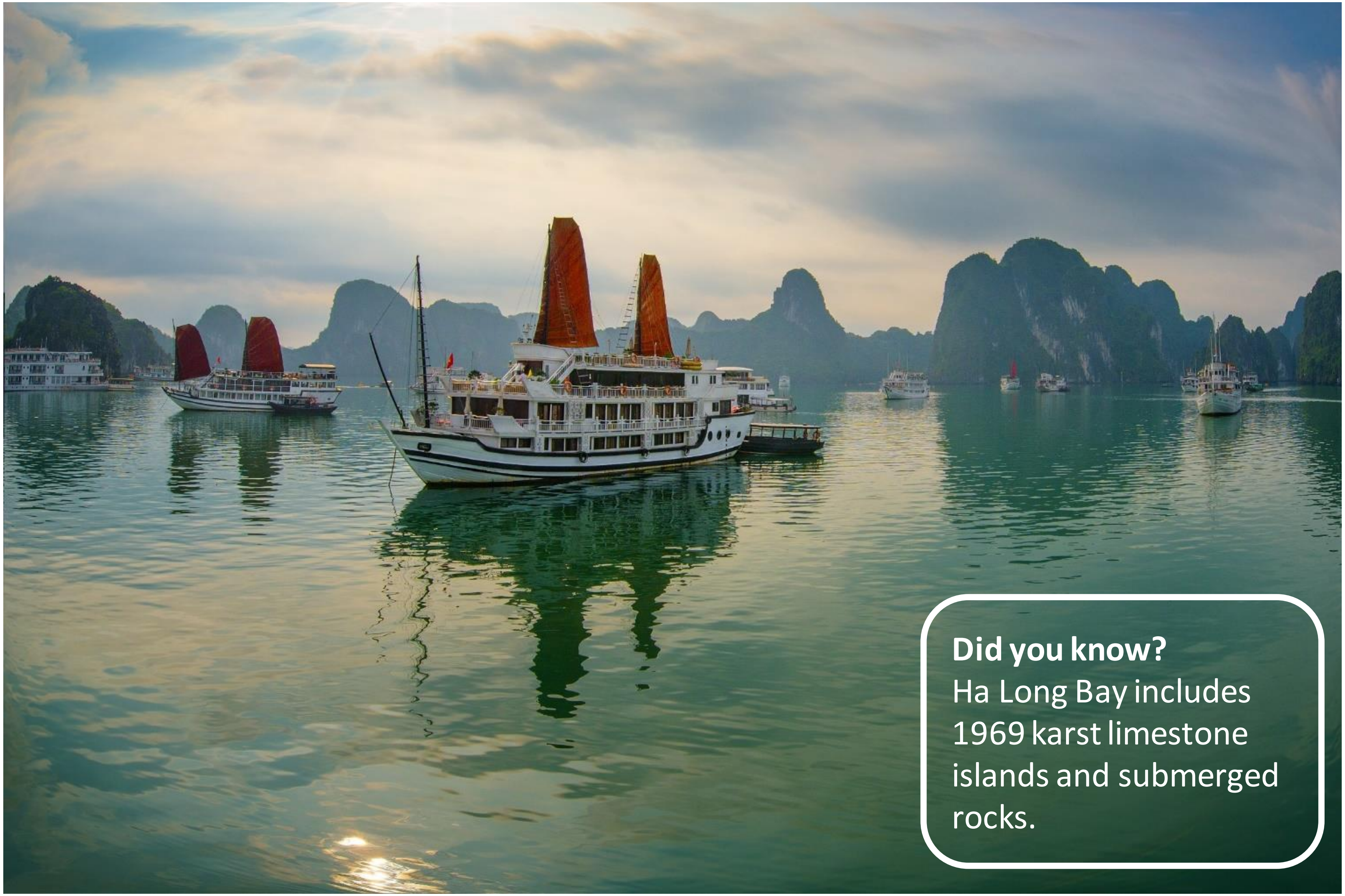

Ha Long Bay, Vietnam

Unsplash.com, wikipedia

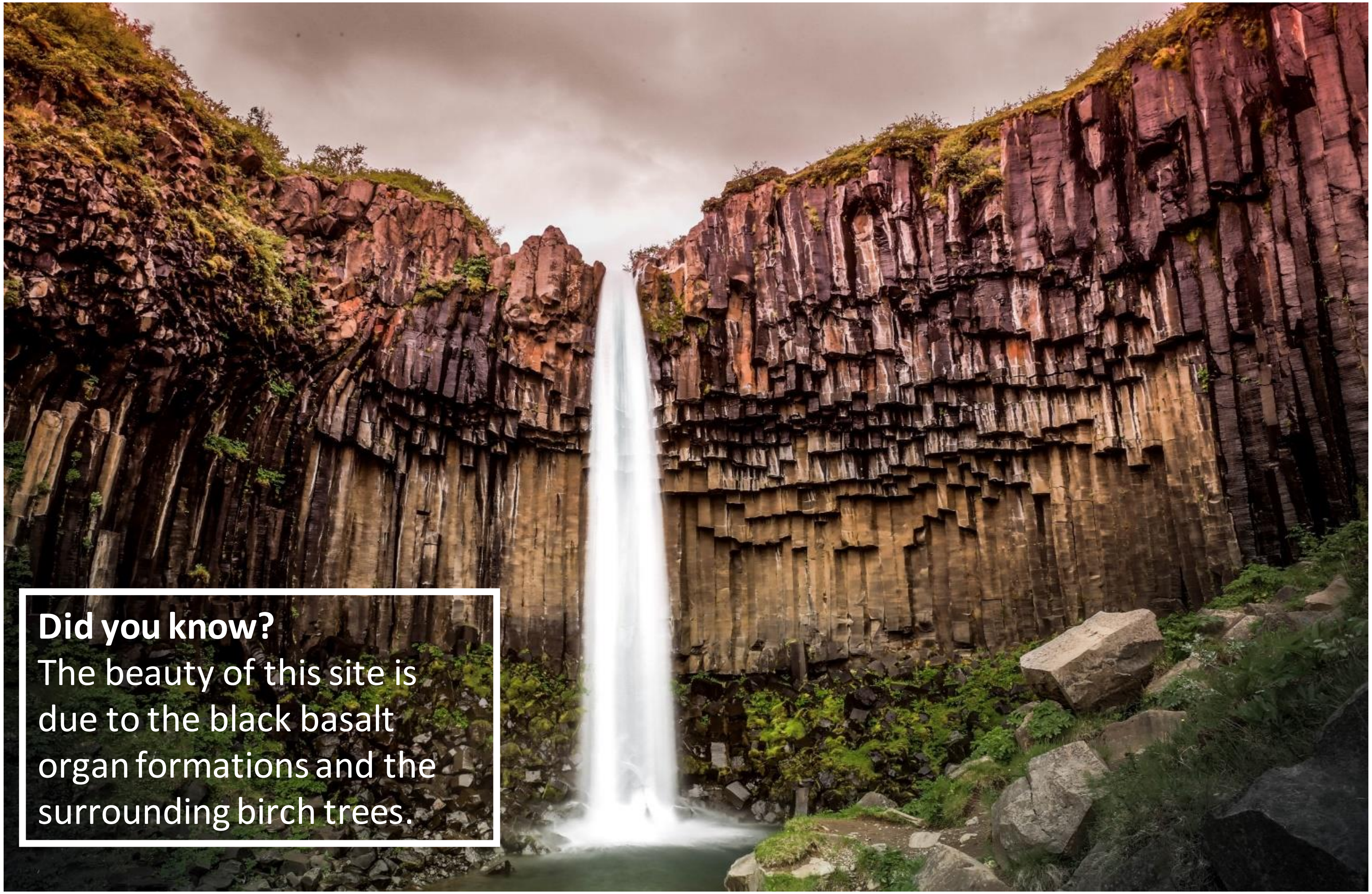

Skaftafell falls, Iceland

Unsplash.com, wikipedia

Did you know?
These mountains are colored because of the various pigments acquired by the minerals that make up the sedimentary layers.

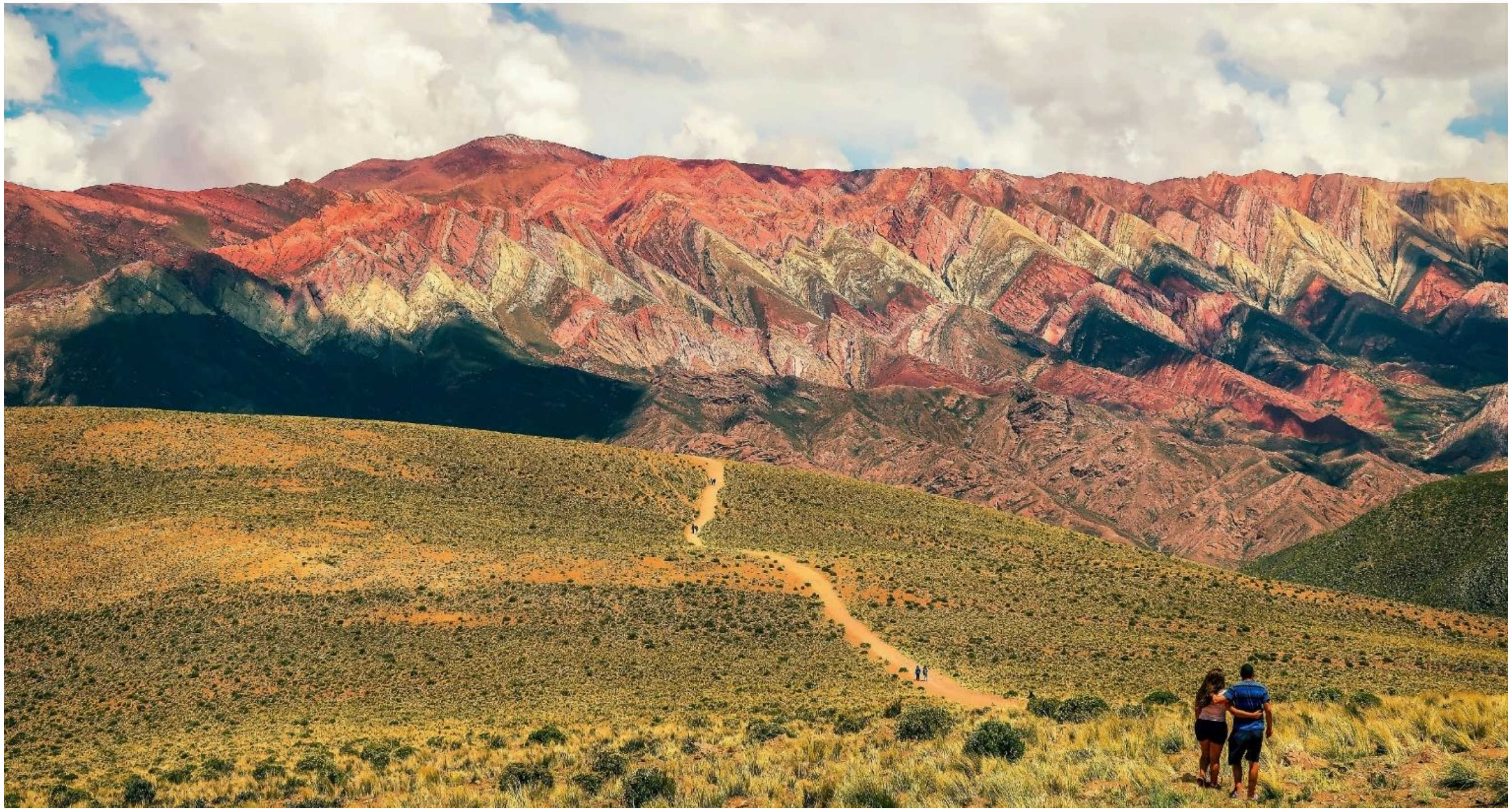

Jujuy, Argentina

Did you know?
Every year, 3-5 million tourists visit this park which has an area of 3'081km^2.

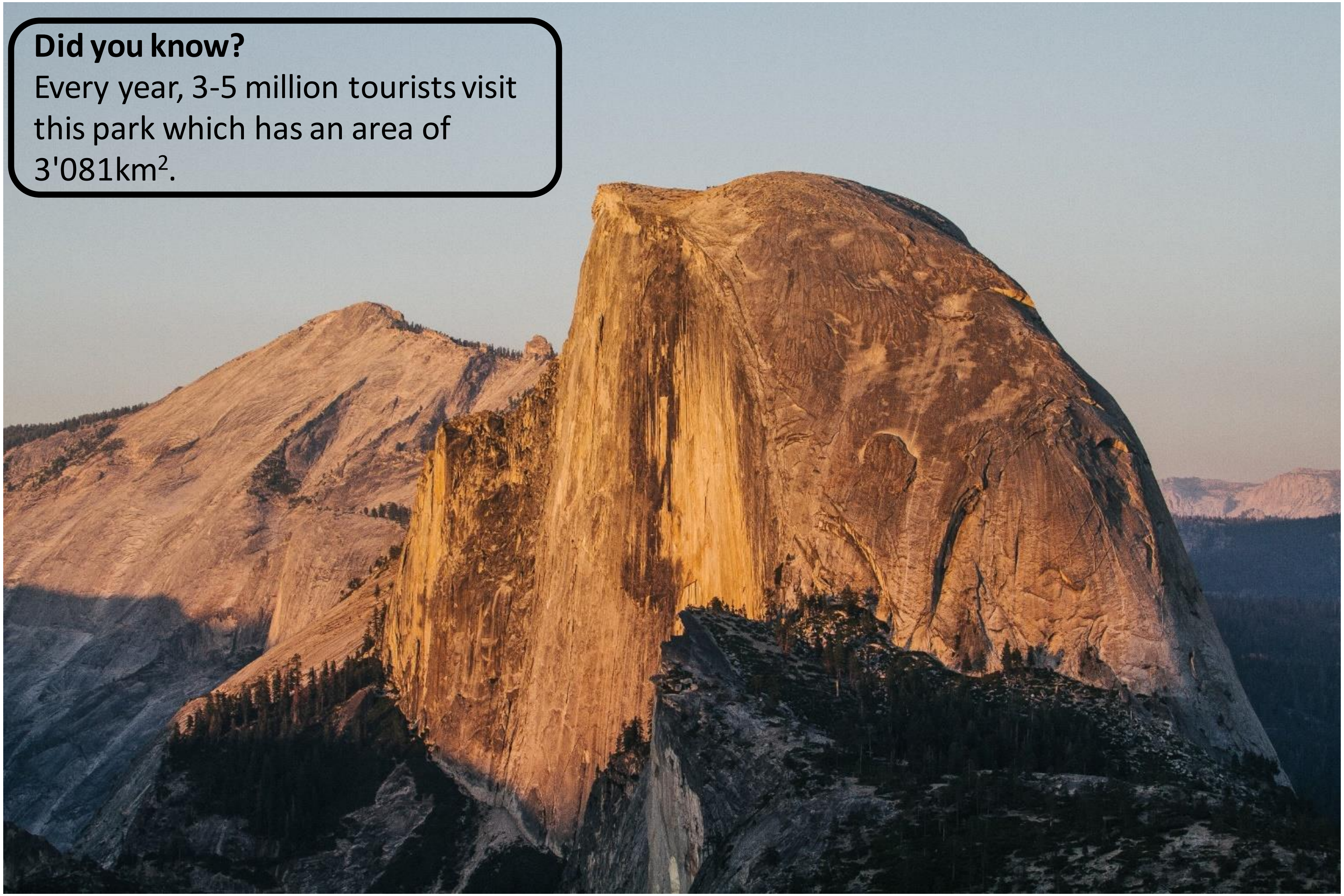

Unsplash.com, wikipedia

Half Dome, Yosemite National park, USA

INTOMOB
V2.0 – 03/2023

Did you know?
With a flow of more than 2'800m^3/s, they are the most powerful falls in North America.

Unsplash.com, wikipedia

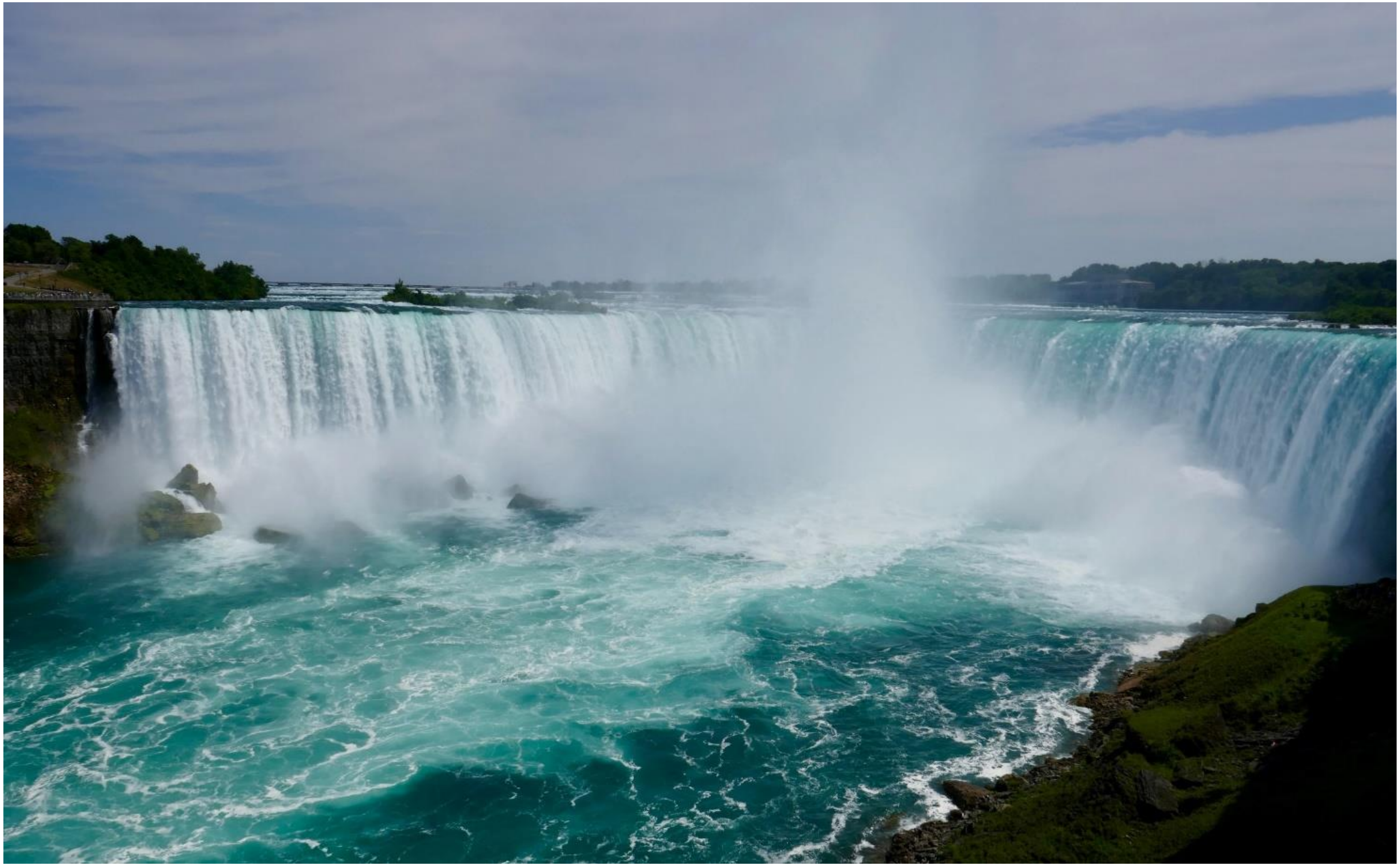

Niagara falls, Canada

INTOMOB
V2.0 – 03/2023

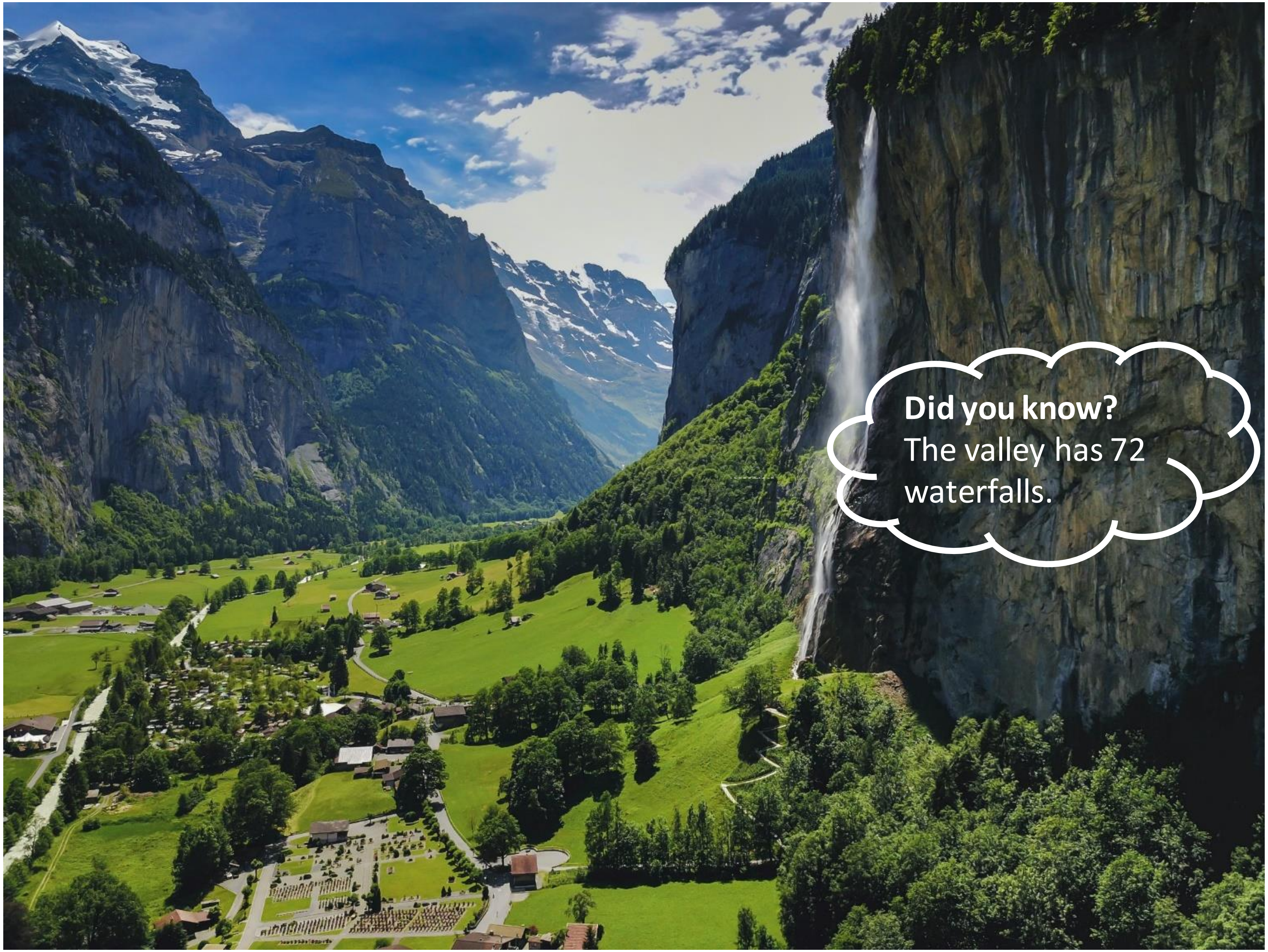

Unsplash.com, wikipedia

Lauterbrunnen, Switzerland

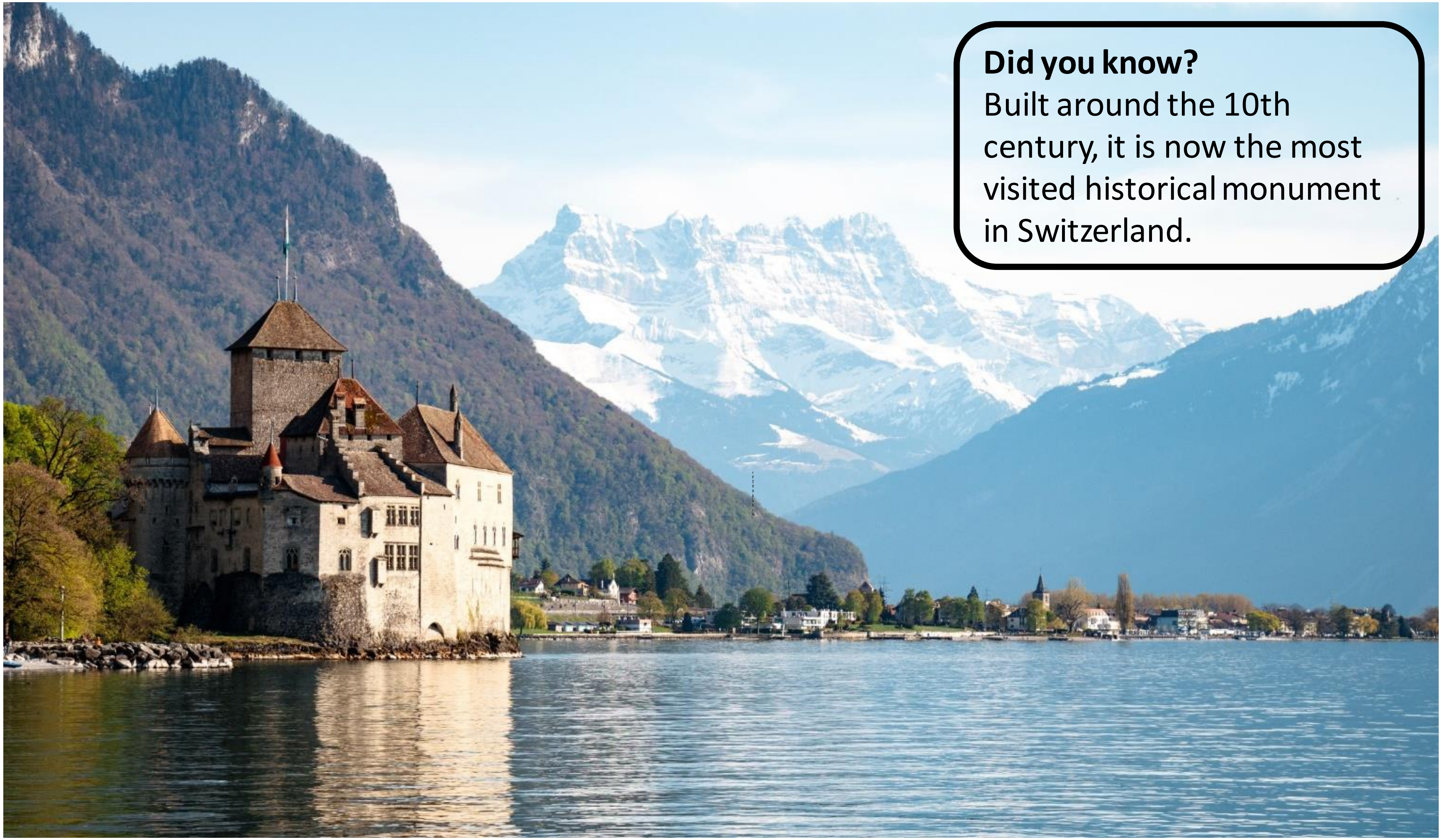

Unsplash.com, wikipedia

Chillon Castle, Switzerland

Did you know?
James Bond jumped from the 220m high dam in the movie "Golden Eye".

INTOMOB
V2.0 – 03/2023

Unsplash.com, wikipedia

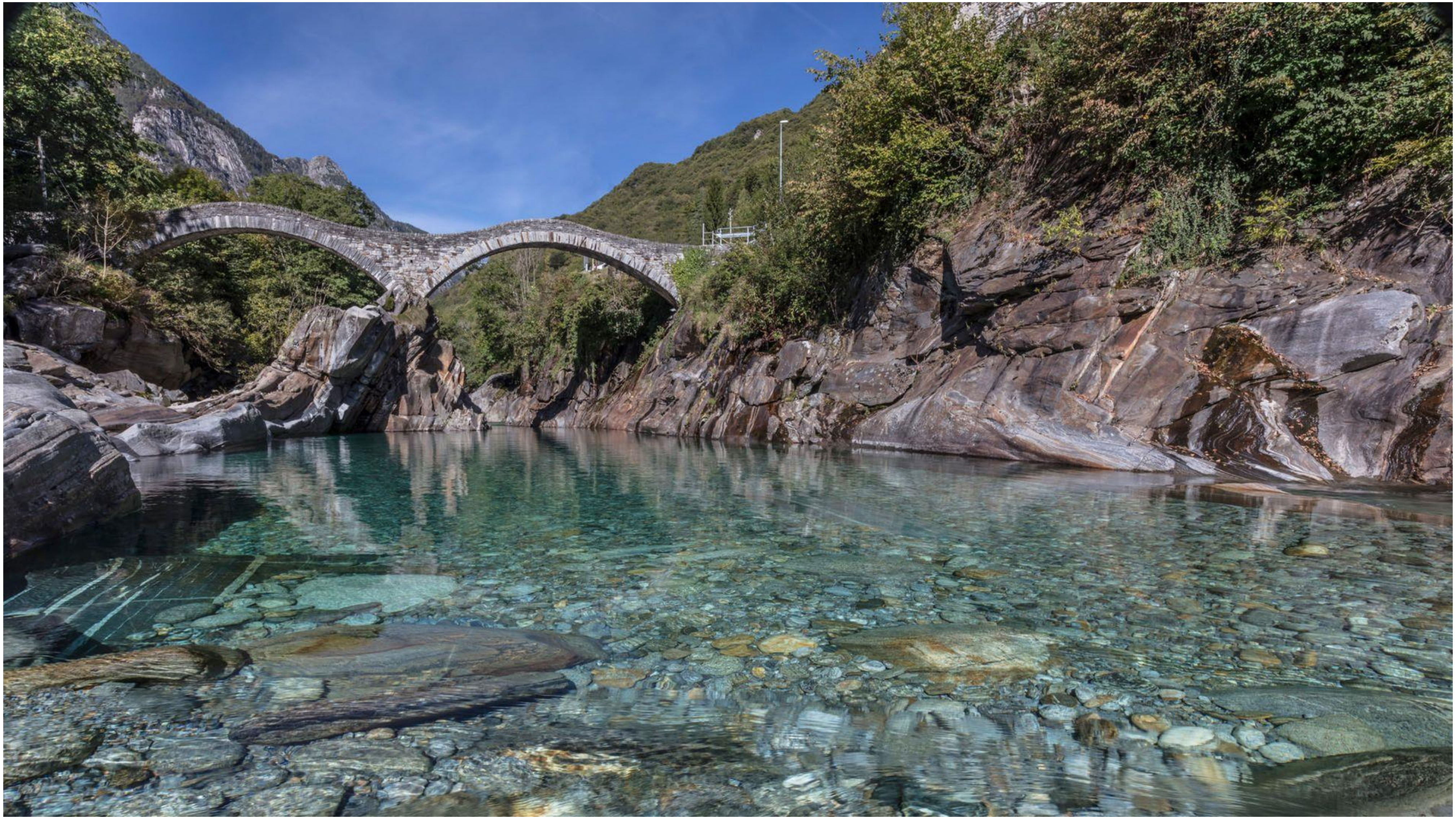

Valle Verzasca, Switzerland
