# Supplementary material for: Impact of an INtervention to increase MOBility in older hospitalized medical patients (INTOMOB): Study protocol for a cluster randomized controlled trial
Source: BMC Geriatr. 2023 Oct 31;23:705. doi: 10.1186/s12877-023-04285-3 (PMC10617203; doi:10.1186/s12877-023-04285-3)
Supplement: Supplementary file 7 — Additional file 7: Supplement 7. a. Posters. b. Landscapes - environment intervention. c. Flowers - environment intervention. d. Animals - environment intervention. e. - Famous people - environment intervention. [file 12877_2023_4285_MOESM7_ESM.zip › 12877_2023_4285_MOESM7_ESM/Supplement 7c - Flowers - environment intervention.pdf]

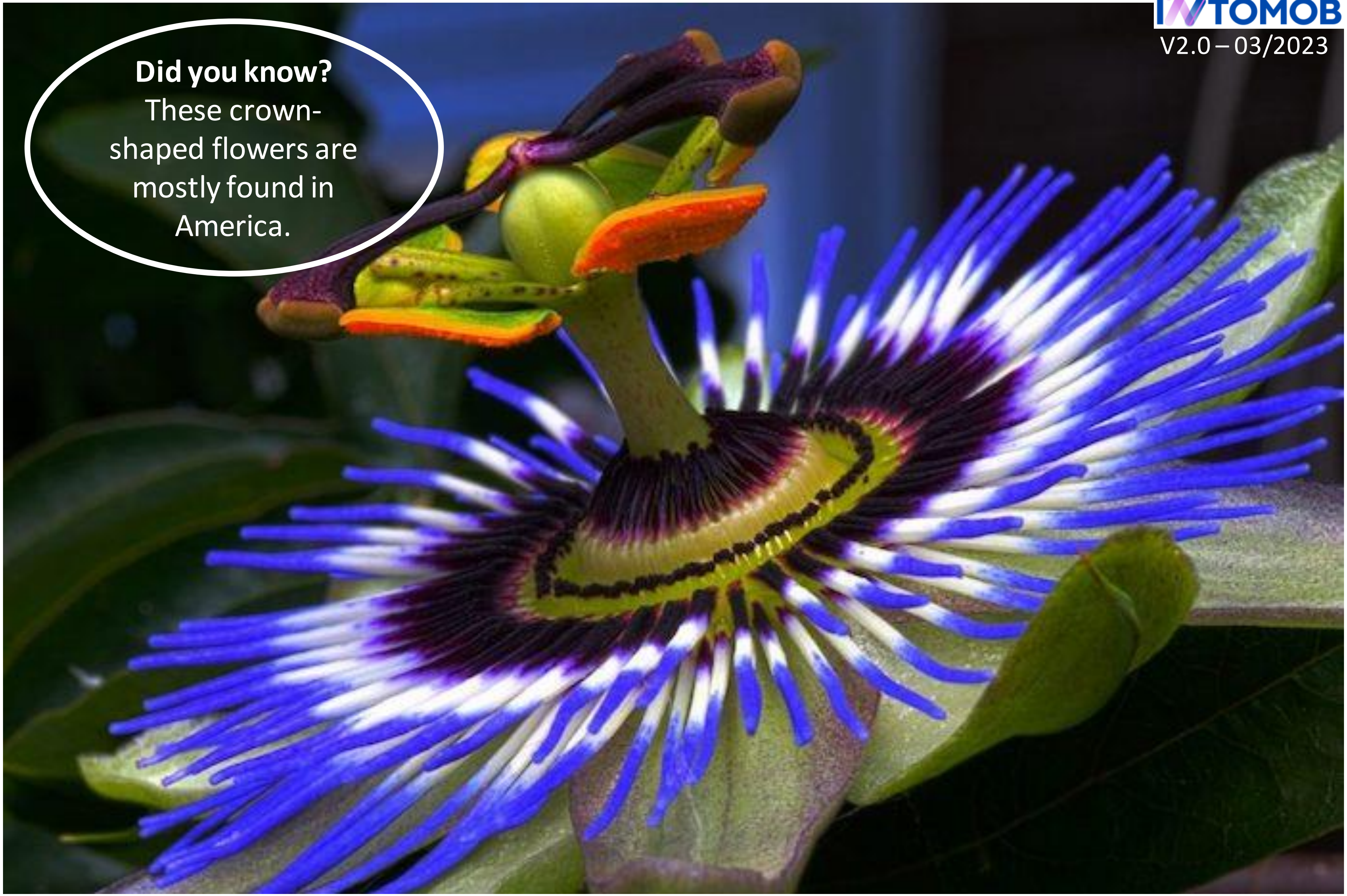

INTOMOB
V2.0 – 03/2023

https://archzine.fr/jardin, Wikipedia

Passion flower

Did you know?
The poppy owes its name to a metaphor between its color and that of the crest of the rooster.

https://www.notretemps.com
https://www.lemonde.fr

INTOMOB
V2.0 – 03/2023

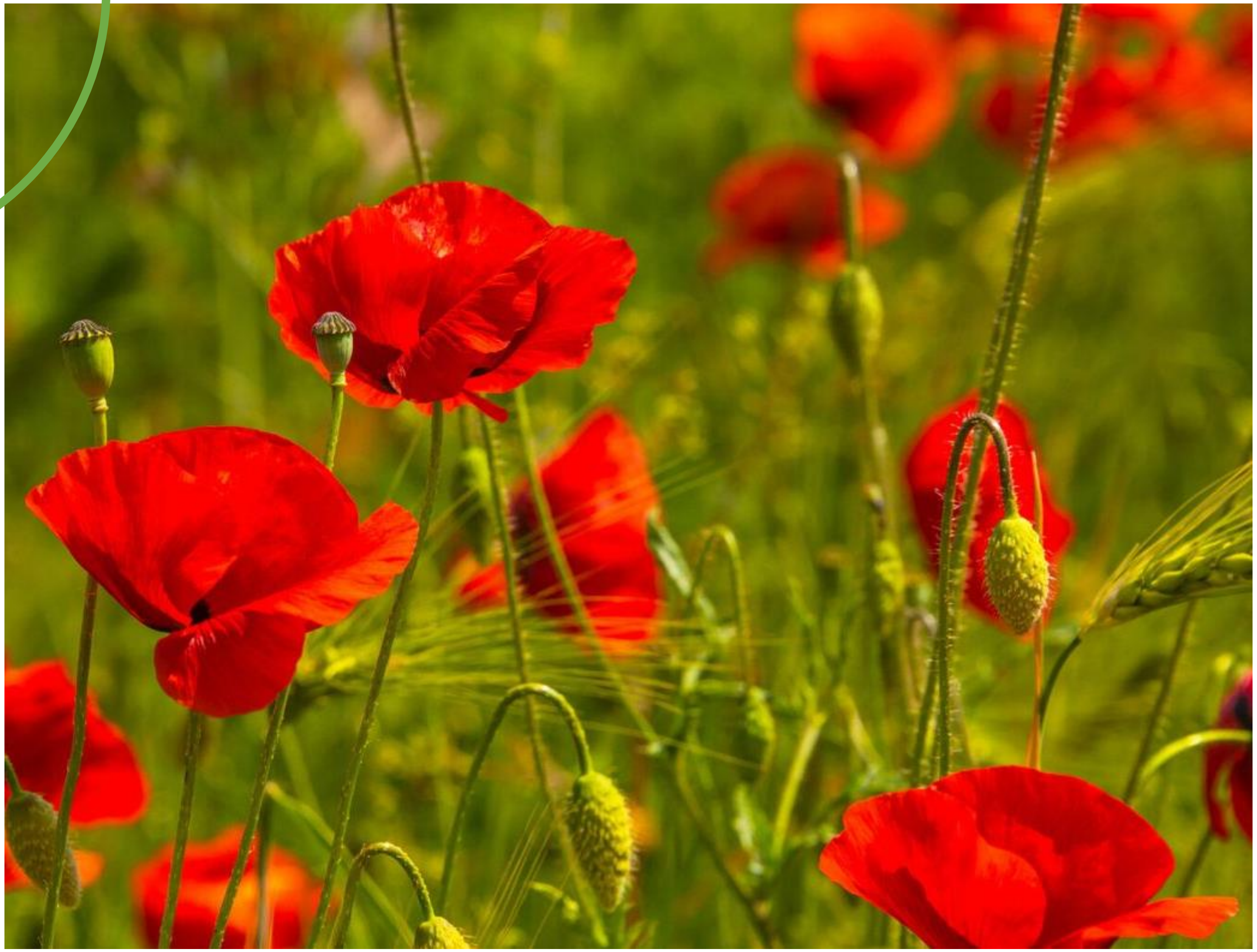

Poppy

INTOMOB
V2.0 – 03/2023

https://www.sac-cas.ch, wikipedia

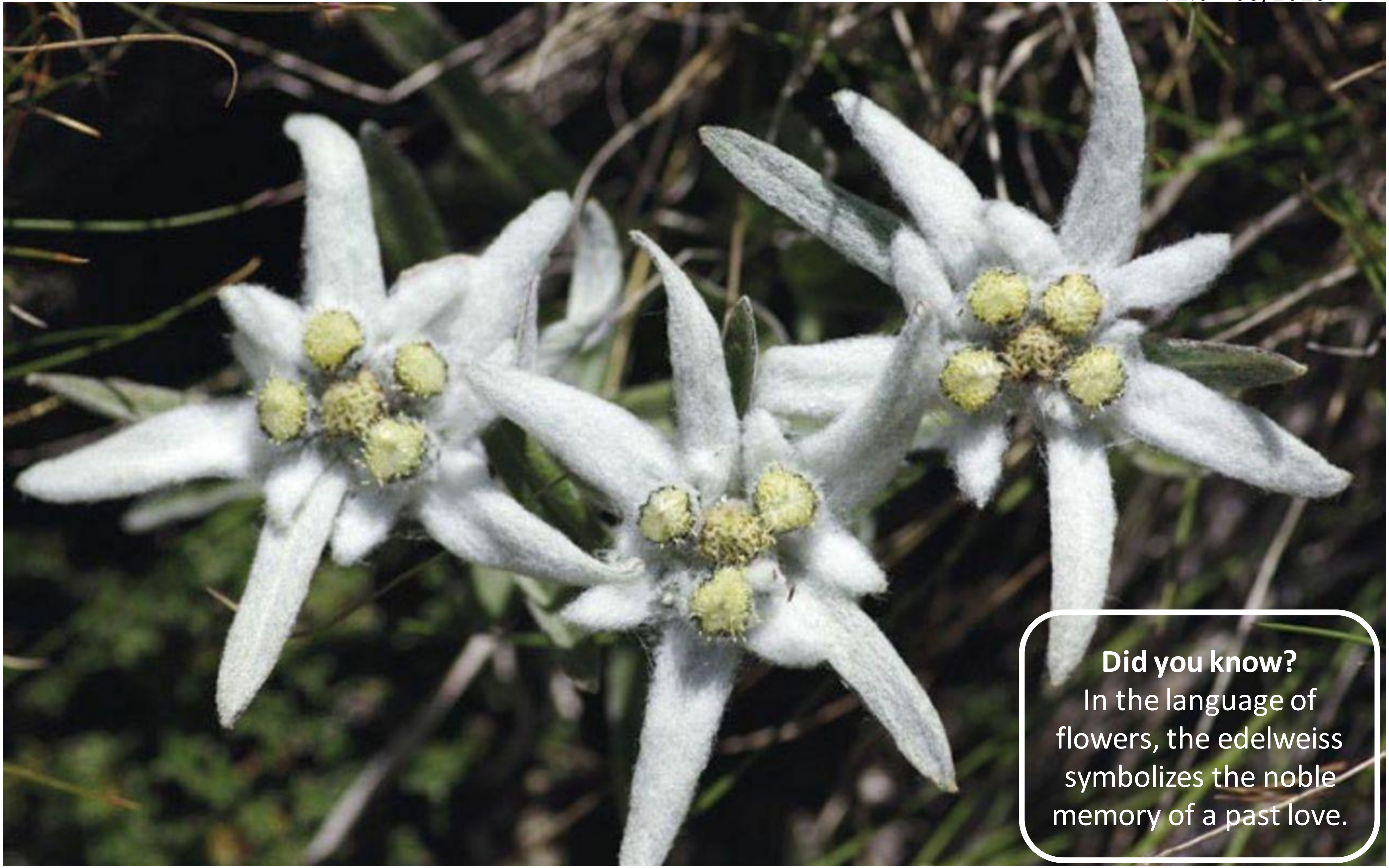

Did you know?
In the language of flowers, the edelweiss symbolizes the noble memory of a past love.

Edelweiss

https://archzine.f, wikipedia

V2.0 – 03/2023

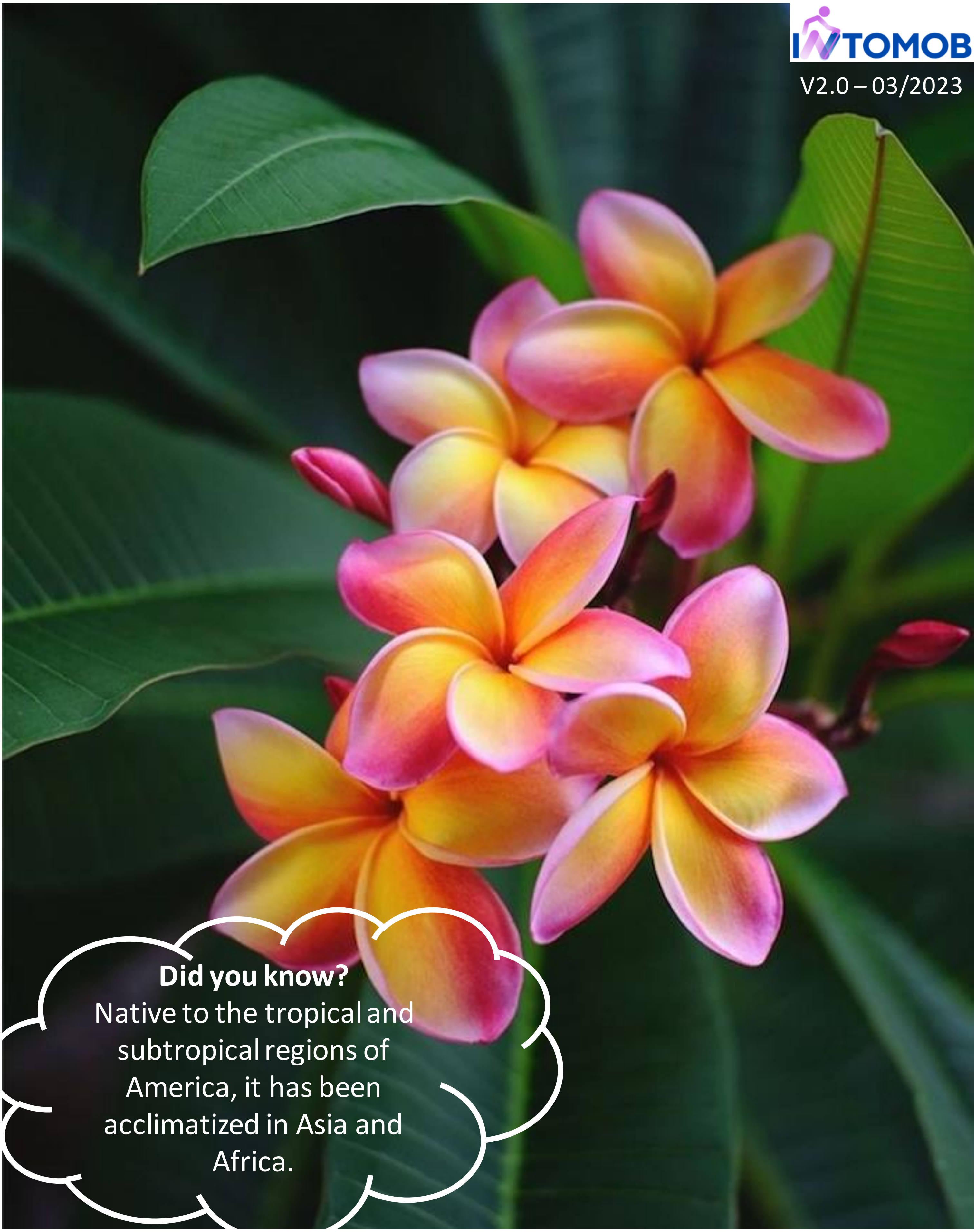

Frangipani

Schon gewusst?
Die Margerite wurde im 17. Jahrhundert in Spanien roh als Salat gegessen.

INTOMOB
V2.0 – 03/2023

https://www.gerbeaud.com/jardin, wikipedia

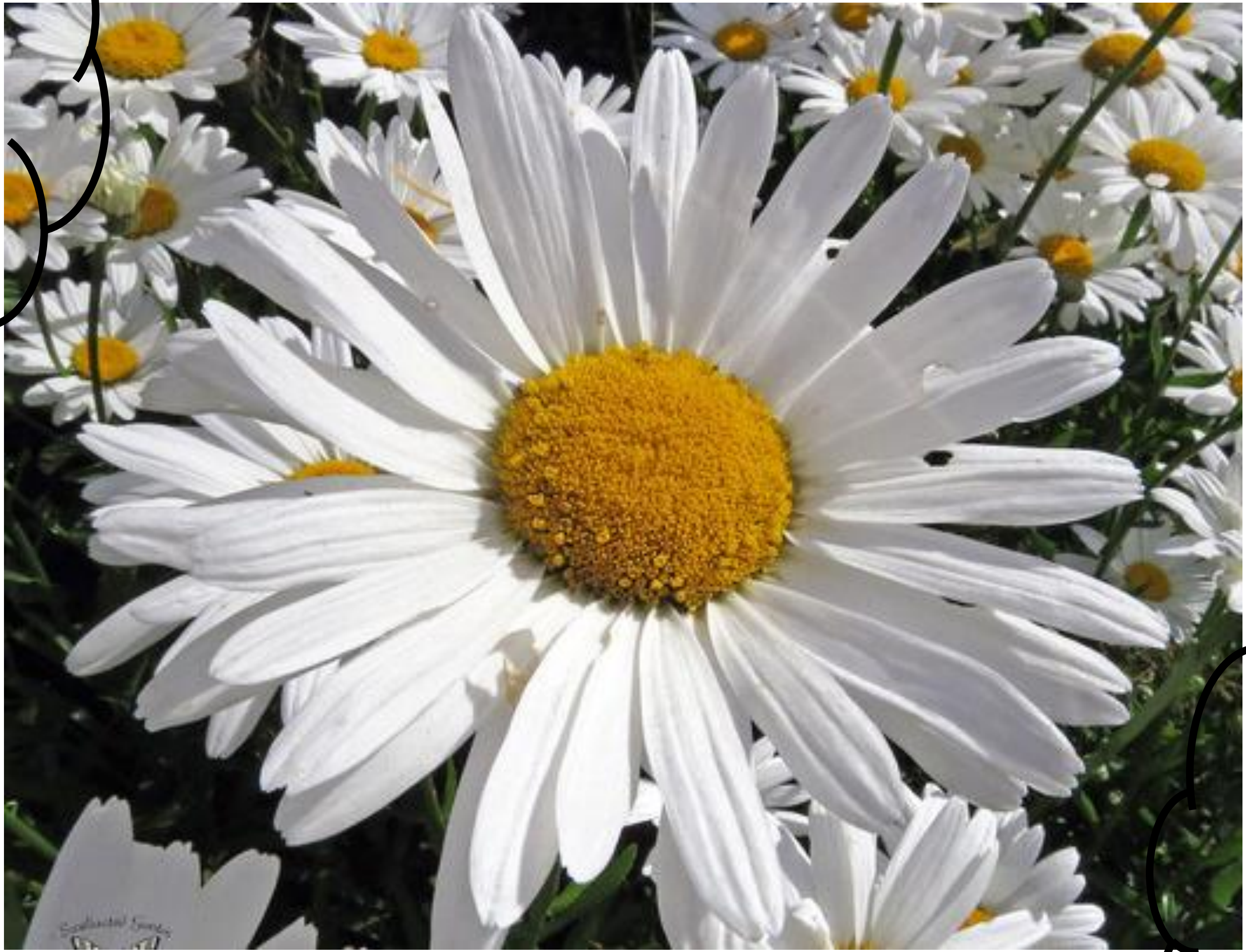

Marguerite / Margerite

Le saviez-vous?
On la consommait crue en salade au 17ème siècle en Espagne.

https://archzine.fr/jardin, https://www.homejardin.com

INTOMOB
V2.0 – 03/2023

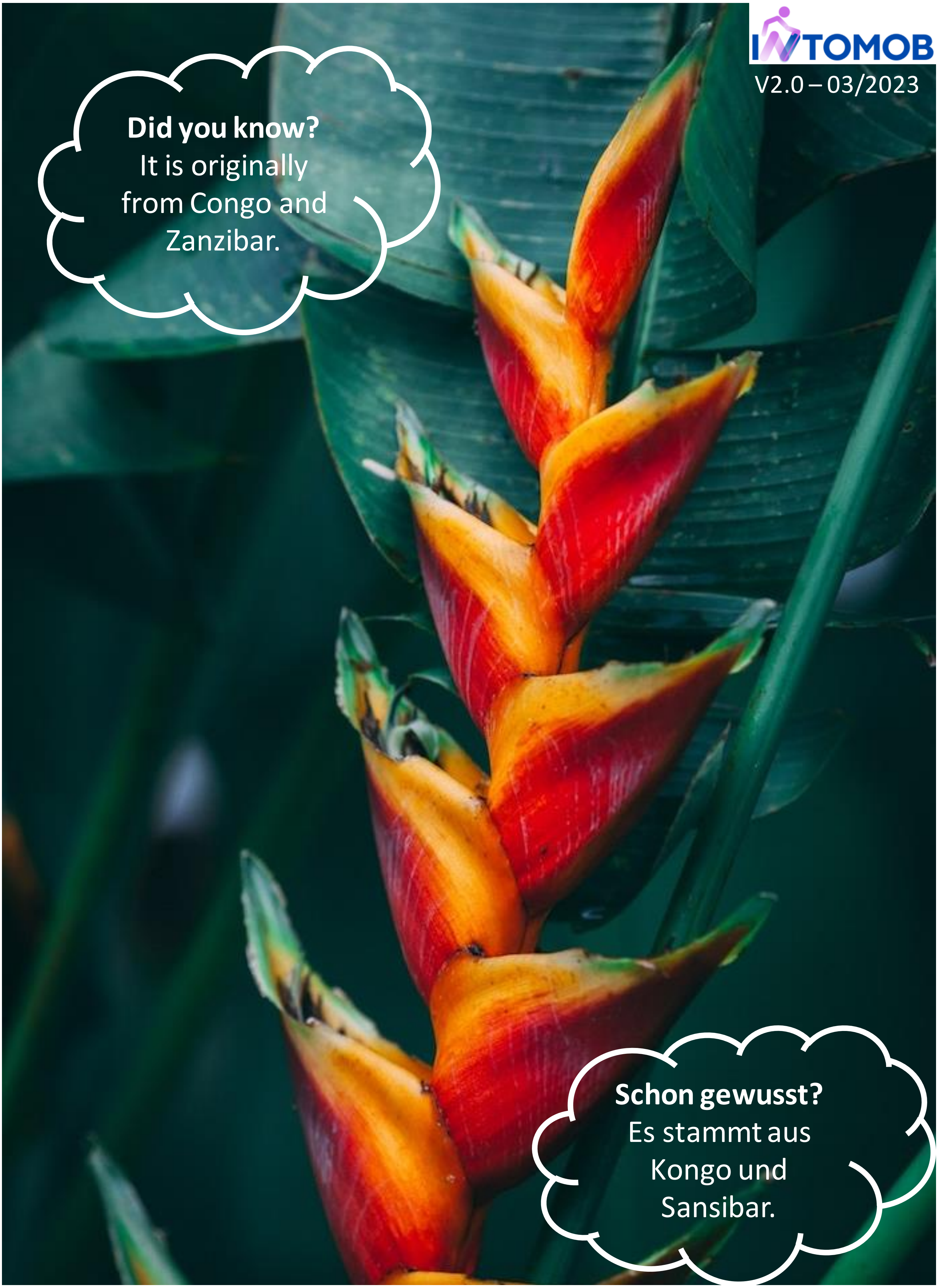

Red-yellow flower of Congo

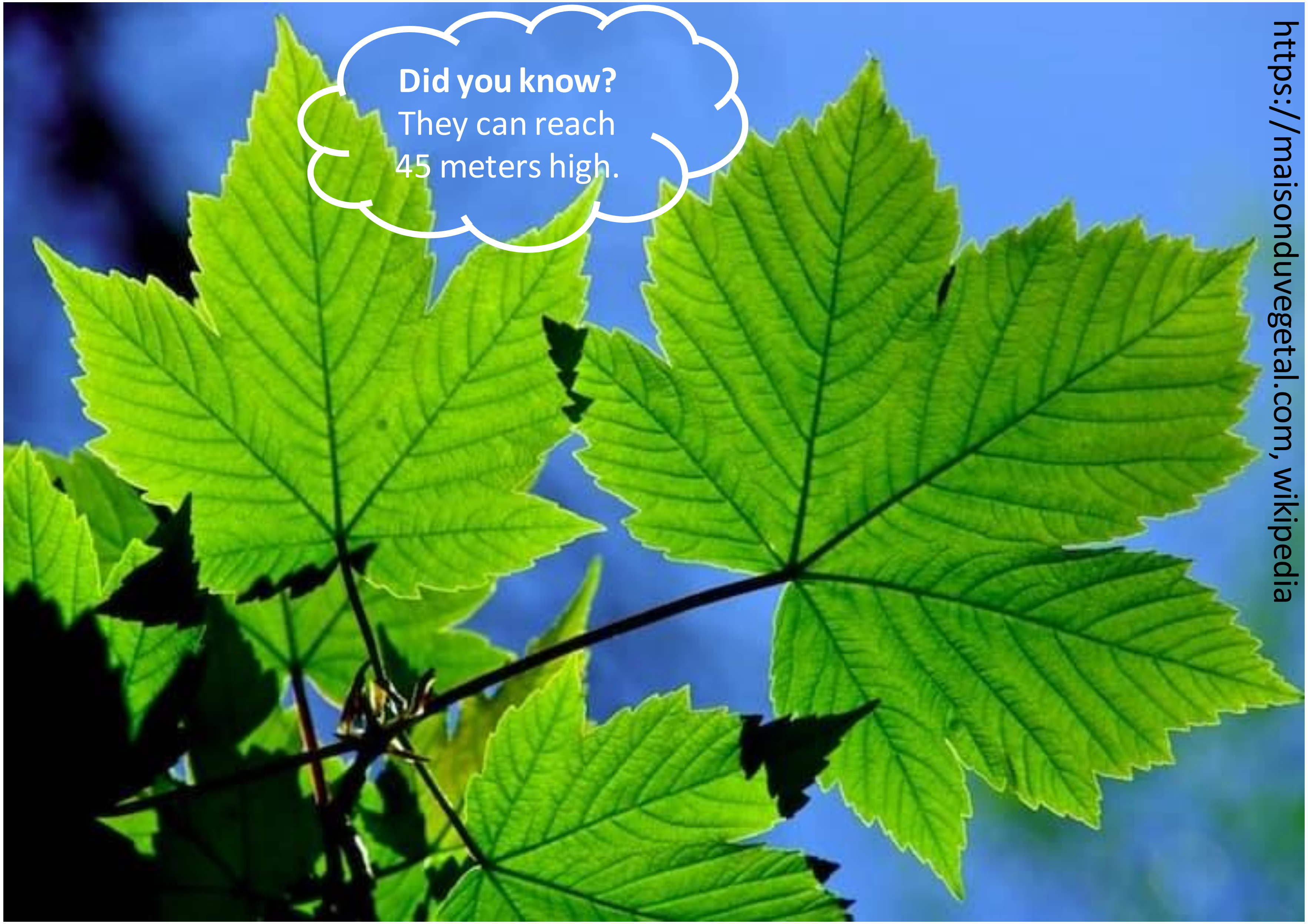
Did you know?
They can reach
45 meters high.
https://maisonduvegetal.com, wikipedia

Maple

https://www.bio-enligne.com, wikipedia

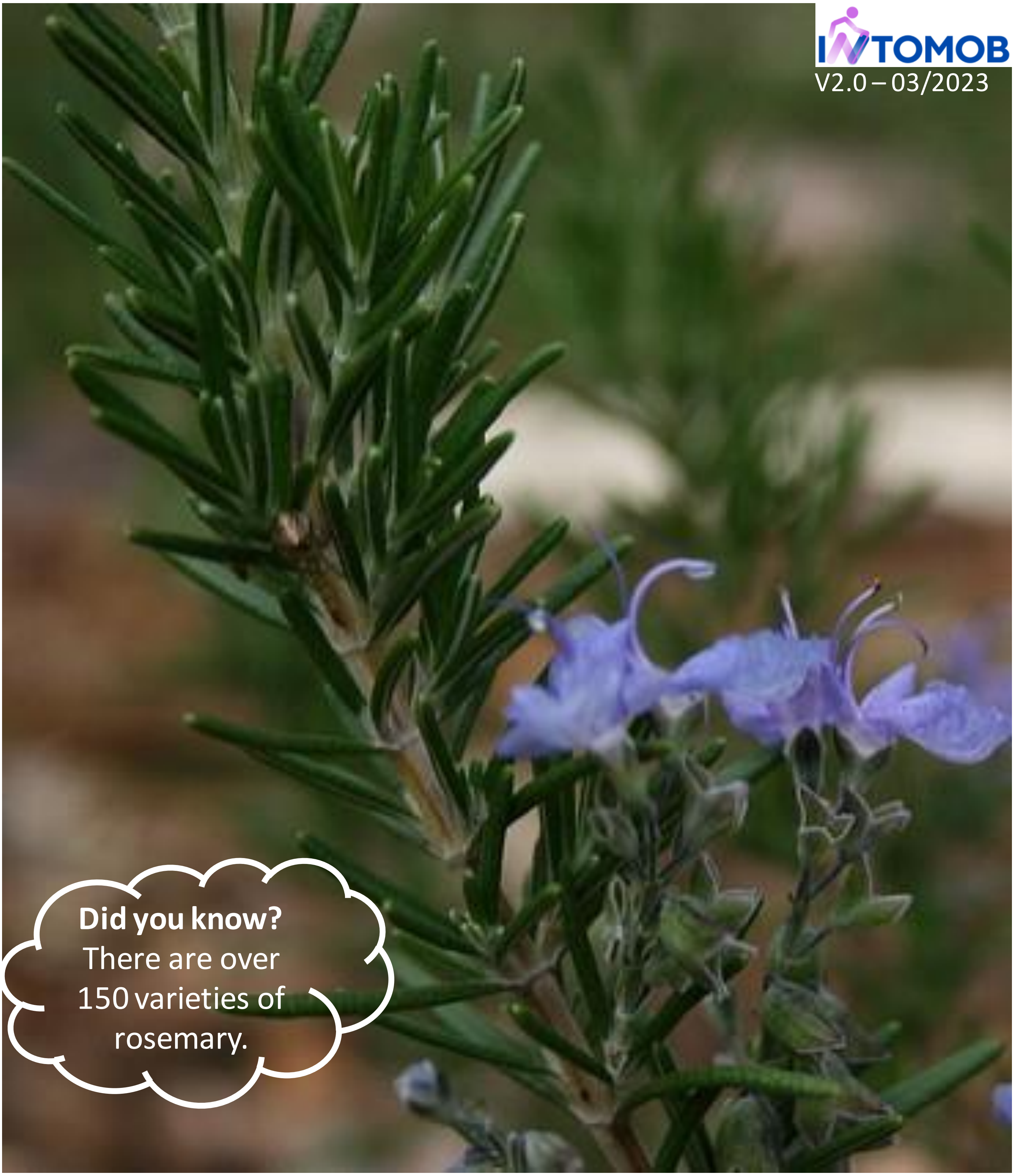

Rosmary

INTOMOB
V2.0 – 03/2023

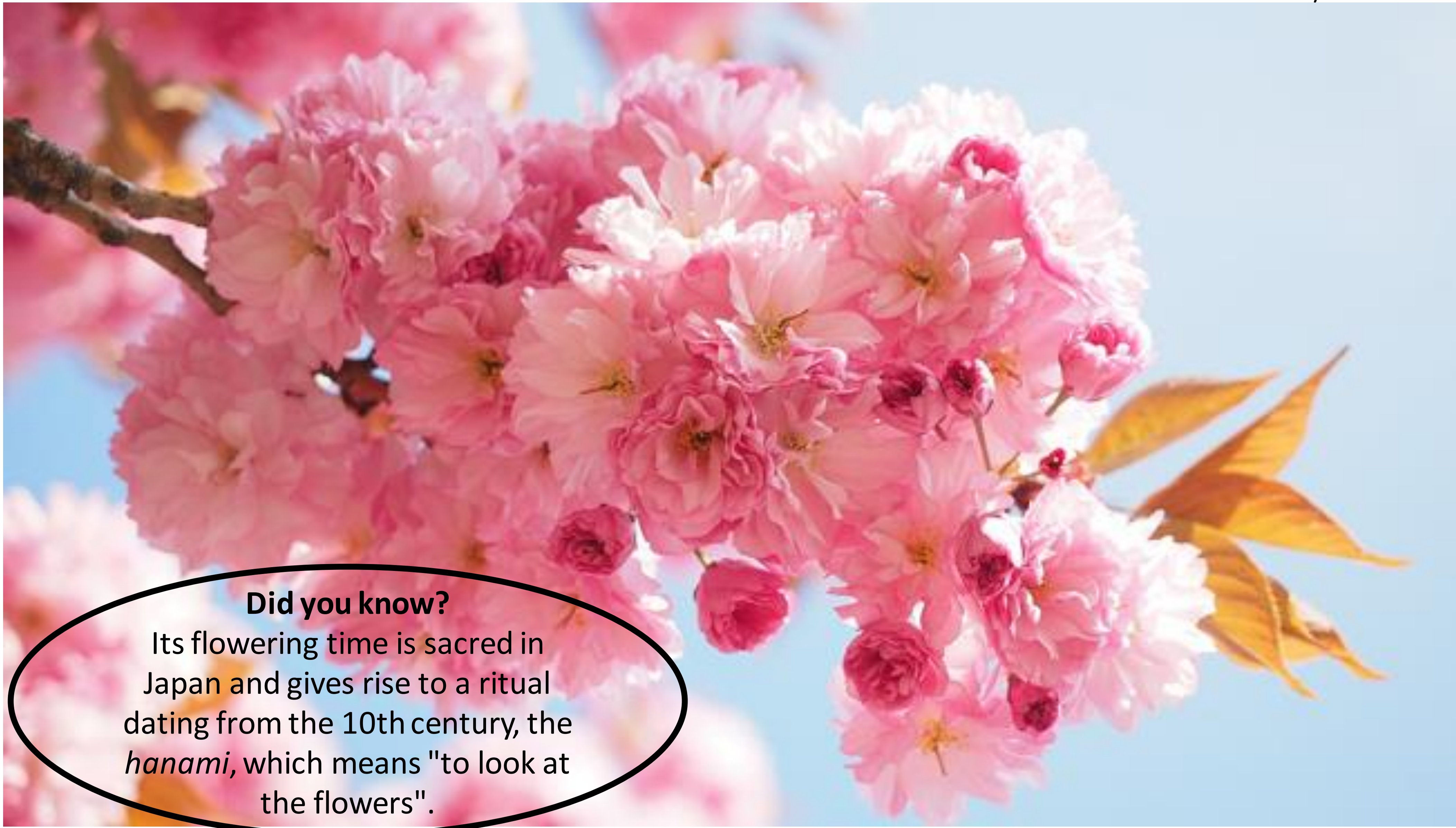

Did you know?
Its flowering time is sacred in Japan and gives rise to a ritual dating from the 10th century, the *hanami*, which means "to look at the flowers".

https://jardinage.lemonde.fr, https://www.gammvert.fr

Japanese cherry

https://www.almanac.com, https://www.jardiner-malin.fr

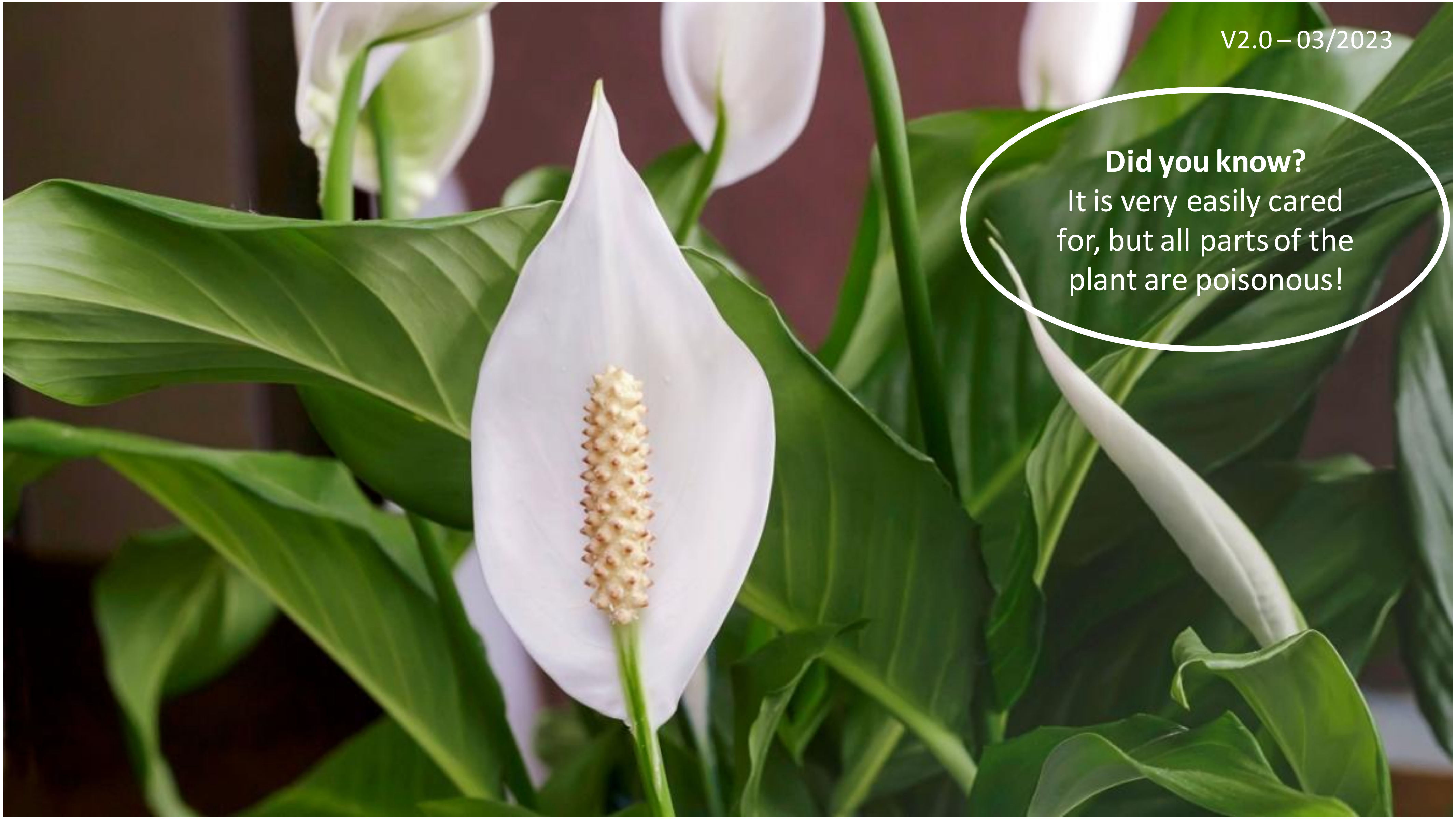

Spathiphyllum
