# Supplementary material for: Impact of an INtervention to increase MOBility in older hospitalized medical patients (INTOMOB): Study protocol for a cluster randomized controlled trial
Source: BMC Geriatr. 2023 Oct 31;23:705. doi: 10.1186/s12877-023-04285-3 (PMC10617203; doi:10.1186/s12877-023-04285-3)
Supplement: Supplementary file 7 — Additional file 7: Supplement 7. a. Posters. b. Landscapes - environment intervention. c. Flowers - environment intervention. d. Animals - environment intervention. e. - Famous people - environment intervention. [file 12877_2023_4285_MOESM7_ESM.zip › 12877_2023_4285_MOESM7_ESM/Supplement 7d - Animals - environment intervention.pdf]

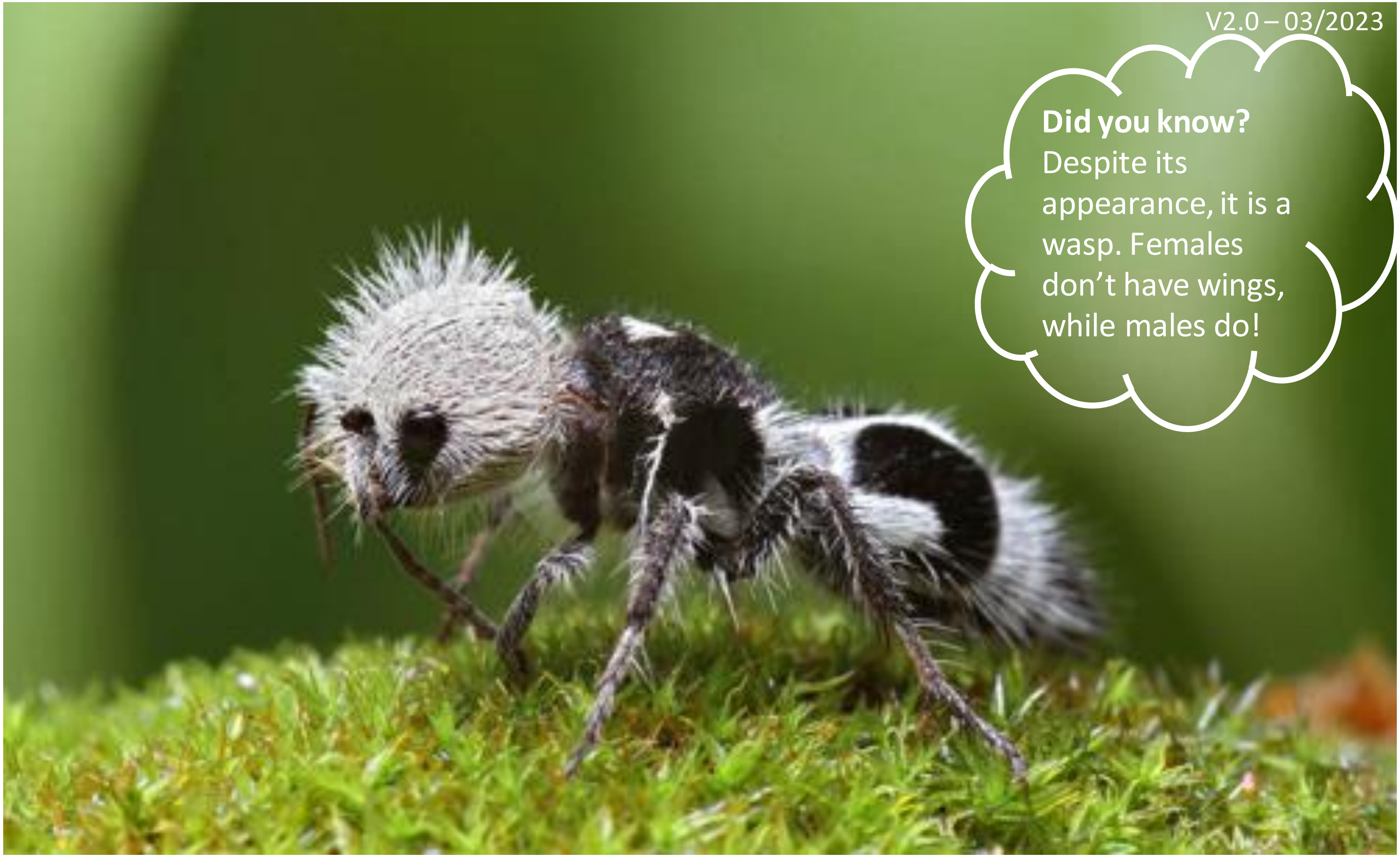

https://wikipedia.org

Panda ant from Chile

INTOMOB

V2.0 – 03/2023

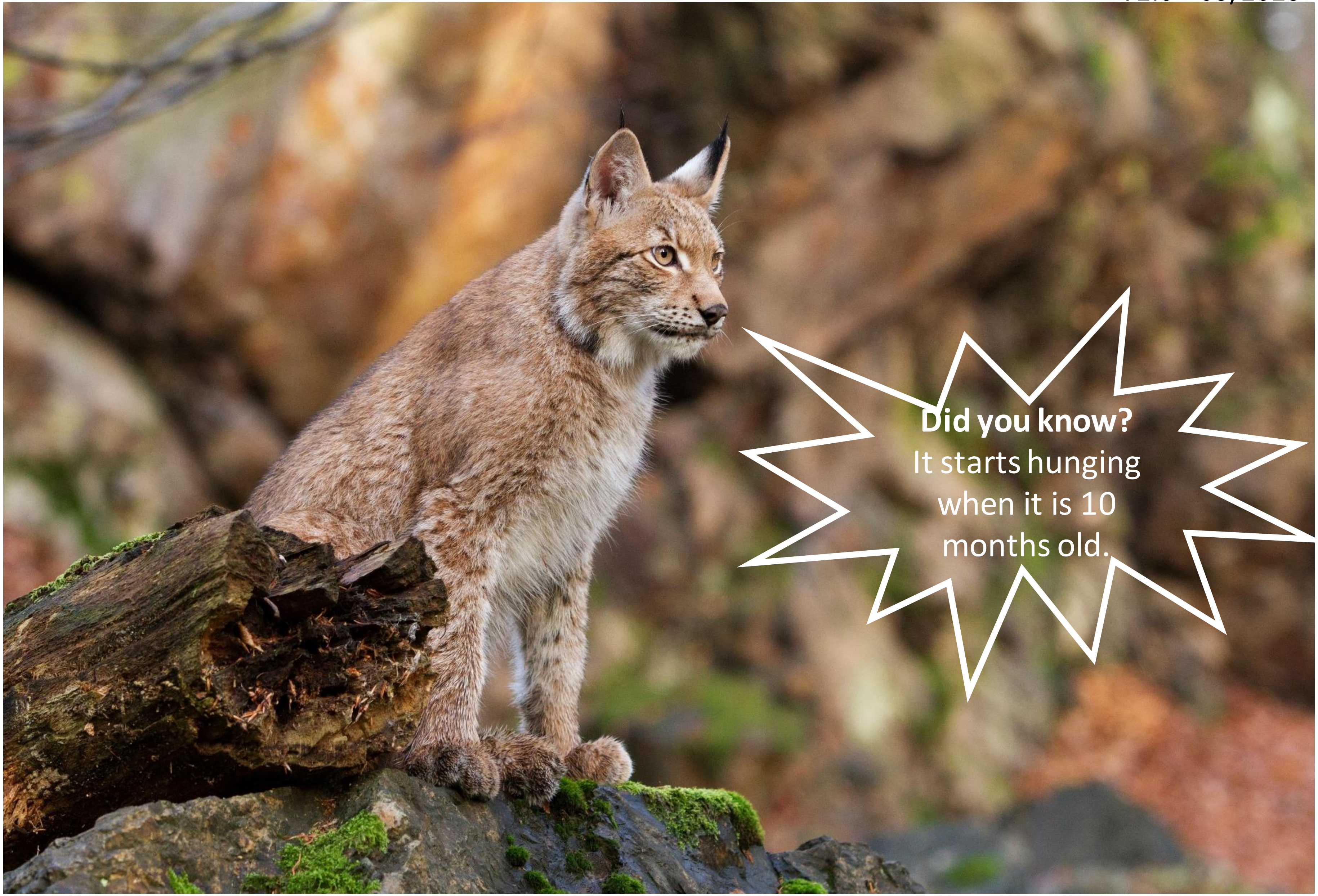

https://houseofswitzerland.org

Lynx

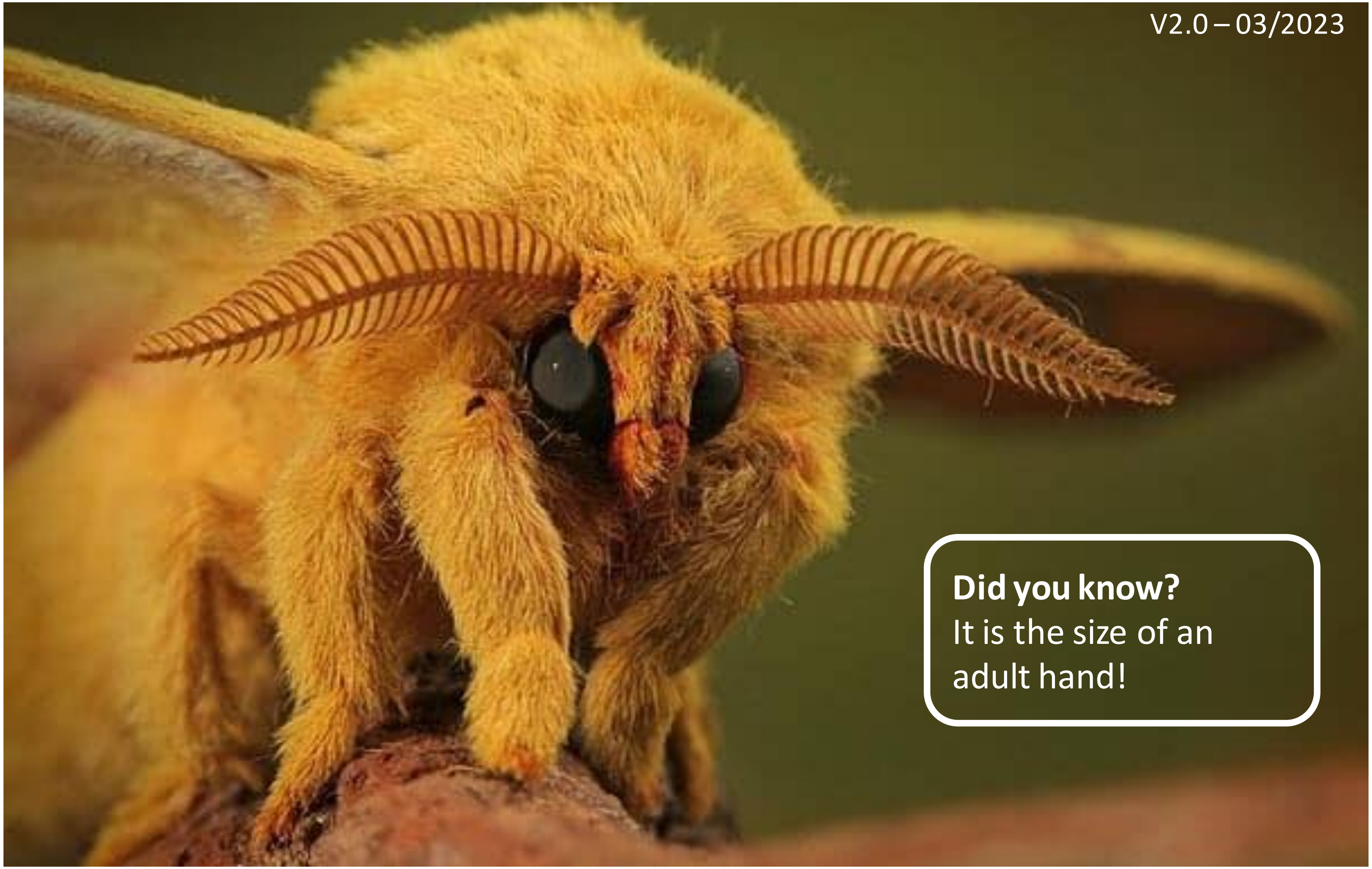

https://generationvoyage.fr/

Poodle-butterfly from Venezuela

INTOMOB

V2.0 – 03/2023

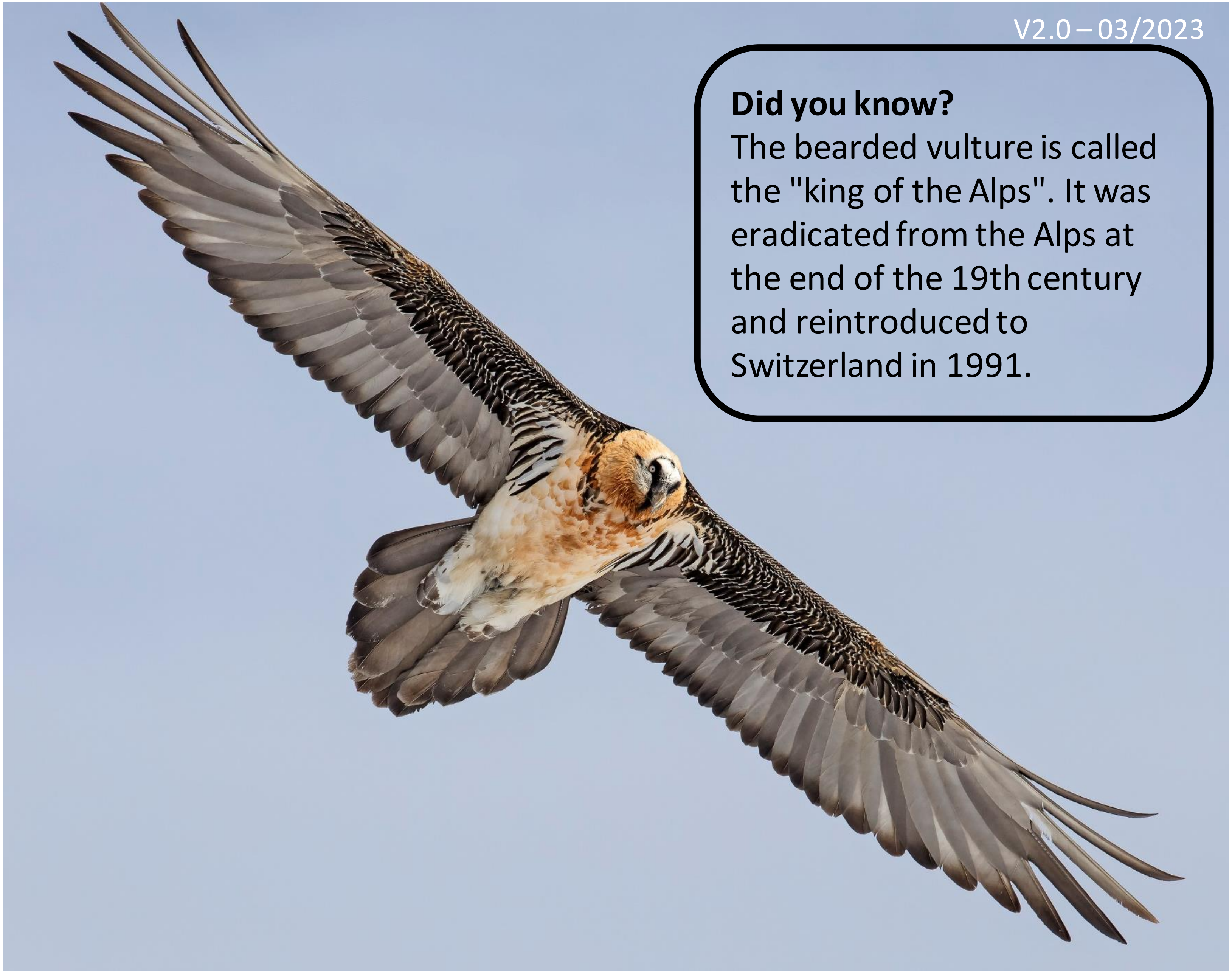

Did you know?
The bearded vulture is called the "king of the Alps". It was eradicated from the Alps at the end of the 19th century and reintroduced to Switzerland in 1991.

https://houseofswitzerland.org

Bearded vulture

INTOMOB
V2.0 – 03/2023

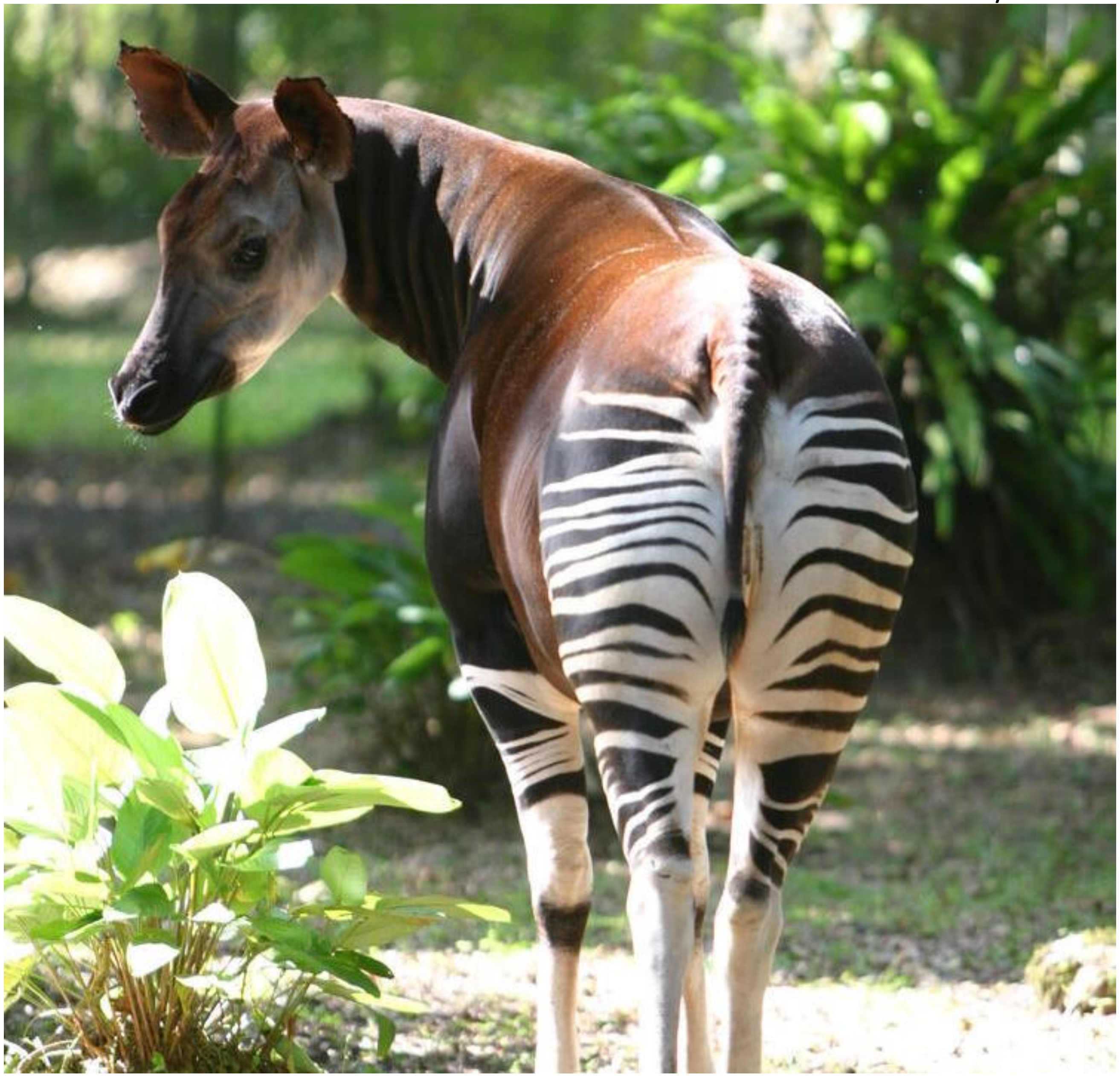

https://whc.unesco.org

Did you know?
About 5000 of the 30000 okapis live in their own reserve in the Congo.

Okapi

INTOMOB

V2.0 – 03/2023

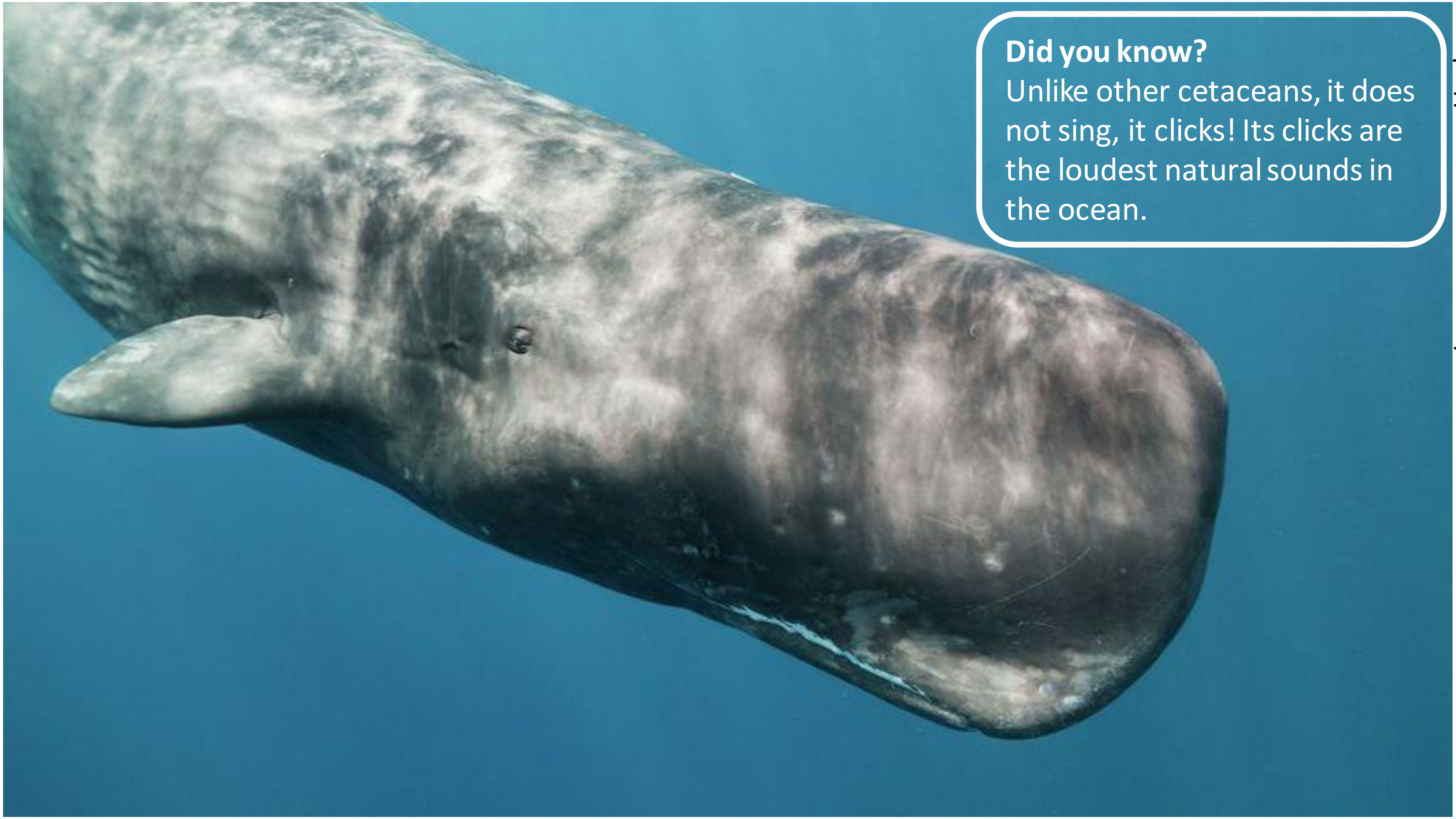

https://www.radiofrance.fr/

Sperm whale

https://generationvoyage.fr

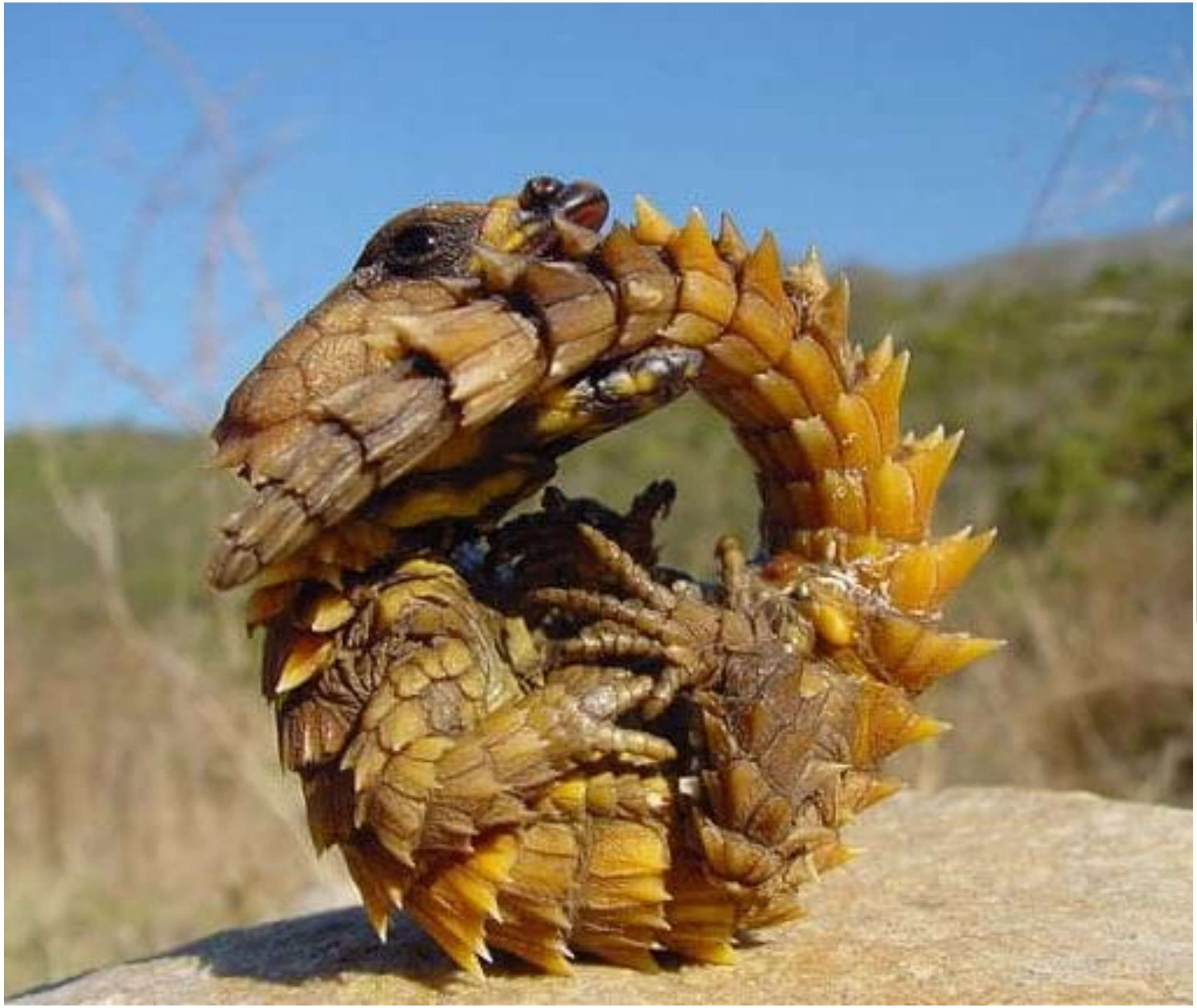

Did you know?
It lives mainly in Australia and eats almost exclusively ants.

Moloch horridus or thorny devil

INTOMOB
V2.0 – 03/2023

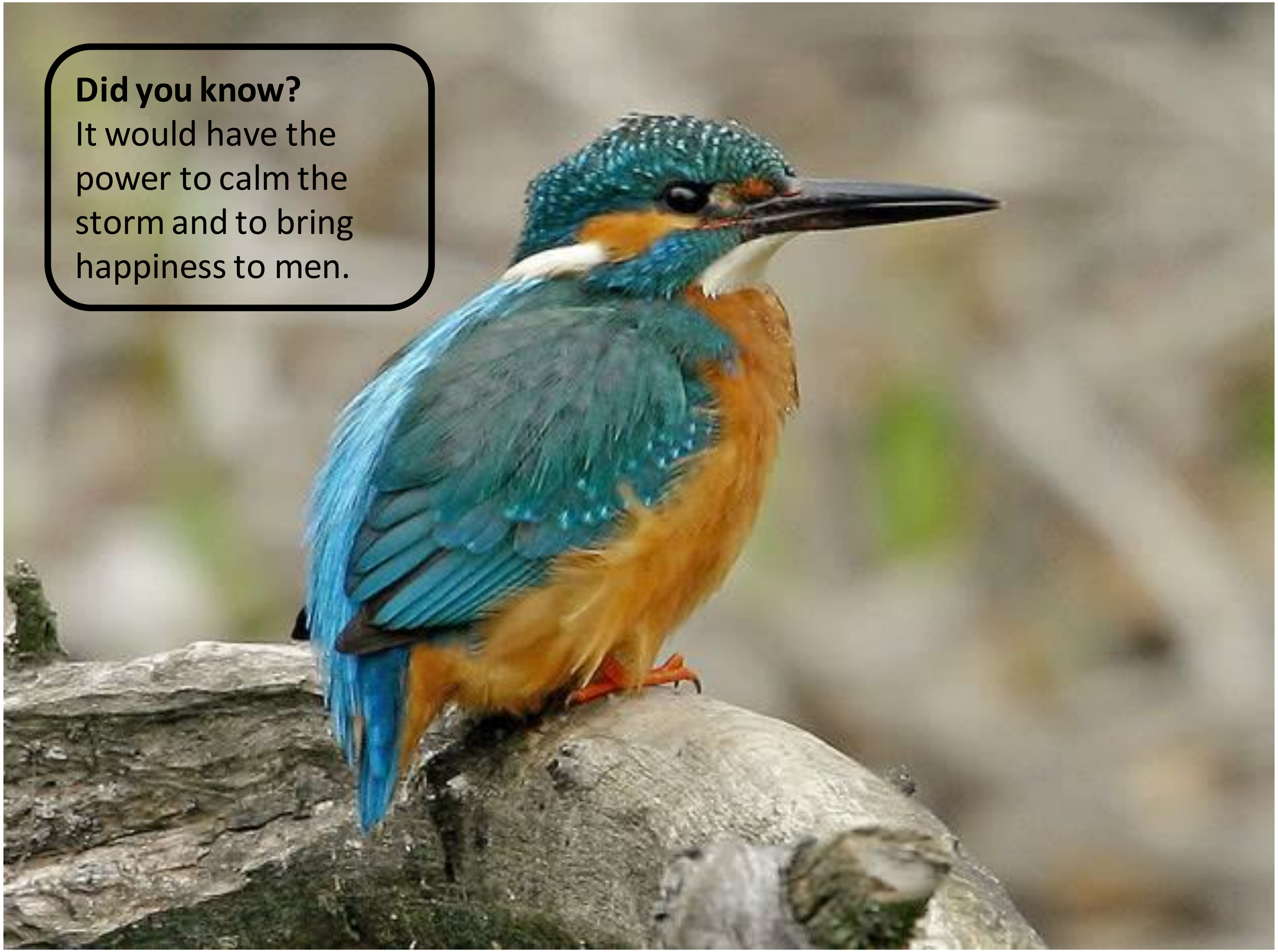

https://www.vogelwarte.ch/

Kingfisher

IVTOMOB

V2.0 – 03/2023

https://generationvoyage.fr/

Did you know?
Being a poor swimmer, it prefers to use its fins to walk on the seabed of the Atlantic.

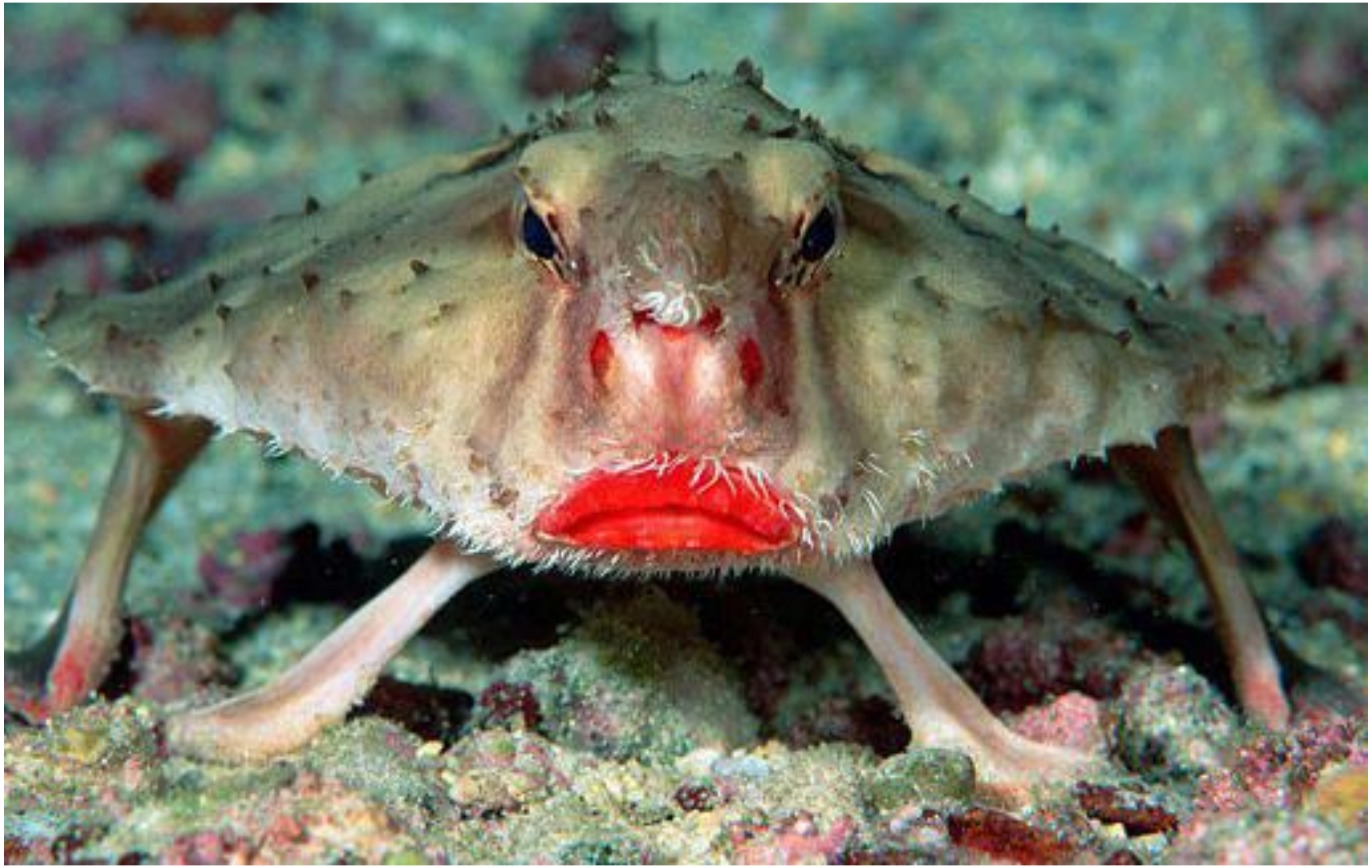

Red-lipped batfish

INTOMOB
V2.0 – 03/2023

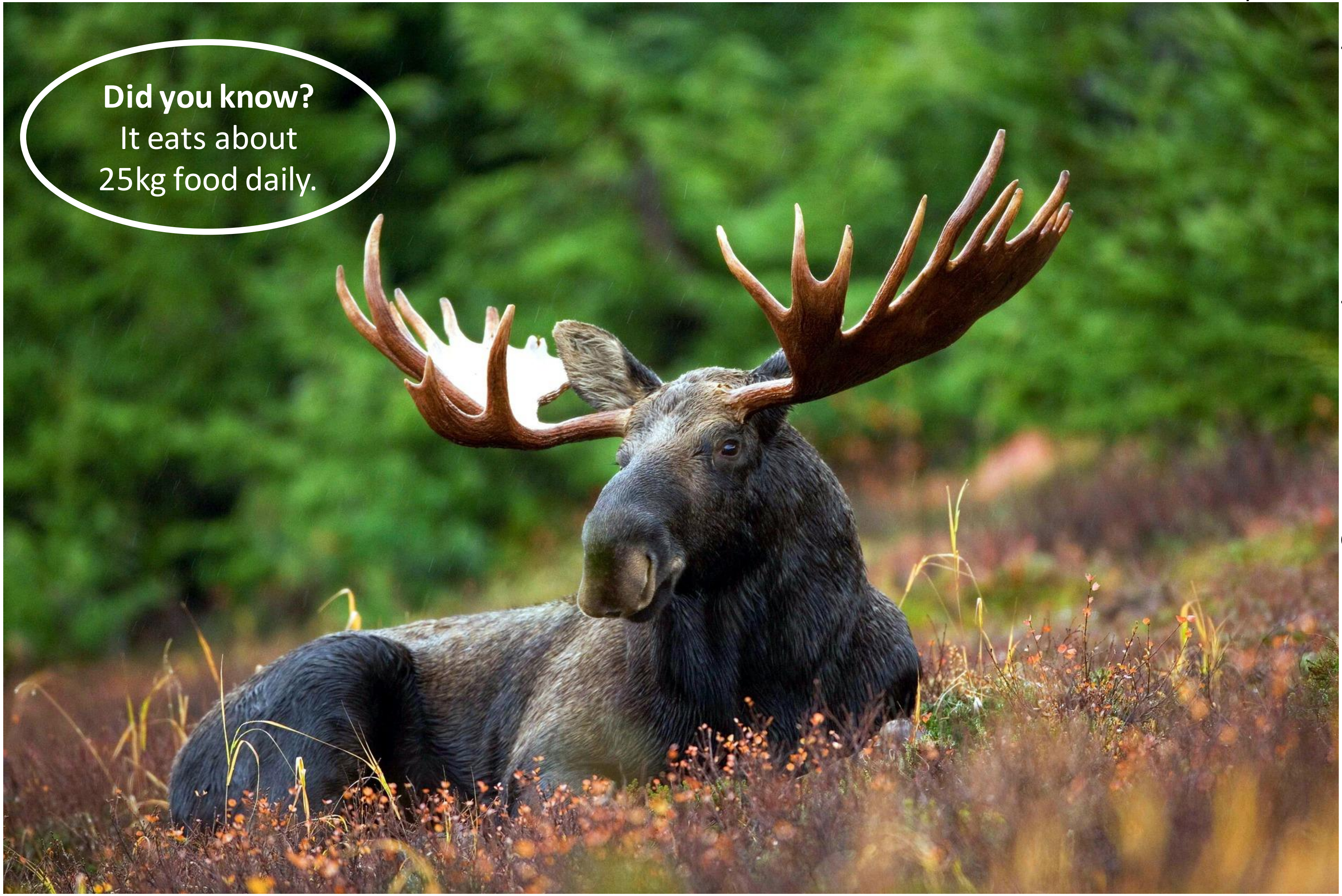

https://www.ensemblepourlesanimaux.org/

Eland
