# Supplementary material for: Impact of an INtervention to increase MOBility in older hospitalized medical patients (INTOMOB): Study protocol for a cluster randomized controlled trial
Source: BMC Geriatr. 2023 Oct 31;23:705. doi: 10.1186/s12877-023-04285-3 (PMC10617203; doi:10.1186/s12877-023-04285-3)
Supplement: Supplementary file 7 — Additional file 7: Supplement 7. a. Posters. b. Landscapes - environment intervention. c. Flowers - environment intervention. d. Animals - environment intervention. e. - Famous people - environment intervention. [file 12877_2023_4285_MOESM7_ESM.zip › 12877_2023_4285_MOESM7_ESM/Supplement 7e - Famous people - environment intervention. .pdf]

Did you know?
In 1992, he was the first Swiss person to go in space.

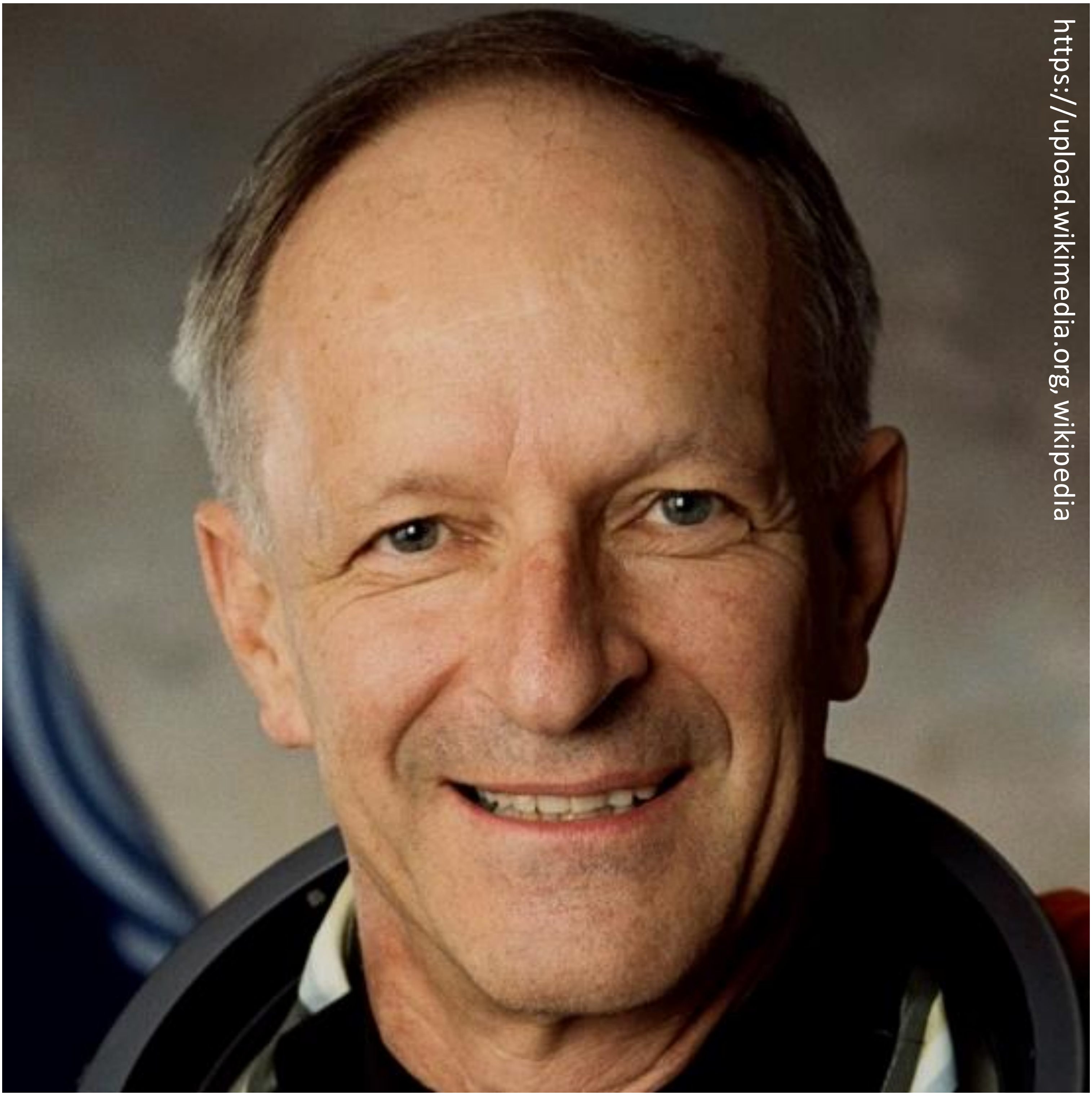

Claude Nicollier, astronaut

Did you know?
She has received over 50 honorary degrees.

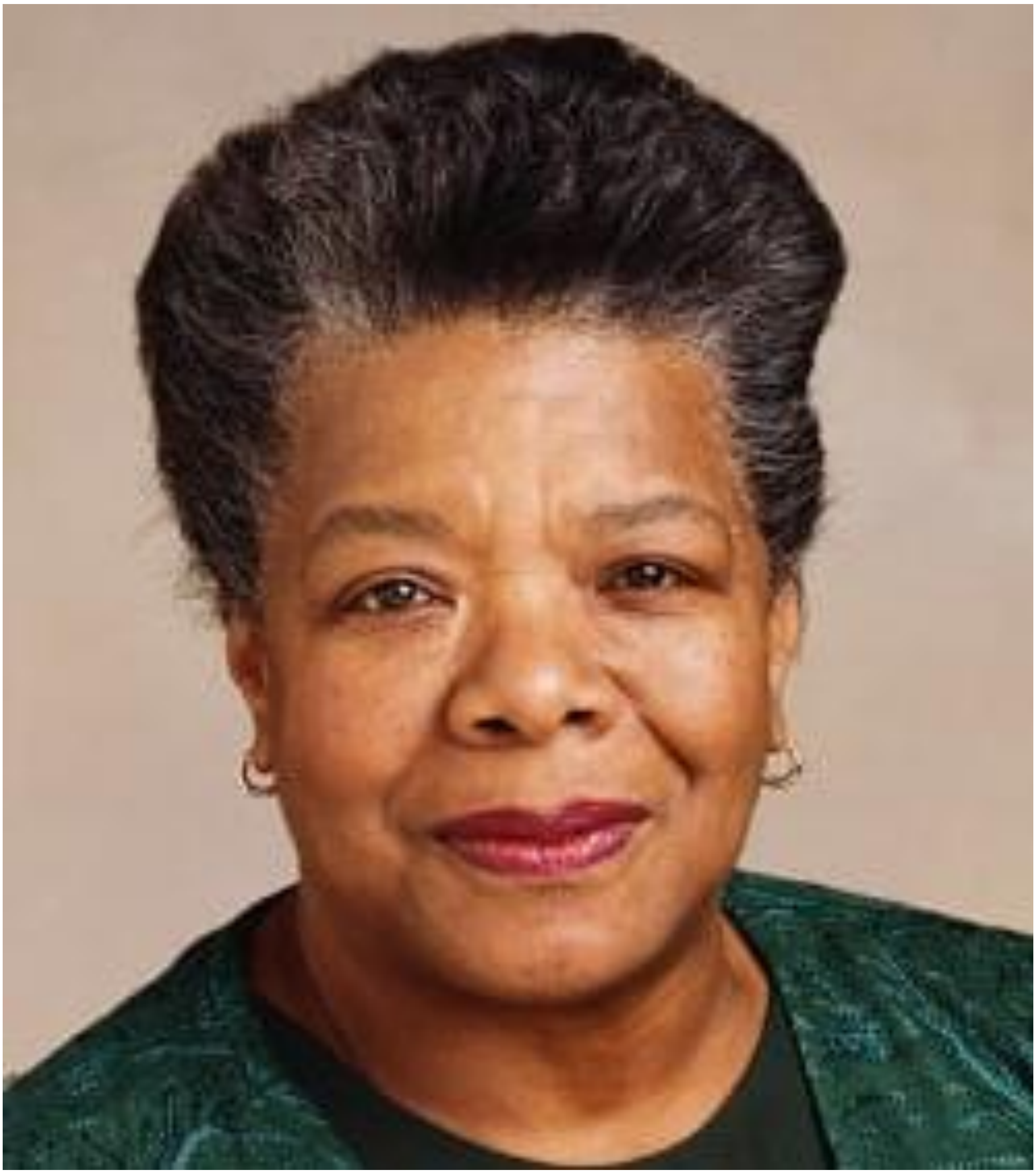

Maya Angelou, author and activist

Biography.com, wikipedia

Did you know?
She is the youngest female world number one in the history of tennis.

V2.0 – 03/2023

Shutterstock.com, wikipedia

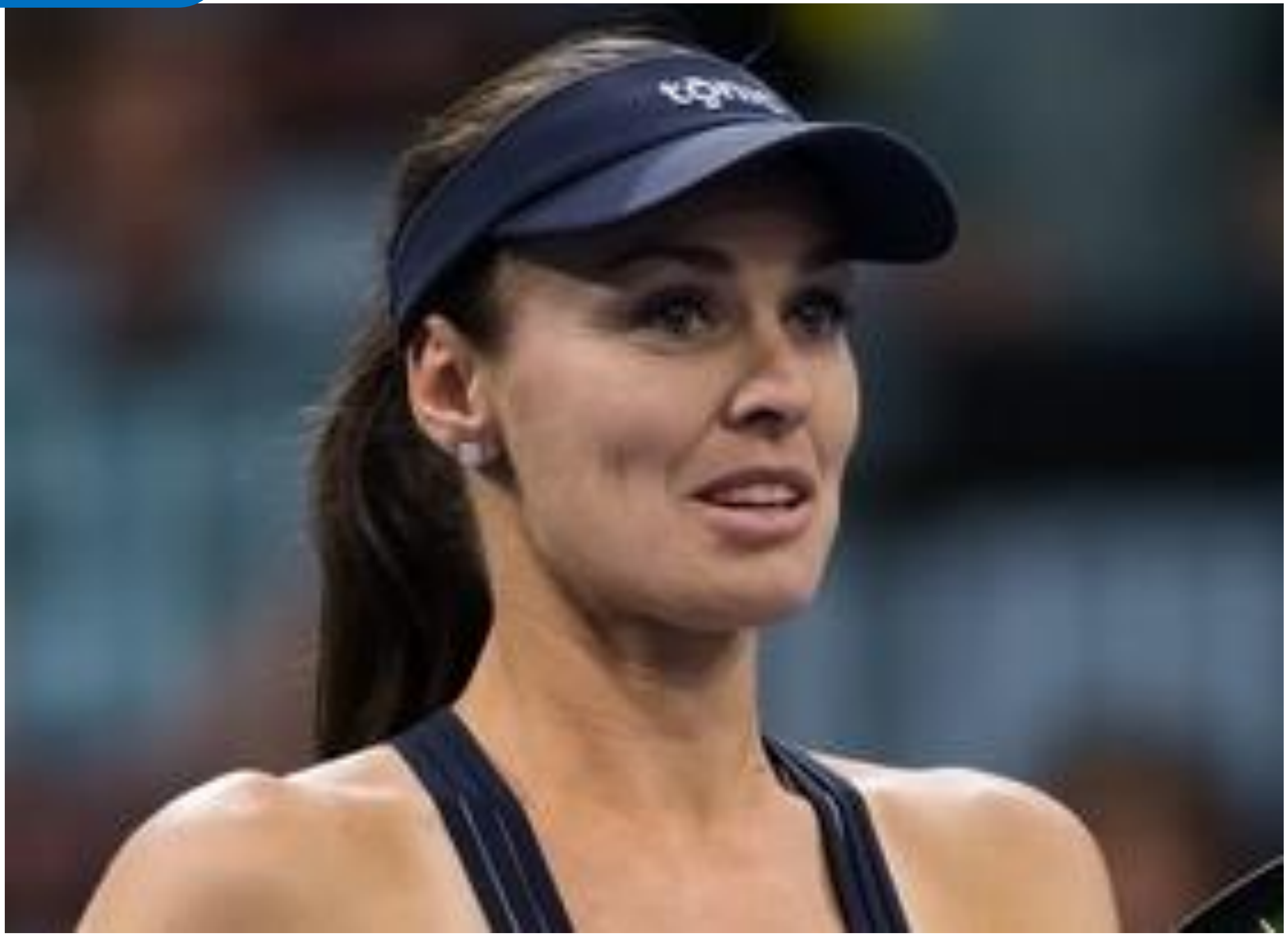

Martina Hingis, tennis player

Did you know?
The founder of analytical psychology, he later diverged from Freud's psychoanalytical work.

V2.0 – 03/2023

www1.wdr.de, wikipedia

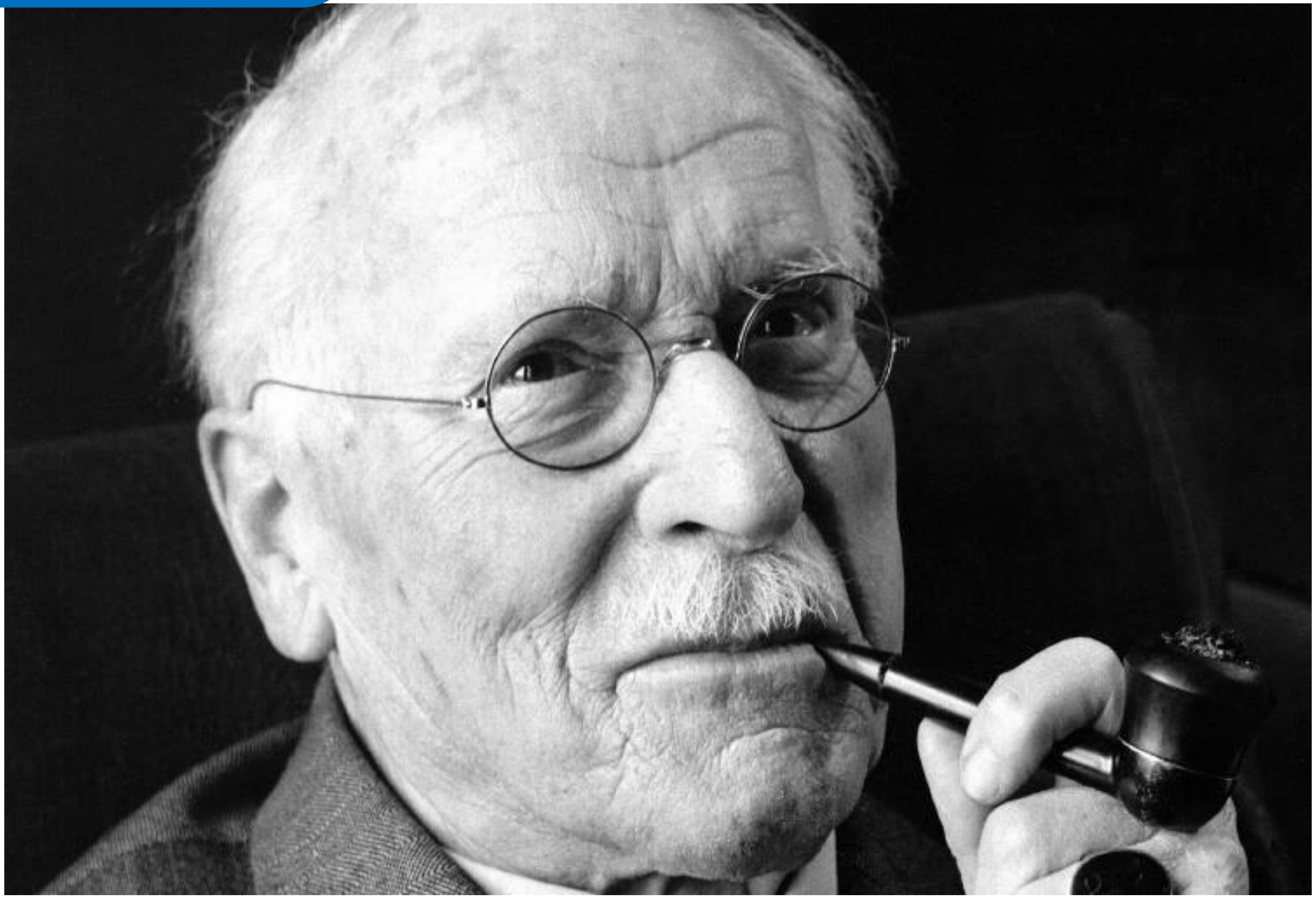

Carl Jung, psychiatrist

V2.0 – 03/2023

Did you know?
At the age of 7, he performed for the President of the United States.

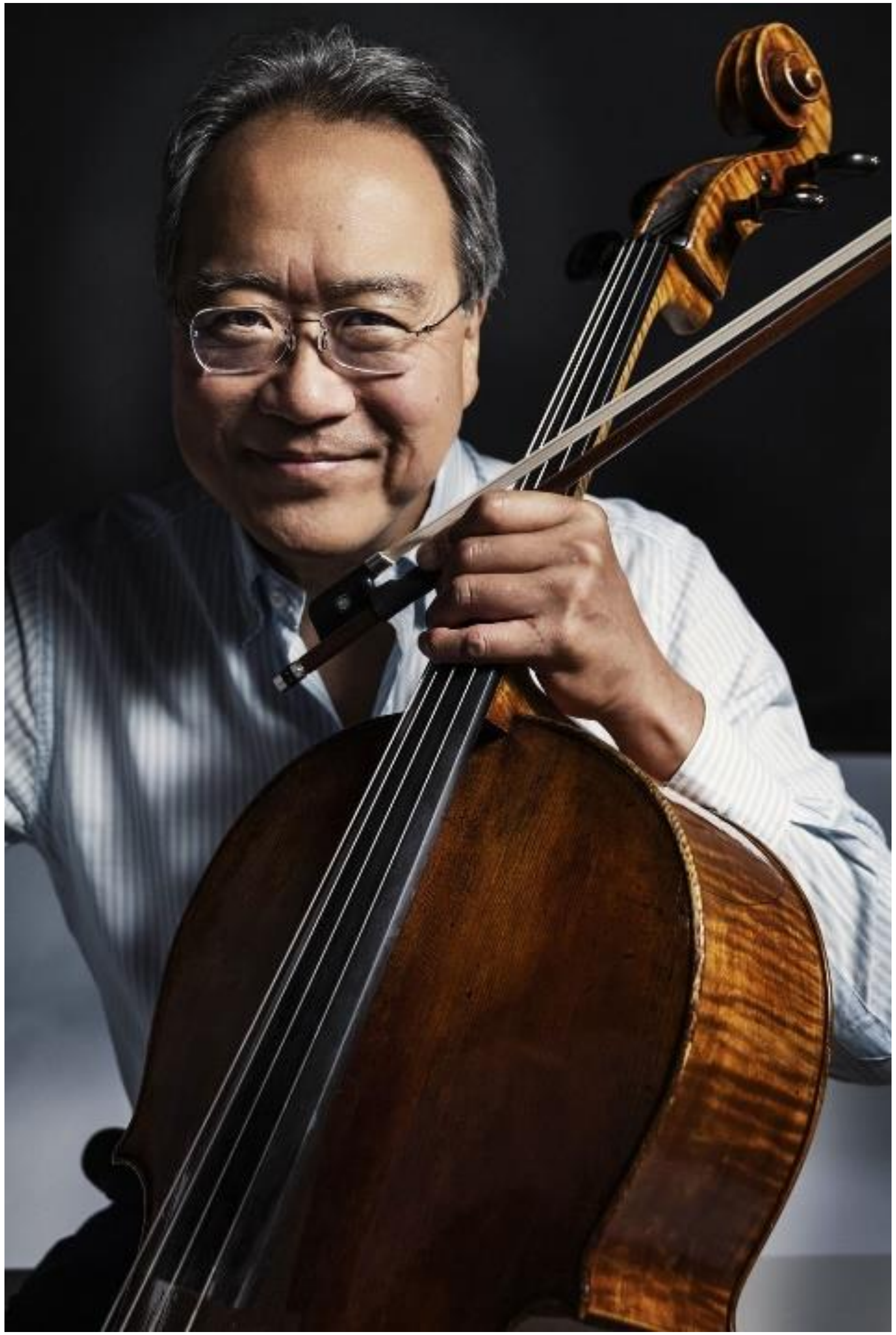

Yo-Yo Ma, cellist

www.harpersbazaar.com, wikipedia

Did you know?
A pioneer of resistance to oppression through mass civil disobedience, he helped lead India to independence.

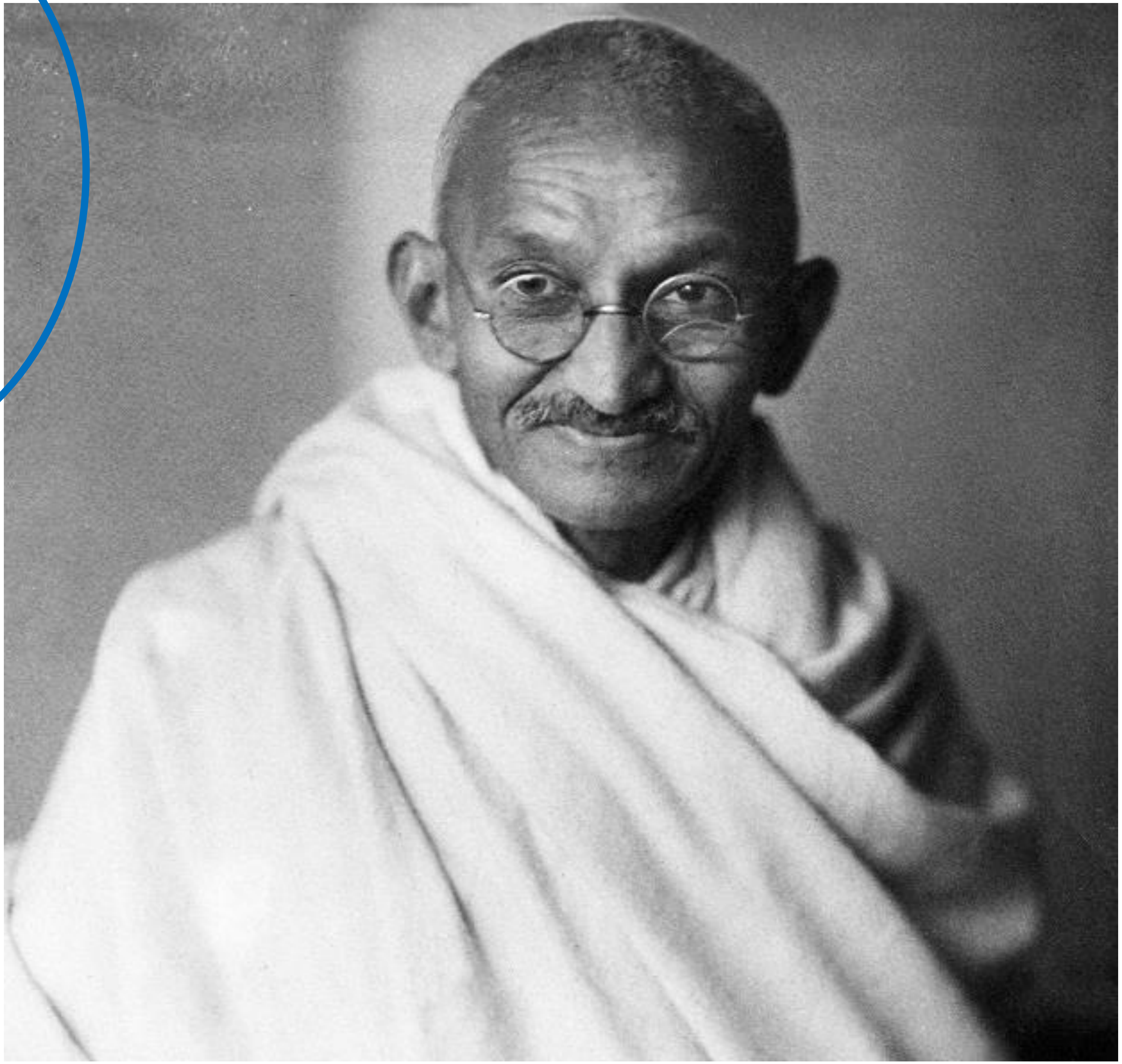

Mohandas Gandhi, political and spiritual guide

Wikipedia.org

INTOMOB
V2.0 – 03/2023

INTOMOB
V2.0 – 03/2023

Did you know?
She is committed to many causes, such as the fight against obesity, the rights of LGBT people or the education of young girls.

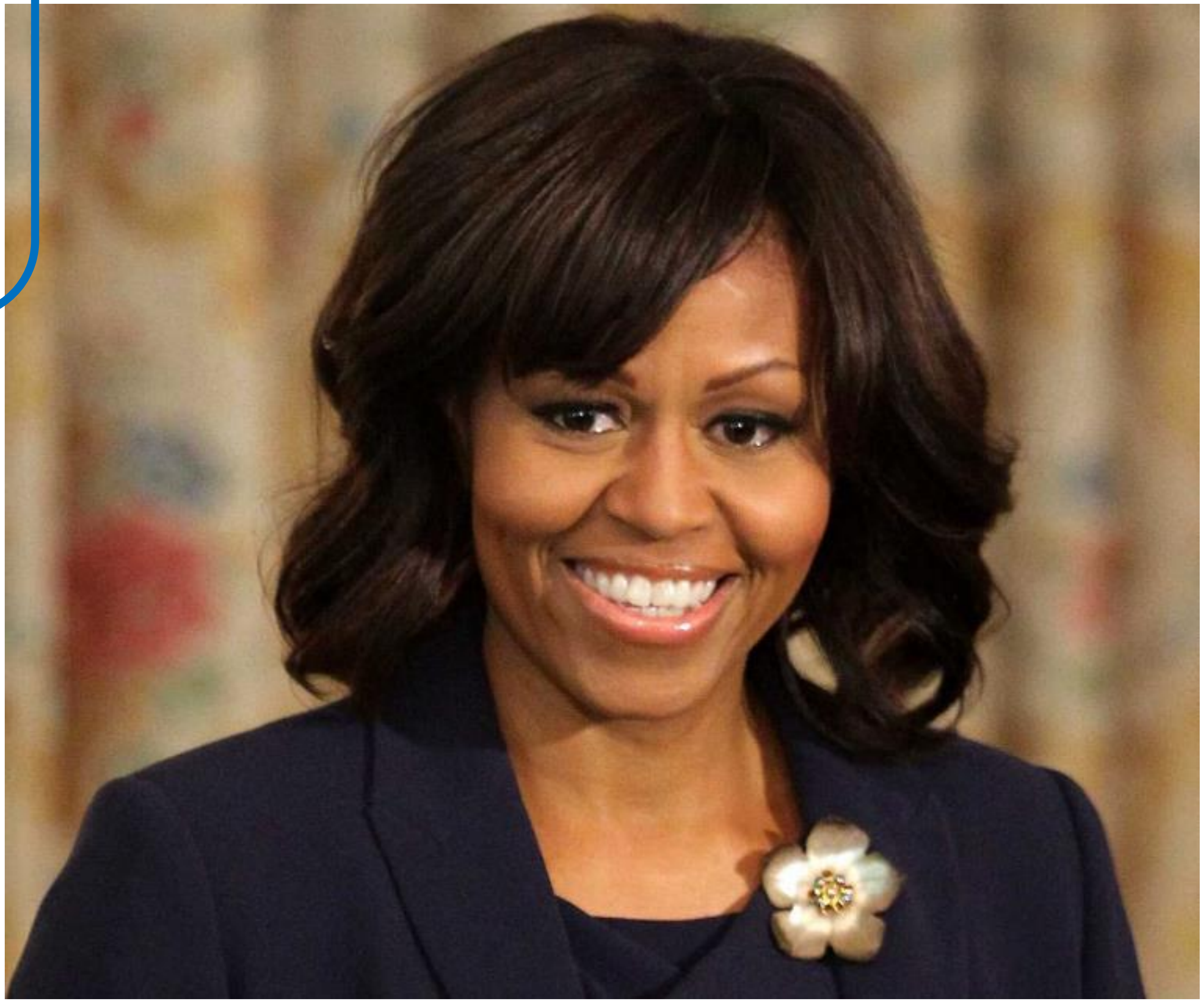

Gala.fr, wikipedia

Michelle Obama, lawyer and author

Did you know?
Between 1938 and 1944, his figures measure a maximum of 7cm: they must reflect the distance from which the sculptor saw the model.

INTOMOB
V2.0 – 03/2023

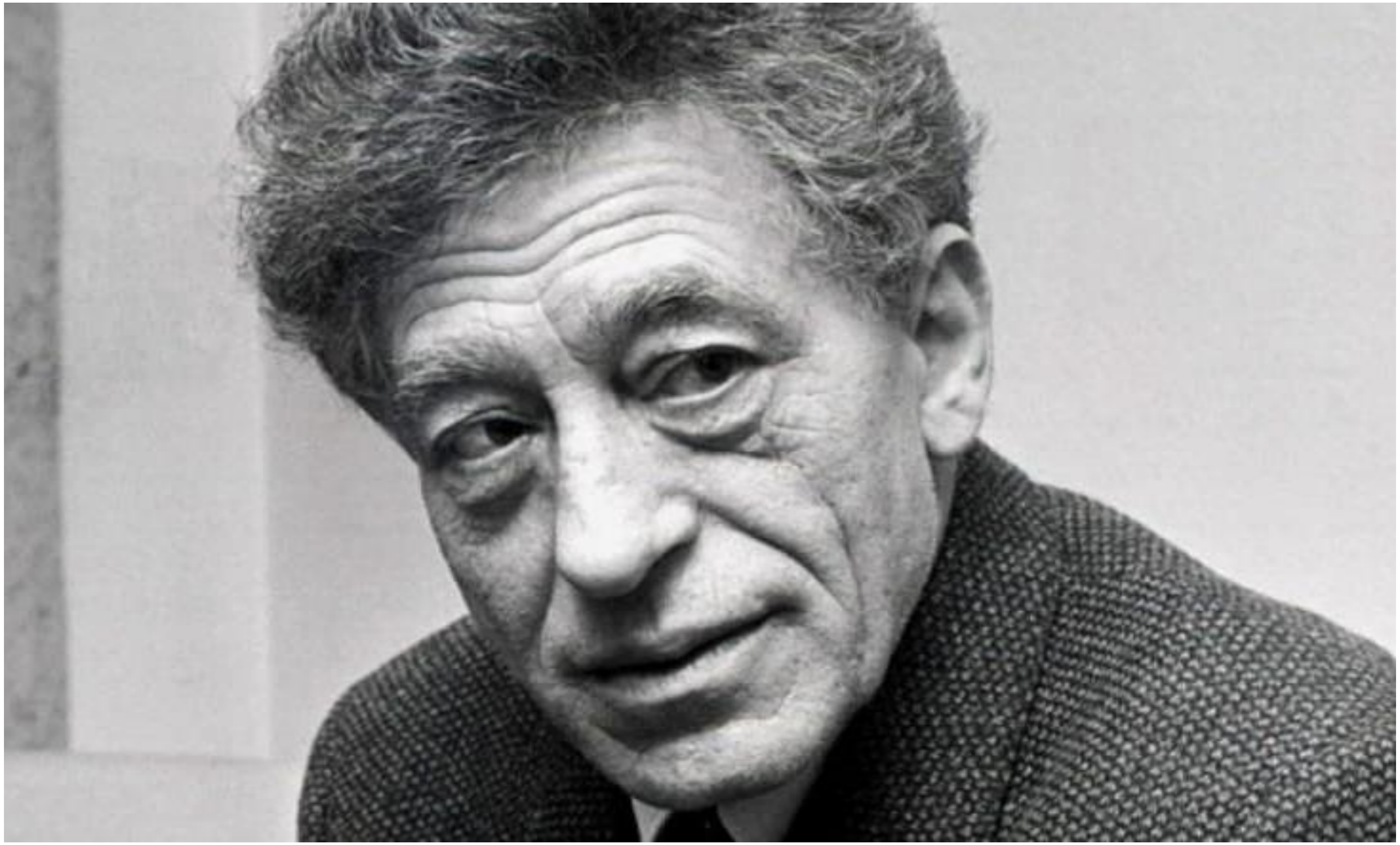

Alberto Giacometti, sculptor

https://www.srf.ch/audio, wikipedia

Did you know?
She was a prosecutor for the International Criminal Tribunal for the former Yugoslavia and for Rwanda.

INTOMOB
V2.0 – 03/2023

https://www.blick.ch, wikipedia

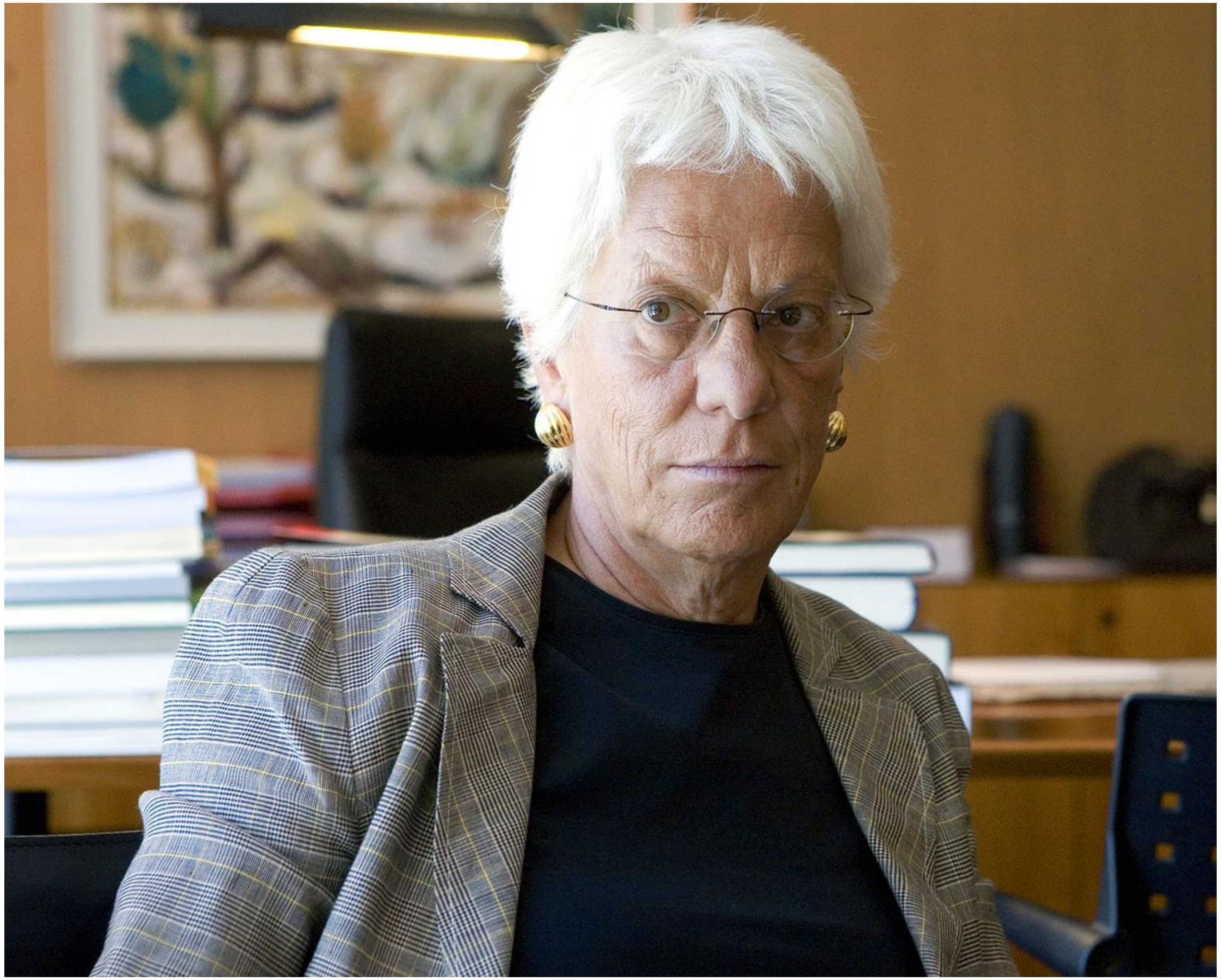

Carla Del Ponte, jurist and diplomate
