# Supplementary material for: Impact of an INtervention to increase MOBility in older hospitalized medical patients (INTOMOB): Study protocol for a cluster randomized controlled trial
Source: BMC Geriatr. 2023 Oct 31;23:705. doi: 10.1186/s12877-023-04285-3 (PMC10617203; doi:10.1186/s12877-023-04285-3)
Supplement: Supplementary file 8 — Additional file 8: Supplement 8. a. - ICF -control. b. - ICF - intervention. [file 12877_2023_4285_MOESM8_ESM.zip › Supplement 8a - ICF -control.pdf]

Request for participation to a medical research project:

Mobility of older hospitalized persons (INTOMOB study)

Dear Sir or Madam,

We would like to invite you to participate in our research project.

Your participation is completely voluntary. All data collected within this project are subject to strict data protection rules. This is a research project of the Insel Gruppe AG (sponsor[1]) under the direction of Dr. Carole E. Aubert, senior physician at the department of general internal medicine of Bern University Hospital. We will let you know the results if you wish.

We will present to you the main elements and answer your questions face-to-face. To give you an overview of the project, here are the key points to remember. You will find more detailed information on the study below.

Why are we conducting this research project?

- During a hospitalization, many things change, such as how and how much we move.
- Our research project aims to assess how much patients move during a hospitalization and how their mobility status changes in the 6 months following hospitalization.
- We plan to include 274 participants in the study.
- The study takes place in three hospitals.

What should I do, if I agree to participate? – What happens to me if I participate?

- Form and process of participation: If you agree to participate in our project, you will wear a pedometer on your wrist (a small device to measure movement) during your hospitalization. The study includes four visits during which we ask you questions (partly in the form of questionnaires) and examine you (visits 1 and 2 only): 1) in the hospital, shortly after your admission to the unit; 2) in the hospital, shortly before you are discharged; 3) by telephone about 1 month after your hospitalization; 4) by telephone about 6 months after your hospitalization. If we cannot complete the second visit before you are discharged from the hospital, we will call you by phone within two days of your discharge.
- Duration: the study lasts for you a total of 6 months.

Benefits for the participants

- It is not certain that you will benefit directly from your participation. Intervention to improve movement may help reduce the impact of lack of movement on you. Through your participation, you help us to obtain important knowledge to optimize the care of future patients.

Risks and constraints

- We do not expect any special risk related to this study.

By signing at the end of the document, you certify that you have understood all the contents and freely consent to take part in the project.

[1] The sponsor is a person or institution with headquarters or representative in Switzerland, who takes responsibility for the launch of a study, i.e., its initiation, management and financing.

Detailed information

1. Objective of the project and selection of participants

The aim of this study is to evaluate whether an intervention to improve movement of older people during hospitalization has a positive effect on the period after hospitalization.

We are asking you because participation is open to persons who are older than 60 years and are hospitalized on an internal medicine ward of a Swiss hospital.

2. General Information

If you participate in the study, you will receive a wrist-worn pedometer (a small device to quantify your movement) that you must wear during your hospitalization.

About 1 and 6 months after the study begins, we will contact you by phone to ask you a few questions. The study is then over for you.

The study is taking place in three hospitals. We plan to include 274 participants in the study.

The study is being conducted in accordance with the requirements of Swiss law. In addition, we follow all internationally recognized guidelines. The study has been reviewed and approved by the relevant ethics committee.

3. Procedures for participants

If you agree to participate in the study, an initial visit with questions and physical examination will take place on the unit where you are hospitalized (lasting approximately 1 hour). This visit includes the following:

- ✓ Verification of the inclusion and exclusion criteria for participating in the study.
- ✓ Questions about your person, e.g. height, weight, place of residence.
- ✓ Questions about your mobility and independence status.
- ✓ Questions about your mood and quality of life.
- ✓ Questions about a possible fear of falling.
- ✓ Physical examination to assess your mobility and muscle strength.
- ✓ We will give you the booklets, the mobility diary and the iPad (see section 2) and explain you how to use them.

Shortly before your discharge from the hospital, a second visit will take place in the unit where you are hospitalized, with questions and a physical examination (lasting approximately 1 hour). If we are unable to complete this visit before your discharge, we will contact you within two days of your discharge to complete it by phone. This is to avoid prolonging your hospitalization. This visit includes the following:

- ✓ Questions about your mobility status.
- ✓ Questions about a possible fear of falling.
- ✓ Questions about your satisfaction with your hospitalization.
- ✓ Questions on your experience of the intervention.
- ✓ Suggestion to improve the intervention.
- ✓ Physical examination to assess your mobility and muscle strength.

Approximately 1 and 6 months after the start of the study, we will contact you by phone to ask you some questions, including questions about your well-being, health, mobility status, where you live, and any hospitalizations. The phone calls last about 1 hour. Between your discharge from the hospital and these calls, we ask you to document any falls, emergency room visits and hospitalizations in a diary that we provide. If we are unable to reach you repeatedly, we will contact your next of kin or your general practitioner, so that we can ask them these questions.

What?	After signature of informed consent	Shortly before / after discharge	About 1 month after study start	About 6 months after study start
How – where?	On the ward	On the ward +/- phone call	Phone call	Phone call
What?	Questions & examination of strength & mobility	Questions & examination of strength & mobility	Questions	Questions
How long?	About 1 hour	About 1 hour	About 1 hour	About 1 hour

No additional blood tests or X-rays are needed for this study. You also do not have to stay in the hospital longer than planned.

We may have to exclude you from the study sooner than expected if the study is stopped earlier than planned. It is also possible that you may have to stop participating in the intervention sooner than expected, should a medical contraindication arise.

4. Benefits for participants

You will not get any direct benefit from your participation. Through your participation, you help us to obtain important knowledge to optimize the care of future patients.

5. Voluntary nature of participation and obligations

Your participation is completely voluntary. If you choose not to participate, or if you choose to participate and change your mind during the course of the study, you do not have to explain your decision. This will not change your medical care.

If you choose to participate in the study, you will be required to:

- Follow the instructions and meet the requirements of the research protocol.
- Report any new symptoms, new conditions, or changes in your condition to the investigator.

6. Risks and constraints for participants

We do not expect any specific risk related to this study.

7. Alternatives

You are entirely free to participate in this study. If you do not participate, your medical care remains unchanged.

8. Results

The investigator will notify you during the study of any important new findings affecting you. You will be informed orally and in writing, and you will be given the opportunity to decide again if you wish to continue to participate in the study.

The individual study results that we collect during the phone calls will be summarized at the end of the study as final results. At the end of the study, we can send you a summary of these overall results.

9. Data confidentiality

9.1. Data processing and coding

During this study, personal and health data are collected and processed, partly automatically. The data is coded. Coding means that all identifying information (name, date of birth, etc.) is replaced by a code. Only a few professionals can see your coded data, and then only to perform tasks related to the study. These people are bound by professional secrecy. As a participant, you have the right to see your data. It is not possible to link the data to your person without the code, which remains permanently within the institution conducting the study.

9.2. Data protection

All data protection guidelines are strictly adhered to. In the case of publications, the summarized data cannot be traced back to you as an individual. Your name will never appear on the internet or in a publication. In the case of publications, some scientific journals require that individual data (called "raw" data) be submitted. When individual data must be transmitted, the data are always coded. It cannot be traced back to you as an individual.

9.3. Data protection in case of data reuse

Your data could later help to answer other questions and be sent to another database located in Switzerland or abroad to be used in other research projects. However, these databases must comply with the same standards and requirements as the database of the present study. For this data reuse, we ask you to sign a separate declaration of consent at the end of this information sheet. This second consent is independent of your participation in the study.

9.4. Consultation right in the context of inspections

The study may be subject to inspection by the relevant ethics committee or by the sponsor who initiated the study. The investigator must then communicate your data for the purpose of these inspections. All persons involved are bound to the strictest professional secrecy.

10. Withdraw

You may withdraw from the study at any time if you wish. The data collected up to that point will remain in coded form in the database and will be analyzed in coded form. You must therefore agree to this before giving your consent.

11. Compensation

You will not get any compensation for your participation in this study. Your participation will have no financial consequences for you or your health insurance.

12. Responsibility

The sponsor who initiated the research project and is responsible for its conduction, is liable for any damage you may suffer in connection with the study. The conditions and procedure in this respect are governed by law. Although this study does not involve any foreseeable risk, the sponsor has taken out insurance to cover any damage for which it is liable.

If you suffer any damage as a result of your participation in the study, you should contact the responsible investigator mentioned in chapter 14 or another member of the research team.

13. Funding

This study is funded by the Swiss National Science Foundation.

14. Contact person

You can ask questions about the study at any time. If you have any doubts, concerns or emergencies during or after the study, you can contact the principal investigator.
