# Supplementary material for: Impact of an INtervention to increase MOBility in older hospitalized medical patients (INTOMOB): Study protocol for a cluster randomized controlled trial
Source: BMC Geriatr. 2023 Oct 31;23:705. doi: 10.1186/s12877-023-04285-3 (PMC10617203; doi:10.1186/s12877-023-04285-3)
Supplement: Supplementary file 8 — Additional file 8: Supplement 8. a. - ICF -control. b. - ICF - intervention. [file 12877_2023_4285_MOESM8_ESM.zip › Supplement 8b - ICF - intervention.pdf]

Request for participation to a medical research project:

Effects of an intervention to improve mobility of older hospitalized persons (INTOMOB study)

Dear Sir or Madam,

We would like to invite you to participate in our research project.

Your participation is completely voluntary. All data collected within this project are subject to strict data protection rules. This is a research project of the Insel Gruppe AG (sponsor[1]) under the direction of Dr. Carole E. Aubert, senior physician at the department of general internal medicine of Bern University Hospital. We will let you know the results if you wish.

We will present to you the main elements and answer your questions face-to-face. To give you an overview of the project, here are the key points to remember. You will find more detailed information on the study below.

Why are we conducting this research project?

- During a hospitalization, patients often do not move enough, which can have negative consequences, such as muscle loss, loss of autonomy, institutionalization or hospital readmissions.
- Our research project aims to assess whether an intervention to improve mobility during hospitalization allows to maintain previous abilities.
- We plan to include 274 participants in the study.
- The study takes place in three hospitals.

What should I do, if I agree to participate? – What happens to me if I participate?

- Form and process of participation: If you agree to participate in our project, you will participate during your hospitalization to an intervention to improve mobility. The intervention consists of recommendations and explanations specific to movement during hospitalization. We will provide you with an iPad with videos of mobilization exercises during your hospitalization. In addition, we ask you to wear a pedometer on your wrist (a small device to measure movement) during your hospitalization. The study includes four visits during which we ask you questions (partly in the form of questionnaires) and examine you (visits 1 and 2 only): 1) in the hospital, shortly after your admission to the unit; 2) in the hospital, shortly before you are discharged; 3) by telephone about 1 month after your hospitalization; 4) by telephone about 6 months after your hospitalization. If we cannot complete the second visit before you are discharged from the hospital, we will call you by phone within two days of your discharge.
- Duration: the study lasts for you a total of 6 months. The intervention to improve movement takes place during hospitalization only.

Benefits for the participants

- You will not get any direct benefit from your participation. Through your participation, you help us to obtain important knowledge to optimize the care of future patients.

[1] The sponsor is a person or institution with headquarters or representative in Switzerland, who takes responsibility for the launch of a study, i.e., its initiation, management and financing.

Risks and constraints

- We do not expect any special risk related to this study.

By signing at the end of the document, you certify that you have understood all the contents and freely consent to take part in the project.

Detailed information

1. Objective of the project and selection of participants

The aim of this study is to evaluate how much older hospitalized people move during hospitalization and how their mobility status changes after hospitalization. In addition, we are examining, among others, their muscle strength, as well as their autonomy, quality of life, psychological state and risk of falling.

We are asking you because participation is open to all persons who are older than 60 years and who are hospitalized on an internal medicine ward of a Swiss hospital. Persons who will most likely remain hospitalized for less than 3 days after the start of the study, or who have a significant limitation in terms of mobility (e.g. mobilization only in a wheelchair or medical indication to remain in bed) cannot participate.

2. General Information

We know that hospitalized patients usually move too little. Low mobility during hospitalization can quickly have negative consequences, such as muscle loss (5% per day spent in bed), bone loss, falls, confusion and constipation, which can result in loss of autonomy that can lead to nursing home placement and even increase mortality.

We have developed an intervention to help patients move more during hospitalization. The main goal is to maintain the level of autonomy prior to hospitalization. In this study, we want to examine whether this intervention can decrease the negative consequences of lack of movement.

If you participate in the study, you will receive:

1) A booklet with information about mobility and low mobility in the hospital, as well as concrete tips and suggestions to move more (for example: where to go, how and when to move), with the aim of increasing your movement.

2) A mobilization diary, where you can note, with the help of the nursing staff, your own mobilization objectives, as well as your results, possible difficulties and needs.

3) A booklet with mobilization exercises (photos with explanations) that you can perform while lying, sitting or standing.

4) An iPad that we provide you with during your hospitalization, with videos of the same exercises as in the exercise booklet (point 3).

Finally, we will ask you to wear a wrist-worn pedometer (a small device to quantify your mobilization) during the hospitalization.

The active participation (intervention) takes place only during your hospitalization.

Approximately 1 and 6 months after the start of the study, we will contact you by phone to ask you a few questions. The study is then over for you.

The study is taking place in three hospitals. We plan to include 274 participants in the study.

There is also a control group that does not receive any intervention and whose results will be compared with those of the intervention group. Allocation to either group is done by chance and by hospital unit; you are hospitalized in a unit where all patients who meet the criteria for participation in the study are approached to ask if they wish to participate.

This study is carried out in accordance with the requirements of Swiss law. In addition, we follow all internationally recognized guidelines. The study has been reviewed and approved by the relevant ethics committee.

A description of the study can also be found on the website of the Federal Office of Public Health: www.kofam.ch (SNCTP000005259).

3. Procedures for participants

If you agree to participate in the study, an initial visit with questions and physical examination will take place on the unit where you are hospitalized (lasting approximately 1 hour). This visit includes the following:

- ✓ Verification of the inclusion and exclusion criteria for participating in the study.
- ✓ Questions about your person, e.g. height, weight, place of residence.
- ✓ Questions about your mobility and independence status.
- ✓ Questions about your mood and quality of life.
- ✓ Questions about a possible fear of falling.
- ✓ Physical examination to assess your mobility and muscle strength.

Shortly before your discharge from the hospital, a second visit will take place in the unit where you are hospitalized, with questions and a physical examination (lasting approximately 1 hour). If we are unable to complete this visit before your discharge, we will contact you within two days of your discharge to complete it by phone. This is to avoid prolonging your hospitalization. This visit includes the following:

- ✓ Questions about your mobility status.
- ✓ Questions about a possible fear of falling.
- ✓ Questions about your satisfaction with your hospitalization.
- ✓ Physical examination to assess your mobility and muscle strength.

Approximately 1 and 6 months after the start of the study, we will contact you by phone to ask you some questions, including questions about your well-being, health, mobility status, where you live, and any hospitalizations. The phone calls last about 1 hour and 15 minutes (1-month visit) and 1 hour (6-month visit). Between your discharge from the hospital and these calls, we ask you to document any falls, emergency room visits and hospitalizations in a diary that we provide. If we are unable to reach you repeatedly, we will contact your next of kin or your general practitioner, so that we can ask them these questions.

Mobility intervention: During hospitalization only

What?	After signature of informed consent	Shortly before / after discharge	About 1 month after study start	About 6 months after study start
How – where?	On the ward	On the ward +/- phone call	Phone call	Phone call
What?	Questions & examination of strength & mobility	Questions & examination of strength & mobility	Questions	Questions
How long?	About 1 hour	About 1 hour	About 1:15 hour	About 1 hour

No additional blood tests or X-rays are needed for this study. You also do not have to stay in the hospital longer than planned.

We may have to exclude you from the study sooner than expected if the study is stopped earlier than planned.

4. Benefits for participants

It is not certain that you will benefit directly from your participation. Intervention to improve movement may help reduce the impact of lack of movement on you. Through your participation, you help us to obtain important knowledge to optimize the care of future patients.

5. Voluntary nature of participation and obligations

Your participation is completely voluntary. If you choose not to participate, or if you choose to participate and change your mind during the course of the study, you do not have to explain your decision. This will not change your medical care.

If you choose to participate in the study, you will be required to:

- Follow the instructions and meet the requirements of the research protocol.
- Report any new symptoms, new conditions, or changes in your condition to the investigator.

6. Risks and constraints for participants

We do not expect any specific risk related to this study.

7. Alternatives

You are entirely free to participate in this study. If you do not participate, you can ensure to move enough without additional support. Your care remains unchanged hospitalization.

8. Results

The investigator will notify you during the study of any important new findings affecting you. You will be informed orally and in writing, and you will be given the opportunity to decide again if you wish to continue to participate in the study.

The individual study results that we collect during the phone calls will be summarized at the end of the study as final results. At the end of the study, we can send you a summary of these overall results.

9. Data confidentiality

9.1. Data processing and coding

During this study, personal and health data are collected and processed, partly automatically. The data is coded. Coding means that all identifying information (name, date of birth, etc.) is replaced by a code. Only a few professionals can see your coded data, and then only to perform tasks related to the study. These people are bound by professional secrecy. As a participant, you have the right to see your data. It is not possible to link the data to your person without the code, which remains permanently within the institution conducting the study.

9.2. Data protection

All data protection guidelines are strictly adhered to. In the case of publications, the summarized data cannot be traced back to you as an individual. Your name will never appear on the internet or in a publication. In the case of publications, some scientific journals require that individual data (called "raw" data) be submitted. When individual data must be transmitted, the data are always coded. It cannot be traced back to you as an individual.

9.3. Data protection in case of data reuse

Your data could later help to answer other questions and be sent to another database located in Switzerland or abroad to be used in other research projects. However, these databases must comply with the same standards and requirements as the database of the present study. For this data reuse, we ask you to sign a separate declaration of consent at the end of this information sheet. This second consent is independent of your participation in the study.

9.4. Consultation right in the context of inspections

The study may be subject to inspection by the relevant ethics committee or by the sponsor who initiated the study. The investigator must then communicate your data for the purpose of these inspections. All persons involved are bound to the strictest professional secrecy.

10. Withdraw

You may withdraw from the study at any time if you wish. The data collected up to that point will remain in coded form in the database and will be analyzed in coded form. You must therefore agree

to this before giving your consent.

11. Compensation

You will not get any compensation for your participation in this study. Your participation will have no financial consequences for you or your health insurance.

12. Responsibility

The sponsor who initiated the research project and is responsible for its conduction, is liable for any damage you may suffer in connection with the study. The conditions and procedure in this respect are governed by law. Although this study does not involve any foreseeable risk, the sponsor has taken out insurance to cover any damage for which it is liable.

If you suffer any damage as a result of your participation in the study, you should contact the responsible investigator mentioned in chapter 14 or another member of the research team.

13. Funding

This study is funded by the Swiss National Science Foundation.

14. Contact person

You can ask questions about the study at any time. If you have any doubts, concerns or emergencies during or after the study, you can contact the principal investigator.
