# Supplementary material for: Impact of an INtervention to increase MOBility in older hospitalized medical patients (INTOMOB): Study protocol for a cluster randomized controlled trial
Source: BMC Geriatr. 2023 Oct 31;23:705. doi: 10.1186/s12877-023-04285-3 (PMC10617203; doi:10.1186/s12877-023-04285-3)
Supplement: Supplementary file 9 — Additional file 9: Supplement 9. Perspectives on mobility survey. [file 12877_2023_4285_MOESM9_ESM.pdf]

Supplement 9a. HAPA items of the survey for patients and clinicians.

VARIABLES	PATIENTS	CLINICIANS
**Self-efficacy**	I am sure that I can move as much as possible during a hospital stay, even if it becomes sometimes hard.	I am sure that I can ensure that my patients move as much as possible, even if it is sometimes difficult (for example because of lack of time).
	I am sure that I can move as much as possible during a hospital stay, even if I have to request a lot of support from the healthcare providers.	I am sure that I can ensure that my patients move as much as possible.
	I am sure that I can move as much as possible during a hospital stay, even if I have to boost myself.	I am sure that I can ensure that my patients move as much as possible, even if I must think carefully when I manage to do so during my work day.
	I am sure that I can move as much as possible during a hospital stay, even if I do <u>not</u> immediately notice positive changes.	I am sure that I can ensure that my patients move as much as possible, even if I have to give myself a push.
	I am sure that I can move as much as possible during a hospital stay, even if I would prefer doing something else.	I am sure that I can ensure that my patients move as much as possible, even if once I do not feel sure (=competent).
	I am sure that I can move as much as possible during a hospital stay, even if my complaints/pain are a barrier.	After starting to ensure that my patients move as much as possible, I am confident that I will continue to manage to do so.
	I am sure that I can move as much as possible during a hospital stay, even if I have to overcome myself each time.	I am sure that I can ensure over the long term that my patients move as much as possible, even if I do <u>not</u> immediately notice progress by my patients.
	I am sure that I can move as much as possible during a hospital stay, even if I have little visits and exams do not let me much time available.	I am sure that I can ensure over the long term that my patients move as much as possible, even if I sometimes feel sorry for my patients when they suffer during mobilization.
	I am sure that I can move as much as possible during a hospital stay, even if I gave up several times.	I am sure that I ensure over the long term that my patients move as much as possible, even if I would actually prefer to do something else.
		I am sure that I can ensure over the long term that my patients move as much as possible, even if I have to adapt my priorities / work organization.
		I am sure that I can ensure over the long term that my patients move as much as possible, even if I have to overcome myself each time.
		I am sure that I can ensure over the long term that my patients move as much as possible, even if this increases my workload.
		I am confident that I can once again ensure that my patients move as much as possible n, if I once did <u>not</u> manage to do so (for example because of lack of time).
		I am confident that I can again ensure that my patients move as much as possible, even if some day I do <u>not</u> have time.
		I am confident that I can again ensure that my patients move as much as possible, even if once I can<u>not</u> pick myself up.
		I am confident that I can again ensure that my patients move as much as possible, even if several times I did <u>not</u> manage to do so (for example because of lack of time).
**Outcome expectancies**	If I move as much as possible during a hospital stay, I do something good for my health.	If I ensure that my patients move as much as possible, I do something good for my patients' health.
	If I move as much as possible during a hospital stay, it always requires self-discipline.	If I ensure that my patients move as much as possible, it has positive effects on psychical health of patients.

	If I move as much as possible during a hospital stay, it makes me self-confident.	If I ensure that my patients move as much as possible, my patients can go back to their everyday independent life.
	If I move as much as possible during a hospital stay, it impacts positively my mental state.	If I ensure that my patients move as much as possible, I am satisfied with my work.
	If I move as much as possible during a hospital stay, I always have to make a big effort.	
	If I move as much as possible during a hospital stay, I am a burden for the healthcare providers.	
	If I move as much as possible during a hospital stay, I feel better afterwards.	
	If I move as much as possible during a hospital stay, I can leave hospital earlier.	
**Risk perception**	I believe that my health will worsen if I do not move as much as possible during a hospital stay.	I believe that the health status of my patients can worsen if I do not ensure that they move as much as possible.
	I believe that I will stay longer at hospital if I do not move as much as possible during hospitalization.	I believe that my patients must stay longer at hospital if I do not ensure that they move as much as possible.
	I believe that I will become dependent on other people / institutions / walking aids to do my activities of daily living if I do not move as much as possible during hospitalization.	I believe that my patients will become more dependent in their everyday activities if I do not ensure that they move as much as possible.
**Intention**	I intend to move as much as possible during a future hospitalization.	I want to ensure during the next 3 months that my patients move as much as possible during their hospitalization.
**Planning**	NA	I have already concretely planned, when (during my work day) I will ensure that my patients move.
		I have already concretely planned, how I will ensure that my patients move.
		I have already concretely planned, where I will ensure that my patients move.
		I have already concretely planned, how often I will ensure that my patients move.
		I have already concretely planned, with whom I will ensure that my patients move.
		I have already concretely planned, how I will ensure that my patients move, even if once I do not feel enough competent.
		I have already concretely planned, how I will ensure that my patients move, even if several times I did not manage to do so (for example because of lack of time).
		I have already concretely planned, how I will ensure that my patients move when the workload is high.
		I have already concretely planned, how I will ensure that my patients move, even if once something acute happens in-between.
**Action control**[a] *Cronbach's α* (patients): 0.83 *Cronbach's α* (clinicians): 0.86	I kept in mind my intention to move during my hospitalization.	I kept in mind my intention to ensure that my patients move.
	During my hospitalization, I paid attention (thought) to move as much as I intended to.	I paid close attention (thought) to as I had planned to ensure that my patients move.
	During my hospitalization, I did anything (behavior) to move as I intended to.	I did everything (behavior) as I had planned to ensure that my patients move.

**Behavior**	I moved every day during my hospitalization.	I ensured that my patients move during hospitalization.
	I moved every day as much as possible during my hospitalization.	I ensured every day that my patients move during hospitalization.
		I ensured as much as possible that my patients move during hospitalization.
		I ensured every day as much as possible that my patients move during hospitalization.

Abbreviations: HAPA, Health Action Process Approach; NA, not applicable (not collected).
Legend: For items in the past, patients were asked to think about their last hospitalization on a medical ward. For items related to the future / long term, participants were asked to think about the next 3 months. Negatively formulated items are highlighted in grey boxes. They were recoded so that all items of a same variable were positively coded (1=less optimal, 5=best).
[a] Additional answer possible: "I had no intention". [b] Additional answer possible: "Not applicable".

Supplement 9b. Non-HAPA items of the survey for patients and clinicians.

NON-HAPA VARIABLES	PATIENTS	CLINICIANS
**Factual knowledge**	I know the consequences of low hospital mobility.	I know the mobility capacities of my patients (= what they are able to do with / without help).
	During a hospital stay, medication or other treatments are more important than moving.	I know the consequences of low patient mobility during a hospitalization.
		I know the contraindications and indications to mobilize my patients.
		Patients who were inactive or dependent in their activities of daily living before admission do not need to move during their hospitalization.
		It is more important to prevent falls during mobilization than to encourage patient mobility.
		Other work tasks are more important than patients' mobilization.
		Mobilization of patients is part of their treatment.
**Action knowledge**	I knew how to handle medical devices (for example urinary catheter, infusion) when I wanted to move.	I know how to advise my patients regarding hospital mobility.
	I knew whether I was allowed to move.	
	I knew when I was allowed to move.	
	I knew where I could move.	
	I knew who to ask to get support to move.	
**Role perception**	I am self-responsible to move during a hospitalization.	Ensuring my patients move is part of my work tasks.
**Fear**	Fear (for example of hurting myself or of not finding the way back) was a barrier to move.	I fear that my patients fall / hurt themselves during mobilization.
		I fear consequences if my patients hurt themselves during mobilization.
		I fear complications that could happen to my patients because of lack of movement.
		I fear to hurt myself when I mobilize my patients.
**Organization / environnement**	Healthcare providers did **not** have time to help me move.	On my department / unit, it is clear, who is responsible for patients' mobilization.
	I received a timetable of my exams / visits.	On my department / unit, patients' mobilization is standardized (algorithms, schemes, responsibilities, …).
	Lack of sitting / resting spots was a barrier to move.	

Abbreviations: HAPA, Health Action Process Approach; NA, not applicable (not collected).
Legend: For items in the past, patients were asked to think about their last hospitalization on a medical ward. For items related to the future / long term, participants were asked to think about the next 3 months. Negatively items that are highlighted in grey boxes were recoded so that all items of a same variable were positively coded (1=less optimal, 5=best).
